# Trends in Healthcare Access in Japan during the First Wave of the COVID-19 Pandemic, up to June 2020

**DOI:** 10.3390/ijerph18063271

**Published:** 2021-03-22

**Authors:** Koji Makiyama, Takayuki Kawashima, Shuhei Nomura, Akifumi Eguchi, Daisuke Yoneoka, Yuta Tanoue, Yumi Kawamura, Haruka Sakamoto, Stuart Gilmour, Shoi Shi, Kentaro Matsuura, Shinya Uryu, Masahiro Hashizume

**Affiliations:** 1 HOXO-M Inc., 1-22-11 Ginza, Chuo-ku, Tokyo 104-0061, Japan; hoxo.smile@gmail.com (K.M.); ceiiinosssttuv@gmail.com (K.M.); 2 Department of Mathematical and Computing Science, Tokyo Institute of Technology, 2-12-1 Ookayama, Meguro-ku, Tokyo 152-8550, Japan; kawashima@c.titech.ac.jp; 3 Department of Health Policy and Management, School of Medicine, Keio University, 35 Shinanomachi, Shinjyuku-ku, Tokyo 160-8582, Japan; amiyumiuni@gmail.com (Y.K.); harukask1231@gmail.com (H.S.); 4 Department of Global Health Policy, Graduate School of Medicine, The University of Tokyo, 7-3-1 Hongo, Bunkyo-ku, Tokyo 113-8654, Japan; hashizume@m.u-tokyo.ac.jp; 5 Department of Sustainable Health Science, Center for Preventive Medical Sciences, Chiba University, 1-33 Yayoi-cho, Inage-ku, Chiba 263-8522, Japan; siero5335@gmail.com; 6 Graduate School of Public Health, St. Luke’s International University, 10-1 Akashi-cho, Chuo-ku, Tokyo 104-0044, Japan; blue.sky.sea.dy@gmail.com (D.Y.); sgilmour@slcn.ac.jp (S.G.); 7 Institute for Business and Finance, Waseda University, 1-6-1 Nishi-Waseda, Shinjuku-ku, Tokyo 169-8050, Japan; tanoue.yuta@aoni.waseda.jp; 8 Department of Systems Pharmacology, Graduate School of Medicine, The University of Tokyo, 7-3-1 Hongo, Bunkyo-ku, Tokyo 113-8654, Japan; shoishi0322@gmail.com; 9 Laboratory for Synthetic Biology, RIKEN Center for Biosystems Dynamics Research, 1-3 Yamadaoka, Suita, Osaka 565-0871, Japan; 10 Department of Management Science, Graduate School of Engineering, Tokyo University of Science, 1-3 Kagurazaka, Shinjuku-ku, Tokyo 162-8601, Japan; 11 Center for Environmental Biology and Ecosystem Studies, National Institute for Environmental Studies (NIES), 16-2 Onogawa, Tsukuba, Ibaraki 305-8506, Japan; uryu.shinya@nies.go.jp

**Keywords:** COVID-19, healthcare access, Japan

## Abstract

We evaluated the impact of the new coronavirus disease (COVID-19) on healthcare access in Japan in terms of the number of outpatients and hospitalized patients as well as the length of hospital stays, during the first wave of the pandemic, up to June 2020. This observational study evaluated the monthly average number of outpatients per day at hospitals, the average number of hospitalized patients per day, and the average length of hospital stays per patient, from December 2010 to June 2020, using the hospital reports data, which are open aggregated data on the utilization of hospitals from the Ministry of Health, Labour and Welfare. These numbers were compared with those from the same period of previous years, using a quasi-Poisson regression model. We found a nationwide decrease in the number of outpatients in general hospitals and hospitalized patients, particularly in long-term care beds in Japan, as well as the excess length of hospital stays among psychiatric care patients during the first wave of the COVID-19. This limited access to healthcare demonstrated the importance of the long-term health monitoring of vulnerable populations and the need for urgent management support to healthcare facilities in preparation for possible prolonged pandemics in the future.

## 1. Introduction

The new coronavirus disease (COVID-19) placed an additional burden on health systems, leading to strain on all resources, including intensive care unit (ICU) beds, human resources, and access to adequate personal protective equipment (PPE) [1]. Additionally, the need to focus and consolidate limited medical resources on critically ill COVID-19 patients and those with possible infection, may have forced patients with other conditions to postpone less urgent surgery, change treatment, restrict emergency care, discontinue outpatient visits, and reallocate personnel [2]. As such, interrupted access to quality healthcare services due to this unprecedented event has been acknowledged in the worst-affected countries [3,4,5]. As an extreme consequence, increased mortality attributable to the reduced or delayed utilization of routine and emergency healthcare services has also been observed in some countries [6,7,8]. For example, in the United States, there is an excess of deaths compared to previous years from non-respiratory diseases, such as cardiovascular disease, diabetes, and Alzheimer’s disease, due to delayed emergency and routine care [9,10,11].

Meanwhile, Japan has avoided an explosive surge of COVID-19 infections to date, and has yet to experience as serious a mortality burden as the United States and Europe [12]. However, there is some evidence to suggest that the impact of COVID-19 on the healthcare system and the associated increase in disease burden may appear after the immediate crisis has passed [13]. In addition, there is a rising concern in Japan that patients refrain from seeking healthcare for fear of nosocomial infection at healthcare facilities [14]. Still, little is known about the change in population access to healthcare services during the COVID-19 pandemic in Japan.

Japan has a universal health coverage (UHC) system characterized by 100% coverage of the population through an affordable insurance system, limited cop-pays, and freedom of healthcare provider choice. Most healthcare services are reimbursed on an itemized fee-for-service basis in Japan, with uniform national prices determined by the government. Thus, the change in the volume of services and patients directly relates to the financial pressures on healthcare providers.

In Japan, COVID-19 was first detected in January 2020, and the first and second waves peaked in April and August, 2020. We evaluated the impact of COVID-19 on healthcare access in Japan by measuring the number of outpatients, the number of hospitalized patients, and the length of hospital stay using published government statistical data over time, up to June 2020 (during the first wave of the pandemic). This study not only provides insights into the capacity of health systems to respond to COVID-19 during times of crisis but also serves as a benchmark for learning more important policy lessons about the secondary health effects of the pandemic.

## 2. Materials and Methods

### 2.1. Data

We obtained open, monthly aggregated data from Hospital Reports [15], which have been administered by the Ministry of Health, Labour and Welfare (MHLW) since 1949, with the aim to clarify the utilization of hospitals and clinics by patients. Under the Medical Care Act, hospitals and clinics across the country equipped with patient beds are obliged to report utilization data every month to the MHLW through prefectural or municipal governments, including data on the number of outpatients, new admissions and discharges, and total days of stay.

We used the following monthly data for all prefectures (national level) and by prefecture: (1) the average number of outpatients per day at hospitals, (2) the average number of hospitalized patients per day, and (3) the average length of hospital stay per patient. Data (1) are also aggregated by hospital type (general or psychiatric hospitals), while data (2) are aggregated by type of bed: (a) infectious disease care beds; (b) psychiatric care beds; (c) tuberculosis care beds; (d) long-term care beds (for patients who primarily need long-term care other than (a), (b), and (c)); and (e) other beds (i.e., general beds). Long-term care beds are sub-grouped into those covered by long-term care insurance (LTCI) (hereinafter referred to as “LTCI care beds”) (f) and those covered by medical insurance, and only the former data are available. For data (3), the aggregation is almost the same as in data (2), but because there are no available data for bed type (a) in data (3) before 2019 (before the COVID-19 pandemic), we were not able to consider this bed type for the analysis of data (3). Note that data aggregation was performed by the MHLW and the background data for the aggregation (e.g., hospital-level and patient-level data) as well as standard deviations, standard errors, or some other measures of variability were not available.

### 2.2. Analyses

We considered these monthly data from December 2010 to June 2020. A quasi-Poisson regression model was used to estimate the expected monthly values, with adjustment for their long-term trend and seasonality, using the Farrington algorithm. Detailed methods are provided in Supplementary Material. We set the point estimate and upper bound of the two-sided 95% prediction interval as the threshold for excess values, and the point estimate and lower bound for exiguous values. A range for excess/exiguous values was then obtained from the differences between the observed values and each of these thresholds. The percent excess and deficit were defined as the excess and exiguous values divided by the thresholds, respectively. Note that compared with the expected values, the use of thresholds using the two-sided upper and lower limits provides a more conservative method to determine excess and exiguous values. Analysis was also performed separately by hospital types for (1) and bed types for (2) and (3), and by all prefectures (national level) and 47 prefectures. However, data for bed type (a) in data (2) were not analyzed at the prefecture level, but only at the national level, because many prefectures had zero monthly data (no patients for bed type (a)), making it difficult to build a prefecture-level model.

Finally, using the Pearson’s correlation coefficient (a common measure of the linear association between two quantitative variables [16,17]), we estimated the relationship between the monthly percent excess and deficit by prefecture and the cumulative number of patients who have tested positive for COVID-19 per population and the number of COVID-19 patients who require hospitalization per population, in order to examine if the excess and exiguous deaths were associated with the COVID-19 infection situation in each prefecture, as observed in other countries [13,18,19]. These COVID-19 data are open data reported from the MHLW [20], and the values on the 15th were used as the monthly data. We used the upper end of the range for excess/exiguous values (i.e., the difference between the point estimate and the observed values). Scatter plots were drawn for associations with a correlation coefficient higher than 0.7.

## 3. Results

**Figure 1:**
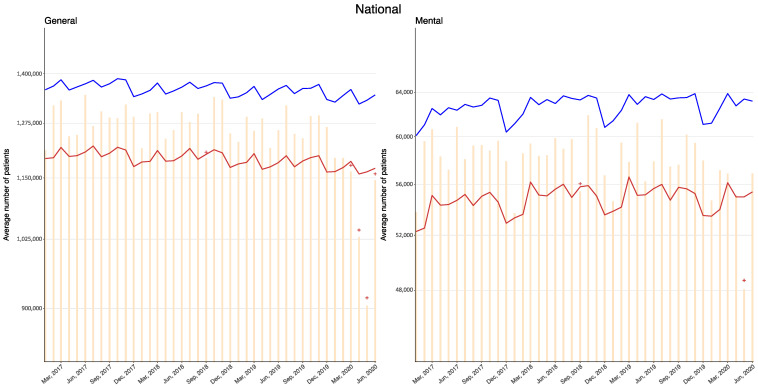
Observed and 95% upper and lower bounds of the expected monthly average number of outpatients per day at general and psychiatric hospitals in Japan up to June 2020. Orange: Observed; blue: Upper bound; red: Lower bound; cross symbols indicate months with the observed value exceeding or falling below the 95% upper or lower bounds.

In Japan, there are more than 1 million outpatient visits in general hospitals every day and more than 50,000 psychiatric visits. Since January 2020, there have been no months in either general or psychiatric hospitals in which the observed number of outpatient visits was above the 95% upper bound of the expected values. Meanwhile, months with values below the 95% lower bound were observed in general hospitals after March 2020 (exiguous values 22,448–106,788, percent deficit 1.77–8.40% for March; 129,350–209,486, 10.44–16.91% for April; 259,029–341,330, 20.79–27.39% for May; 25,704–110,787, 2.05–8.82% for June) and in psychiatric hospitals in May 2020 (6898–11,014, 11.67–18.64%). Monthly observed values and the 95% upper and lower bounds of the expected values in Japan since 2017 are presented in Figure 1, and their exact values in 2020 are provided in Table 1.

**Figure 2:**
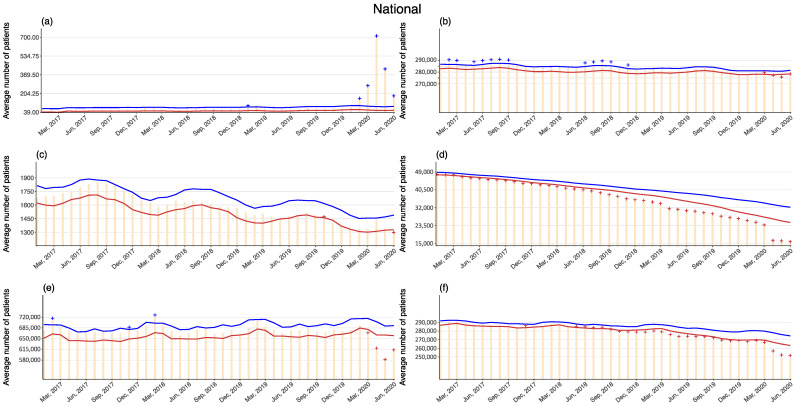
Observed and 95% upper and lower bounds of the expected monthly average number of hospitalized patients per day, in Japan up to June 2020 ((**a**): Infectious disease care beds; (**b**): Psychiatric care beds; (**c**): Tuberculosis care beds; (**d**): Long-term care beds; (**e**): General beds; and (**f**): Long-term care beds covered by long-term care insurance). Orange: Observed; blue: Upper bound; red: Lower bound; cross symbols indicate months with the observed exceeding or falling the 95% upper or lower bounds.

Observed values of hospitalized patients per day exceeded the 95% upper bound since February 2020 for infectious disease care beds with a peak in April (excess values 617–635, percent excess 878.74–904.07). On the other hand, months with observed values below the 95% lower bound were observed in all other hospital beds, with the largest percent decline in LTCI care beds, especially in April (exiguous values 10,673–13,989, percent deficit 35.62–46.68) as well as with the largest absolute amount of decline in general beds, especially in May (86,236–100,975, 12.75–14.93). Monthly observed values and the 95% upper and lower bounds of the expected values in Japan since 2017 are presented in Figure 2, and their exact values in 2020 are provided in Table 1.

**Figure 3:**
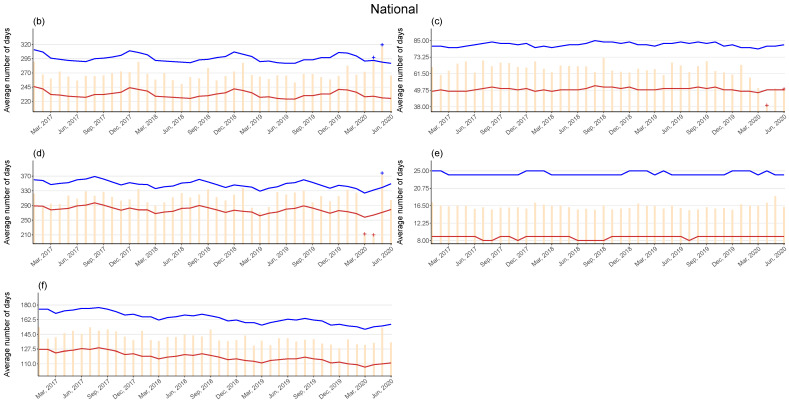
Observed and 95% upper and lower bounds of the expected monthly average length of hospital stays per patient, in Japan up to June 2020 (**b**: Psychiatric care beds; **c**: Tuberculosis care beds; **d**: Long-term care beds; **e**: General beds; and **f**: Long-term care beds covered by long-term care insurance). Orange: Observed; blue: Upper bound; red: Lower bound; cross symbols indicate months with the observed exceeding or falling below the 95% upper or lower bounds.

Observed values of the average length of hospital stays exceeding the 95% upper bound were observed in April and May in psychiatric care beds (excess values 3–35, percent excess 0.85–13.45 for April; 28–60, 10.71–23.24 for May), and in May in LTCI care beds (36–72, 11.76–23.39). Observed values below the 95% lower bound were also observed for tuberculosis care beds in April and June (exiguous values 12–27, percent deficit 18.13–40.64 for April; 1–16, 0.15–23.23 for June) and for LTCI care beds in March and April (50–82, 17.03–28.08 for March; 58–91, 19.49–30.61 for April). Monthly observed values and the 95% upper and lower bounds of the expected values in Japan since 2017 are presented in Figure 3, and their exact values in 2020 are provided in Table 1.

Prefecture-specific estimates are presented in Table S1 and Figures S1–S3 for the average number of outpatients per day at hospitals, the average number of hospitalized patients per day, and the average length of hospital stays per patient, respectively. Their trends were similar to those at the national level. The cumulative number of patients who tested positive for COVID-19 per population exceeded the Pearson’s correlation coefficient of 0.7 with the percent deficit of the average number of outpatients per day at general hospitals in April (r = 0.8121) and May (r = 0.7517) and the percent excess of the average length of hospital stays per patient in general beds in April (r = 0.8711) and May (r = 0.8265). The number of COVID-19 patients who require hospitalization per population also exceeded the coefficient of 0.7 with the percent deficit of the average number of outpatients per day at general hospitals in April (r = 0.8325) and the percent excess of the average length of hospital stays per patient in general beds in April (r = 0.8796) and May (r = 0.7517). Scatter plots for these associations are presented in Figures S4–S10, respectively.

## 4. Discussion

To our knowledge, this is the first study to assess the impact of the COVID-19 pandemic on healthcare access after rigorous consideration of past trends. Japan is currently experiencing a third wave of the COVID-19 as of December 2020, with the first wave occurring in April and the second in August. It should be noted that this study is limited to data up to June, and, therefore, the results are related only to the early phase of the pandemic. In addition, the statistical measures we used are “average values” by prefecture, published by the MHLW, but the distribution may be distorted, so it may be appropriate to evaluate by median values. However, unfortunately, since the published data from MHLW are only average values, it was not possible to evaluate them by median values.

We found that in Japan, the number of outpatients in general hospitals has decreased across the country since around April—when a state of emergency was declared by the government in accordance with an increase in the number of infection cases—and reached its nadir in May. Although caution is necessary as no possible confounder adjustment was conducted, the prefecture-level correlations with the cumulative number of COVID-19 patients as well as those requiring hospitalization were significant. Among the reasons for this may be that patients are reluctant to visit hospitals for fear of nosocomial infections or that hospitals that have difficulty in dealing with potential infected patients due to, for example, lack of protective equipment, such as PPE or consumable medical devices, directed patients to hospitals with appropriate infection control, such as healthcare facilities designated for specific infectious diseases, prescribed by the Act on Prevention of Infectious Diseases and Medical Care for Patients Suffering Infectious Disease.

The excess number of hospitalized patients for infectious disease care beds peaked in April (617–635), corresponding to the reported number of hospitalized patients due to COVID-19; it peaked at 688 on 11 April 2020, during the study period. Meanwhile, the number of hospitalized patients, particularly in LTCI care beds, declined gradually earlier this year, peaking in April. Most people who use these beds are older people. The reason for this result is probably due to efforts to minimize the hospitalization of such older people who are at high risk of developing severe cases of COVID-19 or to prioritize those unable to receive care from their relatives at home. At that time, it was also reported that the number of users of LTCI care services in long-term care facilities or at home (i.e., home visiting nursing) decreased after the pandemic [21]. This decrease in the number of hospitalized LTCI care patients might have resulted in the additional care burden of home care, particularly for women [22]. Previous research on excess suicide mortality has found an increase in suicide mortality among women during this time, which may reflect increased home care burden and the pressures of pandemic isolation, and consideration in future needs to be given to the psychological burden that is placed on women as primary carers when caring facilities suddenly restrict services [23]. An important caution is necessary that, as one of the limitations of this study, the use of hospital beds for long-term care was abolished at the end of 2017, and transfers of patients to nursing facilities have been underway. Since the Farrington algorithm assumes a trend with no change point, such policy changes may result in a bias, i.e., the estimated excess/exiguous values may relate to the mixed effects of this policy in addition to the COVID-19 pandemic.

In addition, in some prefectures, both excess and exiguous lengths of hospital stays per patient were found in the LTCI care beds during the study period. It is likely for many older people who use this bed to be transferred to long-term care facilities when they are discharged from hospital. The results of this study, therefore, indicate that the host long-term care facilities are concerned about the transportation of potential COVID-19 patients as well as their staffing or equipment issues, and that it takes time to prepare adequate protection measures against COVID-19, making it irregular and difficult to coordinate discharge. Similarly, the excess length of hospital stays per patient, observed in many prefectures, especially in May for psychiatric care beds, may mean that the patient’s transition from hospital to home care services has been postponed due to difficulties in coordinating home-visit care, which has prevented the patient’s discharge.

As of August, a previous study found much lower overall excess mortality burden from COVID-19 in Japan than in the United States and Europe [12]. However, among the most worrisome aspects of the decline in the number of hospitalized patients and outpatients is the subsequent deterioration of patients’ chronic illnesses. Among the limitations of this study was that it was not possible to analyze by department, but in July, it was reported that, for example, the number of pediatric outpatients had decreased markedly since March [24]. Our study findings provide evidence suggesting the importance of monitoring the future health status of vulnerable populations, in particular the elderly and children, as well as patients with chronic diseases, such as diabetes and cardiovascular diseases, who are required to have regular monitoring [9]. Some of the literature suggested the potential usefulness of telemedicine services, including remote monitoring and nursing teleconsultation, to ensure the continuity of care and outpatient management during the COVID-19 pandemic [25,26,27].

The effect of COVID-19 on healthcare access also may have caused serious damage to the management of healthcare facilities. In addition to the decline in the number of inpatients and outpatients, many healthcare facilities in Japan are facing serious financial difficulties due to capital investment, such as the expansion of the treatment space for outpatients with fever or any COVID-19-like symptoms [28] and the purchase of protective equipment, as well as an increase in personnel-related costs due to staff recruitment and relocation. According to a survey conducted by the Japan Hospital Association, the All Japan Hospital Association, and the Association of Japanese Healthcare Corporations, of the 1460 hospitals that responded to the survey, those that reported a monthly deficit in April-June increased 20% from last year to more than 60–70% [29]. This tendency was especially pronounced in hospitals admitting patients with COVID-19. Our findings suggest that this may be directly related to a reduction in service provision, and suggest the need for the government to provide some form of financial support to healthcare facilities in the aftermath of the COVID-19 pandemic to ensure their continued operation. Contingency plans should also be prepared for both the next wave of the COVID-19 pandemic and to better manage healthcare resources in the next pandemic.

## 5. Conclusions

This study found a nationwide decrease in the number of outpatients in general hospitals and hospitalized patients, particularly in LTCI care beds in Japan, as well as extended hospital stays among psychiatric care patients during the first wave of COVID-19. This limited access to healthcare demonstrates the importance of the long-term health monitoring of vulnerable populations and the need for urgent management support to healthcare facilities in preparation for possible prolonged pandemics in the future.

## Figures and Tables

**Table 1:** Monthly number of observed, excess (percent), and exiguous (percent) values in Japan up to June 2020, by subgroups.

	Observed	Excess	Percent Excess	Exiguous	Percent Deficit		
(1) Average number of outpatients per day at hospitals							
General hospitals							
January	1,194,286	0–0	0.00–0.00	0–49,539	0.00–3.98		
February	1,194,077	0–0	0.00–0.00	0–62,457	0.00–4.97		
March	1,164,224	0–0	0.00–0.00	22,448–106,788	1.77–8.40		**
April	1,029,399	0–0	0.00–0.00	129,350–209,486	10.44–16.91		**
May	904,647	0–0	0.00–0.00	259,029–341,330	20.79–27.39		**
June	1,145,535	0–0	0.00–0.00	25,704–110,787	2.05–8.82		**
Psychiatric hospitals							
January	54,687	0–0	0.00–0.00	0–2566	0.00–4.48		
February	57,140	0–0	0.00–0.00	0–1038	0.00–1.78		
March	568,70	0–0	0.00–0.00	0–3063	0.00–5.11		
April	55,129	0–0	0.00–0.00	0–3669	0.00–6.24		
May	48,083	0–0	0.00–0.00	6898–11,014	11.67–18.64		**
June	56,883	0–0	0.00–0.00	0–2329	0.00–3.93		
(2) Average number of hospitalized patients per day							
(a) Infectious disease care beds							
January	79	0–1	0.00–1.05	0–0	0.00–0.00		
February	154	57–76	72.47–95.79	0–0	0.00–0.00	*	
March	267	175–193	235.58–259.42	0–0	0.00–0.00	*	
April	705	617–635	878.74–904.07	0–0	0.00–0.00	*	
May	414	327–345	471.57–497.04	0–0	0.00–0.00	*	
June	176	86–104	119.36–144.28	0–0	0.00–0.00	*	
(b) Psychiatric care beds							
January	278,200	0–0	0.00–0.00	0–1217	0.00–0.44		
February	278,345	0–0	0.00–0.00	0–1266	0.00–0.45		
March	276,793	0–0	0.00–0.00	1225–2714	0.44–0.97		**
April	274,334	0–0	0.00–0.00	3362–4942	1.20–1.77		**
May	272,879	0–0	0.00–0.00	5182–6477	1.85–2.32		**
June	275,436	0–0	0.00–0.00	2871–4413	1.03–1.58		**
(c) Tuberculosis care beds							
January	1336	0–0	0.00–0.00	0–74	0.00–5.20		
February	1343	0–0	0.00–0.00	0–37	0.00–2.63		
March	1333	0–0	0.00–0.00	0–45	0.00–3.21		
April	1439	0–57	0.00–4.06	0–0	0.00–0.00		
May	1338	0–0	0.00–0.00	0–58	0.00–4.10		
June	1279	0–0	0.00–0.00	52–127	3.70–8.98		**
(d) Nursing care beds							
January	264,611	0–0	0.00–0.00	5661–10,702	2.06–3.89		**
February	266,089	0–0	0.00–0.00	3792–9073	1.38–3.30		**
March	263,790	0–0	0.00–0.00	5558–10,790	2.02–3.93		**
April	254,053	0–0	0.00–0.00	12,761–18,318	4.69–6.73		**
May	249,345	0–0	0.00–0.00	15,497–20,979	5.73–7.76		**
June	248,918	0–0	0.00–0.00	14,258–19,865	5.30–7.39		**
(e) General beds							
January	678,772	0–0	0.00–0.00	0–12,995	0.00–1.88		
February	693,259	0–0	0.00–0.00	0–6517	0.00–0.93		
March	661,980	0–0	0.00–0.00	18,248–35,869	2.61–5.14		**
April	611,787	0–0	0.00–0.00	50,126–71,440	7.34–10.46		**
May	575,307	0–0	0.00–0.00	86,236–100,975	12.75–14.93		**
June	606,442	0–0	0.00–0.00	53,019–69,453	7.84–10.28		**
(f) Long-term care beds							
January	25,464	0–0	0.00–0.00	5162–7489	15.67–22.72		**
February	24,606	0–0	0.00–0.00	4223–7188	13.28–22.61		**
March	23,329	0–0	0.00–0.00	4595–7672	14.82–24.75		**
April	15,976	0–0	0.00–0.00	10,673–13,989	35.62–46.68		**
May	15,762	0–0	0.00–0.00	9967–13,379	34.20–45.91		**
June	15,398	0–0	0.00–0.00	9583–13,088	33.64–45.94		**
(3) Average length of hospital stays per patient							
(b) Psychiatric care beds							
January	283	0–11	0.00–4.03	0–0	0.00–0.00		
February	267	0–1	0.00–0.01	0–0	0.00–0.00		
March	272	0–13	0.00–4.95	0–0	0.00–0.00		
April	294	3–35	0.85–13.45	0–0	0.00–0.00	*	
May	317	28–60	10.71–23.24	0–0	0.00–0.00	*	
June	265	0–11	0.00–4.03	0–0	0.00–0.00		
(c) Tuberculosis care beds							
January	68	0–5	0.00–6.36	0–0	0.00–0.00		
February	59	0–0	0.00–0.00	0–5	0.00–7.61		
March	51	0–0	0.00–0.00	0–12	0.00–17.86		
April	38	0–0	0.00–0.00	12–27	18.13–40.64		**
May	51	0–0	0.00–0.00	0–14	0.00–21.16		
June	50	0–0	0.00–0.00	1–16	0.15–23.23		**
(d) Long-term care beds							
January	139	0–8	0.00–5.40	0–0	0.00–0.00		
February	133	0–3	0.00–1.69	0–0	0.00–0.00		
March	132	0–5	0.00–3.51	0–0	0.00–0.00		
April	135	0–4	0.00–2.89	0–0	0.00–0.00		
May	153	0–21	0.00–15.82	0–0	0.00–0.00		
June	135	0–2	0.00–1.47	0–0	0.00–0.00		
(e) General beds							
January	17	0–1	0.00–3.01	0–0	0.00–0.00		
February	17	0–1	0.00–0.64	0–0	0.00–0.00		
March	16	0–1	0.00–2.00	0–0	0.00–0.00		
April	17	0–2	0.00–6.52	0–0	0.00–0.00		
May	19	0–3	0.00–18.15	0–0	0.00–0.00		
June	16	0–1	0.00–2.76	0–0	0.00–0.00		
(f) LTCI care beds							
January	333	0–27	0.00–8.59	0–0	0.00–0.00		
February	325	0–24	0.00–7.78	0–0	0.00–0.00		
March	209	0–0	0.00–0.00	50–82	17.03–28.08		**
April	206	0–0	0.00–0.00	58–91	19.49–30.61		**
May	375	36–72	11.76–23.39	0–0	0.00–0.00	*	
June	305	0–0	0.00–0.00	0–9	0.00–2.67		

LTCI care beds: Long-term care beds covered by long-term care insurance. * indicates a month where the observed value exceeded the upper bound of the two-sided 95% prediction interval; ** indicates a month where the observed value fell below the lower bound of the two-sided 95% prediction interval.

## Data Availability

The data are publicly available at https://www.mhlw.go.jp/toukei/list/79-1a.html (accessed on 9 March 2021).

## Supplementary Materials

The following are available online at https://www.mdpi.com/1660-4601/18/6/3271/s1, Document S1: Detailed methods of the analysis, Table S1:Monthly number of observed, excess (percent), and exiguous (percent) values in Japan, up to June 2020 by subgroups by 47 prefectures, Figure S1: Observed and 95% upper and lower bounds of the expected monthly average number of outpatients per day at hospitals, in 47 prefectures up to June 2020 (general and psychiatric hospitals), Figure S2: Observed and 95% upper and lower bounds of the expected monthly average number of hospitalized patients per day, in 47 prefectures up to June 2020 (a: Infectious disease care beds; b: Psychiatric care beds; c: Tuberculosis care beds; d: Long-term care beds; e: General beds; and f: Long-term care beds covered by long-term care insurance), Figure S3: Observed and 95% upper and lower bounds of the expected monthly average length of hospital stays per patient, in 47 prefectures up to June 2020 (b: Psychiatric care beds; c: Tuberculosis care beds; d: Long-term care beds; e: General beds; and f: Long-term care beds covered by long-term care insurance), Figure S4: Comparison of the cumulative number of patients who tested positive for COVID-19 per population and the percent deficit of the average number of outpatients per day at general hospitals in April, with a simple linear regression line, Figure S5: Comparison of the cumulative number of patients who tested positive for COVID-19 per population and the percent deficit of the average number of outpatients per day at general hospitals in May, with a simple linear regression line, Figure S6: Comparison of the cumulative number of patients who tested positive for COVID-19 per population and the percent excess of the average length of hospital stays per patient in general beds in April, with a simple linear regression line, Figure S7: Comparison of the cumulative number of patients who tested positive for COVID-19 per population and the percent excess of the average length of hospital stays per patient in general beds in May, with a simple linear regression line, Figure S8: Comparison of number of COVID-19 patients who required hospitalization per population and the percent deficit of the average number of outpatients per day at general hospitals in April, with a simple linear regression line, Figure S9: Comparison of number of COVID-19 patients who required hospitalization per population and the percent excess of the average length of hospital stays per patient in general beds in April, with a simple linear regression line, Figure S10: Comparison of number of COVID-19 patients who required hospitalization per population and the percent excess of the average length of hospital stays per patient in general beds in May, with a simple linear regression line.
