# Supplementary material for: Trends in Healthcare Access in Japan during the First Wave of the COVID-19 Pandemic, up to June 2020
Source: Int J Environ Res Public Health. 2021 Mar 22;18(6):3271. doi: 10.3390/ijerph18063271 (PMC8004161; doi:10.3390/ijerph18063271)
Supplement: Supplementary file 1 [file ijerph-18-03271-s001.zip › Appendix Text.docx]

**Estimating expected values**

To calculate the expected values and their prediction intervals, we applied the Farrington algorithm, which uses a quasi-Poisson regression model and is commonly used to study the annual and seasonal trends in the burden of disease attributable to seasonal pandemics.[1] The major characteristic of Farrington algorithm is to restrict the data used for the estimation: the expected values at a calendar month $t$ is estimated using only the data during $t-w$ to $t+w$ months of years $h-b$ and $h-1$, where $w$ and $b$ are pre-fixed values and $h$ is the year of $t$. In this study, we considered $b=5$ and $w=1$ [2]. The limited data is referred to as a reference period data. Finally, to consider seasonality in the model, data that is not included in the reference period is equally divided into four periods and included in the regression model as dummy variables. Then, the regression model is written as:

$\log\left( E\left( Y_{t} \right) \right)=\alpha+\beta t+\boldsymbol{f}^{T}\left( t \right)\boldsymbol{\gamma}_{\boldsymbol{f}},$ (1)

where $Y_{t}$ is the values at a certain month $t$, $\alpha$ and $\beta$ are regression parameters, $\boldsymbol{\gamma}_{\boldsymbol{f}}$ is a regression parameter vector representing the seasonality, and$\boldsymbol{f}\mathbf{(}\boldsymbol{t}\mathbf{)}$ is a vector of dummies that equally divides the time points outside the reference period (into four periods in this study to divide into four equal parts of three months). The parameters, including the regression coefficients and overdispersion parameter $\phi$, were estimated by the quasi-likelihood approach. More details can be found in Farrington *et al*. (1996), Noufaily *et al*. (2013) and Bedubourg and Strat (2017).[2-4]

Once the regression parameters were estimated, the expected value is predicted for the week of interest $t_{0}$. The two-sided 95% prediction intervals are then estimated by assuming that the data follows the negative binomial distribution as $Y_{t_{0}}\sim NB(\hat{Y_{t_{0}}},\hat{\nu_{0}})$, where $\hat{Y_{t_{0}}}$ is the mean of the distribution and $\hat{\nu_{0}}=\hat{\frac{Y_{t_{0}}}{\phi-1}}$ is its dispersion parameter. Note that, although we used data during 2010–2020 for the estimation, the results during 2016–2020 were shown in Figures for simplicity.

**Bibliography**

1. Vestergaard LS, Nielsen J, Richter L, et al. Excess all-cause mortality during the COVID-19 pandemic in Europe - preliminary pooled estimates from the EuroMOMO network, March to April 2020. *Euro Surveill* 2020; **25**(26).

2. Bedubourg G, Le Strat Y. Evaluation and comparison of statistical methods for early temporal detection of outbreaks: A simulation-based study. *PLoS One* 2017; **12**(7): e0181227.

3. Farrington CP, Andrews NJ, Beale AD, et al. A Statistical Algorithm for the Early Detection of Outbreaks of Infectious Disease. *Journal of the Royal Statistical Society Series A (Statistics in Society)* 1996; **159**(3): 547-63.

4. Noufaily A, Enki DG, Farrington P, et al. An improved algorithm for outbreak detection in multiple surveillance systems. *Stat Med* 2013; **32**(7): 1206-22.
