# Supplementary material for: Trends in Healthcare Access in Japan during the First Wave of the COVID-19 Pandemic, up to June 2020
Source: Int J Environ Res Public Health. 2021 Mar 22;18(6):3271. doi: 10.3390/ijerph18063271 (PMC8004161; doi:10.3390/ijerph18063271)
Supplement: Supplementary file 1 [file ijerph-18-03271-s001.zip › Appendix Table 1.docx]

**Supplementary table 1: Monthly number of observed, excess (percent), and exiguous (percent) values in 47 prefectures, up to June 2020 by subgroups.**

| Prefecture | Month | Observed | Excess | Percent excess | Exiguous | Percent deficit |  |  |
| --- | --- | --- | --- | --- | --- | --- | --- | --- |
| Hokkaido | (1) Average number of outpatients per day at hospitals |  |  |  |  |  |  |  |
|  | General hospitals |  |  |  |  |  |  |  |
|  | January | 64604 | 0–0 | 0.00–0.00 | 0–3349 | 0.00–4.93 |  |  |
|  | February | 64671 | 0–0 | 0.00–0.00 | 0–3536 | 0.00–5.18 |  |  |
|  | March | 60943 | 0–0 | 0.00–0.00 | 3467–8928 | 4.96–12.78 |  | ** |
|  | April | 61072 | 0–0 | 0.00–0.00 | 2875–7671 | 4.18–11.16 |  | ** |
|  | May | 51432 | 0–0 | 0.00–0.00 | 12403–17673 | 17.95–25.57 |  | ** |
|  | June | 64595 | 0–0 | 0.00–0.00 | 0–4810 | 0.00–6.93 |  |  |
|  | Psychiatric hospitals |  |  |  |  |  |  |  |
|  | January | 3643 | 0–0 | 0.00–0.00 | 0–151 | 0.00–3.97 |  |  |
|  | February | 3670 | 0–0 | 0.00–0.00 | 0–170 | 0.00–4.41 |  |  |
|  | March | 3606 | 0–0 | 0.00–0.00 | 44–368 | 1.11–9.24 |  | ** |
|  | April | 3711 | 0–0 | 0.00–0.00 | 0–179 | 0.00–4.60 |  |  |
|  | May | 3148 | 0–0 | 0.00–0.00 | 460–788 | 11.69–20.01 |  | ** |
|  | June | 3814 | 0–0 | 0.00–0.00 | 0–151 | 0.00–3.80 |  |  |
|  | (2) Average number of hospitalized patients per day |  |  |  |  |  |  |  |
|  | (b) Psychiatric care beds |  |  |  |  |  |  |  |
|  | January | 16913 | 0–33 | 0.00–0.19 | 0–0 | 0.00–0.00 |  |  |
|  | February | 16886 | 0–0 | 0.00–0.00 | 0–11 | 0.00–0.06 |  |  |
|  | March | 16789 | 0–0 | 0.00–0.00 | 0–110 | 0.00–0.65 |  |  |
|  | April | 16703 | 0–0 | 0.00–0.00 | 0–175 | 0.00–1.04 |  |  |
|  | May | 16567 | 0–0 | 0.00–0.00 | 50–304 | 0.30–1.80 |  | ** |
|  | June | 16681 | 0–0 | 0.00–0.00 | 0–184 | 0.00–1.09 |  |  |
|  | (c) Tuberculosis care beds |  |  |  |  |  |  |  |
|  | January | 24 | 0–0 | 0.00–0.00 | 0–9 | 0.00–26.52 |  |  |
|  | February | 29 | 0–0 | 0.00–0.00 | 0–4 | 0.00–9.71 |  |  |
|  | March | 31 | 0–0 | 0.00–0.00 | 0–2 | 0.00–5.60 |  |  |
|  | April | 50 | 3–16 | 8.74–45.61 | 0–0 | 0.00–0.00 | * |  |
|  | May | 51 | 2–15 | 5.52–40.81 | 0–0 | 0.00–0.00 | * |  |
|  | June | 42 | 0–6 | 0.00–15.61 | 0–0 | 0.00–0.00 |  |  |
|  | (d) Long-term care beds |  |  |  |  |  |  |  |
|  | January | 17762 | 0–78 | 0.00–0.44 | 0–0 | 0.00–0.00 |  |  |
|  | February | 17933 | 0–249 | 0.00–1.40 | 0–0 | 0.00–0.00 |  |  |
|  | March | 17822 | 0–139 | 0.00–0.79 | 0–0 | 0.00–0.00 |  |  |
|  | April | 17381 | 0–0 | 0.00–0.00 | 0–219 | 0.00–1.24 |  |  |
|  | May | 17121 | 0–0 | 0.00–0.00 | 157–416 | 0.90–2.37 |  | ** |
|  | June | 17096 | 0–0 | 0.00–0.00 | 126–384 | 0.72–2.20 |  | ** |
|  | (e) General beds |  |  |  |  |  |  |  |
|  | January | 39149 | 0–0 | 0.00–0.00 | 0–793 | 0.00–1.98 |  |  |
|  | February | 40159 | 0–0 | 0.00–0.00 | 0–229 | 0.00–0.56 |  |  |
|  | March | 37413 | 0–0 | 0.00–0.00 | 1881–2933 | 4.66–7.27 |  | ** |
|  | April | 35864 | 0–0 | 0.00–0.00 | 2287–3649 | 5.79–9.23 |  | ** |
|  | May | 33361 | 0–0 | 0.00–0.00 | 4870–5912 | 12.40–15.05 |  | ** |
|  | June | 35005 | 0–0 | 0.00–0.00 | 3269–4283 | 8.32–10.90 |  | ** |
|  | (f) LTCI care beds |  |  |  |  |  |  |  |
|  | January | 1574 | 0–37 | 0.00–2.34 | 0–0 | 0.00–0.00 |  |  |
|  | February | 1576 | 0–66 | 0.00–4.34 | 0–0 | 0.00–0.00 |  |  |
|  | March | 1519 | 0–39 | 0.00–2.60 | 0–0 | 0.00–0.00 |  |  |
|  | April | 1195 | 0–0 | 0.00–0.00 | 97–254 | 6.70–17.48 |  | ** |
|  | May | 1190 | 0–0 | 0.00–0.00 | 81–231 | 5.70–16.22 |  | ** |
|  | June | 1103 | 0–0 | 0.00–0.00 | 150–297 | 10.72–21.20 |  | ** |
|  | (3) Average length of hospital stays per patient |  |  |  |  |  |  |  |
|  | (b) Psychiatric care beds |  |  |  |  |  |  |  |
|  | January | 274 | 0–4 | 0.00–1.28 | 0–0 | 0.00–0.00 |  |  |
|  | February | 274 | 0–8 | 0.00–2.79 | 0–0 | 0.00–0.00 |  |  |
|  | March | 290 | 1–33 | 0.04–12.61 | 0–0 | 0.00–0.00 | * |  |
|  | April | 292 | 4–36 | 1.25–13.69 | 0–0 | 0.00–0.00 | * |  |
|  | May | 316 | 31–63 | 11.92–24.42 | 0–0 | 0.00–0.00 | * |  |
|  | June | 258 | 0–6 | 0.00–2.08 | 0–0 | 0.00–0.00 |  |  |
|  | (c) Tuberculosis care beds |  |  |  |  |  |  |  |
|  | January | 48 | 0–0 | 0.00–0.00 | 0–4 | 0.00–7.36 |  |  |
|  | February | 47 | 0–0 | 0.00–0.00 | 0–8 | 0.00–13.37 |  |  |
|  | March | 23 | 0–0 | 0.00–0.00 | 12–30 | 22.13–55.94 |  | ** |
|  | April | 24 | 0–0 | 0.00–0.00 | 14–33 | 24.19–57.89 |  | ** |
|  | May | 29 | 0–0 | 0.00–0.00 | 6–26 | 10.52–47.05 |  | ** |
|  | June | 32 | 0–0 | 0.00–0.00 | 9–29 | 13.82–47.22 |  | ** |
|  | (d) Long-term care beds |  |  |  |  |  |  |  |
|  | January | 193 | 0–11 | 0.00–5.90 | 0–0 | 0.00–0.00 |  |  |
|  | February | 192 | 0–11 | 0.00–6.01 | 0–0 | 0.00–0.00 |  |  |
|  | March | 202 | 0–27 | 0.00–15.23 | 0–0 | 0.00–0.00 |  |  |
|  | April | 194 | 0–16 | 0.00–8.82 | 0–0 | 0.00–0.00 |  |  |
|  | May | 229 | 25–52 | 13.59–28.71 | 0–0 | 0.00–0.00 | * |  |
|  | June | 189 | 0–11 | 0.00–5.93 | 0–0 | 0.00–0.00 |  |  |
|  | (e) General beds |  |  |  |  |  |  |  |
|  | January | 18 | 0–1 | 0.00–2.55 | 0–0 | 0.00–0.00 |  |  |
|  | February | 18 | 0–1 | 0.00–1.57 | 0–0 | 0.00–0.00 |  |  |
|  | March | 18 | 0–2 | 0.00–6.55 | 0–0 | 0.00–0.00 |  |  |
|  | April | 18 | 0–1 | 0.00–4.88 | 0–0 | 0.00–0.00 |  |  |
|  | May | 20 | 0–4 | 0.00–20.49 | 0–0 | 0.00–0.00 |  |  |
|  | June | 17 | 0–1 | 0.00–2.90 | 0–0 | 0.00–0.00 |  |  |
|  | (f) LTCI care beds |  |  |  |  |  |  |  |
|  | January | 485 | 0–80 | 0.00–19.58 | 0–0 | 0.00–0.00 |  |  |
|  | February | 511 | 10–120 | 2.46–30.71 | 0–0 | 0.00–0.00 | * |  |
|  | March | 342 | 0–0 | 0.00–0.00 | 0–35 | 0.00–9.13 |  |  |
|  | April | 311 | 0–0 | 0.00–0.00 | 0–74 | 0.00–19.17 |  |  |
|  | May | 512 | 0–102 | 0.00–24.71 | 0–0 | 0.00–0.00 |  |  |
|  | June | 250 | 0–0 | 0.00–0.00 | 78–180 | 18.02–41.79 |  | ** |
| Aomori | (1) Average number of outpatients per day at hospitals |  |  |  |  |  |  |  |
|  | General hospitals |  |  |  |  |  |  |  |
|  | January | 11997 | 0–0 | 0.00–0.00 | 0–579 | 0.00–4.60 |  |  |
|  | February | 11962 | 0–0 | 0.00–0.00 | 0–776 | 0.00–6.09 |  |  |
|  | March | 12437 | 0–0 | 0.00–0.00 | 0–533 | 0.00–4.11 |  |  |
|  | April | 11665 | 0–0 | 0.00–0.00 | 239–1067 | 1.88–8.38 |  | ** |
|  | May | 9838 | 0–0 | 0.00–0.00 | 2099–2994 | 16.36–23.33 |  | ** |
|  | June | 12138 | 0–0 | 0.00–0.00 | 0–764 | 0.00–5.92 |  |  |
|  | Psychiatric hospitals |  |  |  |  |  |  |  |
|  | January | 950 | 0–68 | 0.00–7.65 | 0–0 | 0.00–0.00 |  |  |
|  | February | 971 | 0–61 | 0.00–6.69 | 0–0 | 0.00–0.00 |  |  |
|  | March | 1016 | 0–73 | 0.00–7.67 | 0–0 | 0.00–0.00 |  |  |
|  | April | 998 | 0–60 | 0.00–6.34 | 0–0 | 0.00–0.00 |  |  |
|  | May | 847 | 0–0 | 0.00–0.00 | 33–130 | 3.38–13.28 |  | ** |
|  | June | 1023 | 0–38 | 0.00–3.81 | 0–0 | 0.00–0.00 |  |  |
|  | (2) Average number of hospitalized patients per day |  |  |  |  |  |  |  |
|  | (b) Psychiatric care beds |  |  |  |  |  |  |  |
|  | January | 3687 | 0–0 | 0.00–0.00 | 0–18 | 0.00–0.46 |  |  |
|  | February | 3675 | 0–0 | 0.00–0.00 | 0–27 | 0.00–0.71 |  |  |
|  | March | 3683 | 0–0 | 0.00–0.00 | 0–8 | 0.00–0.21 |  |  |
|  | April | 3626 | 0–0 | 0.00–0.00 | 0–44 | 0.00–1.19 |  |  |
|  | May | 3616 | 0–0 | 0.00–0.00 | 0–50 | 0.00–1.34 |  |  |
|  | June | 3648 | 0–0 | 0.00–0.00 | 0–21 | 0.00–0.57 |  |  |
|  | (c) Tuberculosis care beds |  |  |  |  |  |  |  |
|  | January | 12 | 0–5 | 0.00–52.90 | 0–0 | 0.00–0.00 |  |  |
|  | February | 9 | 0–1 | 0.00–10.28 | 0–0 | 0.00–0.00 |  |  |
|  | March | 12 | 0–4 | 0.00–49.83 | 0–0 | 0.00–0.00 |  |  |
|  | April | 9 | 0–2 | 0.00–16.19 | 0–0 | 0.00–0.00 |  |  |
|  | May | 13 | 0–6 | 0.00–67.37 | 0–0 | 0.00–0.00 |  |  |
|  | June | 14 | 0–6 | 0.00–73.88 | 0–0 | 0.00–0.00 |  |  |
|  | (d) Long-term care beds |  |  |  |  |  |  |  |
|  | January | 2314 | 0–0 | 0.00–0.00 | 1–96 | 0.04–3.98 |  | ** |
|  | February | 2346 | 0–0 | 0.00–0.00 | 0–78 | 0.00–3.19 |  |  |
|  | March | 2355 | 0–0 | 0.00–0.00 | 0–59 | 0.00–2.41 |  |  |
|  | April | 2309 | 0–0 | 0.00–0.00 | 0–71 | 0.00–2.98 |  |  |
|  | May | 2258 | 0–0 | 0.00–0.00 | 0–93 | 0.00–3.95 |  |  |
|  | June | 2303 | 0–0 | 0.00–0.00 | 0–36 | 0.00–1.53 |  |  |
|  | (e) General beds |  |  |  |  |  |  |  |
|  | January | 7028 | 0–0 | 0.00–0.00 | 96–347 | 1.30–4.70 |  | ** |
|  | February | 7247 | 0–0 | 0.00–0.00 | 0–146 | 0.00–1.97 |  |  |
|  | March | 7007 | 0–0 | 0.00–0.00 | 72–313 | 0.98–4.27 |  | ** |
|  | April | 6478 | 0–0 | 0.00–0.00 | 435–683 | 6.08–9.53 |  | ** |
|  | May | 6168 | 0–0 | 0.00–0.00 | 770–950 | 10.82–13.34 |  | ** |
|  | June | 6521 | 0–0 | 0.00–0.00 | 386–600 | 5.42–8.42 |  | ** |
|  | (f) LTCI care beds |  |  |  |  |  |  |  |
|  | January | 463 | 0–0 | 0.00–0.00 | 0–13 | 0.00–2.66 |  |  |
|  | February | 474 | 0–0 | 0.00–0.00 | 0–5 | 0.00–1.04 |  |  |
|  | March | 477 | 0–0 | 0.00–0.00 | 0–5 | 0.00–0.93 |  |  |
|  | April | 473 | 0–0 | 0.00–0.00 | 0–12 | 0.00–2.37 |  |  |
|  | May | 473 | 0–0 | 0.00–0.00 | 0–13 | 0.00–2.63 |  |  |
|  | June | 470 | 0–0 | 0.00–0.00 | 0–16 | 0.00–3.23 |  |  |
|  | (3) Average length of hospital stays per patient |  |  |  |  |  |  |  |
|  | (b) Psychiatric care beds |  |  |  |  |  |  |  |
|  | January | 262 | 0–21 | 0.00–8.48 | 0–0 | 0.00–0.00 |  |  |
|  | February | 241 | 0–2 | 0.00–0.83 | 0–0 | 0.00–0.00 |  |  |
|  | March | 244 | 0–11 | 0.00–4.64 | 0–0 | 0.00–0.00 |  |  |
|  | April | 242 | 0–12 | 0.00–5.14 | 0–0 | 0.00–0.00 |  |  |
|  | May | 270 | 10–41 | 4.27–17.69 | 0–0 | 0.00–0.00 | * |  |
|  | June | 231 | 0–3 | 0.00–0.88 | 0–0 | 0.00–0.00 |  |  |
|  | (c) Tuberculosis care beds |  |  |  |  |  |  |  |
|  | January | 121 | 0–37 | 0.00–43.43 | 0–0 | 0.00–0.00 |  |  |
|  | February | 60 | 0–0 | 0.00–0.00 | 0–19 | 0.00–23.92 |  |  |
|  | March | 64 | 0–0 | 0.00–0.00 | 0–14 | 0.00–17.02 |  |  |
|  | April | 63 | 0–0 | 0.00–0.00 | 0–11 | 0.00–14.70 |  |  |
|  | May | 67 | 0–0 | 0.00–0.00 | 0–7 | 0.00–8.53 |  |  |
|  | June | 62 | 0–0 | 0.00–0.00 | 0–6 | 0.00–8.17 |  |  |
|  | (d) Long-term care beds |  |  |  |  |  |  |  |
|  | January | 129 | 0–8 | 0.00–6.16 | 0–0 | 0.00–0.00 |  |  |
|  | February | 124 | 0–3 | 0.00–1.87 | 0–0 | 0.00–0.00 |  |  |
|  | March | 120 | 0–3 | 0.00–1.94 | 0–0 | 0.00–0.00 |  |  |
|  | April | 118 | 0–0 | 0.00–0.00 | 0–1 | 0.00–0.61 |  |  |
|  | May | 138 | 0–21 | 0.00–17.07 | 0–0 | 0.00–0.00 |  |  |
|  | June | 124 | 0–6 | 0.00–4.46 | 0–0 | 0.00–0.00 |  |  |
|  | (e) General beds |  |  |  |  |  |  |  |
|  | January | 18 | 0–0 | 0.00–0.00 | 0–1 | 0.00–1.29 |  |  |
|  | February | 18 | 0–0 | 0.00–0.00 | 0–1 | 0.00–1.64 |  |  |
|  | March | 18 | 0–0 | 0.00–0.00 | 0–1 | 0.00–1.96 |  |  |
|  | April | 18 | 0–0 | 0.00–0.00 | 0–1 | 0.00–2.96 |  |  |
|  | May | 19 | 0–2 | 0.00–6.63 | 0–0 | 0.00–0.00 |  |  |
|  | June | 17 | 0–0 | 0.00–0.00 | 0–1 | 0.00–3.37 |  |  |
|  | (f) LTCI care beds |  |  |  |  |  |  |  |
|  | January | 405 | 0–0 | 0.00–0.00 | 0–55 | 0.00–11.83 |  |  |
|  | February | 654 | 35–189 | 7.34–40.36 | 0–0 | 0.00–0.00 | * |  |
|  | March | 384 | 0–0 | 0.00–0.00 | 0–70 | 0.00–15.28 |  |  |
|  | April | 379 | 0–0 | 0.00–0.00 | 0–84 | 0.00–18.05 |  |  |
|  | May | 391 | 0–0 | 0.00–0.00 | 0–56 | 0.00–12.41 |  |  |
|  | June | 421 | 0–0 | 0.00–0.00 | 0–48 | 0.00–10.19 |  |  |
| Iwate | (1) Average number of outpatients per day at hospitals |  |  |  |  |  |  |  |
|  | General hospitals |  |  |  |  |  |  |  |
|  | January | 10495 | 0–0 | 0.00–0.00 | 0–555 | 0.00–5.02 |  |  |
|  | February | 10390 | 0–0 | 0.00–0.00 | 0–787 | 0.00–7.04 |  |  |
|  | March | 10741 | 0–0 | 0.00–0.00 | 0–494 | 0.00–4.39 |  |  |
|  | April | 10336 | 0–0 | 0.00–0.00 | 0–673 | 0.00–6.11 |  |  |
|  | May | 8790 | 0–0 | 0.00–0.00 | 1516–2275 | 13.70–20.55 |  | ** |
|  | June | 10630 | 0–0 | 0.00–0.00 | 0–504 | 0.00–4.53 |  |  |
|  | Psychiatric hospitals |  |  |  |  |  |  |  |
|  | January | 925 | 0–0 | 0.00–0.00 | 0–38 | 0.00–3.91 |  |  |
|  | February | 949 | 0–0 | 0.00–0.00 | 0–34 | 0.00–3.45 |  |  |
|  | March | 996 | 0–0 | 0.00–0.00 | 0–15 | 0.00–1.44 |  |  |
|  | April | 1008 | 0–14 | 0.00–1.37 | 0–0 | 0.00–0.00 |  |  |
|  | May | 858 | 0–0 | 0.00–0.00 | 69–146 | 6.87–14.53 |  | ** |
|  | June | 994 | 0–0 | 0.00–0.00 | 0–16 | 0.00–1.49 |  |  |
|  | (2) Average number of hospitalized patients per day |  |  |  |  |  |  |  |
|  | (b) Psychiatric care beds |  |  |  |  |  |  |  |
|  | January | 3369 | 0–68 | 0.00–2.04 | 0–0 | 0.00–0.00 |  |  |
|  | February | 3358 | 0–51 | 0.00–1.51 | 0–0 | 0.00–0.00 |  |  |
|  | March | 3346 | 0–37 | 0.00–1.11 | 0–0 | 0.00–0.00 |  |  |
|  | April | 3288 | 0–0 | 0.00–0.00 | 0–16 | 0.00–0.46 |  |  |
|  | May | 3240 | 0–0 | 0.00–0.00 | 0–52 | 0.00–1.56 |  |  |
|  | June | 3297 | 0–5 | 0.00–0.13 | 0–0 | 0.00–0.00 |  |  |
|  | (c) Tuberculosis care beds |  |  |  |  |  |  |  |
|  | January | 11 | 0–2 | 0.00–22.16 | 0–0 | 0.00–0.00 |  |  |
|  | February | 9 | 0–1 | 0.00–3.47 | 0–0 | 0.00–0.00 |  |  |
|  | March | 8 | 0–1 | 0.00–4.09 | 0–0 | 0.00–0.00 |  |  |
|  | April | 4 | 0–0 | 0.00–0.00 | 0–4 | 0.00–43.91 |  |  |
|  | May | 1 | 0–0 | 0.00–0.00 | 2–6 | 29.40–85.30 |  | ** |
|  | June | 2 | 0–0 | 0.00–0.00 | 2–7 | 23.83–76.17 |  | ** |
|  | (d) Long-term care beds |  |  |  |  |  |  |  |
|  | January | 2018 | 0–56 | 0.00–2.85 | 0–0 | 0.00–0.00 |  |  |
|  | February | 2032 | 0–60 | 0.00–3.04 | 0–0 | 0.00–0.00 |  |  |
|  | March | 1995 | 0–28 | 0.00–1.40 | 0–0 | 0.00–0.00 |  |  |
|  | April | 1921 | 0–0 | 0.00–0.00 | 0–32 | 0.00–1.62 |  |  |
|  | May | 1904 | 0–0 | 0.00–0.00 | 0–43 | 0.00–2.18 |  |  |
|  | June | 1909 | 0–0 | 0.00–0.00 | 0–35 | 0.00–1.77 |  |  |
|  | (e) General beds |  |  |  |  |  |  |  |
|  | January | 7170 | 0–0 | 0.00–0.00 | 0–144 | 0.00–1.97 |  |  |
|  | February | 7313 | 0–12 | 0.00–0.16 | 0–0 | 0.00–0.00 |  |  |
|  | March | 7004 | 0–0 | 0.00–0.00 | 0–220 | 0.00–3.04 |  |  |
|  | April | 6630 | 0–0 | 0.00–0.00 | 166–433 | 2.35–6.13 |  | ** |
|  | May | 6271 | 0–0 | 0.00–0.00 | 460–698 | 6.60–10.01 |  | ** |
|  | June | 6524 | 0–0 | 0.00–0.00 | 187–413 | 2.70–5.94 |  | ** |
|  | (f) LTCI care beds |  |  |  |  |  |  |  |
|  | January | 224 | 0–25 | 0.00–12.28 | 0–0 | 0.00–0.00 |  |  |
|  | February | 225 | 0–27 | 0.00–13.32 | 0–0 | 0.00–0.00 |  |  |
|  | March | 223 | 0–27 | 0.00–13.27 | 0–0 | 0.00–0.00 |  |  |
|  | April | 161 | 0–0 | 0.00–0.00 | 7–34 | 3.61–17.05 |  | ** |
|  | May | 162 | 0–0 | 0.00–0.00 | 2–28 | 1.05–14.68 |  | ** |
|  | June | 158 | 0–0 | 0.00–0.00 | 4–31 | 2.13–16.01 |  | ** |
|  | (3) Average length of hospital stays per patient |  |  |  |  |  |  |  |
|  | (b) Psychiatric care beds |  |  |  |  |  |  |  |
|  | January | 279 | 0–13 | 0.00–4.86 | 0–0 | 0.00–0.00 |  |  |
|  | February | 273 | 0–17 | 0.00–6.48 | 0–0 | 0.00–0.00 |  |  |
|  | March | 259 | 0–6 | 0.00–2.11 | 0–0 | 0.00–0.00 |  |  |
|  | April | 265 | 0–18 | 0.00–7.01 | 0–0 | 0.00–0.00 |  |  |
|  | May | 288 | 13–44 | 4.99–17.94 | 0–0 | 0.00–0.00 | * |  |
|  | June | 234 | 0–0 | 0.00–0.00 | 0–6 | 0.00–2.22 |  |  |
|  | (c) Tuberculosis care beds |  |  |  |  |  |  |  |
|  | January | 61 | 0–5 | 0.00–7.62 | 0–0 | 0.00–0.00 |  |  |
|  | February | 77 | 0–17 | 0.00–27.52 | 0–0 | 0.00–0.00 |  |  |
|  | March | 40 | 0–0 | 0.00–0.00 | 0–17 | 0.00–29.79 |  |  |
|  | April | 17 | 0–0 | 0.00–0.00 | 12–38 | 20.94–68.59 |  | ** |
|  | May | 26 | 0–0 | 0.00–0.00 | 0–26 | 0.00–49.29 |  |  |
|  | June | 12 | 0–0 | 0.00–0.00 | 17–47 | 27.72–79.81 |  | ** |
|  | (d) Long-term care beds |  |  |  |  |  |  |  |
|  | January | 136 | 2–24 | 1.61–21.21 | 0–0 | 0.00–0.00 | * |  |
|  | February | 125 | 0–16 | 0.00–14.08 | 0–0 | 0.00–0.00 |  |  |
|  | March | 127 | 0–20 | 0.00–17.90 | 0–0 | 0.00–0.00 |  |  |
|  | April | 112 | 0–3 | 0.00–2.40 | 0–0 | 0.00–0.00 |  |  |
|  | May | 142 | 10–32 | 8.29–28.20 | 0–0 | 0.00–0.00 | * |  |
|  | June | 132 | 0–21 | 0.00–18.31 | 0–0 | 0.00–0.00 |  |  |
|  | (e) General beds |  |  |  |  |  |  |  |
|  | January | 19 | 0–1 | 0.00–3.48 | 0–0 | 0.00–0.00 |  |  |
|  | February | 19 | 0–1 | 0.00–1.87 | 0–0 | 0.00–0.00 |  |  |
|  | March | 18 | 0–1 | 0.00–0.59 | 0–0 | 0.00–0.00 |  |  |
|  | April | 19 | 0–1 | 0.00–1.70 | 0–0 | 0.00–0.00 |  |  |
|  | May | 20 | 0–3 | 0.00–13.03 | 0–0 | 0.00–0.00 |  |  |
|  | June | 18 | 0–1 | 0.00–0.67 | 0–0 | 0.00–0.00 |  |  |
|  | (f) LTCI care beds |  |  |  |  |  |  |  |
|  | January | 421 | 132–237 | 71.55–128.45 | 0–0 | 0.00–0.00 | * |  |
|  | February | 256 | 0–80 | 0.00–44.96 | 0–0 | 0.00–0.00 |  |  |
|  | March | 433 | 132–238 | 67.43–121.67 | 0–0 | 0.00–0.00 | * |  |
|  | April | 95 | 0–0 | 0.00–0.00 | 18–97 | 9.41–50.36 |  | ** |
|  | May | 502 | 215–317 | 115.38–170.58 | 0–0 | 0.00–0.00 | * |  |
|  | June | 296 | 27–122 | 15.11–69.68 | 0–0 | 0.00–0.00 | * |  |
| Miyagi | (1) Average number of outpatients per day at hospitals |  |  |  |  |  |  |  |
|  | General hospitals |  |  |  |  |  |  |  |
|  | January | 18531 | 0–0 | 0.00–0.00 | 0–1317 | 0.00–6.63 |  |  |
|  | February | 18537 | 0–0 | 0.00–0.00 | 0–1544 | 0.00–7.69 |  |  |
|  | March | 18449 | 0–0 | 0.00–0.00 | 263–2023 | 1.28–9.88 |  | ** |
|  | April | 16559 | 0–0 | 0.00–0.00 | 2005–3536 | 9.98–17.60 |  | ** |
|  | May | 14471 | 0–0 | 0.00–0.00 | 4270–5981 | 20.88–29.24 |  | ** |
|  | June | 18743 | 0–0 | 0.00–0.00 | 276–2063 | 1.33–9.92 |  | ** |
|  | Psychiatric hospitals |  |  |  |  |  |  |  |
|  | January | 1280 | 0–0 | 0.00–0.00 | 0–74 | 0.00–5.46 |  |  |
|  | February | 1323 | 0–0 | 0.00–0.00 | 0–43 | 0.00–3.10 |  |  |
|  | March | 1312 | 0–0 | 0.00–0.00 | 0–99 | 0.00–7.01 |  |  |
|  | April | 1306 | 0–0 | 0.00–0.00 | 0–66 | 0.00–4.76 |  |  |
|  | May | 1097 | 0–0 | 0.00–0.00 | 183–284 | 13.25–20.55 |  | ** |
|  | June | 1345 | 0–0 | 0.00–0.00 | 0–27 | 0.00–1.94 |  |  |
|  | (2) Average number of hospitalized patients per day |  |  |  |  |  |  |  |
|  | (b) Psychiatric care beds |  |  |  |  |  |  |  |
|  | January | 5068 | 0–0 | 0.00–0.00 | 0–103 | 0.00–1.99 |  |  |
|  | February | 5071 | 0–0 | 0.00–0.00 | 0–95 | 0.00–1.84 |  |  |
|  | March | 5079 | 0–0 | 0.00–0.00 | 0–90 | 0.00–1.73 |  |  |
|  | April | 5039 | 0–0 | 0.00–0.00 | 0–115 | 0.00–2.22 |  |  |
|  | May | 4955 | 0–0 | 0.00–0.00 | 68–208 | 1.32–4.02 |  | ** |
|  | June | 5059 | 0–0 | 0.00–0.00 | 0–116 | 0.00–2.23 |  |  |
|  | (c) Tuberculosis care beds |  |  |  |  |  |  |  |
|  | January | 7 | 0–1 | 0.00–3.13 | 0–0 | 0.00–0.00 |  |  |
|  | February | 10 | 0–4 | 0.00–48.46 | 0–0 | 0.00–0.00 |  |  |
|  | March | 11 | 0–4 | 0.00–51.73 | 0–0 | 0.00–0.00 |  |  |
|  | April | 11 | 0–4 | 0.00–38.06 | 0–0 | 0.00–0.00 |  |  |
|  | May | 10 | 0–2 | 0.00–23.04 | 0–0 | 0.00–0.00 |  |  |
|  | June | 9 | 0–2 | 0.00–17.35 | 0–0 | 0.00–0.00 |  |  |
|  | (d) Long-term care beds |  |  |  |  |  |  |  |
|  | January | 2824 | 0–0 | 0.00–0.00 | 0–42 | 0.00–1.44 |  |  |
|  | February | 2866 | 0–0 | 0.00–0.00 | 0–13 | 0.00–0.44 |  |  |
|  | March | 2843 | 0–0 | 0.00–0.00 | 0–47 | 0.00–1.60 |  |  |
|  | April | 2759 | 0–0 | 0.00–0.00 | 19–123 | 0.66–4.26 |  | ** |
|  | May | 2693 | 0–0 | 0.00–0.00 | 83–187 | 2.88–6.49 |  | ** |
|  | June | 2618 | 0–0 | 0.00–0.00 | 157–262 | 5.45–9.08 |  | ** |
|  | (e) General beds |  |  |  |  |  |  |  |
|  | January | 11178 | 0–0 | 0.00–0.00 | 0–382 | 0.00–3.30 |  |  |
|  | February | 11522 | 0–0 | 0.00–0.00 | 0–175 | 0.00–1.49 |  |  |
|  | March | 11116 | 0–0 | 0.00–0.00 | 162–536 | 1.39–4.59 |  | ** |
|  | April | 10219 | 0–0 | 0.00–0.00 | 714–1145 | 6.28–10.07 |  | ** |
|  | May | 9596 | 0–0 | 0.00–0.00 | 1330–1658 | 11.82–14.73 |  | ** |
|  | June | 10339 | 0–0 | 0.00–0.00 | 622–958 | 5.51–8.48 |  | ** |
|  | (f) LTCI care beds |  |  |  |  |  |  |  |
|  | January | 79 | 0–0 | 0.00–0.00 | 0–15 | 0.00–15.82 |  |  |
|  | February | 84 | 0–0 | 0.00–0.00 | 0–8 | 0.00–8.15 |  |  |
|  | March | 83 | 0–0 | 0.00–0.00 | 0–6 | 0.00–5.74 |  |  |
|  | April | 55 | 0–0 | 0.00–0.00 | 13–31 | 15.29–35.32 |  | ** |
|  | May | 55 | 0–0 | 0.00–0.00 | 10–28 | 12.19–32.97 |  | ** |
|  | June | 19 | 0–0 | 0.00–0.00 | 45–62 | 56.09–76.32 |  | ** |
|  | (3) Average length of hospital stays per patient |  |  |  |  |  |  |  |
|  | (b) Psychiatric care beds |  |  |  |  |  |  |  |
|  | January | 313 | 3–37 | 0.83–13.02 | 0–0 | 0.00–0.00 | * |  |
|  | February | 308 | 3–37 | 1.11–13.54 | 0–0 | 0.00–0.00 | * |  |
|  | March | 285 | 0–20 | 0.00–7.48 | 0–0 | 0.00–0.00 |  |  |
|  | April | 281 | 0–14 | 0.00–5.13 | 0–0 | 0.00–0.00 |  |  |
|  | May | 348 | 49–83 | 18.42–31.07 | 0–0 | 0.00–0.00 | * |  |
|  | June | 287 | 0–21 | 0.00–7.60 | 0–0 | 0.00–0.00 |  |  |
|  | (c) Tuberculosis care beds |  |  |  |  |  |  |  |
|  | January | 48 | 0–0 | 0.00–0.00 | 0–38 | 0.00–43.74 |  |  |
|  | February | 80 | 0–1 | 0.00–0.48 | 0–0 | 0.00–0.00 |  |  |
|  | March | 101 | 0–16 | 0.00–17.96 | 0–0 | 0.00–0.00 |  |  |
|  | April | 34 | 0–0 | 0.00–0.00 | 12–54 | 13.78–60.97 |  | ** |
|  | May | 63 | 0–0 | 0.00–0.00 | 0–19 | 0.00–23.18 |  |  |
|  | June | 61 | 0–0 | 0.00–0.00 | 0–19 | 0.00–23.03 |  |  |
|  | (d) Long-term care beds |  |  |  |  |  |  |  |
|  | January | 111 | 0–11 | 0.00–10.00 | 0–0 | 0.00–0.00 |  |  |
|  | February | 110 | 0–9 | 0.00–8.22 | 0–0 | 0.00–0.00 |  |  |
|  | March | 100 | 0–2 | 0.00–1.14 | 0–0 | 0.00–0.00 |  |  |
|  | April | 107 | 0–7 | 0.00–5.94 | 0–0 | 0.00–0.00 |  |  |
|  | May | 120 | 0–16 | 0.00–15.44 | 0–0 | 0.00–0.00 |  |  |
|  | June | 114 | 0–8 | 0.00–6.85 | 0–0 | 0.00–0.00 |  |  |
|  | (e) General beds |  |  |  |  |  |  |  |
|  | January | 16 | 0–1 | 0.00–3.17 | 0–0 | 0.00–0.00 |  |  |
|  | February | 15 | 0–1 | 0.00–1.23 | 0–0 | 0.00–0.00 |  |  |
|  | March | 15 | 0–1 | 0.00–0.41 | 0–0 | 0.00–0.00 |  |  |
|  | April | 16 | 0–1 | 0.00–5.59 | 0–0 | 0.00–0.00 |  |  |
|  | May | 17 | 0–3 | 0.00–13.99 | 0–0 | 0.00–0.00 |  |  |
|  | June | 15 | 0–1 | 0.00–1.49 | 0–0 | 0.00–0.00 |  |  |
|  | (f) LTCI care beds |  |  |  |  |  |  |  |
|  | January | 135 | 3–31 | 2.28–28.51 | 0–0 | 0.00–0.00 | * |  |
|  | February | 143 | 3–36 | 2.22–32.62 | 0–0 | 0.00–0.00 | * |  |
|  | March | 109 | 0–0 | 0.00–0.00 | 0–1 | 0.00–0.14 |  |  |
|  | April | 103 | 0–0 | 0.00–0.00 | 0–15 | 0.00–12.51 |  |  |
|  | May | 126 | 0–11 | 0.00–8.69 | 0–0 | 0.00–0.00 |  |  |
|  | June | 61 | 0–0 | 0.00–0.00 | 27–59 | 22.26–48.62 |  | ** |
| Akita | (1) Average number of outpatients per day at hospitals |  |  |  |  |  |  |  |
|  | General hospitals |  |  |  |  |  |  |  |
|  | January | 11024 | 0–0 | 0.00–0.00 | 0–425 | 0.00–3.71 |  |  |
|  | February | 10733 | 0–0 | 0.00–0.00 | 7–808 | 0.06–6.99 |  | ** |
|  | March | 11273 | 0–0 | 0.00–0.00 | 0–468 | 0.00–3.98 |  |  |
|  | April | 10650 | 0–0 | 0.00–0.00 | 137–906 | 1.19–7.83 |  | ** |
|  | May | 9254 | 0–0 | 0.00–0.00 | 1550–2352 | 13.36–20.26 |  | ** |
|  | June | 11225 | 0–0 | 0.00–0.00 | 0–432 | 0.00–3.70 |  |  |
|  | Psychiatric hospitals |  |  |  |  |  |  |  |
|  | January | 490 | 0–0 | 0.00–0.00 | 0–24 | 0.00–4.62 |  |  |
|  | February | 501 | 0–0 | 0.00–0.00 | 0–17 | 0.00–3.17 |  |  |
|  | March | 506 | 0–0 | 0.00–0.00 | 0–30 | 0.00–5.44 |  |  |
|  | April | 518 | 0–0 | 0.00–0.00 | 0–7 | 0.00–1.19 |  |  |
|  | May | 446 | 0–0 | 0.00–0.00 | 37–82 | 7.02–15.42 |  | ** |
|  | June | 525 | 0–0 | 0.00–0.00 | 0–1 | 0.00–0.18 |  |  |
|  | (2) Average number of hospitalized patients per day |  |  |  |  |  |  |  |
|  | (b) Psychiatric care beds |  |  |  |  |  |  |  |
|  | January | 3360 | 0–30 | 0.00–0.89 | 0–0 | 0.00–0.00 |  |  |
|  | February | 3361 | 0–24 | 0.00–0.71 | 0–0 | 0.00–0.00 |  |  |
|  | March | 3341 | 0–7 | 0.00–0.20 | 0–0 | 0.00–0.00 |  |  |
|  | April | 3283 | 0–0 | 0.00–0.00 | 0–44 | 0.00–1.30 |  |  |
|  | May | 3265 | 0–0 | 0.00–0.00 | 0–54 | 0.00–1.62 |  |  |
|  | June | 3302 | 0–0 | 0.00–0.00 | 0–17 | 0.00–0.50 |  |  |
|  | (c) Tuberculosis care beds |  |  |  |  |  |  |  |
|  | January | 10 | 0–3 | 0.00–35.45 | 0–0 | 0.00–0.00 |  |  |
|  | February | 9 | 0–3 | 0.00–36.55 | 0–0 | 0.00–0.00 |  |  |
|  | March | 10 | 0–4 | 0.00–59.43 | 0–0 | 0.00–0.00 |  |  |
|  | April | 9 | 0–3 | 0.00–39.23 | 0–0 | 0.00–0.00 |  |  |
|  | May | 9 | 0–3 | 0.00–35.36 | 0–0 | 0.00–0.00 |  |  |
|  | June | 7 | 0–0 | 0.00–0.00 | 0–1 | 0.00–2.69 |  |  |
|  | (d) Long-term care beds |  |  |  |  |  |  |  |
|  | January | 1819 | 0–0 | 0.00–0.00 | 17–102 | 0.88–5.31 |  | ** |
|  | February | 1849 | 0–0 | 0.00–0.00 | 0–79 | 0.00–4.07 |  |  |
|  | March | 1840 | 0–0 | 0.00–0.00 | 1–86 | 0.05–4.45 |  | ** |
|  | April | 1699 | 0–0 | 0.00–0.00 | 128–213 | 6.70–11.11 |  | ** |
|  | May | 1669 | 0–0 | 0.00–0.00 | 142–227 | 7.49–11.93 |  | ** |
|  | June | 1681 | 0–0 | 0.00–0.00 | 113–197 | 6.02–10.48 |  | ** |
|  | (e) General beds |  |  |  |  |  |  |  |
|  | January | 6210 | 0–0 | 0.00–0.00 | 9–243 | 0.14–3.76 |  | ** |
|  | February | 6436 | 0–0 | 0.00–0.00 | 0–16 | 0.00–0.24 |  |  |
|  | March | 6120 | 0–0 | 0.00–0.00 | 47–249 | 0.74–3.91 |  | ** |
|  | April | 5769 | 0–0 | 0.00–0.00 | 245–472 | 3.93–7.55 |  | ** |
|  | May | 5435 | 0–0 | 0.00–0.00 | 587–750 | 9.49–12.12 |  | ** |
|  | June | 5797 | 0–0 | 0.00–0.00 | 210–397 | 3.39–6.41 |  | ** |
|  | (f) LTCI care beds |  |  |  |  |  |  |  |
|  | January | 177 | 0–0 | 0.00–0.00 | 48–97 | 17.52–35.39 |  | ** |
|  | February | 169 | 0–0 | 0.00–0.00 | 53–103 | 19.50–37.81 |  | ** |
|  | March | 167 | 0–0 | 0.00–0.00 | 44–99 | 16.60–36.99 |  | ** |
|  | April | 52 | 0–0 | 0.00–0.00 | 139–202 | 54.93–79.45 |  | ** |
|  | May | 52 | 0–0 | 0.00–0.00 | 133–197 | 53.52–79.07 |  | ** |
|  | June | 52 | 0–0 | 0.00–0.00 | 111–177 | 48.48–77.29 |  | ** |
|  | (3) Average length of hospital stays per patient |  |  |  |  |  |  |  |
|  | (b) Psychiatric care beds |  |  |  |  |  |  |  |
|  | January | 310 | 2–36 | 0.55–12.76 | 0–0 | 0.00–0.00 | * |  |
|  | February | 300 | 0–21 | 0.00–7.34 | 0–0 | 0.00–0.00 |  |  |
|  | March | 262 | 0–0 | 0.00–0.00 | 0–6 | 0.00–1.91 |  |  |
|  | April | 261 | 0–1 | 0.00–0.10 | 0–0 | 0.00–0.00 |  |  |
|  | May | 314 | 27–60 | 10.54–23.42 | 0–0 | 0.00–0.00 | * |  |
|  | June | 267 | 0–14 | 0.00–5.41 | 0–0 | 0.00–0.00 |  |  |
|  | (c) Tuberculosis care beds |  |  |  |  |  |  |  |
|  | January | 108 | 0–0 | 0.00–0.00 | 0–8 | 0.00–6.73 |  |  |
|  | February | 47 | 0–0 | 0.00–0.00 | 4–60 | 3.09–56.22 |  | ** |
|  | March | 87 | 0–0 | 0.00–0.00 | 0–22 | 0.00–20.06 |  |  |
|  | April | 108 | 0–0 | 0.00–0.00 | 0–9 | 0.00–7.29 |  |  |
|  | May | 114 | 0–0 | 0.00–0.00 | 0–1 | 0.00–0.32 |  |  |
|  | June | 60 | 0–0 | 0.00–0.00 | 0–42 | 0.00–41.10 |  |  |
|  | (d) Long-term care beds |  |  |  |  |  |  |  |
|  | January | 146 | 0–5 | 0.00–3.05 | 0–0 | 0.00–0.00 |  |  |
|  | February | 141 | 0–0 | 0.00–0.00 | 0–1 | 0.00–0.60 |  |  |
|  | March | 121 | 0–0 | 0.00–0.00 | 0–18 | 0.00–12.83 |  |  |
|  | April | 118 | 0–0 | 0.00–0.00 | 0–20 | 0.00–14.15 |  |  |
|  | May | 138 | 0–4 | 0.00–2.25 | 0–0 | 0.00–0.00 |  |  |
|  | June | 120 | 0–0 | 0.00–0.00 | 0–15 | 0.00–10.60 |  |  |
|  | (e) General beds |  |  |  |  |  |  |  |
|  | January | 19 | 0–1 | 0.00–1.08 | 0–0 | 0.00–0.00 |  |  |
|  | February | 18 | 0–0 | 0.00–0.00 | 0–1 | 0.00–1.50 |  |  |
|  | March | 18 | 0–0 | 0.00–0.00 | 0–1 | 0.00–2.63 |  |  |
|  | April | 18 | 0–0 | 0.00–0.00 | 0–1 | 0.00–1.60 |  |  |
|  | May | 19 | 0–2 | 0.00–7.35 | 0–0 | 0.00–0.00 |  |  |
|  | June | 17 | 0–0 | 0.00–0.00 | 0–1 | 0.00–4.02 |  |  |
|  | (f) LTCI care beds |  |  |  |  |  |  |  |
|  | January | 844 | 108–352 | 21.92–71.56 | 0–0 | 0.00–0.00 | * |  |
|  | February | 1631 | 918–1153 | 191.81–240.84 | 0–0 | 0.00–0.00 | * |  |
|  | March | 85 | 0–0 | 0.00–0.00 | 223–404 | 45.51–82.66 |  | ** |
|  | April | 390 | 0–0 | 0.00–0.00 | 0–97 | 0.00–19.79 |  |  |
|  | May | 806 | 86–316 | 17.52–64.19 | 0–0 | 0.00–0.00 | * |  |
|  | June | 390 | 0–0 | 0.00–0.00 | 0–85 | 0.00–17.86 |  |  |
| Yamagata | (1) Average number of outpatients per day at hospitals |  |  |  |  |  |  |  |
|  | General hospitals |  |  |  |  |  |  |  |
|  | January | 10032 | 0–0 | 0.00–0.00 | 0–417 | 0.00–3.99 |  |  |
|  | February | 9913 | 0–0 | 0.00–0.00 | 0–665 | 0.00–6.28 |  |  |
|  | March | 10285 | 0–0 | 0.00–0.00 | 0–484 | 0.00–4.49 |  |  |
|  | April | 9119 | 0–0 | 0.00–0.00 | 814–1492 | 7.67–14.06 |  | ** |
|  | May | 7861 | 0–0 | 0.00–0.00 | 2073–2792 | 19.46–26.20 |  | ** |
|  | June | 9855 | 0–0 | 0.00–0.00 | 135–912 | 1.25–8.47 |  | ** |
|  | Psychiatric hospitals |  |  |  |  |  |  |  |
|  | January | 997 | 0–0 | 0.00–0.00 | 0–57 | 0.00–5.33 |  |  |
|  | February | 1049 | 0–0 | 0.00–0.00 | 0–23 | 0.00–2.09 |  |  |
|  | March | 1076 | 0–0 | 0.00–0.00 | 0–31 | 0.00–2.79 |  |  |
|  | April | 904 | 0–0 | 0.00–0.00 | 119–192 | 10.87–17.45 |  | ** |
|  | May | 830 | 0–0 | 0.00–0.00 | 195–275 | 17.66–24.83 |  | ** |
|  | June | 1078 | 0–0 | 0.00–0.00 | 0–35 | 0.00–3.06 |  |  |
|  | (2) Average number of hospitalized patients per day |  |  |  |  |  |  |  |
|  | (b) Psychiatric care beds |  |  |  |  |  |  |  |
|  | January | 3096 | 0–7 | 0.00–0.22 | 0–0 | 0.00–0.00 |  |  |
|  | February | 3097 | 0–10 | 0.00–0.32 | 0–0 | 0.00–0.00 |  |  |
|  | March | 3055 | 0–0 | 0.00–0.00 | 0–22 | 0.00–0.71 |  |  |
|  | April | 3016 | 0–0 | 0.00–0.00 | 0–49 | 0.00–1.60 |  |  |
|  | May | 3010 | 0–0 | 0.00–0.00 | 0–54 | 0.00–1.74 |  |  |
|  | June | 3068 | 0–0 | 0.00–0.00 | 0–3 | 0.00–0.07 |  |  |
|  | (c) Tuberculosis care beds |  |  |  |  |  |  |  |
|  | January | 0 | NA | NA | NA | NA |  |  |
|  | February | 0 | NA | NA | NA | NA |  |  |
|  | March | 0 | NA | NA | NA | NA |  |  |
|  | April | 0 | NA | NA | NA | NA |  |  |
|  | May | 0 | NA | NA | NA | NA |  |  |
|  | June | 0 | NA | NA | NA | NA |  |  |
|  | (d) Long-term care beds |  |  |  |  |  |  |  |
|  | January | 1861 | 0–61 | 0.00–3.35 | 0–0 | 0.00–0.00 |  |  |
|  | February | 1866 | 0–51 | 0.00–2.79 | 0–0 | 0.00–0.00 |  |  |
|  | March | 1832 | 0–12 | 0.00–0.64 | 0–0 | 0.00–0.00 |  |  |
|  | April | 1822 | 0–21 | 0.00–1.11 | 0–0 | 0.00–0.00 |  |  |
|  | May | 1807 | 0–16 | 0.00–0.85 | 0–0 | 0.00–0.00 |  |  |
|  | June | 1831 | 0–46 | 0.00–2.53 | 0–0 | 0.00–0.00 |  |  |
|  | (e) General beds |  |  |  |  |  |  |  |
|  | January | 6240 | 0–0 | 0.00–0.00 | 112–337 | 1.70–5.12 |  | ** |
|  | February | 6436 | 0–0 | 0.00–0.00 | 9–177 | 0.14–2.67 |  | ** |
|  | March | 6065 | 0–0 | 0.00–0.00 | 315–499 | 4.80–7.59 |  | ** |
|  | April | 5573 | 0–0 | 0.00–0.00 | 629–848 | 9.80–13.20 |  | ** |
|  | May | 5419 | 0–0 | 0.00–0.00 | 771–937 | 12.13–14.74 |  | ** |
|  | June | 5670 | 0–0 | 0.00–0.00 | 503–666 | 7.94–10.51 |  | ** |
|  | (f) LTCI care beds |  |  |  |  |  |  |  |
|  | January | 20 | 0–2 | 0.00–5.63 | 0–0 | 0.00–0.00 |  |  |
|  | February | 19 | 0–0 | 0.00–0.00 | 0–0 | 0.00–0.00 |  |  |
|  | March | 19 | 0–0 | 0.00–0.00 | 0–0 | 0.00–0.00 |  |  |
|  | April | 20 | 0–1 | 0.00–5.26 | 0–0 | 0.00–0.00 |  |  |
|  | May | 19 | 0–0 | 0.00–0.00 | 0–0 | 0.00–0.00 |  |  |
|  | June | 19 | 0–0 | 0.00–0.00 | 0–0 | 0.00–0.00 |  |  |
|  | (3) Average length of hospital stays per patient |  |  |  |  |  |  |  |
|  | (b) Psychiatric care beds |  |  |  |  |  |  |  |
|  | January | 261 | 0–20 | 0.00–8.18 | 0–0 | 0.00–0.00 |  |  |
|  | February | 241 | 0–10 | 0.00–3.99 | 0–0 | 0.00–0.00 |  |  |
|  | March | 222 | 0–0 | 0.00–0.00 | 0–2 | 0.00–0.50 |  |  |
|  | April | 250 | 0–31 | 0.00–13.78 | 0–0 | 0.00–0.00 |  |  |
|  | May | 272 | 29–59 | 13.61–27.64 | 0–0 | 0.00–0.00 | * |  |
|  | June | 222 | 0–13 | 0.00–5.73 | 0–0 | 0.00–0.00 |  |  |
|  | (c) Tuberculosis care beds |  |  |  |  |  |  |  |
|  | January | 0 | NA | NA | NA | NA |  |  |
|  | February | 0 | NA | NA | NA | NA |  |  |
|  | March | 0 | NA | NA | NA | NA |  |  |
|  | April | 0 | NA | NA | NA | NA |  |  |
|  | May | 0 | NA | NA | NA | NA |  |  |
|  | June | 0 | NA | NA | NA | NA |  |  |
|  | (d) Long-term care beds |  |  |  |  |  |  |  |
|  | January | 110 | 0–8 | 0.00–7.42 | 0–0 | 0.00–0.00 |  |  |
|  | February | 104 | 0–2 | 0.00–1.27 | 0–0 | 0.00–0.00 |  |  |
|  | March | 103 | 0–5 | 0.00–4.84 | 0–0 | 0.00–0.00 |  |  |
|  | April | 103 | 0–6 | 0.00–5.30 | 0–0 | 0.00–0.00 |  |  |
|  | May | 112 | 0–17 | 0.00–16.89 | 0–0 | 0.00–0.00 |  |  |
|  | June | 101 | 0–3 | 0.00–2.27 | 0–0 | 0.00–0.00 |  |  |
|  | (e) General beds |  |  |  |  |  |  |  |
|  | January | 17 | 0–0 | 0.00–0.00 | 0–1 | 0.00–0.29 |  |  |
|  | February | 17 | 0–0 | 0.00–0.00 | 0–1 | 0.00–2.65 |  |  |
|  | March | 17 | 0–0 | 0.00–0.00 | 0–1 | 0.00–1.27 |  |  |
|  | April | 18 | 0–1 | 0.00–5.63 | 0–0 | 0.00–0.00 |  |  |
|  | May | 19 | 0–3 | 0.00–16.29 | 0–0 | 0.00–0.00 |  |  |
|  | June | 16 | 0–1 | 0.00–1.44 | 0–0 | 0.00–0.00 |  |  |
|  | (f) LTCI care beds |  |  |  |  |  |  |  |
|  | January | 151 | 0–53 | 0.00–53.26 | 0–0 | 0.00–0.00 |  |  |
|  | February | 274 | 87–179 | 90.67–185.55 | 0–0 | 0.00–0.00 | * |  |
|  | March | 198 | 10–102 | 9.62–105.07 | 0–0 | 0.00–0.00 | * |  |
|  | April | 168 | 0–66 | 0.00–63.69 | 0–0 | 0.00–0.00 |  |  |
|  | May | 401 | 183–284 | 156.37–242.96 | 0–0 | 0.00–0.00 | * |  |
|  | June | 571 | 354–456 | 305.38–392.57 | 0–0 | 0.00–0.00 | * |  |
| Fukushima | (1) Average number of outpatients per day at hospitals |  |  |  |  |  |  |  |
|  | General hospitals |  |  |  |  |  |  |  |
|  | January | 17192 | 0–0 | 0.00–0.00 | 0–515 | 0.00–2.90 |  |  |
|  | February | 17428 | 0–0 | 0.00–0.00 | 0–362 | 0.00–2.03 |  |  |
|  | March | 17372 | 0–0 | 0.00–0.00 | 0–731 | 0.00–4.03 |  |  |
|  | April | 15892 | 0–0 | 0.00–0.00 | 662–1844 | 3.73–10.39 |  | ** |
|  | May | 13474 | 0–0 | 0.00–0.00 | 3182–4395 | 17.81–24.59 |  | ** |
|  | June | 16774 | 0–0 | 0.00–0.00 | 0–1320 | 0.00–7.29 |  |  |
|  | Psychiatric hospitals |  |  |  |  |  |  |  |
|  | January | 1127 | 0–0 | 0.00–0.00 | 0–85 | 0.00–6.98 |  |  |
|  | February | 1186 | 0–0 | 0.00–0.00 | 0–41 | 0.00–3.26 |  |  |
|  | March | 1186 | 0–0 | 0.00–0.00 | 0–81 | 0.00–6.32 |  |  |
|  | April | 1143 | 0–0 | 0.00–0.00 | 9–95 | 0.73–7.64 |  | ** |
|  | May | 981 | 0–0 | 0.00–0.00 | 171–261 | 13.77–21.01 |  | ** |
|  | June | 1145 | 0–0 | 0.00–0.00 | 16–100 | 1.29–7.98 |  | ** |
|  | (2) Average number of hospitalized patients per day |  |  |  |  |  |  |  |
|  | (b) Psychiatric care beds |  |  |  |  |  |  |  |
|  | January | 4542 | 0–9 | 0.00–0.18 | 0–0 | 0.00–0.00 |  |  |
|  | February | 4533 | 0–0 | 0.00–0.00 | 0–18 | 0.00–0.38 |  |  |
|  | March | 4513 | 0–0 | 0.00–0.00 | 0–42 | 0.00–0.92 |  |  |
|  | April | 4428 | 0–0 | 0.00–0.00 | 0–119 | 0.00–2.61 |  |  |
|  | May | 4339 | 0–0 | 0.00–0.00 | 67–199 | 1.48–4.37 |  | ** |
|  | June | 4408 | 0–0 | 0.00–0.00 | 0–132 | 0.00–2.89 |  |  |
|  | (c) Tuberculosis care beds |  |  |  |  |  |  |  |
|  | January | 9 | 0–1 | 0.00–7.78 | 0–0 | 0.00–0.00 |  |  |
|  | February | 5 | 0–0 | 0.00–0.00 | 0–3 | 0.00–33.14 |  |  |
|  | March | 1 | 0–0 | 0.00–0.00 | 2–6 | 29.03–85.48 |  | ** |
|  | April | 12 | 0–5 | 0.00–70.02 | 0–0 | 0.00–0.00 |  |  |
|  | May | 11 | 0–4 | 0.00–49.61 | 0–0 | 0.00–0.00 |  |  |
|  | June | 6 | 0–0 | 0.00–0.00 | 0–2 | 0.00–24.57 |  |  |
|  | (d) Long-term care beds |  |  |  |  |  |  |  |
|  | January | 2551 | 0–0 | 0.00–0.00 | 80–182 | 2.93–6.65 |  | ** |
|  | February | 2580 | 0–0 | 0.00–0.00 | 25–136 | 0.92–4.97 |  | ** |
|  | March | 2584 | 0–0 | 0.00–0.00 | 0–104 | 0.00–3.86 |  |  |
|  | April | 2473 | 0–0 | 0.00–0.00 | 55–169 | 2.08–6.40 |  | ** |
|  | May | 2435 | 0–0 | 0.00–0.00 | 26–140 | 1.01–5.43 |  | ** |
|  | June | 2440 | 0–0 | 0.00–0.00 | 5–111 | 0.20–4.33 |  | ** |
|  | (e) General beds |  |  |  |  |  |  |  |
|  | January | 10340 | 0–0 | 0.00–0.00 | 0–275 | 0.00–2.59 |  |  |
|  | February | 10623 | 0–0 | 0.00–0.00 | 0–100 | 0.00–0.92 |  |  |
|  | March | 10209 | 0–0 | 0.00–0.00 | 157–477 | 1.47–4.46 |  | ** |
|  | April | 9523 | 0–0 | 0.00–0.00 | 520–905 | 4.99–8.68 |  | ** |
|  | May | 8939 | 0–0 | 0.00–0.00 | 1122–1391 | 10.86–13.46 |  | ** |
|  | June | 9438 | 0–0 | 0.00–0.00 | 588–873 | 5.70–8.47 |  | ** |
|  | (f) LTCI care beds |  |  |  |  |  |  |  |
|  | January | 194 | 0–0 | 0.00–0.00 | 116–152 | 33.56–43.87 |  | ** |
|  | February | 198 | 0–0 | 0.00–0.00 | 109–145 | 31.81–42.21 |  | ** |
|  | March | 198 | 0–0 | 0.00–0.00 | 96–134 | 28.94–40.31 |  | ** |
|  | April | 120 | 0–0 | 0.00–0.00 | 115–173 | 39.33–58.96 |  | ** |
|  | May | 123 | 0–0 | 0.00–0.00 | 95–154 | 34.34–55.54 |  | ** |
|  | June | 126 | 0–0 | 0.00–0.00 | 86–147 | 31.61–53.69 |  | ** |
|  | (3) Average length of hospital stays per patient |  |  |  |  |  |  |  |
|  | (b) Psychiatric care beds |  |  |  |  |  |  |  |
|  | January | 355 | 13–48 | 3.93–15.45 | 0–0 | 0.00–0.00 | * |  |
|  | February | 316 | 0–13 | 0.00–4.15 | 0–0 | 0.00–0.00 |  |  |
|  | March | 313 | 0–18 | 0.00–5.83 | 0–0 | 0.00–0.00 |  |  |
|  | April | 352 | 19–54 | 6.34–18.03 | 0–0 | 0.00–0.00 | * |  |
|  | May | 378 | 46–80 | 15.21–26.77 | 0–0 | 0.00–0.00 | * |  |
|  | June | 296 | 0–0 | 0.00–0.00 | 0–2 | 0.00–0.59 |  |  |
|  | (c) Tuberculosis care beds |  |  |  |  |  |  |  |
|  | January | 116 | 0–40 | 0.00–52.27 | 0–0 | 0.00–0.00 |  |  |
|  | February | 35 | 0–0 | 0.00–0.00 | 0–43 | 0.00–55.07 |  |  |
|  | March | 30 | 0–0 | 0.00–0.00 | 0–43 | 0.00–58.88 |  |  |
|  | April | 18 | 0–0 | 0.00–0.00 | 20–66 | 23.16–78.53 |  | ** |
|  | May | 78 | 0–11 | 0.00–15.61 | 0–0 | 0.00–0.00 |  |  |
|  | June | 33 | 0–0 | 0.00–0.00 | 0–27 | 0.00–44.17 |  |  |
|  | (d) Long-term care beds |  |  |  |  |  |  |  |
|  | January | 132 | 0–1 | 0.00–0.01 | 0–0 | 0.00–0.00 |  |  |
|  | February | 129 | 0–0 | 0.00–0.00 | 0–4 | 0.00–2.77 |  |  |
|  | March | 130 | 0–3 | 0.00–2.17 | 0–0 | 0.00–0.00 |  |  |
|  | April | 133 | 0–4 | 0.00–2.68 | 0–0 | 0.00–0.00 |  |  |
|  | May | 154 | 3–25 | 1.62–19.22 | 0–0 | 0.00–0.00 | * |  |
|  | June | 141 | 0–8 | 0.00–5.28 | 0–0 | 0.00–0.00 |  |  |
|  | (e) General beds |  |  |  |  |  |  |  |
|  | January | 18 | 0–1 | 0.00–4.41 | 0–0 | 0.00–0.00 |  |  |
|  | February | 18 | 0–0 | 0.00–0.00 | 0–1 | 0.00–0.33 |  |  |
|  | March | 17 | 0–0 | 0.00–0.00 | 0–1 | 0.00–1.20 |  |  |
|  | April | 18 | 0–1 | 0.00–3.28 | 0–0 | 0.00–0.00 |  |  |
|  | May | 20 | 0–3 | 0.00–15.18 | 0–0 | 0.00–0.00 |  |  |
|  | June | 18 | 0–1 | 0.00–2.68 | 0–0 | 0.00–0.00 |  |  |
|  | (f) LTCI care beds |  |  |  |  |  |  |  |
|  | January | 325 | 0–0 | 0.00–0.00 | 22–113 | 4.98–25.66 |  | ** |
|  | February | 500 | 63–219 | 22.07–77.73 | 0–0 | 0.00–0.00 | * |  |
|  | March | 371 | 0–0 | 0.00–0.00 | 0–34 | 0.00–8.16 |  |  |
|  | April | 205 | 0–0 | 0.00–0.00 | 117–206 | 28.41–50.09 |  | ** |
|  | May | 448 | 0–36 | 0.00–8.61 | 0–0 | 0.00–0.00 |  |  |
|  | June | 443 | 0–15 | 0.00–3.32 | 0–0 | 0.00–0.00 |  |  |
| Ibaraki | (1) Average number of outpatients per day at hospitals |  |  |  |  |  |  |  |
|  | General hospitals |  |  |  |  |  |  |  |
|  | January | 26424 | 0–0 | 0.00–0.00 | 0–1249 | 0.00–4.51 |  |  |
|  | February | 26663 | 0–0 | 0.00–0.00 | 0–1102 | 0.00–3.97 |  |  |
|  | March | 25546 | 0–0 | 0.00–0.00 | 411–2290 | 1.48–8.23 |  | ** |
|  | April | 22363 | 0–0 | 0.00–0.00 | 2881–4609 | 10.68–17.09 |  | ** |
|  | May | 20165 | 0–0 | 0.00–0.00 | 5228–6944 | 19.29–25.61 |  | ** |
|  | June | 25467 | 0–0 | 0.00–0.00 | 30–1913 | 0.11–6.99 |  | ** |
|  | Psychiatric hospitals |  |  |  |  |  |  |  |
|  | January | 1071 | 0–0 | 0.00–0.00 | 26–103 | 2.22–8.73 |  | ** |
|  | February | 1122 | 0–0 | 0.00–0.00 | 0–67 | 0.00–5.63 |  |  |
|  | March | 1116 | 0–0 | 0.00–0.00 | 29–107 | 2.37–8.68 |  | ** |
|  | April | 1096 | 0–0 | 0.00–0.00 | 23–105 | 1.92–8.67 |  | ** |
|  | May | 966 | 0–0 | 0.00–0.00 | 152–237 | 12.64–19.67 |  | ** |
|  | June | 1122 | 0–0 | 0.00–0.00 | 0–79 | 0.00–6.52 |  |  |
|  | (2) Average number of hospitalized patients per day |  |  |  |  |  |  |  |
|  | (b) Psychiatric care beds |  |  |  |  |  |  |  |
|  | January | 5664 | 0–0 | 0.00–0.00 | 0–14 | 0.00–0.24 |  |  |
|  | February | 5671 | 0–0 | 0.00–0.00 | 0–5 | 0.00–0.07 |  |  |
|  | March | 5653 | 0–0 | 0.00–0.00 | 0–19 | 0.00–0.33 |  |  |
|  | April | 5638 | 0–0 | 0.00–0.00 | 0–26 | 0.00–0.45 |  |  |
|  | May | 5679 | 0–15 | 0.00–0.26 | 0–0 | 0.00–0.00 |  |  |
|  | June | 5751 | 0–72 | 0.00–1.26 | 0–0 | 0.00–0.00 |  |  |
|  | (c) Tuberculosis care beds |  |  |  |  |  |  |  |
|  | January | 19 | 0–0 | 0.00–0.00 | 0–5 | 0.00–20.68 |  |  |
|  | February | 18 | 0–0 | 0.00–0.00 | 0–6 | 0.00–22.21 |  |  |
|  | March | 17 | 0–0 | 0.00–0.00 | 0–6 | 0.00–24.45 |  |  |
|  | April | 25 | 0–2 | 0.00–5.93 | 0–0 | 0.00–0.00 |  |  |
|  | May | 23 | 0–0 | 0.00–0.00 | 0–1 | 0.00–3.15 |  |  |
|  | June | 28 | 0–4 | 0.00–13.99 | 0–0 | 0.00–0.00 |  |  |
|  | (d) Long-term care beds |  |  |  |  |  |  |  |
|  | January | 4511 | 0–0 | 0.00–0.00 | 0–68 | 0.00–1.47 |  |  |
|  | February | 4589 | 0–0 | 0.00–0.00 | 0–11 | 0.00–0.23 |  |  |
|  | March | 4552 | 0–0 | 0.00–0.00 | 0–58 | 0.00–1.26 |  |  |
|  | April | 4459 | 0–0 | 0.00–0.00 | 5–138 | 0.11–2.99 |  | ** |
|  | May | 4477 | 0–0 | 0.00–0.00 | 0–107 | 0.00–2.32 |  |  |
|  | June | 4466 | 0–0 | 0.00–0.00 | 0–115 | 0.00–2.51 |  |  |
|  | (e) General beds |  |  |  |  |  |  |  |
|  | January | 13218 | 0–0 | 0.00–0.00 | 0–317 | 0.00–2.34 |  |  |
|  | February | 13400 | 0–0 | 0.00–0.00 | 0–191 | 0.00–1.40 |  |  |
|  | March | 12806 | 0–0 | 0.00–0.00 | 305–696 | 2.26–5.15 |  | ** |
|  | April | 11676 | 0–0 | 0.00–0.00 | 1088–1557 | 8.22–11.76 |  | ** |
|  | May | 11295 | 0–0 | 0.00–0.00 | 1437–1776 | 10.99–13.58 |  | ** |
|  | June | 11694 | 0–0 | 0.00–0.00 | 957–1318 | 7.35–10.13 |  | ** |
|  | (f) LTCI care beds |  |  |  |  |  |  |  |
|  | January | 270 | 0–20 | 0.00–7.60 | 0–0 | 0.00–0.00 |  |  |
|  | February | 263 | 0–15 | 0.00–5.80 | 0–0 | 0.00–0.00 |  |  |
|  | March | 247 | 0–2 | 0.00–0.50 | 0–0 | 0.00–0.00 |  |  |
|  | April | 237 | 0–0 | 0.00–0.00 | 0–6 | 0.00–2.24 |  |  |
|  | May | 238 | 0–0 | 0.00–0.00 | 0–3 | 0.00–0.88 |  |  |
|  | June | 239 | 0–3 | 0.00–0.94 | 0–0 | 0.00–0.00 |  |  |
|  | (3) Average length of hospital stays per patient |  |  |  |  |  |  |  |
|  | (b) Psychiatric care beds |  |  |  |  |  |  |  |
|  | January | 362 | 0–11 | 0.00–3.06 | 0–0 | 0.00–0.00 |  |  |
|  | February | 340 | 0–0 | 0.00–0.00 | 0–9 | 0.00–2.41 |  |  |
|  | March | 346 | 0–4 | 0.00–1.11 | 0–0 | 0.00–0.00 |  |  |
|  | April | 384 | 5–43 | 1.35–12.31 | 0–0 | 0.00–0.00 | * |  |
|  | May | 407 | 31–68 | 8.86–19.82 | 0–0 | 0.00–0.00 | * |  |
|  | June | 334 | 0–0 | 0.00–0.00 | 0–1 | 0.00–0.11 |  |  |
|  | (c) Tuberculosis care beds |  |  |  |  |  |  |  |
|  | January | 69 | 0–0 | 0.00–0.00 | 0–3 | 0.00–3.99 |  |  |
|  | February | 58 | 0–0 | 0.00–0.00 | 0–10 | 0.00–14.72 |  |  |
|  | March | 60 | 0–0 | 0.00–0.00 | 0–6 | 0.00–8.76 |  |  |
|  | April | 65 | 0–0 | 0.00–0.00 | 0–3 | 0.00–3.89 |  |  |
|  | May | 41 | 0–0 | 0.00–0.00 | 0–26 | 0.00–37.89 |  |  |
|  | June | 60 | 0–0 | 0.00–0.00 | 0–10 | 0.00–13.26 |  |  |
|  | (d) Long-term care beds |  |  |  |  |  |  |  |
|  | January | 125 | 0–7 | 0.00–5.92 | 0–0 | 0.00–0.00 |  |  |
|  | February | 129 | 0–12 | 0.00–9.93 | 0–0 | 0.00–0.00 |  |  |
|  | March | 129 | 0–14 | 0.00–12.17 | 0–0 | 0.00–0.00 |  |  |
|  | April | 132 | 0–14 | 0.00–11.80 | 0–0 | 0.00–0.00 |  |  |
|  | May | 146 | 4–27 | 3.01–21.90 | 0–0 | 0.00–0.00 | * |  |
|  | June | 131 | 0–10 | 0.00–8.05 | 0–0 | 0.00–0.00 |  |  |
|  | (e) General beds |  |  |  |  |  |  |  |
|  | January | 16 | 0–1 | 0.00–2.89 | 0–0 | 0.00–0.00 |  |  |
|  | February | 16 | 0–0 | 0.00–0.00 | 0–1 | 0.00–0.37 |  |  |
|  | March | 16 | 0–1 | 0.00–1.44 | 0–0 | 0.00–0.00 |  |  |
|  | April | 17 | 0–1 | 0.00–5.96 | 0–0 | 0.00–0.00 |  |  |
|  | May | 18 | 0–3 | 0.00–16.08 | 0–0 | 0.00–0.00 |  |  |
|  | June | 16 | 0–1 | 0.00–2.31 | 0–0 | 0.00–0.00 |  |  |
|  | (f) LTCI care beds |  |  |  |  |  |  |  |
|  | January | 279 | 93–137 | 65.05–95.58 | 0–0 | 0.00–0.00 | * |  |
|  | February | 340 | 157–199 | 111.00–140.80 | 0–0 | 0.00–0.00 | * |  |
|  | March | 196 | 14–56 | 9.47–39.74 | 0–0 | 0.00–0.00 | * |  |
|  | April | 216 | 31–75 | 21.61–52.27 | 0–0 | 0.00–0.00 | * |  |
|  | May | 235 | 48–90 | 32.85–61.92 | 0–0 | 0.00–0.00 | * |  |
|  | June | 247 | 48–97 | 31.39–63.84 | 0–0 | 0.00–0.00 | * |  |
| Tochigi | (1) Average number of outpatients per day at hospitals |  |  |  |  |  |  |  |
|  | General hospitals |  |  |  |  |  |  |  |
|  | January | 17649 | 0–0 | 0.00–0.00 | 0–637 | 0.00–3.48 |  |  |
|  | February | 17947 | 0–0 | 0.00–0.00 | 0–498 | 0.00–2.70 |  |  |
|  | March | 17527 | 0–0 | 0.00–0.00 | 0–1216 | 0.00–6.49 |  |  |
|  | April | 15748 | 0–0 | 0.00–0.00 | 1397–2586 | 7.62–14.10 |  | ** |
|  | May | 13828 | 0–0 | 0.00–0.00 | 3456–4790 | 18.56–25.73 |  | ** |
|  | June | 17350 | 0–0 | 0.00–0.00 | 112–1539 | 0.59–8.14 |  | ** |
|  | Psychiatric hospitals |  |  |  |  |  |  |  |
|  | January | 1061 | 0–0 | 0.00–0.00 | 0–30 | 0.00–2.73 |  |  |
|  | February | 1130 | 0–23 | 0.00–1.99 | 0–0 | 0.00–0.00 |  |  |
|  | March | 1101 | 0–0 | 0.00–0.00 | 0–43 | 0.00–3.71 |  |  |
|  | April | 1103 | 0–0 | 0.00–0.00 | 0–19 | 0.00–1.64 |  |  |
|  | May | 979 | 0–0 | 0.00–0.00 | 72–155 | 6.35–13.64 |  | ** |
|  | June | 1104 | 0–0 | 0.00–0.00 | 0–35 | 0.00–3.05 |  |  |
|  | (2) Average number of hospitalized patients per day |  |  |  |  |  |  |  |
|  | (b) Psychiatric care beds |  |  |  |  |  |  |  |
|  | January | 4106 | 0–0 | 0.00–0.00 | 28–155 | 0.66–3.63 |  | ** |
|  | February | 4101 | 0–0 | 0.00–0.00 | 19–146 | 0.45–3.43 |  | ** |
|  | March | 4076 | 0–0 | 0.00–0.00 | 35–162 | 0.83–3.82 |  | ** |
|  | April | 4058 | 0–0 | 0.00–0.00 | 41–169 | 0.97–3.98 |  | ** |
|  | May | 4033 | 0–0 | 0.00–0.00 | 58–186 | 1.38–4.39 |  | ** |
|  | June | 4060 | 0–0 | 0.00–0.00 | 28–155 | 0.66–3.67 |  | ** |
|  | (c) Tuberculosis care beds |  |  |  |  |  |  |  |
|  | January | 19 | 0–0 | 0.00–0.00 | 0–1 | 0.00–2.28 |  |  |
|  | February | 19 | 0–1 | 0.00–4.12 | 0–0 | 0.00–0.00 |  |  |
|  | March | 14 | 0–0 | 0.00–0.00 | 0–5 | 0.00–23.95 |  |  |
|  | April | 13 | 0–0 | 0.00–0.00 | 0–5 | 0.00–27.21 |  |  |
|  | May | 13 | 0–0 | 0.00–0.00 | 0–6 | 0.00–29.27 |  |  |
|  | June | 19 | 0–1 | 0.00–1.03 | 0–0 | 0.00–0.00 |  |  |
|  | (d) Long-term care beds |  |  |  |  |  |  |  |
|  | January | 3501 | 0–0 | 0.00–0.00 | 0–110 | 0.00–3.02 |  |  |
|  | February | 3540 | 0–0 | 0.00–0.00 | 0–86 | 0.00–2.34 |  |  |
|  | March | 3545 | 0–0 | 0.00–0.00 | 0–77 | 0.00–2.11 |  |  |
|  | April | 3494 | 0–0 | 0.00–0.00 | 0–98 | 0.00–2.70 |  |  |
|  | May | 3408 | 0–0 | 0.00–0.00 | 43–160 | 1.21–4.46 |  | ** |
|  | June | 3432 | 0–0 | 0.00–0.00 | 11–127 | 0.31–3.56 |  | ** |
|  | (e) General beds |  |  |  |  |  |  |  |
|  | January | 9235 | 0–0 | 0.00–0.00 | 0–135 | 0.00–1.44 |  |  |
|  | February | 9402 | 0–0 | 0.00–0.00 | 0–43 | 0.00–0.45 |  |  |
|  | March | 8947 | 0–0 | 0.00–0.00 | 182–439 | 1.94–4.68 |  | ** |
|  | April | 8267 | 0–0 | 0.00–0.00 | 631–929 | 6.86–10.09 |  | ** |
|  | May | 7910 | 0–0 | 0.00–0.00 | 990–1184 | 10.89–13.02 |  | ** |
|  | June | 8230 | 0–0 | 0.00–0.00 | 634–873 | 6.97–9.58 |  | ** |
|  | (f) LTCI care beds |  |  |  |  |  |  |  |
|  | January | 349 | 0–0 | 0.00–0.00 | 0–17 | 0.00–4.46 |  |  |
|  | February | 328 | 0–0 | 0.00–0.00 | 0–35 | 0.00–9.53 |  |  |
|  | March | 328 | 0–0 | 0.00–0.00 | 0–32 | 0.00–8.73 |  |  |
|  | April | 327 | 0–0 | 0.00–0.00 | 0–29 | 0.00–8.14 |  |  |
|  | May | 325 | 0–0 | 0.00–0.00 | 0–29 | 0.00–7.95 |  |  |
|  | June | 328 | 0–0 | 0.00–0.00 | 0–23 | 0.00–6.49 |  |  |
|  | (3) Average length of hospital stays per patient |  |  |  |  |  |  |  |
|  | (b) Psychiatric care beds |  |  |  |  |  |  |  |
|  | January | 350 | 0–0 | 0.00–0.00 | 0–2 | 0.00–0.48 |  |  |
|  | February | 333 | 0–0 | 0.00–0.00 | 0–8 | 0.00–2.20 |  |  |
|  | March | 320 | 0–0 | 0.00–0.00 | 0–8 | 0.00–2.35 |  |  |
|  | April | 347 | 0–22 | 0.00–6.70 | 0–0 | 0.00–0.00 |  |  |
|  | May | 383 | 29–65 | 9.09–20.42 | 0–0 | 0.00–0.00 | * |  |
|  | June | 312 | 0–1 | 0.00–0.32 | 0–0 | 0.00–0.00 |  |  |
|  | (c) Tuberculosis care beds |  |  |  |  |  |  |  |
|  | January | 99 | 0–19 | 0.00–22.77 | 0–0 | 0.00–0.00 |  |  |
|  | February | 92 | 0–2 | 0.00–1.94 | 0–0 | 0.00–0.00 |  |  |
|  | March | 72 | 0–0 | 0.00–0.00 | 0–19 | 0.00–20.69 |  |  |
|  | April | 60 | 0–0 | 0.00–0.00 | 0–28 | 0.00–31.45 |  |  |
|  | May | 59 | 0–0 | 0.00–0.00 | 0–23 | 0.00–27.65 |  |  |
|  | June | 76 | 0–0 | 0.00–0.00 | 0–13 | 0.00–13.63 |  |  |
|  | (d) Long-term care beds |  |  |  |  |  |  |  |
|  | January | 153 | 0–9 | 0.00–6.23 | 0–0 | 0.00–0.00 |  |  |
|  | February | 144 | 0–0 | 0.00–0.00 | 0–1 | 0.00–0.07 |  |  |
|  | March | 150 | 0–11 | 0.00–7.16 | 0–0 | 0.00–0.00 |  |  |
|  | April | 149 | 0–5 | 0.00–2.80 | 0–0 | 0.00–0.00 |  |  |
|  | May | 174 | 4–29 | 2.13–19.35 | 0–0 | 0.00–0.00 | * |  |
|  | June | 146 | 0–0 | 0.00–0.00 | 0–3 | 0.00–1.36 |  |  |
|  | (e) General beds |  |  |  |  |  |  |  |
|  | January | 17 | 0–1 | 0.00–3.39 | 0–0 | 0.00–0.00 |  |  |
|  | February | 17 | 0–0 | 0.00–0.00 | 0–1 | 0.00–0.79 |  |  |
|  | March | 16 | 0–0 | 0.00–0.00 | 0–1 | 0.00–0.54 |  |  |
|  | April | 17 | 0–1 | 0.00–2.95 | 0–0 | 0.00–0.00 |  |  |
|  | May | 19 | 0–3 | 0.00–13.38 | 0–0 | 0.00–0.00 |  |  |
|  | June | 16 | 0–0 | 0.00–0.00 | 0–1 | 0.00–0.04 |  |  |
|  | (f) LTCI care beds |  |  |  |  |  |  |  |
|  | January | 285 | 0–0 | 0.00–0.00 | 0–23 | 0.00–7.23 |  |  |
|  | February | 293 | 0–0 | 0.00–0.00 | 0–18 | 0.00–5.58 |  |  |
|  | March | 399 | 0–85 | 0.00–27.02 | 0–0 | 0.00–0.00 |  |  |
|  | April | 356 | 0–37 | 0.00–11.44 | 0–0 | 0.00–0.00 |  |  |
|  | May | 458 | 13–123 | 3.66–36.39 | 0–0 | 0.00–0.00 | * |  |
|  | June | 410 | 0–61 | 0.00–17.32 | 0–0 | 0.00–0.00 |  |  |
| Gunma | (1) Average number of outpatients per day at hospitals |  |  |  |  |  |  |  |
|  | General hospitals |  |  |  |  |  |  |  |
|  | January | 17593 | 0–0 | 0.00–0.00 | 15–1231 | 0.08–6.54 |  | ** |
|  | February | 17729 | 0–0 | 0.00–0.00 | 0–1164 | 0.00–6.16 |  |  |
|  | March | 17227 | 0–0 | 0.00–0.00 | 485–1818 | 2.55–9.54 |  | ** |
|  | April | 15327 | 0–0 | 0.00–0.00 | 1978–3124 | 10.72–16.93 |  | ** |
|  | May | 13470 | 0–0 | 0.00–0.00 | 3908–5063 | 21.09–27.32 |  | ** |
|  | June | 16744 | 0–0 | 0.00–0.00 | 730–2071 | 3.88–11.00 |  | ** |
|  | Psychiatric hospitals |  |  |  |  |  |  |  |
|  | January | 840 | 0–0 | 0.00–0.00 | 0–33 | 0.00–3.77 |  |  |
|  | February | 885 | 0–0 | 0.00–0.00 | 0–4 | 0.00–0.34 |  |  |
|  | March | 888 | 0–0 | 0.00–0.00 | 0–31 | 0.00–3.32 |  |  |
|  | April | 864 | 0–0 | 0.00–0.00 | 0–39 | 0.00–4.31 |  |  |
|  | May | 751 | 0–0 | 0.00–0.00 | 95–158 | 10.45–17.36 |  | ** |
|  | June | 870 | 0–0 | 0.00–0.00 | 0–44 | 0.00–4.72 |  |  |
|  | (2) Average number of hospitalized patients per day |  |  |  |  |  |  |  |
|  | (b) Psychiatric care beds |  |  |  |  |  |  |  |
|  | January | 4505 | 0–28 | 0.00–0.60 | 0–0 | 0.00–0.00 |  |  |
|  | February | 4501 | 0–14 | 0.00–0.31 | 0–0 | 0.00–0.00 |  |  |
|  | March | 4470 | 0–0 | 0.00–0.00 | 0–28 | 0.00–0.62 |  |  |
|  | April | 4427 | 0–0 | 0.00–0.00 | 0–76 | 0.00–1.69 |  |  |
|  | May | 4429 | 0–0 | 0.00–0.00 | 0–86 | 0.00–1.90 |  |  |
|  | June | 4475 | 0–0 | 0.00–0.00 | 0–51 | 0.00–1.12 |  |  |
|  | (c) Tuberculosis care beds |  |  |  |  |  |  |  |
|  | January | 20 | 0–2 | 0.00–9.20 | 0–0 | 0.00–0.00 |  |  |
|  | February | 21 | 0–5 | 0.00–24.03 | 0–0 | 0.00–0.00 |  |  |
|  | March | 22 | 0–7 | 0.00–45.34 | 0–0 | 0.00–0.00 |  |  |
|  | April | 28 | 5–14 | 34.08–90.86 | 0–0 | 0.00–0.00 | * |  |
|  | May | 23 | 0–8 | 0.00–48.26 | 0–0 | 0.00–0.00 |  |  |
|  | June | 23 | 0–6 | 0.00–28.05 | 0–0 | 0.00–0.00 |  |  |
|  | (d) Long-term care beds |  |  |  |  |  |  |  |
|  | January | 3570 | 0–0 | 0.00–0.00 | 28–172 | 0.75–4.58 |  | ** |
|  | February | 3648 | 0–0 | 0.00–0.00 | 0–106 | 0.00–2.80 |  |  |
|  | March | 3632 | 0–0 | 0.00–0.00 | 0–105 | 0.00–2.80 |  |  |
|  | April | 3468 | 0–0 | 0.00–0.00 | 97–235 | 2.62–6.32 |  | ** |
|  | May | 3448 | 0–0 | 0.00–0.00 | 72–208 | 1.97–5.68 |  | ** |
|  | June | 3421 | 0–0 | 0.00–0.00 | 75–202 | 2.07–5.55 |  | ** |
|  | (e) General beds |  |  |  |  |  |  |  |
|  | January | 10881 | 0–0 | 0.00–0.00 | 14–418 | 0.12–3.70 |  | ** |
|  | February | 11109 | 0–0 | 0.00–0.00 | 0–269 | 0.00–2.36 |  |  |
|  | March | 10531 | 0–0 | 0.00–0.00 | 406–760 | 3.60–6.73 |  | ** |
|  | April | 9789 | 0–0 | 0.00–0.00 | 782–1175 | 7.13–10.71 |  | ** |
|  | May | 9380 | 0–0 | 0.00–0.00 | 1156–1432 | 10.69–13.24 |  | ** |
|  | June | 9706 | 0–0 | 0.00–0.00 | 729–1064 | 6.77–9.87 |  | ** |
|  | (f) LTCI care beds |  |  |  |  |  |  |  |
|  | January | 204 | 0–0 | 0.00–0.00 | 0–53 | 0.00–20.38 |  |  |
|  | February | 202 | 0–0 | 0.00–0.00 | 0–48 | 0.00–19.18 |  |  |
|  | March | 206 | 0–0 | 0.00–0.00 | 0–38 | 0.00–15.52 |  |  |
|  | April | 144 | 0–0 | 0.00–0.00 | 33–96 | 13.78–39.86 |  | ** |
|  | May | 140 | 0–0 | 0.00–0.00 | 30–93 | 12.92–39.70 |  | ** |
|  | June | 110 | 0–0 | 0.00–0.00 | 54–117 | 23.87–51.37 |  | ** |
|  | (3) Average length of hospital stays per patient |  |  |  |  |  |  |  |
|  | (b) Psychiatric care beds |  |  |  |  |  |  |  |
|  | January | 311 | 0–0 | 0.00–0.00 | 0–1 | 0.00–0.02 |  |  |
|  | February | 321 | 0–11 | 0.00–3.51 | 0–0 | 0.00–0.00 |  |  |
|  | March | 289 | 0–0 | 0.00–0.00 | 0–4 | 0.00–1.15 |  |  |
|  | April | 349 | 23–59 | 7.78–20.00 | 0–0 | 0.00–0.00 | * |  |
|  | May | 337 | 10–47 | 3.27–15.92 | 0–0 | 0.00–0.00 | * |  |
|  | June | 284 | 0–0 | 0.00–0.00 | 0–5 | 0.00–1.49 |  |  |
|  | (c) Tuberculosis care beds |  |  |  |  |  |  |  |
|  | January | 97 | 0–5 | 0.00–5.17 | 0–0 | 0.00–0.00 |  |  |
|  | February | 73 | 0–0 | 0.00–0.00 | 0–8 | 0.00–9.13 |  |  |
|  | March | 73 | 0–3 | 0.00–4.19 | 0–0 | 0.00–0.00 |  |  |
|  | April | 57 | 0–0 | 0.00–0.00 | 0–15 | 0.00–20.14 |  |  |
|  | May | 60 | 0–0 | 0.00–0.00 | 0–21 | 0.00–25.95 |  |  |
|  | June | 73 | 0–0 | 0.00–0.00 | 0–10 | 0.00–11.34 |  |  |
|  | (d) Long-term care beds |  |  |  |  |  |  |  |
|  | January | 107 | 0–9 | 0.00–9.01 | 0–0 | 0.00–0.00 |  |  |
|  | February | 103 | 0–5 | 0.00–4.51 | 0–0 | 0.00–0.00 |  |  |
|  | March | 110 | 0–13 | 0.00–12.99 | 0–0 | 0.00–0.00 |  |  |
|  | April | 108 | 0–8 | 0.00–6.96 | 0–0 | 0.00–0.00 |  |  |
|  | May | 125 | 2–23 | 1.85–21.86 | 0–0 | 0.00–0.00 | * |  |
|  | June | 110 | 0–6 | 0.00–5.38 | 0–0 | 0.00–0.00 |  |  |
|  | (e) General beds |  |  |  |  |  |  |  |
|  | January | 18 | 0–1 | 0.00–5.48 | 0–0 | 0.00–0.00 |  |  |
|  | February | 17 | 0–1 | 0.00–1.23 | 0–0 | 0.00–0.00 |  |  |
|  | March | 17 | 0–1 | 0.00–0.50 | 0–0 | 0.00–0.00 |  |  |
|  | April | 18 | 0–2 | 0.00–7.19 | 0–0 | 0.00–0.00 |  |  |
|  | May | 20 | 0–4 | 0.00–18.69 | 0–0 | 0.00–0.00 |  |  |
|  | June | 17 | 0–1 | 0.00–3.76 | 0–0 | 0.00–0.00 |  |  |
|  | (f) LTCI care beds |  |  |  |  |  |  |  |
|  | January | 394 | 0–88 | 0.00–28.54 | 0–0 | 0.00–0.00 |  |  |
|  | February | 267 | 0–0 | 0.00–0.00 | 0–45 | 0.00–14.36 |  |  |
|  | March | 297 | 0–0 | 0.00–0.00 | 0–7 | 0.00–2.03 |  |  |
|  | April | 80 | 0–0 | 0.00–0.00 | 115–211 | 39.52–72.45 |  | ** |
|  | May | 543 | 129–249 | 43.55–84.31 | 0–0 | 0.00–0.00 | * |  |
|  | June | 440 | 0–117 | 0.00–35.82 | 0–0 | 0.00–0.00 |  |  |
| Saitama | (1) Average number of outpatients per day at hospitals |  |  |  |  |  |  |  |
|  | General hospitals |  |  |  |  |  |  |  |
|  | January | 57338 | 0–0 | 0.00–0.00 | 0–3184 | 0.00–5.26 |  |  |
|  | February | 58268 | 0–0 | 0.00–0.00 | 0–2484 | 0.00–4.09 |  |  |
|  | March | 54824 | 0–0 | 0.00–0.00 | 2321–6385 | 3.79–10.43 |  | ** |
|  | April | 46999 | 0–0 | 0.00–0.00 | 8599–12434 | 14.47–20.92 |  | ** |
|  | May | 41984 | 0–0 | 0.00–0.00 | 14106–17532 | 23.70–29.46 |  | ** |
|  | June | 52603 | 0–0 | 0.00–0.00 | 3614–7707 | 5.99–12.78 |  | ** |
|  | Psychiatric hospitals |  |  |  |  |  |  |  |
|  | January | 2558 | 0–0 | 0.00–0.00 | 48–254 | 1.71–9.02 |  | ** |
|  | February | 2732 | 0–0 | 0.00–0.00 | 0–111 | 0.00–3.88 |  |  |
|  | March | 2646 | 0–0 | 0.00–0.00 | 68–271 | 2.33–9.28 |  | ** |
|  | April | 2501 | 0–0 | 0.00–0.00 | 151–351 | 5.30–12.28 |  | ** |
|  | May | 2162 | 0–0 | 0.00–0.00 | 469–677 | 16.53–23.82 |  | ** |
|  | June | 2538 | 0–0 | 0.00–0.00 | 101–293 | 3.57–10.35 |  | ** |
|  | (2) Average number of hospitalized patients per day |  |  |  |  |  |  |  |
|  | (b) Psychiatric care beds |  |  |  |  |  |  |  |
|  | January | 12248 | 0–0 | 0.00–0.00 | 0–47 | 0.00–0.38 |  |  |
|  | February | 12223 | 0–0 | 0.00–0.00 | 0–68 | 0.00–0.55 |  |  |
|  | March | 12152 | 0–0 | 0.00–0.00 | 0–132 | 0.00–1.07 |  |  |
|  | April | 12071 | 0–0 | 0.00–0.00 | 0–197 | 0.00–1.60 |  |  |
|  | May | 11956 | 0–0 | 0.00–0.00 | 98–315 | 0.80–2.57 |  | ** |
|  | June | 12052 | 0–0 | 0.00–0.00 | 16–233 | 0.13–1.89 |  | ** |
|  | (c) Tuberculosis care beds |  |  |  |  |  |  |  |
|  | January | 46 | 0–13 | 0.00–37.36 | 0–0 | 0.00–0.00 |  |  |
|  | February | 43 | 0–8 | 0.00–21.08 | 0–0 | 0.00–0.00 |  |  |
|  | March | 36 | 0–0 | 0.00–0.00 | 0–2 | 0.00–5.17 |  |  |
|  | April | 33 | 0–0 | 0.00–0.00 | 0–6 | 0.00–15.23 |  |  |
|  | May | 29 | 0–0 | 0.00–0.00 | 0–9 | 0.00–23.45 |  |  |
|  | June | 35 | 0–0 | 0.00–0.00 | 0–4 | 0.00–8.75 |  |  |
|  | (d) Long-term care beds |  |  |  |  |  |  |  |
|  | January | 10030 | 0–0 | 0.00–0.00 | 0–168 | 0.00–1.65 |  |  |
|  | February | 10107 | 0–0 | 0.00–0.00 | 0–114 | 0.00–1.11 |  |  |
|  | March | 10091 | 0–0 | 0.00–0.00 | 0–164 | 0.00–1.60 |  |  |
|  | April | 9912 | 0–0 | 0.00–0.00 | 115–313 | 1.12–3.05 |  | ** |
|  | May | 9630 | 0–0 | 0.00–0.00 | 344–541 | 3.38–5.32 |  | ** |
|  | June | 9693 | 0–0 | 0.00–0.00 | 224–421 | 2.21–4.16 |  | ** |
|  | (e) General beds |  |  |  |  |  |  |  |
|  | January | 29079 | 0–0 | 0.00–0.00 | 0–244 | 0.00–0.83 |  |  |
|  | February | 29484 | 0–0 | 0.00–0.00 | 0–112 | 0.00–0.38 |  |  |
|  | March | 28167 | 0–0 | 0.00–0.00 | 563–1315 | 1.91–4.46 |  | ** |
|  | April | 25771 | 0–0 | 0.00–0.00 | 2294–3192 | 7.92–11.02 |  | ** |
|  | May | 24531 | 0–0 | 0.00–0.00 | 3519–4202 | 12.25–14.62 |  | ** |
|  | June | 25959 | 0–0 | 0.00–0.00 | 2073–2794 | 7.21–9.72 |  | ** |
|  | (f) LTCI care beds |  |  |  |  |  |  |  |
|  | January | 536 | 0–0 | 0.00–0.00 | 56–115 | 8.61–17.64 |  | ** |
|  | February | 545 | 0–0 | 0.00–0.00 | 41–101 | 6.35–15.55 |  | ** |
|  | March | 543 | 0–0 | 0.00–0.00 | 34–96 | 5.33–14.90 |  | ** |
|  | April | 539 | 0–0 | 0.00–0.00 | 6–78 | 0.97–12.63 |  | ** |
|  | May | 521 | 0–0 | 0.00–0.00 | 0–63 | 0.00–10.69 |  |  |
|  | June | 524 | 0–0 | 0.00–0.00 | 0–22 | 0.00–3.98 |  |  |
|  | (3) Average length of hospital stays per patient |  |  |  |  |  |  |  |
|  | (b) Psychiatric care beds |  |  |  |  |  |  |  |
|  | January | 294 | 0–12 | 0.00–3.99 | 0–0 | 0.00–0.00 |  |  |
|  | February | 274 | 0–0 | 0.00–0.00 | 0–6 | 0.00–1.85 |  |  |
|  | March | 290 | 0–17 | 0.00–6.08 | 0–0 | 0.00–0.00 |  |  |
|  | April | 331 | 22–56 | 7.84–20.07 | 0–0 | 0.00–0.00 | * |  |
|  | May | 351 | 42–76 | 15.18–27.37 | 0–0 | 0.00–0.00 | * |  |
|  | June | 278 | 0–5 | 0.00–1.61 | 0–0 | 0.00–0.00 |  |  |
|  | (c) Tuberculosis care beds |  |  |  |  |  |  |  |
|  | January | 57 | 0–0 | 0.00–0.00 | 0–5 | 0.00–6.66 |  |  |
|  | February | 69 | 0–8 | 0.00–12.47 | 0–0 | 0.00–0.00 |  |  |
|  | March | 51 | 0–0 | 0.00–0.00 | 0–10 | 0.00–15.98 |  |  |
|  | April | 29 | 0–0 | 0.00–0.00 | 19–35 | 29.30–54.09 |  | ** |
|  | May | 33 | 0–0 | 0.00–0.00 | 12–29 | 19.60–46.10 |  | ** |
|  | June | 42 | 0–0 | 0.00–0.00 | 1–17 | 0.86–28.54 |  | ** |
|  | (d) Long-term care beds |  |  |  |  |  |  |  |
|  | January | 154 | 0–6 | 0.00–3.49 | 0–0 | 0.00–0.00 |  |  |
|  | February | 149 | 0–2 | 0.00–1.17 | 0–0 | 0.00–0.00 |  |  |
|  | March | 154 | 0–10 | 0.00–6.51 | 0–0 | 0.00–0.00 |  |  |
|  | April | 160 | 0–12 | 0.00–7.93 | 0–0 | 0.00–0.00 |  |  |
|  | May | 186 | 13–38 | 8.30–24.82 | 0–0 | 0.00–0.00 | * |  |
|  | June | 169 | 0–18 | 0.00–11.80 | 0–0 | 0.00–0.00 |  |  |
|  | (e) General beds |  |  |  |  |  |  |  |
|  | January | 17 | 0–1 | 0.00–4.79 | 0–0 | 0.00–0.00 |  |  |
|  | February | 16 | 0–1 | 0.00–0.44 | 0–0 | 0.00–0.00 |  |  |
|  | March | 16 | 0–1 | 0.00–2.14 | 0–0 | 0.00–0.00 |  |  |
|  | April | 18 | 0–2 | 0.00–8.64 | 0–0 | 0.00–0.00 |  |  |
|  | May | 19 | 0–4 | 0.00–19.27 | 0–0 | 0.00–0.00 |  |  |
|  | June | 17 | 0–1 | 0.00–4.35 | 0–0 | 0.00–0.00 |  |  |
|  | (f) LTCI care beds |  |  |  |  |  |  |  |
|  | January | 320 | 0–15 | 0.00–4.76 | 0–0 | 0.00–0.00 |  |  |
|  | February | 433 | 49–135 | 16.38–45.26 | 0–0 | 0.00–0.00 | * |  |
|  | March | 438 | 56–146 | 19.01–49.65 | 0–0 | 0.00–0.00 | * |  |
|  | April | 394 | 6–104 | 1.83–35.82 | 0–0 | 0.00–0.00 | * |  |
|  | May | 448 | 61–152 | 20.33–50.95 | 0–0 | 0.00–0.00 | * |  |
|  | June | 425 | 0–88 | 0.00–25.78 | 0–0 | 0.00–0.00 |  |  |
| Chiba | (1) Average number of outpatients per day at hospitals |  |  |  |  |  |  |  |
|  | General hospitals |  |  |  |  |  |  |  |
|  | January | 54978 | 0–0 | 0.00–0.00 | 0–3067 | 0.00–5.28 |  |  |
|  | February | 55597 | 0–0 | 0.00–0.00 | 0–2774 | 0.00–4.75 |  |  |
|  | March | 52457 | 0–0 | 0.00–0.00 | 2327–6202 | 3.97–10.57 |  | ** |
|  | April | 45225 | 0–0 | 0.00–0.00 | 7825–11504 | 13.79–20.28 |  | ** |
|  | May | 40275 | 0–0 | 0.00–0.00 | 13108–16724 | 23.00–29.34 |  | ** |
|  | June | 51190 | 0–0 | 0.00–0.00 | 2645–6302 | 4.60–10.96 |  | ** |
|  | Psychiatric hospitals |  |  |  |  |  |  |  |
|  | January | 1544 | 0–0 | 0.00–0.00 | 4–106 | 0.24–6.40 |  | ** |
|  | February | 1648 | 0–0 | 0.00–0.00 | 0–29 | 0.00–1.73 |  |  |
|  | March | 1571 | 0–0 | 0.00–0.00 | 62–159 | 3.58–9.17 |  | ** |
|  | April | 1468 | 0–0 | 0.00–0.00 | 121–225 | 7.15–13.28 |  | ** |
|  | May | 1294 | 0–0 | 0.00–0.00 | 287–399 | 16.96–23.53 |  | ** |
|  | June | 1511 | 0–0 | 0.00–0.00 | 80–180 | 4.73–10.63 |  | ** |
|  | (2) Average number of hospitalized patients per day |  |  |  |  |  |  |  |
|  | (b) Psychiatric care beds |  |  |  |  |  |  |  |
|  | January | 9984 | 0–0 | 0.00–0.00 | 0–84 | 0.00–0.83 |  |  |
|  | February | 9991 | 0–0 | 0.00–0.00 | 0–81 | 0.00–0.80 |  |  |
|  | March | 9950 | 0–0 | 0.00–0.00 | 0–126 | 0.00–1.24 |  |  |
|  | April | 9886 | 0–0 | 0.00–0.00 | 1–198 | 0.01–1.96 |  | ** |
|  | May | 9841 | 0–0 | 0.00–0.00 | 70–267 | 0.69–2.64 |  | ** |
|  | June | 10006 | 0–0 | 0.00–0.00 | 0–134 | 0.00–1.31 |  |  |
|  | (c) Tuberculosis care beds |  |  |  |  |  |  |  |
|  | January | 26 | 0–0 | 0.00–0.00 | 0–10 | 0.00–26.09 |  |  |
|  | February | 30 | 0–0 | 0.00–0.00 | 0–5 | 0.00–13.08 |  |  |
|  | March | 29 | 0–0 | 0.00–0.00 | 0–6 | 0.00–15.38 |  |  |
|  | April | 29 | 0–0 | 0.00–0.00 | 0–6 | 0.00–15.40 |  |  |
|  | May | 27 | 0–0 | 0.00–0.00 | 0–8 | 0.00–22.53 |  |  |
|  | June | 20 | 0–0 | 0.00–0.00 | 4–15 | 11.57–42.17 |  | ** |
|  | (d) Long-term care beds |  |  |  |  |  |  |  |
|  | January | 9146 | 0–0 | 0.00–0.00 | 173–393 | 1.81–4.12 |  | ** |
|  | February | 9347 | 0–0 | 0.00–0.00 | 0–185 | 0.00–1.94 |  |  |
|  | March | 9293 | 0–0 | 0.00–0.00 | 0–244 | 0.00–2.55 |  |  |
|  | April | 9123 | 0–0 | 0.00–0.00 | 144–393 | 1.51–4.12 |  | ** |
|  | May | 8998 | 0–0 | 0.00–0.00 | 259–510 | 2.72–5.36 |  | ** |
|  | June | 9013 | 0–0 | 0.00–0.00 | 224–479 | 2.36–5.04 |  | ** |
|  | (e) General beds |  |  |  |  |  |  |  |
|  | January | 27466 | 0–0 | 0.00–0.00 | 0–506 | 0.00–1.81 |  |  |
|  | February | 27864 | 0–0 | 0.00–0.00 | 0–336 | 0.00–1.19 |  |  |
|  | March | 26566 | 0–0 | 0.00–0.00 | 759–1602 | 2.69–5.69 |  | ** |
|  | April | 24594 | 0–0 | 0.00–0.00 | 2047–2961 | 7.43–10.74 |  | ** |
|  | May | 23266 | 0–0 | 0.00–0.00 | 3469–4069 | 12.69–14.89 |  | ** |
|  | June | 24741 | 0–0 | 0.00–0.00 | 1935–2687 | 7.05–9.80 |  | ** |
|  | (f) LTCI care beds |  |  |  |  |  |  |  |
|  | January | 560 | 0–0 | 0.00–0.00 | 37–148 | 5.23–20.86 |  | ** |
|  | February | 558 | 0–0 | 0.00–0.00 | 0–105 | 0.00–15.79 |  |  |
|  | March | 552 | 0–0 | 0.00–0.00 | 0–64 | 0.00–10.36 |  |  |
|  | April | 414 | 0–0 | 0.00–0.00 | 67–192 | 11.07–31.61 |  | ** |
|  | May | 414 | 0–0 | 0.00–0.00 | 37–168 | 6.37–28.78 |  | ** |
|  | June | 417 | 0–0 | 0.00–0.00 | 22–152 | 3.87–26.67 |  | ** |
|  | (3) Average length of hospital stays per patient |  |  |  |  |  |  |  |
|  | (b) Psychiatric care beds |  |  |  |  |  |  |  |
|  | January | 320 | 0–13 | 0.00–4.22 | 0–0 | 0.00–0.00 |  |  |
|  | February | 312 | 0–13 | 0.00–4.23 | 0–0 | 0.00–0.00 |  |  |
|  | March | 293 | 0–4 | 0.00–1.27 | 0–0 | 0.00–0.00 |  |  |
|  | April | 364 | 38–73 | 12.77–24.75 | 0–0 | 0.00–0.00 | * |  |
|  | May | 382 | 57–91 | 19.29–30.98 | 0–0 | 0.00–0.00 | * |  |
|  | June | 309 | 0–19 | 0.00–6.22 | 0–0 | 0.00–0.00 |  |  |
|  | (c) Tuberculosis care beds |  |  |  |  |  |  |  |
|  | January | 57 | 0–0 | 0.00–0.00 | 0–5 | 0.00–6.91 |  |  |
|  | February | 66 | 0–4 | 0.00–6.29 | 0–0 | 0.00–0.00 |  |  |
|  | March | 91 | 13–31 | 20.20–49.97 | 0–0 | 0.00–0.00 | * |  |
|  | April | 49 | 0–0 | 0.00–0.00 | 1–17 | 1.54–24.71 |  | ** |
|  | May | 77 | 0–11 | 0.00–15.56 | 0–0 | 0.00–0.00 |  |  |
|  | June | 57 | 0–0 | 0.00–0.00 | 0–11 | 0.00–15.41 |  |  |
|  | (d) Long-term care beds |  |  |  |  |  |  |  |
|  | January | 160 | 0–6 | 0.00–3.87 | 0–0 | 0.00–0.00 |  |  |
|  | February | 151 | 0–0 | 0.00–0.00 | 0–3 | 0.00–1.94 |  |  |
|  | March | 150 | 0–1 | 0.00–0.13 | 0–0 | 0.00–0.00 |  |  |
|  | April | 149 | 0–0 | 0.00–0.00 | 0–6 | 0.00–3.71 |  |  |
|  | May | 179 | 0–24 | 0.00–15.10 | 0–0 | 0.00–0.00 |  |  |
|  | June | 157 | 0–0 | 0.00–0.00 | 0–2 | 0.00–1.11 |  |  |
|  | (e) General beds |  |  |  |  |  |  |  |
|  | January | 16 | 0–1 | 0.00–5.10 | 0–0 | 0.00–0.00 |  |  |
|  | February | 16 | 0–1 | 0.00–1.10 | 0–0 | 0.00–0.00 |  |  |
|  | March | 16 | 0–1 | 0.00–1.75 | 0–0 | 0.00–0.00 |  |  |
|  | April | 17 | 0–2 | 0.00–8.79 | 0–0 | 0.00–0.00 |  |  |
|  | May | 18 | 0–4 | 0.00–20.10 | 0–0 | 0.00–0.00 |  |  |
|  | June | 16 | 0–1 | 0.00–3.98 | 0–0 | 0.00–0.00 |  |  |
|  | (f) LTCI care beds |  |  |  |  |  |  |  |
|  | January | 235 | 0–0 | 0.00–0.00 | 0–53 | 0.00–18.36 |  |  |
|  | February | 240 | 0–0 | 0.00–0.00 | 0–32 | 0.00–11.57 |  |  |
|  | March | 245 | 0–2 | 0.00–0.55 | 0–0 | 0.00–0.00 |  |  |
|  | April | 123 | 0–0 | 0.00–0.00 | 72–134 | 28.01–52.04 |  | ** |
|  | May | 467 | 147–221 | 59.67–89.57 | 0–0 | 0.00–0.00 | * |  |
|  | June | 321 | 3–72 | 1.08–28.50 | 0–0 | 0.00–0.00 | * |  |
| Tokyo | (1) Average number of outpatients per day at hospitals |  |  |  |  |  |  |  |
|  | General hospitals |  |  |  |  |  |  |  |
|  | January | 128313 | 0–0 | 0.00–0.00 | 0–6789 | 0.00–5.02 |  |  |
|  | February | 128842 | 0–0 | 0.00–0.00 | 0–7649 | 0.00–5.60 |  |  |
|  | March | 122829 | 0–0 | 0.00–0.00 | 5572–15041 | 4.04–10.91 |  | ** |
|  | April | 94560 | 0–0 | 0.00–0.00 | 29751–39102 | 22.26–29.25 |  | ** |
|  | May | 84047 | 0–0 | 0.00–0.00 | 41085–50226 | 30.60–37.41 |  | ** |
|  | June | 114770 | 0–0 | 0.00–0.00 | 10813–20254 | 8.01–15.00 |  | ** |
|  | Psychiatric hospitals |  |  |  |  |  |  |  |
|  | January | 2536 | 0–0 | 0.00–0.00 | 0–163 | 0.00–6.02 |  |  |
|  | February | 2663 | 0–0 | 0.00–0.00 | 0–84 | 0.00–3.04 |  |  |
|  | March | 2585 | 0–0 | 0.00–0.00 | 55–232 | 1.95–8.20 |  | ** |
|  | April | 2415 | 0–0 | 0.00–0.00 | 171–351 | 6.18–12.68 |  | ** |
|  | May | 2155 | 0–0 | 0.00–0.00 | 396–602 | 14.36–21.83 |  | ** |
|  | June | 2414 | 0–0 | 0.00–0.00 | 160–332 | 5.83–12.09 |  | ** |
|  | (2) Average number of hospitalized patients per day |  |  |  |  |  |  |  |
|  | (b) Psychiatric care beds |  |  |  |  |  |  |  |
|  | January | 19023 | 0–50 | 0.00–0.26 | 0–0 | 0.00–0.00 |  |  |
|  | February | 19023 | 0–58 | 0.00–0.30 | 0–0 | 0.00–0.00 |  |  |
|  | March | 18904 | 0–0 | 0.00–0.00 | 0–67 | 0.00–0.35 |  |  |
|  | April | 18547 | 0–0 | 0.00–0.00 | 162–431 | 0.85–2.27 |  | ** |
|  | May | 18404 | 0–0 | 0.00–0.00 | 335–605 | 1.76–3.18 |  | ** |
|  | June | 18430 | 0–0 | 0.00–0.00 | 357–626 | 1.87–3.28 |  | ** |
|  | (c) Tuberculosis care beds |  |  |  |  |  |  |  |
|  | January | 205 | 0–0 | 0.00–0.00 | 0–5 | 0.00–2.09 |  |  |
|  | February | 194 | 0–0 | 0.00–0.00 | 0–12 | 0.00–5.62 |  |  |
|  | March | 203 | 0–0 | 0.00–0.00 | 0–3 | 0.00–1.38 |  |  |
|  | April | 202 | 0–0 | 0.00–0.00 | 0–6 | 0.00–2.73 |  |  |
|  | May | 198 | 0–0 | 0.00–0.00 | 0–13 | 0.00–5.90 |  |  |
|  | June | 198 | 0–0 | 0.00–0.00 | 0–16 | 0.00–7.06 |  |  |
|  | (d) Long-term care beds |  |  |  |  |  |  |  |
|  | January | 21080 | 0–0 | 0.00–0.00 | 313–620 | 1.44–2.85 |  | ** |
|  | February | 21272 | 0–0 | 0.00–0.00 | 0–121 | 0.00–0.56 |  |  |
|  | March | 21194 | 0–0 | 0.00–0.00 | 0–272 | 0.00–1.26 |  |  |
|  | April | 20262 | 0–0 | 0.00–0.00 | 783–1169 | 3.65–5.45 |  | ** |
|  | May | 19788 | 0–0 | 0.00–0.00 | 1619–1907 | 7.46–8.79 |  | ** |
|  | June | 19843 | 0–0 | 0.00–0.00 | 1504–1792 | 6.95–8.28 |  | ** |
|  | (e) General beds |  |  |  |  |  |  |  |
|  | January | 61176 | 0–0 | 0.00–0.00 | 0–1034 | 0.00–1.66 |  |  |
|  | February | 62886 | 0–0 | 0.00–0.00 | 0–117 | 0.00–0.19 |  |  |
|  | March | 59812 | 0–0 | 0.00–0.00 | 1628–3365 | 2.58–5.33 |  | ** |
|  | April | 52278 | 0–0 | 0.00–0.00 | 7318–9472 | 11.85–15.34 |  | ** |
|  | May | 47849 | 0–0 | 0.00–0.00 | 11548–13257 | 18.90–21.70 |  | ** |
|  | June | 51718 | 0–0 | 0.00–0.00 | 7725–9384 | 12.64–15.36 |  | ** |
|  | (f) LTCI care beds |  |  |  |  |  |  |  |
|  | January | 2874 | 0–0 | 0.00–0.00 | 174–388 | 5.33–11.89 |  | ** |
|  | February | 2881 | 0–0 | 0.00–0.00 | 133–352 | 4.11–10.89 |  | ** |
|  | March | 2670 | 0–0 | 0.00–0.00 | 321–534 | 10.02–16.66 |  | ** |
|  | April | 1742 | 0–0 | 0.00–0.00 | 1213–1421 | 38.36–44.91 |  | ** |
|  | May | 1720 | 0–0 | 0.00–0.00 | 1181–1388 | 38.00–44.65 |  | ** |
|  | June | 1723 | 0–0 | 0.00–0.00 | 1139–1340 | 37.19–43.74 |  | ** |
|  | (3) Average length of hospital stays per patient |  |  |  |  |  |  |  |
|  | (b) Psychiatric care beds |  |  |  |  |  |  |  |
|  | January | 200 | 0–7 | 0.00–3.37 | 0–0 | 0.00–0.00 |  |  |
|  | February | 154 | 0–0 | 0.00–0.00 | 10–36 | 5.03–18.76 |  | ** |
|  | March | 188 | 0–6 | 0.00–2.90 | 0–0 | 0.00–0.00 |  |  |
|  | April | 218 | 5–33 | 2.69–17.47 | 0–0 | 0.00–0.00 | * |  |
|  | May | 248 | 36–63 | 19.26–33.98 | 0–0 | 0.00–0.00 | * |  |
|  | June | 193 | 0–10 | 0.00–5.36 | 0–0 | 0.00–0.00 |  |  |
|  | (c) Tuberculosis care beds |  |  |  |  |  |  |  |
|  | January | 59 | 0–5 | 0.00–8.91 | 0–0 | 0.00–0.00 |  |  |
|  | February | 36 | 0–0 | 0.00–0.00 | 5–18 | 7.81–33.39 |  | ** |
|  | March | 36 | 0–0 | 0.00–0.00 | 3–16 | 4.69–30.48 |  | ** |
|  | April | 29 | 0–0 | 0.00–0.00 | 10–23 | 18.61–43.78 |  | ** |
|  | May | 31 | 0–0 | 0.00–0.00 | 9–22 | 15.76–41.72 |  | ** |
|  | June | 28 | 0–0 | 0.00–0.00 | 13–26 | 22.99–48.22 |  | ** |
|  | (d) Long-term care beds |  |  |  |  |  |  |  |
|  | January | 149 | 0–20 | 0.00–15.30 | 0–0 | 0.00–0.00 |  |  |
|  | February | 135 | 0–7 | 0.00–4.79 | 0–0 | 0.00–0.00 |  |  |
|  | March | 139 | 0–13 | 0.00–9.77 | 0–0 | 0.00–0.00 |  |  |
|  | April | 142 | 0–12 | 0.00–8.90 | 0–0 | 0.00–0.00 |  |  |
|  | May | 162 | 8–31 | 5.81–23.62 | 0–0 | 0.00–0.00 | * |  |
|  | June | 139 | 0–7 | 0.00–4.62 | 0–0 | 0.00–0.00 |  |  |
|  | (e) General beds |  |  |  |  |  |  |  |
|  | January | 14 | 0–1 | 0.00–3.93 | 0–0 | 0.00–0.00 |  |  |
|  | February | 14 | 0–1 | 0.00–1.18 | 0–0 | 0.00–0.00 |  |  |
|  | March | 14 | 0–1 | 0.00–2.23 | 0–0 | 0.00–0.00 |  |  |
|  | April | 16 | 0–3 | 0.00–16.54 | 0–0 | 0.00–0.00 |  |  |
|  | May | 17 | 0–4 | 0.00–26.91 | 0–0 | 0.00–0.00 |  |  |
|  | June | 14 | 0–1 | 0.00–4.80 | 0–0 | 0.00–0.00 |  |  |
|  | (f) LTCI care beds |  |  |  |  |  |  |  |
|  | January | 410 | 0–56 | 0.00–15.71 | 0–0 | 0.00–0.00 |  |  |
|  | February | 438 | 19–77 | 5.13–21.23 | 0–0 | 0.00–0.00 | * |  |
|  | March | 432 | 10–70 | 2.54–19.28 | 0–0 | 0.00–0.00 | * |  |
|  | April | 203 | 0–0 | 0.00–0.00 | 113–165 | 30.65–44.75 |  | ** |
|  | May | 627 | 211–269 | 58.53–74.59 | 0–0 | 0.00–0.00 | * |  |
|  | June | 373 | 0–5 | 0.00–1.32 | 0–0 | 0.00–0.00 |  |  |
| Kanagawa | (1) Average number of outpatients per day at hospitals |  |  |  |  |  |  |  |
|  | General hospitals |  |  |  |  |  |  |  |
|  | January | 68022 | 0–0 | 0.00–0.00 | 0–3819 | 0.00–5.32 |  |  |
|  | February | 68414 | 0–0 | 0.00–0.00 | 0–4303 | 0.00–5.92 |  |  |
|  | March | 65373 | 0–0 | 0.00–0.00 | 2944–8002 | 4.01–10.90 |  | ** |
|  | April | 54755 | 0–0 | 0.00–0.00 | 11304–16309 | 15.91–22.95 |  | ** |
|  | May | 48126 | 0–0 | 0.00–0.00 | 18290–23240 | 25.63–32.56 |  | ** |
|  | June | 62958 | 0–0 | 0.00–0.00 | 3891–8946 | 5.41–12.44 |  | ** |
|  | Psychiatric hospitals |  |  |  |  |  |  |  |
|  | January | 2278 | 0–0 | 0.00–0.00 | 0–46 | 0.00–1.95 |  |  |
|  | February | 2408 | 0–37 | 0.00–1.52 | 0–0 | 0.00–0.00 |  |  |
|  | March | 2330 | 0–0 | 0.00–0.00 | 0–115 | 0.00–4.70 |  |  |
|  | April | 2143 | 0–0 | 0.00–0.00 | 88–247 | 3.68–10.32 |  | ** |
|  | May | 1879 | 0–0 | 0.00–0.00 | 338–509 | 14.16–21.29 |  | ** |
|  | June | 2264 | 0–0 | 0.00–0.00 | 0–121 | 0.00–5.06 |  |  |
|  | (2) Average number of hospitalized patients per day |  |  |  |  |  |  |  |
|  | (b) Psychiatric care beds |  |  |  |  |  |  |  |
|  | January | 11577 | 0–0 | 0.00–0.00 | 37–249 | 0.31–2.11 |  | ** |
|  | February | 11536 | 0–0 | 0.00–0.00 | 69–282 | 0.58–2.38 |  | ** |
|  | March | 11437 | 0–0 | 0.00–0.00 | 156–368 | 1.32–3.11 |  | ** |
|  | April | 11360 | 0–0 | 0.00–0.00 | 209–421 | 1.77–3.57 |  | ** |
|  | May | 11258 | 0–0 | 0.00–0.00 | 316–528 | 2.68–4.48 |  | ** |
|  | June | 11413 | 0–0 | 0.00–0.00 | 174–387 | 1.47–3.27 |  | ** |
|  | (c) Tuberculosis care beds |  |  |  |  |  |  |  |
|  | January | 59 | 0–0 | 0.00–0.00 | 0–13 | 0.00–17.40 |  |  |
|  | February | 63 | 0–0 | 0.00–0.00 | 0–10 | 0.00–12.86 |  |  |
|  | March | 72 | 0–0 | 0.00–0.00 | 0–1 | 0.00–1.24 |  |  |
|  | April | 84 | 0–12 | 0.00–15.38 | 0–0 | 0.00–0.00 |  |  |
|  | May | 66 | 0–0 | 0.00–0.00 | 0–5 | 0.00–6.45 |  |  |
|  | June | 51 | 0–0 | 0.00–0.00 | 3–19 | 4.34–26.30 |  | ** |
|  | (d) Long-term care beds |  |  |  |  |  |  |  |
|  | January | 11710 | 0–104 | 0.00–0.89 | 0–0 | 0.00–0.00 |  |  |
|  | February | 11829 | 13–226 | 0.11–1.95 | 0–0 | 0.00–0.00 | * |  |
|  | March | 11798 | 3–215 | 0.03–1.86 | 0–0 | 0.00–0.00 | * |  |
|  | April | 11827 | 50–263 | 0.43–2.27 | 0–0 | 0.00–0.00 | * |  |
|  | May | 11558 | 0–4 | 0.00–0.03 | 0–0 | 0.00–0.00 |  |  |
|  | June | 11573 | 0–30 | 0.00–0.26 | 0–0 | 0.00–0.00 |  |  |
|  | (e) General beds |  |  |  |  |  |  |  |
|  | January | 35962 | 0–0 | 0.00–0.00 | 61–1318 | 0.16–3.53 |  | ** |
|  | February | 37001 | 0–0 | 0.00–0.00 | 0–647 | 0.00–1.72 |  |  |
|  | March | 34990 | 0–0 | 0.00–0.00 | 1491–2608 | 3.97–6.94 |  | ** |
|  | April | 31771 | 0–0 | 0.00–0.00 | 3748–4966 | 10.20–13.52 |  | ** |
|  | May | 29863 | 0–0 | 0.00–0.00 | 5716–6595 | 15.68–18.09 |  | ** |
|  | June | 31493 | 0–0 | 0.00–0.00 | 4054–5063 | 11.09–13.85 |  | ** |
|  | (f) LTCI care beds |  |  |  |  |  |  |  |
|  | January | 884 | 0–0 | 0.00–0.00 | 0–66 | 0.00–6.91 |  |  |
|  | February | 886 | 0–0 | 0.00–0.00 | 0–39 | 0.00–4.19 |  |  |
|  | March | 881 | 0–0 | 0.00–0.00 | 0–13 | 0.00–1.44 |  |  |
|  | April | 868 | 0–1 | 0.00–0.11 | 0–0 | 0.00–0.00 |  |  |
|  | May | 873 | 0–28 | 0.00–3.20 | 0–0 | 0.00–0.00 |  |  |
|  | June | 873 | 0–41 | 0.00–4.90 | 0–0 | 0.00–0.00 |  |  |
|  | (3) Average length of hospital stays per patient |  |  |  |  |  |  |  |
|  | (b) Psychiatric care beds |  |  |  |  |  |  |  |
|  | January | 248 | 0–9 | 0.00–3.44 | 0–0 | 0.00–0.00 |  |  |
|  | February | 234 | 0–0 | 0.00–0.00 | 0–1 | 0.00–0.07 |  |  |
|  | March | 238 | 0–11 | 0.00–4.60 | 0–0 | 0.00–0.00 |  |  |
|  | April | 257 | 0–30 | 0.00–12.78 | 0–0 | 0.00–0.00 |  |  |
|  | May | 294 | 37–68 | 16.19–29.85 | 0–0 | 0.00–0.00 | * |  |
|  | June | 237 | 0–12 | 0.00–5.13 | 0–0 | 0.00–0.00 |  |  |
|  | (c) Tuberculosis care beds |  |  |  |  |  |  |  |
|  | January | 58 | 0–0 | 0.00–0.00 | 0–5 | 0.00–7.68 |  |  |
|  | February | 45 | 0–0 | 0.00–0.00 | 6–21 | 8.35–31.05 |  | ** |
|  | March | 53 | 0–0 | 0.00–0.00 | 0–11 | 0.00–16.79 |  |  |
|  | April | 22 | 0–0 | 0.00–0.00 | 26–41 | 41.65–64.76 |  | ** |
|  | May | 40 | 0–0 | 0.00–0.00 | 7–21 | 10.79–34.42 |  | ** |
|  | June | 30 | 0–0 | 0.00–0.00 | 16–31 | 25.55–50.20 |  | ** |
|  | (d) Long-term care beds |  |  |  |  |  |  |  |
|  | January | 173 | 0–18 | 0.00–11.18 | 0–0 | 0.00–0.00 |  |  |
|  | February | 160 | 0–6 | 0.00–3.24 | 0–0 | 0.00–0.00 |  |  |
|  | March | 166 | 0–17 | 0.00–10.89 | 0–0 | 0.00–0.00 |  |  |
|  | April | 174 | 0–20 | 0.00–12.70 | 0–0 | 0.00–0.00 |  |  |
|  | May | 187 | 7–32 | 4.05–20.39 | 0–0 | 0.00–0.00 | * |  |
|  | June | 165 | 0–6 | 0.00–3.63 | 0–0 | 0.00–0.00 |  |  |
|  | (e) General beds |  |  |  |  |  |  |  |
|  | January | 15 | 0–1 | 0.00–3.97 | 0–0 | 0.00–0.00 |  |  |
|  | February | 14 | 0–1 | 0.00–0.76 | 0–0 | 0.00–0.00 |  |  |
|  | March | 14 | 0–1 | 0.00–1.18 | 0–0 | 0.00–0.00 |  |  |
|  | April | 15 | 0–2 | 0.00–7.73 | 0–0 | 0.00–0.00 |  |  |
|  | May | 17 | 0–3 | 0.00–21.64 | 0–0 | 0.00–0.00 |  |  |
|  | June | 14 | 0–1 | 0.00–4.18 | 0–0 | 0.00–0.00 |  |  |
|  | (f) LTCI care beds |  |  |  |  |  |  |  |
|  | January | 559 | 0–106 | 0.00–23.34 | 0–0 | 0.00–0.00 |  |  |
|  | February | 345 | 0–0 | 0.00–0.00 | 5–102 | 0.94–22.78 |  | ** |
|  | March | 459 | 0–16 | 0.00–3.57 | 0–0 | 0.00–0.00 |  |  |
|  | April | 573 | 0–90 | 0.00–18.53 | 0–0 | 0.00–0.00 |  |  |
|  | May | 576 | 0–57 | 0.00–10.82 | 0–0 | 0.00–0.00 |  |  |
|  | June | 563 | 0–32 | 0.00–5.95 | 0–0 | 0.00–0.00 |  |  |
| Niigata | (1) Average number of outpatients per day at hospitals |  |  |  |  |  |  |  |
|  | General hospitals |  |  |  |  |  |  |  |
|  | January | 21208 | 0–0 | 0.00–0.00 | 0–948 | 0.00–4.28 |  |  |
|  | February | 20900 | 0–0 | 0.00–0.00 | 0–1522 | 0.00–6.79 |  |  |
|  | March | 21817 | 0–0 | 0.00–0.00 | 0–1102 | 0.00–4.81 |  |  |
|  | April | 20408 | 0–0 | 0.00–0.00 | 465–2157 | 2.06–9.56 |  | ** |
|  | May | 17432 | 0–0 | 0.00–0.00 | 3419–5169 | 15.13–22.87 |  | ** |
|  | June | 21568 | 0–0 | 0.00–0.00 | 0–1260 | 0.00–5.52 |  |  |
|  | Psychiatric hospitals |  |  |  |  |  |  |  |
|  | January | 1000 | 0–0 | 0.00–0.00 | 1–94 | 0.09–8.58 |  | ** |
|  | February | 1098 | 0–0 | 0.00–0.00 | 0–7 | 0.00–0.59 |  |  |
|  | March | 1140 | 0–6 | 0.00–0.46 | 0–0 | 0.00–0.00 |  |  |
|  | April | 1136 | 0–24 | 0.00–2.13 | 0–0 | 0.00–0.00 |  |  |
|  | May | 971 | 0–0 | 0.00–0.00 | 41–139 | 3.70–12.45 |  | ** |
|  | June | 1145 | 0–38 | 0.00–3.40 | 0–0 | 0.00–0.00 |  |  |
|  | (2) Average number of hospitalized patients per day |  |  |  |  |  |  |  |
|  | (b) Psychiatric care beds |  |  |  |  |  |  |  |
|  | January | 5306 | 0–0 | 0.00–0.00 | 0–60 | 0.00–1.10 |  |  |
|  | February | 5304 | 0–0 | 0.00–0.00 | 0–56 | 0.00–1.04 |  |  |
|  | March | 5249 | 0–0 | 0.00–0.00 | 0–93 | 0.00–1.73 |  |  |
|  | April | 5190 | 0–0 | 0.00–0.00 | 0–124 | 0.00–2.32 |  |  |
|  | May | 5216 | 0–0 | 0.00–0.00 | 0–77 | 0.00–1.45 |  |  |
|  | June | 5279 | 0–0 | 0.00–0.00 | 0–8 | 0.00–0.14 |  |  |
|  | (c) Tuberculosis care beds |  |  |  |  |  |  |  |
|  | January | 15 | 0–4 | 0.00–28.46 | 0–0 | 0.00–0.00 |  |  |
|  | February | 15 | 0–5 | 0.00–47.17 | 0–0 | 0.00–0.00 |  |  |
|  | March | 10 | 0–1 | 0.00–4.92 | 0–0 | 0.00–0.00 |  |  |
|  | April | 7 | 0–0 | 0.00–0.00 | 0–3 | 0.00–24.95 |  |  |
|  | May | 2 | 0–0 | 0.00–0.00 | 2–8 | 21.98–78.02 |  | ** |
|  | June | 9 | 0–0 | 0.00–0.00 | 0–1 | 0.00–3.68 |  |  |
|  | (d) Long-term care beds |  |  |  |  |  |  |  |
|  | January | 3816 | 0–0 | 0.00–0.00 | 283–410 | 6.70–9.68 |  | ** |
|  | February | 3808 | 0–0 | 0.00–0.00 | 295–422 | 6.97–9.97 |  | ** |
|  | March | 3680 | 0–0 | 0.00–0.00 | 416–543 | 9.85–12.85 |  | ** |
|  | April | 3283 | 0–0 | 0.00–0.00 | 792–918 | 18.85–21.85 |  | ** |
|  | May | 3239 | 0–0 | 0.00–0.00 | 819–945 | 19.57–22.59 |  | ** |
|  | June | 3213 | 0–0 | 0.00–0.00 | 837–964 | 20.04–23.06 |  | ** |
|  | (e) General beds |  |  |  |  |  |  |  |
|  | January | 12604 | 0–0 | 0.00–0.00 | 0–268 | 0.00–2.08 |  |  |
|  | February | 12985 | 0–0 | 0.00–0.00 | 0–16 | 0.00–0.12 |  |  |
|  | March | 12406 | 0–0 | 0.00–0.00 | 149–506 | 1.15–3.92 |  | ** |
|  | April | 11596 | 0–0 | 0.00–0.00 | 643–1055 | 5.08–8.34 |  | ** |
|  | May | 11065 | 0–0 | 0.00–0.00 | 1139–1415 | 9.13–11.33 |  | ** |
|  | June | 11505 | 0–0 | 0.00–0.00 | 646–969 | 5.18–7.76 |  | ** |
|  | (f) LTCI care beds |  |  |  |  |  |  |  |
|  | January | 1026 | 0–0 | 0.00–0.00 | 251–323 | 18.61–23.93 |  | ** |
|  | February | 975 | 0–0 | 0.00–0.00 | 304–376 | 22.51–27.80 |  | ** |
|  | March | 875 | 0–0 | 0.00–0.00 | 402–474 | 29.81–35.12 |  | ** |
|  | April | 521 | 0–0 | 0.00–0.00 | 751–823 | 55.90–61.22 |  | ** |
|  | May | 511 | 0–0 | 0.00–0.00 | 754–826 | 56.43–61.76 |  | ** |
|  | June | 505 | 0–0 | 0.00–0.00 | 755–827 | 56.72–62.06 |  | ** |
|  | (3) Average length of hospital stays per patient |  |  |  |  |  |  |  |
|  | (b) Psychiatric care beds |  |  |  |  |  |  |  |
|  | January | 324 | 0–1 | 0.00–0.22 | 0–0 | 0.00–0.00 |  |  |
|  | February | 329 | 0–6 | 0.00–1.64 | 0–0 | 0.00–0.00 |  |  |
|  | March | 309 | 0–0 | 0.00–0.00 | 0–7 | 0.00–2.10 |  |  |
|  | April | 333 | 0–25 | 0.00–8.04 | 0–0 | 0.00–0.00 |  |  |
|  | May | 362 | 26–61 | 8.34–19.92 | 0–0 | 0.00–0.00 | * |  |
|  | June | 286 | 0–0 | 0.00–0.00 | 0–13 | 0.00–4.10 |  |  |
|  | (c) Tuberculosis care beds |  |  |  |  |  |  |  |
|  | January | 79 | 0–18 | 0.00–29.09 | 0–0 | 0.00–0.00 |  |  |
|  | February | 53 | 0–0 | 0.00–0.00 | 0–14 | 0.00–20.92 |  |  |
|  | March | 69 | 0–6 | 0.00–9.41 | 0–0 | 0.00–0.00 |  |  |
|  | April | 55 | 0–0 | 0.00–0.00 | 0–12 | 0.00–17.96 |  |  |
|  | May | 22 | 0–0 | 0.00–0.00 | 18–45 | 26.03–67.35 |  | ** |
|  | June | 138 | 33–76 | 52.39–119.10 | 0–0 | 0.00–0.00 | * |  |
|  | (d) Long-term care beds |  |  |  |  |  |  |  |
|  | January | 154 | 0–0 | 0.00–0.00 | 0–10 | 0.00–5.97 |  |  |
|  | February | 162 | 0–0 | 0.00–0.00 | 0–2 | 0.00–1.07 |  |  |
|  | March | 141 | 0–0 | 0.00–0.00 | 0–17 | 0.00–10.72 |  |  |
|  | April | 149 | 0–0 | 0.00–0.00 | 0–11 | 0.00–6.63 |  |  |
|  | May | 163 | 0–3 | 0.00–1.88 | 0–0 | 0.00–0.00 |  |  |
|  | June | 145 | 0–0 | 0.00–0.00 | 0–20 | 0.00–11.81 |  |  |
|  | (e) General beds |  |  |  |  |  |  |  |
|  | January | 19 | 0–1 | 0.00–2.95 | 0–0 | 0.00–0.00 |  |  |
|  | February | 19 | 0–1 | 0.00–0.92 | 0–0 | 0.00–0.00 |  |  |
|  | March | 19 | 0–1 | 0.00–0.56 | 0–0 | 0.00–0.00 |  |  |
|  | April | 19 | 0–1 | 0.00–0.56 | 0–0 | 0.00–0.00 |  |  |
|  | May | 20 | 0–2 | 0.00–9.89 | 0–0 | 0.00–0.00 |  |  |
|  | June | 18 | 0–1 | 0.00–0.29 | 0–0 | 0.00–0.00 |  |  |
|  | (f) LTCI care beds |  |  |  |  |  |  |  |
|  | January | 187 | 0–0 | 0.00–0.00 | 110–192 | 29.00–50.64 |  | ** |
|  | February | 327 | 0–0 | 0.00–0.00 | 0–63 | 0.00–15.95 |  |  |
|  | March | 174 | 0–0 | 0.00–0.00 | 122–197 | 32.73–53.00 |  | ** |
|  | April | 569 | 103–190 | 27.08–49.83 | 0–0 | 0.00–0.00 | * |  |
|  | May | 396 | 0–34 | 0.00–9.31 | 0–0 | 0.00–0.00 |  |  |
|  | June | 384 | 0–14 | 0.00–3.76 | 0–0 | 0.00–0.00 |  |  |
| Toyama | (1) Average number of outpatients per day at hospitals |  |  |  |  |  |  |  |
|  | General hospitals |  |  |  |  |  |  |  |
|  | January | 12056 | 0–0 | 0.00–0.00 | 0–411 | 0.00–3.29 |  |  |
|  | February | 12065 | 0–0 | 0.00–0.00 | 0–595 | 0.00–4.70 |  |  |
|  | March | 12411 | 0–0 | 0.00–0.00 | 0–478 | 0.00–3.70 |  |  |
|  | April | 10773 | 0–0 | 0.00–0.00 | 1027–1893 | 8.11–14.94 |  | ** |
|  | May | 9155 | 0–0 | 0.00–0.00 | 2630–3589 | 20.64–28.16 |  | ** |
|  | June | 11713 | 0–0 | 0.00–0.00 | 209–1207 | 1.62–9.34 |  | ** |
|  | Psychiatric hospitals |  |  |  |  |  |  |  |
|  | January | 689 | 0–0 | 0.00–0.00 | 0–3 | 0.00–0.35 |  |  |
|  | February | 736 | 0–31 | 0.00–4.39 | 0–0 | 0.00–0.00 |  |  |
|  | March | 735 | 0–1 | 0.00–0.02 | 0–0 | 0.00–0.00 |  |  |
|  | April | 683 | 0–0 | 0.00–0.00 | 0–43 | 0.00–5.86 |  |  |
|  | May | 580 | 0–0 | 0.00–0.00 | 97–156 | 13.19–21.14 |  | ** |
|  | June | 706 | 0–0 | 0.00–0.00 | 0–35 | 0.00–4.61 |  |  |
|  | (2) Average number of hospitalized patients per day |  |  |  |  |  |  |  |
|  | (b) Psychiatric care beds |  |  |  |  |  |  |  |
|  | January | 2816 | 0–0 | 0.00–0.00 | 0–8 | 0.00–0.26 |  |  |
|  | February | 2822 | 0–0 | 0.00–0.00 | 0–2 | 0.00–0.07 |  |  |
|  | March | 2816 | 0–0 | 0.00–0.00 | 0–4 | 0.00–0.13 |  |  |
|  | April | 2785 | 0–0 | 0.00–0.00 | 0–26 | 0.00–0.89 |  |  |
|  | May | 2785 | 0–0 | 0.00–0.00 | 0–22 | 0.00–0.78 |  |  |
|  | June | 2818 | 0–13 | 0.00–0.46 | 0–0 | 0.00–0.00 |  |  |
|  | (c) Tuberculosis care beds |  |  |  |  |  |  |  |
|  | January | 5 | 0–1 | 0.00–1.27 | 0–0 | 0.00–0.00 |  |  |
|  | February | 4 | 0–0 | 0.00–0.00 | 0–2 | 0.00–29.08 |  |  |
|  | March | 5 | 0–0 | 0.00–0.00 | 0–1 | 0.00–4.30 |  |  |
|  | April | 12 | 2–8 | 41.74–150.45 | 0–0 | 0.00–0.00 | * |  |
|  | May | 6 | 0–2 | 0.00–29.20 | 0–0 | 0.00–0.00 |  |  |
|  | June | 6 | 0–2 | 0.00–25.74 | 0–0 | 0.00–0.00 |  |  |
|  | (d) Long-term care beds |  |  |  |  |  |  |  |
|  | January | 3694 | 0–0 | 0.00–0.00 | 267–529 | 6.32–12.51 |  | ** |
|  | February | 3719 | 0–0 | 0.00–0.00 | 235–500 | 5.57–11.84 |  | ** |
|  | March | 3672 | 0–0 | 0.00–0.00 | 128–451 | 3.10–10.93 |  | ** |
|  | April | 3623 | 0–0 | 0.00–0.00 | 121–448 | 2.97–11.00 |  | ** |
|  | May | 3565 | 0–0 | 0.00–0.00 | 96–429 | 2.40–10.73 |  | ** |
|  | June | 3595 | 0–0 | 0.00–0.00 | 0–335 | 0.00–8.52 |  |  |
|  | (e) General beds |  |  |  |  |  |  |  |
|  | January | 6146 | 0–0 | 0.00–0.00 | 0–238 | 0.00–3.71 |  |  |
|  | February | 6241 | 0–0 | 0.00–0.00 | 15–206 | 0.23–3.18 |  | ** |
|  | March | 5972 | 0–0 | 0.00–0.00 | 201–427 | 3.14–6.67 |  | ** |
|  | April | 5522 | 0–0 | 0.00–0.00 | 438–697 | 7.04–11.20 |  | ** |
|  | May | 5084 | 0–0 | 0.00–0.00 | 853–1041 | 13.93–16.99 |  | ** |
|  | June | 5394 | 0–0 | 0.00–0.00 | 510–715 | 8.35–11.70 |  | ** |
|  | (f) LTCI care beds |  |  |  |  |  |  |  |
|  | January | 494 | 0–0 | 0.00–0.00 | 236–514 | 23.42–50.97 |  | ** |
|  | February | 494 | 0–0 | 0.00–0.00 | 214–490 | 21.76–49.76 |  | ** |
|  | March | 477 | 0–0 | 0.00–0.00 | 129–429 | 14.25–47.31 |  | ** |
|  | April | 430 | 0–0 | 0.00–0.00 | 92–404 | 11.04–48.40 |  | ** |
|  | May | 430 | 0–0 | 0.00–0.00 | 43–350 | 5.51–44.86 |  | ** |
|  | June | 419 | 0–0 | 0.00–0.00 | 11–315 | 1.50–42.86 |  | ** |
|  | (3) Average length of hospital stays per patient |  |  |  |  |  |  |  |
|  | (b) Psychiatric care beds |  |  |  |  |  |  |  |
|  | January | 367 | 0–21 | 0.00–5.98 | 0–0 | 0.00–0.00 |  |  |
|  | February | 346 | 0–6 | 0.00–1.58 | 0–0 | 0.00–0.00 |  |  |
|  | March | 346 | 0–13 | 0.00–3.87 | 0–0 | 0.00–0.00 |  |  |
|  | April | 421 | 51–92 | 15.49–27.85 | 0–0 | 0.00–0.00 | * |  |
|  | May | 437 | 58–99 | 16.88–29.25 | 0–0 | 0.00–0.00 | * |  |
|  | June | 375 | 6–47 | 1.80–14.07 | 0–0 | 0.00–0.00 | * |  |
|  | (c) Tuberculosis care beds |  |  |  |  |  |  |  |
|  | January | 48 | 0–0 | 0.00–0.00 | 0–5 | 0.00–8.90 |  |  |
|  | February | 31 | 0–0 | 0.00–0.00 | 0–19 | 0.00–37.53 |  |  |
|  | March | 50 | 0–14 | 0.00–36.60 | 0–0 | 0.00–0.00 |  |  |
|  | April | 15 | 0–0 | 0.00–0.00 | 0–21 | 0.00–58.36 |  |  |
|  | May | 16 | 0–0 | 0.00–0.00 | 0–20 | 0.00–54.98 |  |  |
|  | June | 18 | 0–0 | 0.00–0.00 | 0–24 | 0.00–57.35 |  |  |
|  | (d) Long-term care beds |  |  |  |  |  |  |  |
|  | January | 204 | 0–6 | 0.00–2.68 | 0–0 | 0.00–0.00 |  |  |
|  | February | 203 | 0–6 | 0.00–2.84 | 0–0 | 0.00–0.00 |  |  |
|  | March | 203 | 0–8 | 0.00–4.07 | 0–0 | 0.00–0.00 |  |  |
|  | April | 235 | 9–38 | 4.56–19.18 | 0–0 | 0.00–0.00 | * |  |
|  | May | 258 | 35–63 | 17.44–31.67 | 0–0 | 0.00–0.00 | * |  |
|  | June | 192 | 0–0 | 0.00–0.00 | 0–6 | 0.00–2.70 |  |  |
|  | (e) General beds |  |  |  |  |  |  |  |
|  | January | 16 | 0–1 | 0.00–2.75 | 0–0 | 0.00–0.00 |  |  |
|  | February | 16 | 0–1 | 0.00–0.15 | 0–0 | 0.00–0.00 |  |  |
|  | March | 15 | 0–0 | 0.00–0.00 | 0–1 | 0.00–3.33 |  |  |
|  | April | 17 | 0–2 | 0.00–6.93 | 0–0 | 0.00–0.00 |  |  |
|  | May | 18 | 0–3 | 0.00–18.67 | 0–0 | 0.00–0.00 |  |  |
|  | June | 16 | 0–1 | 0.00–2.03 | 0–0 | 0.00–0.00 |  |  |
|  | (f) LTCI care beds |  |  |  |  |  |  |  |
|  | January | 369 | 0–0 | 0.00–0.00 | 0–4 | 0.00–0.95 |  |  |
|  | February | 268 | 0–0 | 0.00–0.00 | 24–91 | 6.69–25.32 |  | ** |
|  | March | 287 | 0–0 | 0.00–0.00 | 0–63 | 0.00–17.98 |  |  |
|  | April | 287 | 0–0 | 0.00–0.00 | 0–63 | 0.00–17.99 |  |  |
|  | May | 468 | 56–130 | 16.40–38.14 | 0–0 | 0.00–0.00 | * |  |
|  | June | 152 | 0–0 | 0.00–0.00 | 110–190 | 32.04–55.44 |  | ** |
| Ishikawa | (1) Average number of outpatients per day at hospitals |  |  |  |  |  |  |  |
|  | General hospitals |  |  |  |  |  |  |  |
|  | January | 13280 | 0–0 | 0.00–0.00 | 0–572 | 0.00–4.13 |  |  |
|  | February | 13150 | 0–0 | 0.00–0.00 | 0–864 | 0.00–6.16 |  |  |
|  | March | 13246 | 0–0 | 0.00–0.00 | 0–999 | 0.00–7.01 |  |  |
|  | April | 11665 | 0–0 | 0.00–0.00 | 1424–2373 | 10.14–16.90 |  | ** |
|  | May | 10078 | 0–0 | 0.00–0.00 | 3004–4021 | 21.31–28.52 |  | ** |
|  | June | 12722 | 0–0 | 0.00–0.00 | 465–1564 | 3.26–10.95 |  | ** |
|  | Psychiatric hospitals |  |  |  |  |  |  |  |
|  | January | 579 | 0–0 | 0.00–0.00 | 0–9 | 0.00–1.48 |  |  |
|  | February | 591 | 0–0 | 0.00–0.00 | 0–9 | 0.00–1.37 |  |  |
|  | March | 607 | 0–0 | 0.00–0.00 | 0–13 | 0.00–1.97 |  |  |
|  | April | 544 | 0–0 | 0.00–0.00 | 21–69 | 3.43–11.19 |  | ** |
|  | May | 455 | 0–0 | 0.00–0.00 | 114–165 | 18.40–26.57 |  | ** |
|  | June | 580 | 0–0 | 0.00–0.00 | 0–43 | 0.00–6.85 |  |  |
|  | (2) Average number of hospitalized patients per day |  |  |  |  |  |  |  |
|  | (b) Psychiatric care beds |  |  |  |  |  |  |  |
|  | January | 3117 | 0–0 | 0.00–0.00 | 0–10 | 0.00–0.32 |  |  |
|  | February | 3150 | 0–13 | 0.00–0.41 | 0–0 | 0.00–0.00 |  |  |
|  | March | 3107 | 0–0 | 0.00–0.00 | 0–32 | 0.00–0.99 |  |  |
|  | April | 3077 | 0–0 | 0.00–0.00 | 0–60 | 0.00–1.90 |  |  |
|  | May | 3045 | 0–0 | 0.00–0.00 | 0–90 | 0.00–2.87 |  |  |
|  | June | 3064 | 0–0 | 0.00–0.00 | 0–74 | 0.00–2.35 |  |  |
|  | (c) Tuberculosis care beds |  |  |  |  |  |  |  |
|  | January | 8 | 0–0 | 0.00–0.00 | 0–5 | 0.00–35.72 |  |  |
|  | February | 9 | 0–0 | 0.00–0.00 | 0–4 | 0.00–26.29 |  |  |
|  | March | 9 | 0–0 | 0.00–0.00 | 0–4 | 0.00–29.30 |  |  |
|  | April | 23 | 2–10 | 15.21–74.94 | 0–0 | 0.00–0.00 | * |  |
|  | May | 21 | 0–7 | 0.00–49.25 | 0–0 | 0.00–0.00 |  |  |
|  | June | 17 | 0–2 | 0.00–8.94 | 0–0 | 0.00–0.00 |  |  |
|  | (d) Long-term care beds |  |  |  |  |  |  |  |
|  | January | 3147 | 0–0 | 0.00–0.00 | 136–250 | 4.00–7.33 |  | ** |
|  | February | 3150 | 0–0 | 0.00–0.00 | 132–246 | 3.89–7.23 |  | ** |
|  | March | 3139 | 0–0 | 0.00–0.00 | 125–238 | 3.70–7.03 |  | ** |
|  | April | 2787 | 0–0 | 0.00–0.00 | 446–558 | 13.33–16.68 |  | ** |
|  | May | 2762 | 0–0 | 0.00–0.00 | 433–550 | 13.08–16.60 |  | ** |
|  | June | 2719 | 0–0 | 0.00–0.00 | 444–570 | 13.50–17.32 |  | ** |
|  | (e) General beds |  |  |  |  |  |  |  |
|  | January | 7673 | 0–0 | 0.00–0.00 | 0–240 | 0.00–3.03 |  |  |
|  | February | 7869 | 0–0 | 0.00–0.00 | 0–152 | 0.00–1.88 |  |  |
|  | March | 7464 | 0–0 | 0.00–0.00 | 269–526 | 3.37–6.57 |  | ** |
|  | April | 6837 | 0–0 | 0.00–0.00 | 680–980 | 8.70–12.53 |  | ** |
|  | May | 6438 | 0–0 | 0.00–0.00 | 1079–1304 | 13.94–16.84 |  | ** |
|  | June | 6792 | 0–0 | 0.00–0.00 | 687–929 | 8.90–12.02 |  | ** |
|  | (f) LTCI care beds |  |  |  |  |  |  |  |
|  | January | 419 | 0–0 | 0.00–0.00 | 100–160 | 17.27–27.62 |  | ** |
|  | February | 422 | 0–0 | 0.00–0.00 | 91–151 | 15.90–26.26 |  | ** |
|  | March | 420 | 0–0 | 0.00–0.00 | 84–143 | 14.93–25.35 |  | ** |
|  | April | 164 | 0–0 | 0.00–0.00 | 322–384 | 58.86–70.02 |  | ** |
|  | May | 132 | 0–0 | 0.00–0.00 | 344–404 | 64.18–75.37 |  | ** |
|  | June | 110 | 0–0 | 0.00–0.00 | 348–415 | 66.30–79.04 |  | ** |
|  | (3) Average length of hospital stays per patient |  |  |  |  |  |  |  |
|  | (b) Psychiatric care beds |  |  |  |  |  |  |  |
|  | January | 289 | 0–28 | 0.00–10.49 | 0–0 | 0.00–0.00 |  |  |
|  | February | 278 | 0–22 | 0.00–8.50 | 0–0 | 0.00–0.00 |  |  |
|  | March | 248 | 0–2 | 0.00–0.60 | 0–0 | 0.00–0.00 |  |  |
|  | April | 282 | 11–43 | 4.29–17.59 | 0–0 | 0.00–0.00 | * |  |
|  | May | 316 | 52–83 | 22.18–35.43 | 0–0 | 0.00–0.00 | * |  |
|  | June | 270 | 2–34 | 0.80–13.93 | 0–0 | 0.00–0.00 | * |  |
|  | (c) Tuberculosis care beds |  |  |  |  |  |  |  |
|  | January | 63 | 0–0 | 0.00–0.00 | 0–20 | 0.00–23.82 |  |  |
|  | February | 86 | 0–6 | 0.00–6.90 | 0–0 | 0.00–0.00 |  |  |
|  | March | 28 | 0–0 | 0.00–0.00 | 19–53 | 23.55–65.29 |  | ** |
|  | April | 21 | 0–0 | 0.00–0.00 | 24–59 | 30.11–73.66 |  | ** |
|  | May | 66 | 0–0 | 0.00–0.00 | 0–19 | 0.00–21.67 |  |  |
|  | June | 110 | 0–22 | 0.00–24.17 | 0–0 | 0.00–0.00 |  |  |
|  | (d) Long-term care beds |  |  |  |  |  |  |  |
|  | January | 186 | 0–0 | 0.00–0.00 | 0–7 | 0.00–3.56 |  |  |
|  | February | 173 | 0–0 | 0.00–0.00 | 0–22 | 0.00–11.02 |  |  |
|  | March | 166 | 0–0 | 0.00–0.00 | 0–25 | 0.00–13.09 |  |  |
|  | April | 160 | 0–0 | 0.00–0.00 | 9–36 | 4.60–18.19 |  | ** |
|  | May | 185 | 0–0 | 0.00–0.00 | 0–13 | 0.00–6.44 |  |  |
|  | June | 168 | 0–0 | 0.00–0.00 | 6–33 | 2.99–16.36 |  | ** |
|  | (e) General beds |  |  |  |  |  |  |  |
|  | January | 18 | 0–1 | 0.00–1.63 | 0–0 | 0.00–0.00 |  |  |
|  | February | 19 | 0–1 | 0.00–1.40 | 0–0 | 0.00–0.00 |  |  |
|  | March | 18 | 0–1 | 0.00–0.99 | 0–0 | 0.00–0.00 |  |  |
|  | April | 20 | 0–2 | 0.00–9.36 | 0–0 | 0.00–0.00 |  |  |
|  | May | 22 | 0–5 | 0.00–25.82 | 0–0 | 0.00–0.00 |  |  |
|  | June | 18 | 0–1 | 0.00–5.44 | 0–0 | 0.00–0.00 |  |  |
|  | (f) LTCI care beds |  |  |  |  |  |  |  |
|  | January | 440 | 0–66 | 0.00–17.57 | 0–0 | 0.00–0.00 |  |  |
|  | February | 422 | 0–43 | 0.00–11.33 | 0–0 | 0.00–0.00 |  |  |
|  | March | 166 | 0–0 | 0.00–0.00 | 97–209 | 25.71–55.75 |  | ** |
|  | April | 64 | 0–0 | 0.00–0.00 | 222–338 | 55.29–84.06 |  | ** |
|  | May | 154 | 0–0 | 0.00–0.00 | 127–241 | 31.99–61.02 |  | ** |
|  | June | 367 | 0–0 | 0.00–0.00 | 0–29 | 0.00–7.12 |  |  |
| Fukui | (1) Average number of outpatients per day at hospitals |  |  |  |  |  |  |  |
|  | General hospitals |  |  |  |  |  |  |  |
|  | January | 9641 | 0–0 | 0.00–0.00 | 0–299 | 0.00–3.01 |  |  |
|  | February | 9557 | 0–0 | 0.00–0.00 | 0–478 | 0.00–4.76 |  |  |
|  | March | 9535 | 0–0 | 0.00–0.00 | 0–673 | 0.00–6.59 |  |  |
|  | April | 7973 | 0–0 | 0.00–0.00 | 1425–2101 | 14.15–20.85 |  | ** |
|  | May | 7388 | 0–0 | 0.00–0.00 | 2051–2756 | 20.22–27.17 |  | ** |
|  | June | 9379 | 0–0 | 0.00–0.00 | 94–846 | 0.92–8.27 |  | ** |
|  | Psychiatric hospitals |  |  |  |  |  |  |  |
|  | January | 497 | 0–0 | 0.00–0.00 | 0–17 | 0.00–3.23 |  |  |
|  | February | 538 | 0–16 | 0.00–3.00 | 0–0 | 0.00–0.00 |  |  |
|  | March | 547 | 0–0 | 0.00–0.00 | 0–1 | 0.00–0.16 |  |  |
|  | April | 516 | 0–0 | 0.00–0.00 | 0–24 | 0.00–4.28 |  |  |
|  | May | 462 | 0–0 | 0.00–0.00 | 38–83 | 6.97–15.21 |  | ** |
|  | June | 562 | 0–16 | 0.00–2.90 | 0–0 | 0.00–0.00 |  |  |
|  | (2) Average number of hospitalized patients per day |  |  |  |  |  |  |  |
|  | (b) Psychiatric care beds |  |  |  |  |  |  |  |
|  | January | 1862 | 0–0 | 0.00–0.00 | 0–18 | 0.00–0.91 |  |  |
|  | February | 1880 | 0–0 | 0.00–0.00 | 0–9 | 0.00–0.47 |  |  |
|  | March | 1884 | 0–0 | 0.00–0.00 | 0–3 | 0.00–0.14 |  |  |
|  | April | 1841 | 0–0 | 0.00–0.00 | 0–44 | 0.00–2.31 |  |  |
|  | May | 1802 | 0–0 | 0.00–0.00 | 0–83 | 0.00–4.40 |  |  |
|  | June | 1841 | 0–0 | 0.00–0.00 | 0–52 | 0.00–2.73 |  |  |
|  | (c) Tuberculosis care beds |  |  |  |  |  |  |  |
|  | January | 6 | 0–0 | 0.00–0.00 | 0–3 | 0.00–31.87 |  |  |
|  | February | 5 | 0–0 | 0.00–0.00 | 0–5 | 0.00–45.19 |  |  |
|  | March | 2 | 0–0 | 0.00–0.00 | 2–7 | 24.19–75.81 |  | ** |
|  | April | 3 | 0–0 | 0.00–0.00 | 0–5 | 0.00–59.46 |  |  |
|  | May | 2 | 0–0 | 0.00–0.00 | 1–7 | 12.41–75.17 |  | ** |
|  | June | 3 | 0–0 | 0.00–0.00 | 1–6 | 11.99–64.04 |  | ** |
|  | (d) Long-term care beds |  |  |  |  |  |  |  |
|  | January | 1605 | 0–0 | 0.00–0.00 | 27–108 | 1.58–6.26 |  | ** |
|  | February | 1626 | 0–0 | 0.00–0.00 | 0–68 | 0.00–3.97 |  |  |
|  | March | 1610 | 0–0 | 0.00–0.00 | 0–52 | 0.00–3.12 |  |  |
|  | April | 1554 | 0–0 | 0.00–0.00 | 0–72 | 0.00–4.40 |  |  |
|  | May | 1549 | 0–0 | 0.00–0.00 | 0–52 | 0.00–3.20 |  |  |
|  | June | 1556 | 0–0 | 0.00–0.00 | 0–34 | 0.00–2.12 |  |  |
|  | (e) General beds |  |  |  |  |  |  |  |
|  | January | 4952 | 0–0 | 0.00–0.00 | 0–81 | 0.00–1.59 |  |  |
|  | February | 5079 | 0–2 | 0.00–0.02 | 0–0 | 0.00–0.00 |  |  |
|  | March | 4821 | 0–0 | 0.00–0.00 | 69–219 | 1.37–4.34 |  | ** |
|  | April | 4304 | 0–0 | 0.00–0.00 | 460–634 | 9.32–12.82 |  | ** |
|  | May | 4079 | 0–0 | 0.00–0.00 | 678–814 | 13.86–16.63 |  | ** |
|  | June | 4366 | 0–0 | 0.00–0.00 | 370–512 | 7.59–10.50 |  | ** |
|  | (f) LTCI care beds |  |  |  |  |  |  |  |
|  | January | 100 | 0–0 | 0.00–0.00 | 34–77 | 19.28–43.28 |  | ** |
|  | February | 100 | 0–0 | 0.00–0.00 | 24–65 | 14.61–39.11 |  | ** |
|  | March | 100 | 0–0 | 0.00–0.00 | 14–51 | 9.31–33.47 |  | ** |
|  | April | 57 | 0–0 | 0.00–0.00 | 49–84 | 34.95–59.34 |  | ** |
|  | May | 54 | 0–0 | 0.00–0.00 | 47–81 | 35.02–59.77 |  | ** |
|  | June | 53 | 0–0 | 0.00–0.00 | 45–78 | 34.44–59.43 |  | ** |
|  | (3) Average length of hospital stays per patient |  |  |  |  |  |  |  |
|  | (b) Psychiatric care beds |  |  |  |  |  |  |  |
|  | January | 269 | 0–12 | 0.00–4.56 | 0–0 | 0.00–0.00 |  |  |
|  | February | 267 | 0–13 | 0.00–5.02 | 0–0 | 0.00–0.00 |  |  |
|  | March | 232 | 0–0 | 0.00–0.00 | 0–15 | 0.00–5.78 |  |  |
|  | April | 267 | 0–24 | 0.00–9.46 | 0–0 | 0.00–0.00 |  |  |
|  | May | 293 | 17–52 | 6.75–21.54 | 0–0 | 0.00–0.00 | * |  |
|  | June | 258 | 0–19 | 0.00–7.51 | 0–0 | 0.00–0.00 |  |  |
|  | (c) Tuberculosis care beds |  |  |  |  |  |  |  |
|  | January | 19 | 0–0 | 0.00–0.00 | 0–3 | 0.00–12.95 |  |  |
|  | February | 17 | 0–0 | 0.00–0.00 | 0–4 | 0.00–17.28 |  |  |
|  | March | 15 | 0–0 | 0.00–0.00 | 0–5 | 0.00–21.04 |  |  |
|  | April | 11 | 0–0 | 0.00–0.00 | 0–9 | 0.00–42.90 |  |  |
|  | May | 13 | 0–0 | 0.00–0.00 | 0–7 | 0.00–32.89 |  |  |
|  | June | 17 | 0–0 | 0.00–0.00 | 0–3 | 0.00–13.42 |  |  |
|  | (d) Long-term care beds |  |  |  |  |  |  |  |
|  | January | 145 | 0–0 | 0.00–0.00 | 0–11 | 0.00–6.64 |  |  |
|  | February | 140 | 0–0 | 0.00–0.00 | 0–16 | 0.00–9.81 |  |  |
|  | March | 139 | 0–0 | 0.00–0.00 | 0–11 | 0.00–6.89 |  |  |
|  | April | 144 | 0–0 | 0.00–0.00 | 0–7 | 0.00–4.15 |  |  |
|  | May | 160 | 0–11 | 0.00–7.31 | 0–0 | 0.00–0.00 |  |  |
|  | June | 133 | 0–0 | 0.00–0.00 | 0–17 | 0.00–11.18 |  |  |
|  | (e) General beds |  |  |  |  |  |  |  |
|  | January | 18 | 0–1 | 0.00–1.57 | 0–0 | 0.00–0.00 |  |  |
|  | February | 18 | 0–0 | 0.00–0.00 | 0–1 | 0.00–0.55 |  |  |
|  | March | 18 | 0–1 | 0.00–1.69 | 0–0 | 0.00–0.00 |  |  |
|  | April | 19 | 0–2 | 0.00–11.71 | 0–0 | 0.00–0.00 |  |  |
|  | May | 20 | 0–4 | 0.00–20.61 | 0–0 | 0.00–0.00 |  |  |
|  | June | 17 | 0–1 | 0.00–1.08 | 0–0 | 0.00–0.00 |  |  |
|  | (f) LTCI care beds |  |  |  |  |  |  |  |
|  | January | 779 | 0–198 | 0.00–33.93 | 0–0 | 0.00–0.00 |  |  |
|  | February | 1454 | 593–880 | 103.14–152.89 | 0–0 | 0.00–0.00 | * |  |
|  | March | 1030 | 214–487 | 39.35–89.38 | 0–0 | 0.00–0.00 | * |  |
|  | April | 680 | 0–115 | 0.00–20.17 | 0–0 | 0.00–0.00 |  |  |
|  | May | 334 | 0–0 | 0.00–0.00 | 30–285 | 4.82–45.94 |  | ** |
|  | June | 263 | 0–0 | 0.00–0.00 | 210–461 | 28.95–63.66 |  | ** |
| Yamanashi | (1) Average number of outpatients per day at hospitals |  |  |  |  |  |  |  |
|  | General hospitals |  |  |  |  |  |  |  |
|  | January | 8214 | 0–0 | 0.00–0.00 | 0–368 | 0.00–4.28 |  |  |
|  | February | 8304 | 0–0 | 0.00–0.00 | 0–361 | 0.00–4.16 |  |  |
|  | March | 7989 | 0–0 | 0.00–0.00 | 141–768 | 1.61–8.76 |  | ** |
|  | April | 7266 | 0–0 | 0.00–0.00 | 636–1229 | 7.49–14.46 |  | ** |
|  | May | 6459 | 0–0 | 0.00–0.00 | 1483–2075 | 17.38–24.31 |  | ** |
|  | June | 8050 | 0–0 | 0.00–0.00 | 0–536 | 0.00–6.24 |  |  |
|  | Psychiatric hospitals |  |  |  |  |  |  |  |
|  | January | 502 | 0–0 | 0.00–0.00 | 0–33 | 0.00–6.06 |  |  |
|  | February | 515 | 0–0 | 0.00–0.00 | 0–31 | 0.00–5.64 |  |  |
|  | March | 524 | 0–0 | 0.00–0.00 | 0–35 | 0.00–6.17 |  |  |
|  | April | 513 | 0–0 | 0.00–0.00 | 0–31 | 0.00–5.60 |  |  |
|  | May | 443 | 0–0 | 0.00–0.00 | 56–102 | 10.29–18.61 |  | ** |
|  | June | 537 | 0–0 | 0.00–0.00 | 0–11 | 0.00–1.84 |  |  |
|  | (2) Average number of hospitalized patients per day |  |  |  |  |  |  |  |
|  | (b) Psychiatric care beds |  |  |  |  |  |  |  |
|  | January | 1798 | 0–4 | 0.00–0.20 | 0–0 | 0.00–0.00 |  |  |
|  | February | 1822 | 0–28 | 0.00–1.52 | 0–0 | 0.00–0.00 |  |  |
|  | March | 1815 | 0–16 | 0.00–0.87 | 0–0 | 0.00–0.00 |  |  |
|  | April | 1812 | 0–12 | 0.00–0.64 | 0–0 | 0.00–0.00 |  |  |
|  | May | 1815 | 0–14 | 0.00–0.76 | 0–0 | 0.00–0.00 |  |  |
|  | June | 1833 | 0–29 | 0.00–1.59 | 0–0 | 0.00–0.00 |  |  |
|  | (c) Tuberculosis care beds |  |  |  |  |  |  |  |
|  | January | 9 | 0–4 | 0.00–73.14 | 0–0 | 0.00–0.00 |  |  |
|  | February | 3 | 0–0 | 0.00–0.00 | 0–2 | 0.00–36.27 |  |  |
|  | March | 1 | 0–0 | 0.00–0.00 | 1–5 | 18.32–81.68 |  | ** |
|  | April | 4 | 0–0 | 0.00–0.00 | 0–3 | 0.00–36.17 |  |  |
|  | May | 1 | 0–0 | 0.00–0.00 | 2–7 | 26.96–86.52 |  | ** |
|  | June | 1 | 0–0 | 0.00–0.00 | 3–8 | 33.40–88.87 |  | ** |
|  | (d) Long-term care beds |  |  |  |  |  |  |  |
|  | January | 1766 | 0–0 | 0.00–0.00 | 0–16 | 0.00–0.88 |  |  |
|  | February | 1765 | 0–0 | 0.00–0.00 | 0–34 | 0.00–1.86 |  |  |
|  | March | 1754 | 0–0 | 0.00–0.00 | 0–37 | 0.00–2.02 |  |  |
|  | April | 1746 | 0–0 | 0.00–0.00 | 0–21 | 0.00–1.18 |  |  |
|  | May | 1723 | 0–0 | 0.00–0.00 | 0–29 | 0.00–1.60 |  |  |
|  | June | 1700 | 0–0 | 0.00–0.00 | 0–41 | 0.00–2.32 |  |  |
|  | (e) General beds |  |  |  |  |  |  |  |
|  | January | 4471 | 0–0 | 0.00–0.00 | 17–197 | 0.36–4.21 |  | ** |
|  | February | 4546 | 0–0 | 0.00–0.00 | 18–152 | 0.38–3.22 |  | ** |
|  | March | 4280 | 0–0 | 0.00–0.00 | 204–357 | 4.40–7.69 |  | ** |
|  | April | 3910 | 0–0 | 0.00–0.00 | 438–610 | 9.69–13.48 |  | ** |
|  | May | 3693 | 0–0 | 0.00–0.00 | 631–761 | 14.17–17.08 |  | ** |
|  | June | 3917 | 0–0 | 0.00–0.00 | 373–516 | 8.41–11.64 |  | ** |
|  | (f) LTCI care beds |  |  |  |  |  |  |  |
|  | January | 25 | 0–0 | 0.00–0.00 | 26–57 | 32.02–69.21 |  | ** |
|  | February | 22 | 0–0 | 0.00–0.00 | 21–53 | 28.37–70.28 |  | ** |
|  | March | 25 | 0–0 | 0.00–0.00 | 14–44 | 20.30–63.76 |  | ** |
|  | April | 25 | 0–0 | 0.00–0.00 | 10–39 | 15.77–60.58 |  | ** |
|  | May | 24 | 0–0 | 0.00–0.00 | 6–34 | 10.35–58.62 |  | ** |
|  | June | 17 | 0–0 | 0.00–0.00 | 9–37 | 16.83–68.21 |  | ** |
|  | (3) Average length of hospital stays per patient |  |  |  |  |  |  |  |
|  | (b) Psychiatric care beds |  |  |  |  |  |  |  |
|  | January | 269 | 0–18 | 0.00–7.00 | 0–0 | 0.00–0.00 |  |  |
|  | February | 238 | 0–0 | 0.00–0.00 | 0–8 | 0.00–3.17 |  |  |
|  | March | 243 | 0–6 | 0.00–2.50 | 0–0 | 0.00–0.00 |  |  |
|  | April | 253 | 0–19 | 0.00–8.08 | 0–0 | 0.00–0.00 |  |  |
|  | May | 255 | 0–23 | 0.00–9.88 | 0–0 | 0.00–0.00 |  |  |
|  | June | 232 | 0–1 | 0.00–0.28 | 0–0 | 0.00–0.00 |  |  |
|  | (c) Tuberculosis care beds |  |  |  |  |  |  |  |
|  | January | 81 | 0–28 | 0.00–52.07 | 0–0 | 0.00–0.00 |  |  |
|  | February | 34 | 0–0 | 0.00–0.00 | 0–27 | 0.00–43.31 |  |  |
|  | March | 3 | 0–0 | 0.00–0.00 | 6–53 | 10.44–94.24 |  | ** |
|  | April | 8 | 0–0 | 0.00–0.00 | 6–59 | 9.01–87.98 |  | ** |
|  | May | 4 | 0–0 | 0.00–0.00 | 16–64 | 23.78–94.05 |  | ** |
|  | June | 4 | 0–0 | 0.00–0.00 | 24–75 | 30.10–94.54 |  | ** |
|  | (d) Long-term care beds |  |  |  |  |  |  |  |
|  | January | 125 | 0–0 | 0.00–0.00 | 0–3 | 0.00–1.96 |  |  |
|  | February | 111 | 0–0 | 0.00–0.00 | 0–20 | 0.00–14.89 |  |  |
|  | March | 119 | 0–0 | 0.00–0.00 | 0–8 | 0.00–5.85 |  |  |
|  | April | 130 | 0–2 | 0.00–1.19 | 0–0 | 0.00–0.00 |  |  |
|  | May | 147 | 0–17 | 0.00–13.05 | 0–0 | 0.00–0.00 |  |  |
|  | June | 126 | 0–0 | 0.00–0.00 | 0–8 | 0.00–5.60 |  |  |
|  | (e) General beds |  |  |  |  |  |  |  |
|  | January | 18 | 0–1 | 0.00–5.07 | 0–0 | 0.00–0.00 |  |  |
|  | February | 18 | 0–1 | 0.00–2.27 | 0–0 | 0.00–0.00 |  |  |
|  | March | 18 | 0–1 | 0.00–1.98 | 0–0 | 0.00–0.00 |  |  |
|  | April | 18 | 0–1 | 0.00–3.62 | 0–0 | 0.00–0.00 |  |  |
|  | May | 20 | 0–3 | 0.00–14.85 | 0–0 | 0.00–0.00 |  |  |
|  | June | 17 | 0–1 | 0.00–3.06 | 0–0 | 0.00–0.00 |  |  |
|  | (f) LTCI care beds |  |  |  |  |  |  |  |
|  | January | 110 | 0–0 | 0.00–0.00 | 0–29 | 0.00–20.74 |  |  |
|  | February | 84 | 0–0 | 0.00–0.00 | 10–55 | 6.90–39.35 |  | ** |
|  | March | 52 | 0–0 | 0.00–0.00 | 42–85 | 30.32–62.05 |  | ** |
|  | April | 108 | 0–0 | 0.00–0.00 | 0–18 | 0.00–13.74 |  |  |
|  | May | 124 | 0–4 | 0.00–2.87 | 0–0 | 0.00–0.00 |  |  |
|  | June | 68 | 0–0 | 0.00–0.00 | 8–47 | 6.50–40.59 |  | ** |
| Nagano | (1) Average number of outpatients per day at hospitals |  |  |  |  |  |  |  |
|  | General hospitals |  |  |  |  |  |  |  |
|  | January | 23275 | 0–0 | 0.00–0.00 | 0–1040 | 0.00–4.27 |  |  |
|  | February | 23217 | 0–0 | 0.00–0.00 | 0–1301 | 0.00–5.30 |  |  |
|  | March | 23122 | 0–0 | 0.00–0.00 | 0–1519 | 0.00–6.16 |  |  |
|  | April | 21193 | 0–0 | 0.00–0.00 | 1400–2979 | 5.79–12.32 |  | ** |
|  | May | 18309 | 0–0 | 0.00–0.00 | 4382–6003 | 18.02–24.69 |  | ** |
|  | June | 23129 | 0–0 | 0.00–0.00 | 0–1441 | 0.00–5.86 |  |  |
|  | Psychiatric hospitals |  |  |  |  |  |  |  |
|  | January | 673 | 0–0 | 0.00–0.00 | 0–7 | 0.00–0.97 |  |  |
|  | February | 708 | 0–17 | 0.00–2.39 | 0–0 | 0.00–0.00 |  |  |
|  | March | 713 | 0–0 | 0.00–0.00 | 0–1 | 0.00–0.01 |  |  |
|  | April | 693 | 0–0 | 0.00–0.00 | 0–9 | 0.00–1.21 |  |  |
|  | May | 590 | 0–0 | 0.00–0.00 | 66–118 | 9.33–16.60 |  | ** |
|  | June | 714 | 0–4 | 0.00–0.47 | 0–0 | 0.00–0.00 |  |  |
|  | (2) Average number of hospitalized patients per day |  |  |  |  |  |  |  |
|  | (b) Psychiatric care beds |  |  |  |  |  |  |  |
|  | January | 3916 | 0–0 | 0.00–0.00 | 0–39 | 0.00–0.98 |  |  |
|  | February | 3919 | 0–0 | 0.00–0.00 | 0–49 | 0.00–1.23 |  |  |
|  | March | 3877 | 0–0 | 0.00–0.00 | 0–84 | 0.00–2.10 |  |  |
|  | April | 3819 | 0–0 | 0.00–0.00 | 0–122 | 0.00–3.07 |  |  |
|  | May | 3789 | 0–0 | 0.00–0.00 | 21–144 | 0.53–3.65 |  | ** |
|  | June | 3842 | 0–0 | 0.00–0.00 | 0–99 | 0.00–2.49 |  |  |
|  | (c) Tuberculosis care beds |  |  |  |  |  |  |  |
|  | January | 20 | 0–0 | 0.00–0.00 | 0–3 | 0.00–10.11 |  |  |
|  | February | 18 | 0–0 | 0.00–0.00 | 0–5 | 0.00–18.85 |  |  |
|  | March | 13 | 0–0 | 0.00–0.00 | 0–9 | 0.00–39.93 |  |  |
|  | April | 12 | 0–0 | 0.00–0.00 | 2–10 | 9.13–45.20 |  | ** |
|  | May | 11 | 0–0 | 0.00–0.00 | 3–12 | 13.53–50.38 |  | ** |
|  | June | 14 | 0–0 | 0.00–0.00 | 0–9 | 0.00–37.45 |  |  |
|  | (d) Long-term care beds |  |  |  |  |  |  |  |
|  | January | 3087 | 0–0 | 0.00–0.00 | 49–208 | 1.49–6.31 |  | ** |
|  | February | 3073 | 0–0 | 0.00–0.00 | 26–199 | 0.79–6.08 |  | ** |
|  | March | 3011 | 0–0 | 0.00–0.00 | 48–228 | 1.48–7.02 |  | ** |
|  | April | 2899 | 0–0 | 0.00–0.00 | 101–280 | 3.18–8.79 |  | ** |
|  | May | 2865 | 0–0 | 0.00–0.00 | 97–276 | 3.09–8.76 |  | ** |
|  | June | 2834 | 0–0 | 0.00–0.00 | 101–284 | 3.24–9.10 |  | ** |
|  | (e) General beds |  |  |  |  |  |  |  |
|  | January | 11895 | 0–0 | 0.00–0.00 | 0–243 | 0.00–2.00 |  |  |
|  | February | 12076 | 0–0 | 0.00–0.00 | 0–154 | 0.00–1.26 |  |  |
|  | March | 11455 | 0–0 | 0.00–0.00 | 252–651 | 2.08–5.37 |  | ** |
|  | April | 10657 | 0–0 | 0.00–0.00 | 692–1115 | 5.88–9.47 |  | ** |
|  | May | 10082 | 0–0 | 0.00–0.00 | 1253–1535 | 10.79–13.21 |  | ** |
|  | June | 10555 | 0–0 | 0.00–0.00 | 736–1058 | 6.34–9.11 |  | ** |
|  | (f) LTCI care beds |  |  |  |  |  |  |  |
|  | January | 484 | 0–0 | 0.00–0.00 | 17–136 | 2.74–21.91 |  | ** |
|  | February | 466 | 0–0 | 0.00–0.00 | 1–129 | 0.17–21.62 |  | ** |
|  | March | 459 | 0–0 | 0.00–0.00 | 0–113 | 0.00–19.70 |  |  |
|  | April | 351 | 0–0 | 0.00–0.00 | 66–196 | 12.09–35.73 |  | ** |
|  | May | 343 | 0–0 | 0.00–0.00 | 53–182 | 10.10–34.64 |  | ** |
|  | June | 344 | 0–0 | 0.00–0.00 | 40–169 | 7.81–32.87 |  | ** |
|  | (3) Average length of hospital stays per patient |  |  |  |  |  |  |  |
|  | (b) Psychiatric care beds |  |  |  |  |  |  |  |
|  | January | 244 | 0–25 | 0.00–11.35 | 0–0 | 0.00–0.00 |  |  |
|  | February | 235 | 0–17 | 0.00–7.70 | 0–0 | 0.00–0.00 |  |  |
|  | March | 236 | 0–23 | 0.00–10.57 | 0–0 | 0.00–0.00 |  |  |
|  | April | 239 | 0–26 | 0.00–12.05 | 0–0 | 0.00–0.00 |  |  |
|  | May | 234 | 0–19 | 0.00–8.73 | 0–0 | 0.00–0.00 |  |  |
|  | June | 207 | 0–0 | 0.00–0.00 | 0–11 | 0.00–4.77 |  |  |
|  | (c) Tuberculosis care beds |  |  |  |  |  |  |  |
|  | January | 112 | 0–38 | 0.00–50.11 | 0–0 | 0.00–0.00 |  |  |
|  | February | 58 | 0–0 | 0.00–0.00 | 0–20 | 0.00–25.06 |  |  |
|  | March | 55 | 0–0 | 0.00–0.00 | 0–16 | 0.00–21.43 |  |  |
|  | April | 48 | 0–0 | 0.00–0.00 | 0–26 | 0.00–34.82 |  |  |
|  | May | 84 | 0–9 | 0.00–11.16 | 0–0 | 0.00–0.00 |  |  |
|  | June | 77 | 0–0 | 0.00–0.00 | 0–3 | 0.00–3.72 |  |  |
|  | (d) Long-term care beds |  |  |  |  |  |  |  |
|  | January | 108 | 0–7 | 0.00–6.91 | 0–0 | 0.00–0.00 |  |  |
|  | February | 108 | 0–8 | 0.00–7.39 | 0–0 | 0.00–0.00 |  |  |
|  | March | 106 | 0–9 | 0.00–9.00 | 0–0 | 0.00–0.00 |  |  |
|  | April | 106 | 0–9 | 0.00–9.13 | 0–0 | 0.00–0.00 |  |  |
|  | May | 114 | 0–16 | 0.00–16.03 | 0–0 | 0.00–0.00 |  |  |
|  | June | 111 | 0–11 | 0.00–10.86 | 0–0 | 0.00–0.00 |  |  |
|  | (e) General beds |  |  |  |  |  |  |  |
|  | January | 17 | 0–1 | 0.00–4.80 | 0–0 | 0.00–0.00 |  |  |
|  | February | 16 | 0–1 | 0.00–0.81 | 0–0 | 0.00–0.00 |  |  |
|  | March | 16 | 0–1 | 0.00–0.99 | 0–0 | 0.00–0.00 |  |  |
|  | April | 16 | 0–1 | 0.00–3.15 | 0–0 | 0.00–0.00 |  |  |
|  | May | 18 | 0–3 | 0.00–13.58 | 0–0 | 0.00–0.00 |  |  |
|  | June | 15 | 0–0 | 0.00–0.00 | 0–1 | 0.00–0.78 |  |  |
|  | (f) LTCI care beds |  |  |  |  |  |  |  |
|  | January | 161 | 13–37 | 9.89–29.73 | 0–0 | 0.00–0.00 | * |  |
|  | February | 160 | 13–37 | 10.47–29.79 | 0–0 | 0.00–0.00 | * |  |
|  | March | 163 | 24–49 | 20.55–42.09 | 0–0 | 0.00–0.00 | * |  |
|  | April | 173 | 40–63 | 35.96–56.11 | 0–0 | 0.00–0.00 | * |  |
|  | May | 184 | 51–74 | 45.77–66.33 | 0–0 | 0.00–0.00 | * |  |
|  | June | 143 | 6–30 | 4.85–25.78 | 0–0 | 0.00–0.00 | * |  |
| Gifu | (1) Average number of outpatients per day at hospitals |  |  |  |  |  |  |  |
|  | General hospitals |  |  |  |  |  |  |  |
|  | January | 18317 | 0–0 | 0.00–0.00 | 0–301 | 0.00–1.61 |  |  |
|  | February | 17951 | 0–0 | 0.00–0.00 | 0–821 | 0.00–4.37 |  |  |
|  | March | 17507 | 0–0 | 0.00–0.00 | 14–1257 | 0.07–6.70 |  | ** |
|  | April | 15551 | 0–0 | 0.00–0.00 | 1538–2755 | 8.40–15.05 |  | ** |
|  | May | 14138 | 0–0 | 0.00–0.00 | 2921–4188 | 15.94–22.85 |  | ** |
|  | June | 17532 | 0–0 | 0.00–0.00 | 0–995 | 0.00–5.37 |  |  |
|  | Psychiatric hospitals |  |  |  |  |  |  |  |
|  | January | 713 | 0–0 | 0.00–0.00 | 0–40 | 0.00–5.27 |  |  |
|  | February | 761 | 0–1 | 0.00–0.03 | 0–0 | 0.00–0.00 |  |  |
|  | March | 759 | 0–0 | 0.00–0.00 | 0–28 | 0.00–3.51 |  |  |
|  | April | 725 | 0–0 | 0.00–0.00 | 0–47 | 0.00–6.02 |  |  |
|  | May | 683 | 0–0 | 0.00–0.00 | 40–94 | 5.15–12.03 |  | ** |
|  | June | 766 | 0–0 | 0.00–0.00 | 0–12 | 0.00–1.44 |  |  |
|  | (2) Average number of hospitalized patients per day |  |  |  |  |  |  |  |
|  | (b) Psychiatric care beds |  |  |  |  |  |  |  |
|  | January | 3443 | 0–0 | 0.00–0.00 | 0–79 | 0.00–2.23 |  |  |
|  | February | 3440 | 0–0 | 0.00–0.00 | 0–83 | 0.00–2.33 |  |  |
|  | March | 3441 | 0–0 | 0.00–0.00 | 0–83 | 0.00–2.34 |  |  |
|  | April | 3423 | 0–0 | 0.00–0.00 | 0–98 | 0.00–2.77 |  |  |
|  | May | 3423 | 0–0 | 0.00–0.00 | 0–99 | 0.00–2.79 |  |  |
|  | June | 3450 | 0–0 | 0.00–0.00 | 0–73 | 0.00–2.07 |  |  |
|  | (c) Tuberculosis care beds |  |  |  |  |  |  |  |
|  | January | 19 | 0–0 | 0.00–0.00 | 0–2 | 0.00–9.21 |  |  |
|  | February | 20 | 0–0 | 0.00–0.00 | 0–1 | 0.00–2.52 |  |  |
|  | March | 21 | 0–0 | 0.00–0.00 | 0–1 | 0.00–0.86 |  |  |
|  | April | 23 | 0–1 | 0.00–3.29 | 0–0 | 0.00–0.00 |  |  |
|  | May | 19 | 0–0 | 0.00–0.00 | 0–5 | 0.00–17.50 |  |  |
|  | June | 21 | 0–0 | 0.00–0.00 | 0–2 | 0.00–7.60 |  |  |
|  | (d) Long-term care beds |  |  |  |  |  |  |  |
|  | January | 2419 | 0–0 | 0.00–0.00 | 0–57 | 0.00–2.30 |  |  |
|  | February | 2437 | 0–0 | 0.00–0.00 | 0–56 | 0.00–2.22 |  |  |
|  | March | 2430 | 0–0 | 0.00–0.00 | 0–59 | 0.00–2.37 |  |  |
|  | April | 2377 | 0–0 | 0.00–0.00 | 0–90 | 0.00–3.61 |  |  |
|  | May | 2341 | 0–0 | 0.00–0.00 | 3–100 | 0.12–4.07 |  | ** |
|  | June | 2341 | 0–0 | 0.00–0.00 | 0–87 | 0.00–3.57 |  |  |
|  | (e) General beds |  |  |  |  |  |  |  |
|  | January | 9069 | 0–0 | 0.00–0.00 | 0–389 | 0.00–4.11 |  |  |
|  | February | 9179 | 0–0 | 0.00–0.00 | 0–331 | 0.00–3.47 |  |  |
|  | March | 8701 | 0–0 | 0.00–0.00 | 312–691 | 3.32–7.36 |  | ** |
|  | April | 8046 | 0–0 | 0.00–0.00 | 639–1046 | 7.03–11.50 |  | ** |
|  | May | 7639 | 0–0 | 0.00–0.00 | 946–1258 | 10.63–14.13 |  | ** |
|  | June | 7906 | 0–0 | 0.00–0.00 | 578–923 | 6.55–10.45 |  | ** |
|  | (f) LTCI care beds |  |  |  |  |  |  |  |
|  | January | 259 | 0–26 | 0.00–11.13 | 0–0 | 0.00–0.00 |  |  |
|  | February | 260 | 0–29 | 0.00–12.15 | 0–0 | 0.00–0.00 |  |  |
|  | March | 264 | 6–37 | 2.64–16.01 | 0–0 | 0.00–0.00 | * |  |
|  | April | 256 | 2–33 | 0.90–14.70 | 0–0 | 0.00–0.00 | * |  |
|  | May | 251 | 0–31 | 0.00–13.69 | 0–0 | 0.00–0.00 |  |  |
|  | June | 246 | 0–23 | 0.00–10.08 | 0–0 | 0.00–0.00 |  |  |
|  | (3) Average length of hospital stays per patient |  |  |  |  |  |  |  |
|  | (b) Psychiatric care beds |  |  |  |  |  |  |  |
|  | January | 281 | 0–11 | 0.00–3.89 | 0–0 | 0.00–0.00 |  |  |
|  | February | 256 | 0–0 | 0.00–0.00 | 0–12 | 0.00–4.24 |  |  |
|  | March | 214 | 0–0 | 0.00–0.00 | 16–47 | 5.85–17.73 |  | ** |
|  | April | 285 | 0–23 | 0.00–8.49 | 0–0 | 0.00–0.00 |  |  |
|  | May | 288 | 0–27 | 0.00–10.22 | 0–0 | 0.00–0.00 |  |  |
|  | June | 276 | 0–15 | 0.00–5.36 | 0–0 | 0.00–0.00 |  |  |
|  | (c) Tuberculosis care beds |  |  |  |  |  |  |  |
|  | January | 74 | 0–16 | 0.00–27.55 | 0–0 | 0.00–0.00 |  |  |
|  | February | 65 | 0–11 | 0.00–19.62 | 0–0 | 0.00–0.00 |  |  |
|  | March | 69 | 0–8 | 0.00–12.40 | 0–0 | 0.00–0.00 |  |  |
|  | April | 34 | 0–0 | 0.00–0.00 | 4–28 | 5.02–45.05 |  | ** |
|  | May | 57 | 0–0 | 0.00–0.00 | 0–2 | 0.00–2.47 |  |  |
|  | June | 33 | 0–0 | 0.00–0.00 | 1–25 | 0.88–42.72 |  | ** |
|  | (d) Long-term care beds |  |  |  |  |  |  |  |
|  | January | 121 | 0–11 | 0.00–9.34 | 0–0 | 0.00–0.00 |  |  |
|  | February | 116 | 0–6 | 0.00–5.28 | 0–0 | 0.00–0.00 |  |  |
|  | March | 119 | 0–10 | 0.00–8.74 | 0–0 | 0.00–0.00 |  |  |
|  | April | 132 | 0–19 | 0.00–16.25 | 0–0 | 0.00–0.00 |  |  |
|  | May | 150 | 14–36 | 11.61–30.35 | 0–0 | 0.00–0.00 | * |  |
|  | June | 136 | 0–20 | 0.00–16.24 | 0–0 | 0.00–0.00 |  |  |
|  | (e) General beds |  |  |  |  |  |  |  |
|  | January | 16 | 0–1 | 0.00–1.66 | 0–0 | 0.00–0.00 |  |  |
|  | February | 16 | 0–0 | 0.00–0.00 | 0–1 | 0.00–2.14 |  |  |
|  | March | 15 | 0–0 | 0.00–0.00 | 0–1 | 0.00–2.59 |  |  |
|  | April | 16 | 0–1 | 0.00–4.21 | 0–0 | 0.00–0.00 |  |  |
|  | May | 17 | 0–2 | 0.00–11.28 | 0–0 | 0.00–0.00 |  |  |
|  | June | 15 | 0–1 | 0.00–0.46 | 0–0 | 0.00–0.00 |  |  |
|  | (f) LTCI care beds |  |  |  |  |  |  |  |
|  | January | 201 | 0–0 | 0.00–0.00 | 0–40 | 0.00–16.29 |  |  |
|  | February | 209 | 0–0 | 0.00–0.00 | 0–24 | 0.00–10.20 |  |  |
|  | March | 252 | 0–21 | 0.00–8.76 | 0–0 | 0.00–0.00 |  |  |
|  | April | 307 | 9–78 | 3.57–33.59 | 0–0 | 0.00–0.00 | * |  |
|  | May | 278 | 0–39 | 0.00–16.15 | 0–0 | 0.00–0.00 |  |  |
|  | June | 259 | 0–8 | 0.00–2.81 | 0–0 | 0.00–0.00 |  |  |
| Shizuoka | (1) Average number of outpatients per day at hospitals |  |  |  |  |  |  |  |
|  | General hospitals |  |  |  |  |  |  |  |
|  | January | 27742 | 0–0 | 0.00–0.00 | 0–881 | 0.00–3.08 |  |  |
|  | February | 27935 | 0–0 | 0.00–0.00 | 0–1072 | 0.00–3.69 |  |  |
|  | March | 27797 | 0–0 | 0.00–0.00 | 0–1249 | 0.00–4.30 |  |  |
|  | April | 25460 | 0–0 | 0.00–0.00 | 1174–2979 | 4.13–10.47 |  | ** |
|  | May | 22250 | 0–0 | 0.00–0.00 | 4458–6403 | 15.56–22.35 |  | ** |
|  | June | 27401 | 0–0 | 0.00–0.00 | 0–1629 | 0.00–5.61 |  |  |
|  | Psychiatric hospitals |  |  |  |  |  |  |  |
|  | January | 1354 | 0–0 | 0.00–0.00 | 0–82 | 0.00–5.66 |  |  |
|  | February | 1423 | 0–0 | 0.00–0.00 | 0–37 | 0.00–2.48 |  |  |
|  | March | 1438 | 0–0 | 0.00–0.00 | 0–55 | 0.00–3.64 |  |  |
|  | April | 1427 | 0–0 | 0.00–0.00 | 0–37 | 0.00–2.50 |  |  |
|  | May | 1272 | 0–0 | 0.00–0.00 | 108–202 | 7.33–13.68 |  | ** |
|  | June | 1443 | 0–0 | 0.00–0.00 | 0–32 | 0.00–2.16 |  |  |
|  | (2) Average number of hospitalized patients per day |  |  |  |  |  |  |  |
|  | (b) Psychiatric care beds |  |  |  |  |  |  |  |
|  | January | 5362 | 0–31 | 0.00–0.58 | 0–0 | 0.00–0.00 |  |  |
|  | February | 5322 | 0–0 | 0.00–0.00 | 0–11 | 0.00–0.20 |  |  |
|  | March | 5310 | 0–0 | 0.00–0.00 | 0–28 | 0.00–0.51 |  |  |
|  | April | 5259 | 0–0 | 0.00–0.00 | 0–87 | 0.00–1.61 |  |  |
|  | May | 5224 | 0–0 | 0.00–0.00 | 0–129 | 0.00–2.40 |  |  |
|  | June | 5310 | 0–0 | 0.00–0.00 | 0–55 | 0.00–1.01 |  |  |
|  | (c) Tuberculosis care beds |  |  |  |  |  |  |  |
|  | January | 20 | 0–0 | 0.00–0.00 | 0–10 | 0.00–32.20 |  |  |
|  | February | 23 | 0–0 | 0.00–0.00 | 0–4 | 0.00–14.74 |  |  |
|  | March | 21 | 0–0 | 0.00–0.00 | 0–6 | 0.00–20.67 |  |  |
|  | April | 22 | 0–0 | 0.00–0.00 | 0–6 | 0.00–18.73 |  |  |
|  | May | 21 | 0–0 | 0.00–0.00 | 0–7 | 0.00–24.80 |  |  |
|  | June | 23 | 0–0 | 0.00–0.00 | 0–6 | 0.00–19.16 |  |  |
|  | (d) Long-term care beds |  |  |  |  |  |  |  |
|  | January | 8237 | 0–0 | 0.00–0.00 | 304–755 | 3.38–8.39 |  | ** |
|  | February | 8333 | 0–0 | 0.00–0.00 | 175–641 | 1.95–7.14 |  | ** |
|  | March | 8330 | 0–0 | 0.00–0.00 | 145–609 | 1.62–6.81 |  | ** |
|  | April | 7702 | 0–0 | 0.00–0.00 | 690–1158 | 7.79–13.06 |  | ** |
|  | May | 7599 | 0–0 | 0.00–0.00 | 678–1156 | 7.74–13.20 |  | ** |
|  | June | 7594 | 0–0 | 0.00–0.00 | 572–1059 | 6.61–12.23 |  | ** |
|  | (e) General beds |  |  |  |  |  |  |  |
|  | January | 15773 | 0–0 | 0.00–0.00 | 0–470 | 0.00–2.89 |  |  |
|  | February | 16136 | 0–0 | 0.00–0.00 | 0–336 | 0.00–2.04 |  |  |
|  | March | 15576 | 0–0 | 0.00–0.00 | 205–813 | 1.25–4.96 |  | ** |
|  | April | 14459 | 0–0 | 0.00–0.00 | 743–1462 | 4.67–9.18 |  | ** |
|  | May | 13564 | 0–0 | 0.00–0.00 | 1604–2118 | 10.23–13.50 |  | ** |
|  | June | 14099 | 0–0 | 0.00–0.00 | 920–1457 | 5.91–9.36 |  | ** |
|  | (f) LTCI care beds |  |  |  |  |  |  |  |
|  | January | 901 | 0–0 | 0.00–0.00 | 143–309 | 11.83–25.48 |  | ** |
|  | February | 913 | 0–0 | 0.00–0.00 | 109–278 | 9.15–23.33 |  | ** |
|  | March | 913 | 0–0 | 0.00–0.00 | 92–263 | 7.83–22.34 |  | ** |
|  | April | 395 | 0–0 | 0.00–0.00 | 594–765 | 51.24–65.93 |  | ** |
|  | May | 401 | 0–0 | 0.00–0.00 | 571–742 | 49.97–64.91 |  | ** |
|  | June | 403 | 0–0 | 0.00–0.00 | 503–692 | 45.95–63.18 |  | ** |
|  | (3) Average length of hospital stays per patient |  |  |  |  |  |  |  |
|  | (b) Psychiatric care beds |  |  |  |  |  |  |  |
|  | January | 281 | 0–26 | 0.00–10.04 | 0–0 | 0.00–0.00 |  |  |
|  | February | 259 | 0–10 | 0.00–3.96 | 0–0 | 0.00–0.00 |  |  |
|  | March | 255 | 0–13 | 0.00–5.24 | 0–0 | 0.00–0.00 |  |  |
|  | April | 263 | 0–20 | 0.00–7.93 | 0–0 | 0.00–0.00 |  |  |
|  | May | 261 | 0–16 | 0.00–6.33 | 0–0 | 0.00–0.00 |  |  |
|  | June | 244 | 0–1 | 0.00–0.33 | 0–0 | 0.00–0.00 |  |  |
|  | (c) Tuberculosis care beds |  |  |  |  |  |  |  |
|  | January | 58 | 0–0 | 0.00–0.00 | 0–8 | 0.00–10.98 |  |  |
|  | February | 55 | 0–0 | 0.00–0.00 | 0–8 | 0.00–11.45 |  |  |
|  | March | 65 | 0–9 | 0.00–15.20 | 0–0 | 0.00–0.00 |  |  |
|  | April | 47 | 0–0 | 0.00–0.00 | 0–9 | 0.00–15.19 |  |  |
|  | May | 59 | 0–6 | 0.00–9.30 | 0–0 | 0.00–0.00 |  |  |
|  | June | 56 | 0–0 | 0.00–0.00 | 0–3 | 0.00–4.92 |  |  |
|  | (d) Long-term care beds |  |  |  |  |  |  |  |
|  | January | 151 | 0–13 | 0.00–9.26 | 0–0 | 0.00–0.00 |  |  |
|  | February | 141 | 0–4 | 0.00–2.65 | 0–0 | 0.00–0.00 |  |  |
|  | March | 124 | 0–0 | 0.00–0.00 | 0–11 | 0.00–7.45 |  |  |
|  | April | 133 | 0–0 | 0.00–0.00 | 0–6 | 0.00–3.98 |  |  |
|  | May | 153 | 0–13 | 0.00–8.58 | 0–0 | 0.00–0.00 |  |  |
|  | June | 138 | 0–0 | 0.00–0.00 | 0–5 | 0.00–3.09 |  |  |
|  | (e) General beds |  |  |  |  |  |  |  |
|  | January | 16 | 0–1 | 0.00–2.57 | 0–0 | 0.00–0.00 |  |  |
|  | February | 16 | 0–1 | 0.00–0.37 | 0–0 | 0.00–0.00 |  |  |
|  | March | 15 | 0–1 | 0.00–0.15 | 0–0 | 0.00–0.00 |  |  |
|  | April | 16 | 0–1 | 0.00–2.16 | 0–0 | 0.00–0.00 |  |  |
|  | May | 17 | 0–2 | 0.00–11.97 | 0–0 | 0.00–0.00 |  |  |
|  | June | 15 | 0–0 | 0.00–0.00 | 0–1 | 0.00–0.41 |  |  |
|  | (f) LTCI care beds |  |  |  |  |  |  |  |
|  | January | 338 | 0–57 | 0.00–20.06 | 0–0 | 0.00–0.00 |  |  |
|  | February | 335 | 0–58 | 0.00–20.70 | 0–0 | 0.00–0.00 |  |  |
|  | March | 88 | 0–0 | 0.00–0.00 | 128–178 | 48.07–67.01 |  | ** |
|  | April | 185 | 0–0 | 0.00–0.00 | 41–94 | 14.59–33.57 |  | ** |
|  | May | 311 | 0–28 | 0.00–9.61 | 0–0 | 0.00–0.00 |  |  |
|  | June | 269 | 0–0 | 0.00–0.00 | 0–10 | 0.00–3.31 |  |  |
| Aichi | (1) Average number of outpatients per day at hospitals |  |  |  |  |  |  |  |
|  | General hospitals |  |  |  |  |  |  |  |
|  | January | 61090 | 0–0 | 0.00–0.00 | 0–1374 | 0.00–2.20 |  |  |
|  | February | 60240 | 0–0 | 0.00–0.00 | 0–2948 | 0.00–4.66 |  |  |
|  | March | 57920 | 0–0 | 0.00–0.00 | 1376–5693 | 2.16–8.95 |  | ** |
|  | April | 52416 | 0–0 | 0.00–0.00 | 5369–9416 | 8.68–15.23 |  | ** |
|  | May | 46986 | 0–0 | 0.00–0.00 | 10935–15113 | 17.61–24.34 |  | ** |
|  | June | 59129 | 0–0 | 0.00–0.00 | 0–3706 | 0.00–5.90 |  |  |
|  | Psychiatric hospitals |  |  |  |  |  |  |  |
|  | January | 2550 | 0–0 | 0.00–0.00 | 0–146 | 0.00–5.40 |  |  |
|  | February | 2672 | 0–0 | 0.00–0.00 | 0–74 | 0.00–2.66 |  |  |
|  | March | 2571 | 0–0 | 0.00–0.00 | 73–255 | 2.58–9.02 |  | ** |
|  | April | 2551 | 0–0 | 0.00–0.00 | 26–213 | 0.94–7.70 |  | ** |
|  | May | 2246 | 0–0 | 0.00–0.00 | 327–532 | 11.77–19.13 |  | ** |
|  | June | 2693 | 0–0 | 0.00–0.00 | 0–91 | 0.00–3.26 |  |  |
|  | (2) Average number of hospitalized patients per day |  |  |  |  |  |  |  |
|  | (b) Psychiatric care beds |  |  |  |  |  |  |  |
|  | January | 11008 | 0–0 | 0.00–0.00 | 0–44 | 0.00–0.40 |  |  |
|  | February | 11035 | 0–0 | 0.00–0.00 | 0–28 | 0.00–0.25 |  |  |
|  | March | 10892 | 0–0 | 0.00–0.00 | 0–194 | 0.00–1.74 |  |  |
|  | April | 10790 | 0–0 | 0.00–0.00 | 95–301 | 0.86–2.71 |  | ** |
|  | May | 10738 | 0–0 | 0.00–0.00 | 172–378 | 1.55–3.40 |  | ** |
|  | June | 10923 | 0–0 | 0.00–0.00 | 22–229 | 0.20–2.05 |  | ** |
|  | (c) Tuberculosis care beds |  |  |  |  |  |  |  |
|  | January | 69 | 0–4 | 0.00–5.91 | 0–0 | 0.00–0.00 |  |  |
|  | February | 73 | 0–10 | 0.00–15.45 | 0–0 | 0.00–0.00 |  |  |
|  | March | 72 | 0–9 | 0.00–13.50 | 0–0 | 0.00–0.00 |  |  |
|  | April | 70 | 0–6 | 0.00–9.14 | 0–0 | 0.00–0.00 |  |  |
|  | May | 67 | 0–2 | 0.00–1.95 | 0–0 | 0.00–0.00 |  |  |
|  | June | 68 | 0–2 | 0.00–1.97 | 0–0 | 0.00–0.00 |  |  |
|  | (d) Long-term care beds |  |  |  |  |  |  |  |
|  | January | 12411 | 0–0 | 0.00–0.00 | 193–535 | 1.49–4.13 |  | ** |
|  | February | 12529 | 0–0 | 0.00–0.00 | 0–356 | 0.00–2.76 |  |  |
|  | March | 12531 | 0–0 | 0.00–0.00 | 0–331 | 0.00–2.57 |  |  |
|  | April | 11878 | 0–0 | 0.00–0.00 | 439–861 | 3.45–6.76 |  | ** |
|  | May | 11664 | 0–0 | 0.00–0.00 | 542–962 | 4.29–7.61 |  | ** |
|  | June | 11714 | 0–0 | 0.00–0.00 | 405–816 | 3.23–6.51 |  | ** |
|  | (e) General beds |  |  |  |  |  |  |  |
|  | January | 30680 | 0–0 | 0.00–0.00 | 0–609 | 0.00–1.95 |  |  |
|  | February | 31152 | 0–0 | 0.00–0.00 | 0–577 | 0.00–1.82 |  |  |
|  | March | 29668 | 0–0 | 0.00–0.00 | 1039–2013 | 3.28–6.35 |  | ** |
|  | April | 27579 | 0–0 | 0.00–0.00 | 2048–3279 | 6.64–10.63 |  | ** |
|  | May | 25851 | 0–0 | 0.00–0.00 | 3659–4535 | 12.04–14.92 |  | ** |
|  | June | 26838 | 0–0 | 0.00–0.00 | 2536–3392 | 8.39–11.22 |  | ** |
|  | (f) LTCI care beds |  |  |  |  |  |  |  |
|  | January | 952 | 0–0 | 0.00–0.00 | 323–464 | 22.81–32.76 |  | ** |
|  | February | 955 | 0–0 | 0.00–0.00 | 295–450 | 21.01–31.98 |  | ** |
|  | March | 949 | 0–0 | 0.00–0.00 | 111–355 | 8.51–27.21 |  | ** |
|  | April | 464 | 0–0 | 0.00–0.00 | 446–722 | 37.63–60.85 |  | ** |
|  | May | 463 | 0–0 | 0.00–0.00 | 392–669 | 34.65–59.07 |  | ** |
|  | June | 463 | 0–0 | 0.00–0.00 | 358–640 | 32.48–57.99 |  | ** |
|  | (3) Average length of hospital stays per patient |  |  |  |  |  |  |  |
|  | (b) Psychiatric care beds |  |  |  |  |  |  |  |
|  | January | 251 | 0–3 | 0.00–1.14 | 0–0 | 0.00–0.00 |  |  |
|  | February | 256 | 0–11 | 0.00–4.20 | 0–0 | 0.00–0.00 |  |  |
|  | March | 248 | 0–9 | 0.00–3.72 | 0–0 | 0.00–0.00 |  |  |
|  | April | 259 | 0–20 | 0.00–8.01 | 0–0 | 0.00–0.00 |  |  |
|  | May | 275 | 11–42 | 4.32–17.72 | 0–0 | 0.00–0.00 | * |  |
|  | June | 238 | 0–5 | 0.00–1.83 | 0–0 | 0.00–0.00 |  |  |
|  | (c) Tuberculosis care beds |  |  |  |  |  |  |  |
|  | January | 75 | 0–13 | 0.00–19.93 | 0–0 | 0.00–0.00 |  |  |
|  | February | 67 | 0–6 | 0.00–9.53 | 0–0 | 0.00–0.00 |  |  |
|  | March | 52 | 0–0 | 0.00–0.00 | 0–9 | 0.00–14.71 |  |  |
|  | April | 52 | 0–0 | 0.00–0.00 | 0–11 | 0.00–16.35 |  |  |
|  | May | 82 | 0–16 | 0.00–23.07 | 0–0 | 0.00–0.00 |  |  |
|  | June | 75 | 0–11 | 0.00–15.32 | 0–0 | 0.00–0.00 |  |  |
|  | (d) Long-term care beds |  |  |  |  |  |  |  |
|  | January | 123 | 0–2 | 0.00–1.10 | 0–0 | 0.00–0.00 |  |  |
|  | February | 120 | 0–1 | 0.00–0.33 | 0–0 | 0.00–0.00 |  |  |
|  | March | 117 | 0–0 | 0.00–0.00 | 0–1 | 0.00–0.75 |  |  |
|  | April | 119 | 0–0 | 0.00–0.00 | 0–3 | 0.00–1.80 |  |  |
|  | May | 138 | 0–17 | 0.00–13.43 | 0–0 | 0.00–0.00 |  |  |
|  | June | 122 | 0–0 | 0.00–0.00 | 0–2 | 0.00–1.44 |  |  |
|  | (e) General beds |  |  |  |  |  |  |  |
|  | January | 14 | 0–1 | 0.00–3.27 | 0–0 | 0.00–0.00 |  |  |
|  | February | 14 | 0–1 | 0.00–0.50 | 0–0 | 0.00–0.00 |  |  |
|  | March | 14 | 0–1 | 0.00–2.20 | 0–0 | 0.00–0.00 |  |  |
|  | April | 15 | 0–1 | 0.00–4.51 | 0–0 | 0.00–0.00 |  |  |
|  | May | 16 | 0–3 | 0.00–14.71 | 0–0 | 0.00–0.00 |  |  |
|  | June | 14 | 0–1 | 0.00–2.65 | 0–0 | 0.00–0.00 |  |  |
|  | (f) LTCI care beds |  |  |  |  |  |  |  |
|  | January | 381 | 17–95 | 5.84–33.02 | 0–0 | 0.00–0.00 | * |  |
|  | February | 390 | 13–96 | 4.38–32.37 | 0–0 | 0.00–0.00 | * |  |
|  | March | 135 | 0–0 | 0.00–0.00 | 90–164 | 29.96–54.80 |  | ** |
|  | April | 480 | 64–158 | 19.82–49.04 | 0–0 | 0.00–0.00 | * |  |
|  | May | 478 | 39–136 | 11.11–39.43 | 0–0 | 0.00–0.00 | * |  |
|  | June | 603 | 134–233 | 36.00–62.91 | 0–0 | 0.00–0.00 | * |  |
| Mie | (1) Average number of outpatients per day at hospitals |  |  |  |  |  |  |  |
|  | General hospitals |  |  |  |  |  |  |  |
|  | January | 14841 | 0–0 | 0.00–0.00 | 0–510 | 0.00–3.32 |  |  |
|  | February | 14669 | 0–0 | 0.00–0.00 | 0–678 | 0.00–4.41 |  |  |
|  | March | 14399 | 0–0 | 0.00–0.00 | 0–1083 | 0.00–6.99 |  |  |
|  | April | 13305 | 0–0 | 0.00–0.00 | 937–1844 | 6.19–12.17 |  | ** |
|  | May | 11591 | 0–0 | 0.00–0.00 | 2660–3699 | 17.40–24.19 |  | ** |
|  | June | 14621 | 0–0 | 0.00–0.00 | 0–868 | 0.00–5.60 |  |  |
|  | Psychiatric hospitals |  |  |  |  |  |  |  |
|  | January | 955 | 0–0 | 0.00–0.00 | 0–93 | 0.00–8.82 |  |  |
|  | February | 980 | 0–0 | 0.00–0.00 | 0–85 | 0.00–7.90 |  |  |
|  | March | 1000 | 0–0 | 0.00–0.00 | 0–92 | 0.00–8.42 |  |  |
|  | April | 983 | 0–0 | 0.00–0.00 | 0–83 | 0.00–7.71 |  |  |
|  | May | 864 | 0–0 | 0.00–0.00 | 95–202 | 8.91–18.93 |  | ** |
|  | June | 1033 | 0–0 | 0.00–0.00 | 0–32 | 0.00–2.97 |  |  |
|  | (2) Average number of hospitalized patients per day |  |  |  |  |  |  |  |
|  | (b) Psychiatric care beds |  |  |  |  |  |  |  |
|  | January | 4039 | 0–0 | 0.00–0.00 | 0–17 | 0.00–0.40 |  |  |
|  | February | 4058 | 0–0 | 0.00–0.00 | 0–7 | 0.00–0.16 |  |  |
|  | March | 4001 | 0–0 | 0.00–0.00 | 0–61 | 0.00–1.48 |  |  |
|  | April | 3962 | 0–0 | 0.00–0.00 | 0–86 | 0.00–2.11 |  |  |
|  | May | 3933 | 0–0 | 0.00–0.00 | 0–107 | 0.00–2.63 |  |  |
|  | June | 3940 | 0–0 | 0.00–0.00 | 0–105 | 0.00–2.58 |  |  |
|  | (c) Tuberculosis care beds |  |  |  |  |  |  |  |
|  | January | 13 | 0–1 | 0.00–1.49 | 0–0 | 0.00–0.00 |  |  |
|  | February | 12 | 0–0 | 0.00–0.00 | 0–1 | 0.00–5.61 |  |  |
|  | March | 10 | 0–0 | 0.00–0.00 | 0–3 | 0.00–21.53 |  |  |
|  | April | 13 | 0–0 | 0.00–0.00 | 0–2 | 0.00–9.77 |  |  |
|  | May | 16 | 0–1 | 0.00–1.17 | 0–0 | 0.00–0.00 |  |  |
|  | June | 13 | 0–0 | 0.00–0.00 | 0–4 | 0.00–23.12 |  |  |
|  | (d) Long-term care beds |  |  |  |  |  |  |  |
|  | January | 3326 | 0–0 | 0.00–0.00 | 0–33 | 0.00–0.97 |  |  |
|  | February | 3404 | 0–45 | 0.00–1.33 | 0–0 | 0.00–0.00 |  |  |
|  | March | 3385 | 0–23 | 0.00–0.68 | 0–0 | 0.00–0.00 |  |  |
|  | April | 3244 | 0–0 | 0.00–0.00 | 0–100 | 0.00–2.99 |  |  |
|  | May | 3158 | 0–0 | 0.00–0.00 | 58–171 | 1.74–5.11 |  | ** |
|  | June | 3153 | 0–0 | 0.00–0.00 | 58–171 | 1.75–5.12 |  | ** |
|  | (e) General beds |  |  |  |  |  |  |  |
|  | January | 8259 | 0–0 | 0.00–0.00 | 0–242 | 0.00–2.84 |  |  |
|  | February | 8442 | 0–0 | 0.00–0.00 | 0–152 | 0.00–1.76 |  |  |
|  | March | 8077 | 0–0 | 0.00–0.00 | 209–467 | 2.45–5.46 |  | ** |
|  | April | 7507 | 0–0 | 0.00–0.00 | 513–837 | 6.15–10.03 |  | ** |
|  | May | 7122 | 0–0 | 0.00–0.00 | 882–1109 | 10.72–13.47 |  | ** |
|  | June | 7396 | 0–0 | 0.00–0.00 | 541–798 | 6.60–9.73 |  | ** |
|  | (f) LTCI care beds |  |  |  |  |  |  |  |
|  | January | 267 | 0–44 | 0.00–19.29 | 0–0 | 0.00–0.00 |  |  |
|  | February | 265 | 0–47 | 0.00–21.30 | 0–0 | 0.00–0.00 |  |  |
|  | March | 272 | 0–60 | 0.00–27.94 | 0–0 | 0.00–0.00 |  |  |
|  | April | 164 | 0–0 | 0.00–0.00 | 0–47 | 0.00–22.18 |  |  |
|  | May | 161 | 0–0 | 0.00–0.00 | 0–47 | 0.00–22.58 |  |  |
|  | June | 159 | 0–0 | 0.00–0.00 | 0–49 | 0.00–23.45 |  |  |
|  | (3) Average length of hospital stays per patient |  |  |  |  |  |  |  |
|  | (b) Psychiatric care beds |  |  |  |  |  |  |  |
|  | January | 329 | 0–19 | 0.00–5.79 | 0–0 | 0.00–0.00 |  |  |
|  | February | 308 | 0–7 | 0.00–2.32 | 0–0 | 0.00–0.00 |  |  |
|  | March | 320 | 0–24 | 0.00–7.86 | 0–0 | 0.00–0.00 |  |  |
|  | April | 317 | 0–21 | 0.00–6.96 | 0–0 | 0.00–0.00 |  |  |
|  | May | 367 | 35–70 | 11.48–23.24 | 0–0 | 0.00–0.00 | * |  |
|  | June | 328 | 0–30 | 0.00–10.03 | 0–0 | 0.00–0.00 |  |  |
|  | (c) Tuberculosis care beds |  |  |  |  |  |  |  |
|  | January | 46 | 0–0 | 0.00–0.00 | 0–26 | 0.00–35.55 |  |  |
|  | February | 75 | 0–5 | 0.00–6.38 | 0–0 | 0.00–0.00 |  |  |
|  | March | 91 | 0–21 | 0.00–28.45 | 0–0 | 0.00–0.00 |  |  |
|  | April | 65 | 0–0 | 0.00–0.00 | 0–16 | 0.00–18.90 |  |  |
|  | May | 85 | 0–7 | 0.00–8.10 | 0–0 | 0.00–0.00 |  |  |
|  | June | 96 | 0–25 | 0.00–35.29 | 0–0 | 0.00–0.00 |  |  |
|  | (d) Long-term care beds |  |  |  |  |  |  |  |
|  | January | 120 | 0–7 | 0.00–5.64 | 0–0 | 0.00–0.00 |  |  |
|  | February | 116 | 0–3 | 0.00–2.05 | 0–0 | 0.00–0.00 |  |  |
|  | March | 107 | 0–0 | 0.00–0.00 | 0–4 | 0.00–2.91 |  |  |
|  | April | 117 | 0–4 | 0.00–2.94 | 0–0 | 0.00–0.00 |  |  |
|  | May | 129 | 0–16 | 0.00–13.84 | 0–0 | 0.00–0.00 |  |  |
|  | June | 112 | 0–0 | 0.00–0.00 | 0–3 | 0.00–2.54 |  |  |
|  | (e) General beds |  |  |  |  |  |  |  |
|  | January | 16 | 0–1 | 0.00–3.14 | 0–0 | 0.00–0.00 |  |  |
|  | February | 16 | 0–1 | 0.00–1.14 | 0–0 | 0.00–0.00 |  |  |
|  | March | 16 | 0–1 | 0.00–1.62 | 0–0 | 0.00–0.00 |  |  |
|  | April | 16 | 0–1 | 0.00–1.88 | 0–0 | 0.00–0.00 |  |  |
|  | May | 18 | 0–3 | 0.00–15.93 | 0–0 | 0.00–0.00 |  |  |
|  | June | 15 | 0–0 | 0.00–0.00 | 0–1 | 0.00–0.95 |  |  |
|  | (f) LTCI care beds |  |  |  |  |  |  |  |
|  | January | 572 | 0–145 | 0.00–33.95 | 0–0 | 0.00–0.00 |  |  |
|  | February | 365 | 0–0 | 0.00–0.00 | 0–38 | 0.00–9.35 |  |  |
|  | March | 102 | 0–0 | 0.00–0.00 | 143–252 | 40.42–71.12 |  | ** |
|  | April | 758 | 225–375 | 58.37–97.35 | 0–0 | 0.00–0.00 | * |  |
|  | May | 370 | 0–0 | 0.00–0.00 | 0–10 | 0.00–2.52 |  |  |
|  | June | 272 | 0–0 | 0.00–0.00 | 12–127 | 2.83–31.84 |  | ** |
| Shiga | (1) Average number of outpatients per day at hospitals |  |  |  |  |  |  |  |
|  | General hospitals |  |  |  |  |  |  |  |
|  | January | 12367 | 0–0 | 0.00–0.00 | 0–71 | 0.00–0.57 |  |  |
|  | February | 12184 | 0–0 | 0.00–0.00 | 0–459 | 0.00–3.62 |  |  |
|  | March | 12159 | 0–0 | 0.00–0.00 | 0–637 | 0.00–4.97 |  |  |
|  | April | 10984 | 0–0 | 0.00–0.00 | 665–1508 | 5.32–12.07 |  | ** |
|  | May | 9273 | 0–0 | 0.00–0.00 | 2414–3321 | 19.17–26.37 |  | ** |
|  | June | 11726 | 0–0 | 0.00–0.00 | 136–1031 | 1.07–8.08 |  | ** |
|  | Psychiatric hospitals |  |  |  |  |  |  |  |
|  | January | 373 | 0–0 | 0.00–0.00 | 0–5 | 0.00–1.22 |  |  |
|  | February | 379 | 0–0 | 0.00–0.00 | 0–8 | 0.00–1.93 |  |  |
|  | March | 375 | 0–0 | 0.00–0.00 | 0–22 | 0.00–5.49 |  |  |
|  | April | 368 | 0–0 | 0.00–0.00 | 0–21 | 0.00–5.35 |  |  |
|  | May | 327 | 0–0 | 0.00–0.00 | 23–61 | 5.93–15.69 |  | ** |
|  | June | 354 | 0–0 | 0.00–0.00 | 0–35 | 0.00–8.87 |  |  |
|  | (2) Average number of hospitalized patients per day |  |  |  |  |  |  |  |
|  | (b) Psychiatric care beds |  |  |  |  |  |  |  |
|  | January | 1920 | 0–0 | 0.00–0.00 | 0–51 | 0.00–2.58 |  |  |
|  | February | 1917 | 0–0 | 0.00–0.00 | 0–59 | 0.00–2.94 |  |  |
|  | March | 1905 | 0–0 | 0.00–0.00 | 0–74 | 0.00–3.71 |  |  |
|  | April | 1860 | 0–0 | 0.00–0.00 | 37–124 | 1.87–6.21 |  | ** |
|  | May | 1845 | 0–0 | 0.00–0.00 | 54–141 | 2.72–7.10 |  | ** |
|  | June | 1881 | 0–0 | 0.00–0.00 | 20–107 | 1.01–5.37 |  | ** |
|  | (c) Tuberculosis care beds |  |  |  |  |  |  |  |
|  | January | 10 | 0–0 | 0.00–0.00 | 0–2 | 0.00–10.20 |  |  |
|  | February | 12 | 0–2 | 0.00–14.99 | 0–0 | 0.00–0.00 |  |  |
|  | March | 12 | 0–2 | 0.00–16.33 | 0–0 | 0.00–0.00 |  |  |
|  | April | 12 | 0–1 | 0.00–7.03 | 0–0 | 0.00–0.00 |  |  |
|  | May | 12 | 0–1 | 0.00–2.03 | 0–0 | 0.00–0.00 |  |  |
|  | June | 10 | 0–0 | 0.00–0.00 | 0–2 | 0.00–15.34 |  |  |
|  | (d) Long-term care beds |  |  |  |  |  |  |  |
|  | January | 2371 | 0–0 | 0.00–0.00 | 24–121 | 0.96–4.85 |  | ** |
|  | February | 2393 | 0–0 | 0.00–0.00 | 1–99 | 0.04–3.95 |  | ** |
|  | March | 2397 | 0–0 | 0.00–0.00 | 0–86 | 0.00–3.45 |  |  |
|  | April | 2257 | 0–0 | 0.00–0.00 | 104–200 | 4.23–8.13 |  | ** |
|  | May | 2232 | 0–0 | 0.00–0.00 | 106–202 | 4.36–8.28 |  | ** |
|  | June | 2231 | 0–0 | 0.00–0.00 | 96–192 | 3.96–7.90 |  | ** |
|  | (e) General beds |  |  |  |  |  |  |  |
|  | January | 7027 | 0–0 | 0.00–0.00 | 0–219 | 0.00–3.01 |  |  |
|  | February | 7206 | 0–0 | 0.00–0.00 | 0–127 | 0.00–1.73 |  |  |
|  | March | 6976 | 0–0 | 0.00–0.00 | 104–342 | 1.42–4.67 |  | ** |
|  | April | 6366 | 0–0 | 0.00–0.00 | 490–777 | 6.86–10.88 |  | ** |
|  | May | 5805 | 0–0 | 0.00–0.00 | 1019–1250 | 14.44–17.71 |  | ** |
|  | June | 6221 | 0–0 | 0.00–0.00 | 597–823 | 8.48–11.68 |  | ** |
|  | (f) LTCI care beds |  |  |  |  |  |  |  |
|  | January | 184 | 0–0 | 0.00–0.00 | 117–152 | 34.87–45.17 |  | ** |
|  | February | 182 | 0–0 | 0.00–0.00 | 117–152 | 35.08–45.43 |  | ** |
|  | March | 179 | 0–0 | 0.00–0.00 | 117–152 | 35.44–45.78 |  | ** |
|  | April | 69 | 0–0 | 0.00–0.00 | 148–214 | 52.42–75.56 |  | ** |
|  | May | 69 | 0–0 | 0.00–0.00 | 108–180 | 43.44–72.24 |  | ** |
|  | June | 69 | 0–0 | 0.00–0.00 | 99–173 | 40.93–71.47 |  | ** |
|  | (3) Average length of hospital stays per patient |  |  |  |  |  |  |  |
|  | (b) Psychiatric care beds |  |  |  |  |  |  |  |
|  | January | 231 | 0–2 | 0.00–0.57 | 0–0 | 0.00–0.00 |  |  |
|  | February | 224 | 0–0 | 0.00–0.00 | 0–1 | 0.00–0.29 |  |  |
|  | March | 217 | 0–0 | 0.00–0.00 | 0–3 | 0.00–1.18 |  |  |
|  | April | 231 | 0–14 | 0.00–6.32 | 0–0 | 0.00–0.00 |  |  |
|  | May | 242 | 0–25 | 0.00–11.52 | 0–0 | 0.00–0.00 |  |  |
|  | June | 199 | 0–0 | 0.00–0.00 | 0–15 | 0.00–6.76 |  |  |
|  | (c) Tuberculosis care beds |  |  |  |  |  |  |  |
|  | January | 103 | 0–28 | 0.00–36.68 | 0–0 | 0.00–0.00 |  |  |
|  | February | 78 | 0–8 | 0.00–10.59 | 0–0 | 0.00–0.00 |  |  |
|  | March | 92 | 0–13 | 0.00–16.05 | 0–0 | 0.00–0.00 |  |  |
|  | April | 89 | 0–2 | 0.00–1.40 | 0–0 | 0.00–0.00 |  |  |
|  | May | 84 | 0–5 | 0.00–5.89 | 0–0 | 0.00–0.00 |  |  |
|  | June | 66 | 0–0 | 0.00–0.00 | 0–12 | 0.00–14.85 |  |  |
|  | (d) Long-term care beds |  |  |  |  |  |  |  |
|  | January | 145 | 0–0 | 0.00–0.00 | 0–7 | 0.00–4.39 |  |  |
|  | February | 153 | 0–2 | 0.00–1.27 | 0–0 | 0.00–0.00 |  |  |
|  | March | 149 | 0–2 | 0.00–0.94 | 0–0 | 0.00–0.00 |  |  |
|  | April | 133 | 0–0 | 0.00–0.00 | 0–19 | 0.00–12.07 |  |  |
|  | May | 189 | 14–39 | 8.99–25.49 | 0–0 | 0.00–0.00 | * |  |
|  | June | 159 | 0–9 | 0.00–5.51 | 0–0 | 0.00–0.00 |  |  |
|  | (e) General beds |  |  |  |  |  |  |  |
|  | January | 16 | 0–1 | 0.00–1.22 | 0–0 | 0.00–0.00 |  |  |
|  | February | 16 | 0–0 | 0.00–0.00 | 0–1 | 0.00–0.55 |  |  |
|  | March | 16 | 0–0 | 0.00–0.00 | 0–1 | 0.00–0.09 |  |  |
|  | April | 17 | 0–1 | 0.00–3.50 | 0–0 | 0.00–0.00 |  |  |
|  | May | 19 | 0–3 | 0.00–18.83 | 0–0 | 0.00–0.00 |  |  |
|  | June | 17 | 0–1 | 0.00–5.97 | 0–0 | 0.00–0.00 |  |  |
|  | (f) LTCI care beds |  |  |  |  |  |  |  |
|  | January | 760 | 215–354 | 52.62–86.82 | 0–0 | 0.00–0.00 | * |  |
|  | February | 557 | 2–147 | 0.37–35.86 | 0–0 | 0.00–0.00 | * |  |
|  | March | 444 | 0–42 | 0.00–10.36 | 0–0 | 0.00–0.00 |  |  |
|  | April | 30 | 0–0 | 0.00–0.00 | 290–438 | 61.98–93.56 |  | ** |
|  | May | 430 | 0–0 | 0.00–0.00 | 0–44 | 0.00–9.12 |  |  |
|  | June | 378 | 0–0 | 0.00–0.00 | 0–103 | 0.00–21.39 |  |  |
| Kyoto | (1) Average number of outpatients per day at hospitals |  |  |  |  |  |  |  |
|  | General hospitals |  |  |  |  |  |  |  |
|  | January | 28200 | 0–0 | 0.00–0.00 | 0–973 | 0.00–3.33 |  |  |
|  | February | 27871 | 0–0 | 0.00–0.00 | 0–1646 | 0.00–5.57 |  |  |
|  | March | 27488 | 0–0 | 0.00–0.00 | 269–2253 | 0.90–7.57 |  | ** |
|  | April | 24177 | 0–0 | 0.00–0.00 | 2921–4822 | 10.07–16.63 |  | ** |
|  | May | 21300 | 0–0 | 0.00–0.00 | 5882–7835 | 20.19–26.89 |  | ** |
|  | June | 26794 | 0–0 | 0.00–0.00 | 576–2563 | 1.96–8.73 |  | ** |
|  | Psychiatric hospitals |  |  |  |  |  |  |  |
|  | January | 514 | 0–35 | 0.00–7.16 | 0–0 | 0.00–0.00 |  |  |
|  | February | 543 | 15–60 | 3.10–12.32 | 0–0 | 0.00–0.00 | * |  |
|  | March | 529 | 0–23 | 0.00–4.37 | 0–0 | 0.00–0.00 |  |  |
|  | April | 499 | 0–0 | 0.00–0.00 | 0–6 | 0.00–1.08 |  |  |
|  | May | 415 | 0–0 | 0.00–0.00 | 54–99 | 10.51–19.21 |  | ** |
|  | June | 507 | 0–0 | 0.00–0.00 | 0–5 | 0.00–0.84 |  |  |
|  | (2) Average number of hospitalized patients per day |  |  |  |  |  |  |  |
|  | (b) Psychiatric care beds |  |  |  |  |  |  |  |
|  | January | 4688 | 0–24 | 0.00–0.50 | 0–0 | 0.00–0.00 |  |  |
|  | February | 4697 | 0–33 | 0.00–0.70 | 0–0 | 0.00–0.00 |  |  |
|  | March | 4623 | 0–0 | 0.00–0.00 | 0–33 | 0.00–0.71 |  |  |
|  | April | 4546 | 0–0 | 0.00–0.00 | 0–107 | 0.00–2.30 |  |  |
|  | May | 4503 | 0–0 | 0.00–0.00 | 19–152 | 0.41–3.25 |  | ** |
|  | June | 4527 | 0–0 | 0.00–0.00 | 13–147 | 0.28–3.13 |  | ** |
|  | (c) Tuberculosis care beds |  |  |  |  |  |  |  |
|  | January | 30 | 0–6 | 0.00–22.72 | 0–0 | 0.00–0.00 |  |  |
|  | February | 28 | 0–5 | 0.00–17.18 | 0–0 | 0.00–0.00 |  |  |
|  | March | 24 | 0–0 | 0.00–0.00 | 0–1 | 0.00–0.52 |  |  |
|  | April | 28 | 0–4 | 0.00–16.24 | 0–0 | 0.00–0.00 |  |  |
|  | May | 22 | 0–0 | 0.00–0.00 | 0–3 | 0.00–10.77 |  |  |
|  | June | 16 | 0–0 | 0.00–0.00 | 0–10 | 0.00–37.09 |  |  |
|  | (d) Long-term care beds |  |  |  |  |  |  |  |
|  | January | 4910 | 0–0 | 0.00–0.00 | 522–668 | 9.36–11.97 |  | ** |
|  | February | 4397 | 0–0 | 0.00–0.00 | 1035–1181 | 18.56–21.17 |  | ** |
|  | March | 3870 | 0–0 | 0.00–0.00 | 1555–1701 | 27.91–30.53 |  | ** |
|  | April | 3526 | 0–0 | 0.00–0.00 | 1880–2025 | 33.87–36.48 |  | ** |
|  | May | 3495 | 0–0 | 0.00–0.00 | 1892–2037 | 34.20–36.82 |  | ** |
|  | June | 3504 | 0–0 | 0.00–0.00 | 1836–1981 | 33.48–36.11 |  | ** |
|  | (e) General beds |  |  |  |  |  |  |  |
|  | January | 17584 | 0–0 | 0.00–0.00 | 0–299 | 0.00–1.67 |  |  |
|  | February | 17957 | 0–0 | 0.00–0.00 | 0–79 | 0.00–0.44 |  |  |
|  | March | 17144 | 0–0 | 0.00–0.00 | 333–851 | 1.85–4.73 |  | ** |
|  | April | 15871 | 0–0 | 0.00–0.00 | 1204–1769 | 6.83–10.03 |  | ** |
|  | May | 14893 | 0–0 | 0.00–0.00 | 2080–2504 | 11.96–14.39 |  | ** |
|  | June | 15584 | 0–0 | 0.00–0.00 | 1306–1769 | 7.53–10.19 |  | ** |
|  | (f) LTCI care beds |  |  |  |  |  |  |  |
|  | January | 1802 | 0–0 | 0.00–0.00 | 461–555 | 19.56–23.54 |  | ** |
|  | February | 1286 | 0–0 | 0.00–0.00 | 965–1059 | 41.15–45.16 |  | ** |
|  | March | 781 | 0–0 | 0.00–0.00 | 1465–1559 | 62.61–66.62 |  | ** |
|  | April | 506 | 0–0 | 0.00–0.00 | 1727–1821 | 74.23–78.25 |  | ** |
|  | May | 499 | 0–0 | 0.00–0.00 | 1711–1817 | 73.89–78.45 |  | ** |
|  | June | 510 | 0–0 | 0.00–0.00 | 1695–1803 | 73.31–77.94 |  | ** |
|  | (3) Average length of hospital stays per patient |  |  |  |  |  |  |  |
|  | (b) Psychiatric care beds |  |  |  |  |  |  |  |
|  | January | 234 | 0–0 | 0.00–0.00 | 0–12 | 0.00–4.70 |  |  |
|  | February | 237 | 0–0 | 0.00–0.00 | 0–8 | 0.00–2.88 |  |  |
|  | March | 230 | 0–0 | 0.00–0.00 | 0–8 | 0.00–3.02 |  |  |
|  | April | 261 | 0–27 | 0.00–11.11 | 0–0 | 0.00–0.00 |  |  |
|  | May | 283 | 21–52 | 8.90–22.06 | 0–0 | 0.00–0.00 | * |  |
|  | June | 240 | 0–9 | 0.00–3.76 | 0–0 | 0.00–0.00 |  |  |
|  | (c) Tuberculosis care beds |  |  |  |  |  |  |  |
|  | January | 79 | 5–27 | 9.16–50.33 | 0–0 | 0.00–0.00 | * |  |
|  | February | 80 | 7–29 | 11.92–54.14 | 0–0 | 0.00–0.00 | * |  |
|  | March | 23 | 0–0 | 0.00–0.00 | 11–30 | 20.11–56.21 |  | ** |
|  | April | 13 | 0–0 | 0.00–0.00 | 23–42 | 41.13–76.58 |  | ** |
|  | May | 36 | 0–0 | 0.00–0.00 | 2–20 | 2.75–34.88 |  | ** |
|  | June | 48 | 0–0 | 0.00–0.00 | 0–11 | 0.00–17.30 |  |  |
|  | (d) Long-term care beds |  |  |  |  |  |  |  |
|  | January | 192 | 0–4 | 0.00–2.10 | 0–0 | 0.00–0.00 |  |  |
|  | February | 123 | 0–0 | 0.00–0.00 | 37–63 | 19.92–33.78 |  | ** |
|  | March | 128 | 0–0 | 0.00–0.00 | 25–50 | 13.68–28.13 |  | ** |
|  | April | 131 | 0–0 | 0.00–0.00 | 24–50 | 13.16–27.45 |  | ** |
|  | May | 168 | 0–0 | 0.00–0.00 | 0–15 | 0.00–8.04 |  |  |
|  | June | 141 | 0–0 | 0.00–0.00 | 21–46 | 10.93–24.65 |  | ** |
|  | (e) General beds |  |  |  |  |  |  |  |
|  | January | 19 | 0–1 | 0.00–3.35 | 0–0 | 0.00–0.00 |  |  |
|  | February | 19 | 0–1 | 0.00–0.64 | 0–0 | 0.00–0.00 |  |  |
|  | March | 19 | 0–1 | 0.00–1.85 | 0–0 | 0.00–0.00 |  |  |
|  | April | 20 | 0–2 | 0.00–7.64 | 0–0 | 0.00–0.00 |  |  |
|  | May | 22 | 0–4 | 0.00–19.41 | 0–0 | 0.00–0.00 |  |  |
|  | June | 19 | 0–1 | 0.00–3.37 | 0–0 | 0.00–0.00 |  |  |
|  | (f) LTCI care beds |  |  |  |  |  |  |  |
|  | January | 494 | 0–29 | 0.00–6.17 | 0–0 | 0.00–0.00 |  |  |
|  | February | 399 | 0–0 | 0.00–0.00 | 0–58 | 0.00–12.54 |  |  |
|  | March | 151 | 0–0 | 0.00–0.00 | 227–291 | 51.39–65.74 |  | ** |
|  | April | 159 | 0–0 | 0.00–0.00 | 213–285 | 47.88–64.17 |  | ** |
|  | May | 373 | 0–0 | 0.00–0.00 | 0–71 | 0.00–15.95 |  |  |
|  | June | 251 | 0–0 | 0.00–0.00 | 136–213 | 29.12–45.92 |  | ** |
| Osaka | (1) Average number of outpatients per day at hospitals |  |  |  |  |  |  |  |
|  | General hospitals |  |  |  |  |  |  |  |
|  | January | 84244 | 0–0 | 0.00–0.00 | 0–2791 | 0.00–3.21 |  |  |
|  | February | 84427 | 0–0 | 0.00–0.00 | 0–3967 | 0.00–4.49 |  |  |
|  | March | 82396 | 0–0 | 0.00–0.00 | 1333–7209 | 1.49–8.04 |  | ** |
|  | April | 70988 | 0–0 | 0.00–0.00 | 10400–16356 | 11.91–18.73 |  | ** |
|  | May | 62338 | 0–0 | 0.00–0.00 | 19312–25381 | 22.02–28.93 |  | ** |
|  | June | 80770 | 0–0 | 0.00–0.00 | 1427–7341 | 1.62–8.33 |  | ** |
|  | Psychiatric hospitals |  |  |  |  |  |  |  |
|  | January | 2531 | 0–0 | 0.00–0.00 | 0–129 | 0.00–4.83 |  |  |
|  | February | 2653 | 0–0 | 0.00–0.00 | 0–37 | 0.00–1.36 |  |  |
|  | March | 2657 | 0–0 | 0.00–0.00 | 0–104 | 0.00–3.75 |  |  |
|  | April | 2526 | 0–0 | 0.00–0.00 | 0–181 | 0.00–6.67 |  |  |
|  | May | 2167 | 0–0 | 0.00–0.00 | 345–541 | 12.74–19.97 |  | ** |
|  | June | 2589 | 0–0 | 0.00–0.00 | 0–109 | 0.00–4.01 |  |  |
|  | (2) Average number of hospitalized patients per day |  |  |  |  |  |  |  |
|  | (b) Psychiatric care beds |  |  |  |  |  |  |  |
|  | January | 15882 | 0–0 | 0.00–0.00 | 0–3 | 0.00–0.02 |  |  |
|  | February | 15979 | 0–78 | 0.00–0.49 | 0–0 | 0.00–0.00 |  |  |
|  | March | 15842 | 0–0 | 0.00–0.00 | 0–60 | 0.00–0.37 |  |  |
|  | April | 15615 | 0–0 | 0.00–0.00 | 36–283 | 0.23–1.77 |  | ** |
|  | May | 15525 | 0–0 | 0.00–0.00 | 153–400 | 0.96–2.51 |  | ** |
|  | June | 15737 | 0–0 | 0.00–0.00 | 20–268 | 0.12–1.67 |  | ** |
|  | (c) Tuberculosis care beds |  |  |  |  |  |  |  |
|  | January | 215 | 0–0 | 0.00–0.00 | 0–27 | 0.00–10.87 |  |  |
|  | February | 218 | 0–0 | 0.00–0.00 | 0–20 | 0.00–8.38 |  |  |
|  | March | 217 | 0–0 | 0.00–0.00 | 0–22 | 0.00–9.19 |  |  |
|  | April | 234 | 0–0 | 0.00–0.00 | 0–4 | 0.00–1.55 |  |  |
|  | May | 219 | 0–0 | 0.00–0.00 | 0–18 | 0.00–7.51 |  |  |
|  | June | 198 | 0–0 | 0.00–0.00 | 8–37 | 3.40–15.73 |  | ** |
|  | (d) Long-term care beds |  |  |  |  |  |  |  |
|  | January | 18986 | 0–0 | 0.00–0.00 | 220–493 | 1.13–2.53 |  | ** |
|  | February | 19084 | 0–0 | 0.00–0.00 | 165–438 | 0.85–2.24 |  | ** |
|  | March | 19061 | 0–0 | 0.00–0.00 | 214–488 | 1.09–2.49 |  | ** |
|  | April | 18999 | 0–0 | 0.00–0.00 | 188–461 | 0.97–2.37 |  | ** |
|  | May | 18494 | 0–0 | 0.00–0.00 | 607–879 | 3.13–4.54 |  | ** |
|  | June | 18481 | 0–0 | 0.00–0.00 | 527–798 | 2.73–4.14 |  | ** |
|  | (e) General beds |  |  |  |  |  |  |  |
|  | January | 52118 | 0–0 | 0.00–0.00 | 0–642 | 0.00–1.22 |  |  |
|  | February | 53140 | 0–0 | 0.00–0.00 | 0–315 | 0.00–0.59 |  |  |
|  | March | 50919 | 0–0 | 0.00–0.00 | 1198–2582 | 2.24–4.82 |  | ** |
|  | April | 47391 | 0–0 | 0.00–0.00 | 3337–5054 | 6.36–9.64 |  | ** |
|  | May | 44004 | 0–0 | 0.00–0.00 | 6602–7936 | 12.71–15.28 |  | ** |
|  | June | 46812 | 0–0 | 0.00–0.00 | 3756–5086 | 7.24–9.80 |  | ** |
|  | (f) LTCI care beds |  |  |  |  |  |  |  |
|  | January | 802 | 0–0 | 0.00–0.00 | 94–268 | 8.79–24.98 |  | ** |
|  | February | 808 | 0–0 | 0.00–0.00 | 66–234 | 6.34–22.38 |  | ** |
|  | March | 788 | 0–0 | 0.00–0.00 | 62–225 | 6.12–22.19 |  | ** |
|  | April | 591 | 0–0 | 0.00–0.00 | 228–388 | 23.30–39.62 |  | ** |
|  | May | 596 | 0–0 | 0.00–0.00 | 198–355 | 20.82–37.33 |  | ** |
|  | June | 607 | 0–0 | 0.00–0.00 | 172–321 | 18.54–34.56 |  | ** |
|  | (3) Average length of hospital stays per patient |  |  |  |  |  |  |  |
|  | (b) Psychiatric care beds |  |  |  |  |  |  |  |
|  | January | 232 | 0–6 | 0.00–2.53 | 0–0 | 0.00–0.00 |  |  |
|  | February | 220 | 0–0 | 0.00–0.00 | 0–1 | 0.00–0.31 |  |  |
|  | March | 223 | 0–7 | 0.00–3.11 | 0–0 | 0.00–0.00 |  |  |
|  | April | 248 | 2–31 | 0.60–14.20 | 0–0 | 0.00–0.00 | * |  |
|  | May | 258 | 14–44 | 6.34–20.10 | 0–0 | 0.00–0.00 | * |  |
|  | June | 225 | 0–13 | 0.00–5.77 | 0–0 | 0.00–0.00 |  |  |
|  | (c) Tuberculosis care beds |  |  |  |  |  |  |  |
|  | January | 76 | 0–4 | 0.00–4.58 | 0–0 | 0.00–0.00 |  |  |
|  | February | 74 | 0–0 | 0.00–0.00 | 0–1 | 0.00–1.20 |  |  |
|  | March | 60 | 0–0 | 0.00–0.00 | 0–16 | 0.00–20.53 |  |  |
|  | April | 71 | 0–0 | 0.00–0.00 | 0–8 | 0.00–9.64 |  |  |
|  | May | 78 | 0–0 | 0.00–0.00 | 0–2 | 0.00–1.81 |  |  |
|  | June | 79 | 0–2 | 0.00–2.36 | 0–0 | 0.00–0.00 |  |  |
|  | (d) Long-term care beds |  |  |  |  |  |  |  |
|  | January | 151 | 0–4 | 0.00–2.28 | 0–0 | 0.00–0.00 |  |  |
|  | February | 150 | 0–4 | 0.00–2.60 | 0–0 | 0.00–0.00 |  |  |
|  | March | 152 | 0–9 | 0.00–5.77 | 0–0 | 0.00–0.00 |  |  |
|  | April | 159 | 0–12 | 0.00–7.49 | 0–0 | 0.00–0.00 |  |  |
|  | May | 172 | 0–23 | 0.00–15.26 | 0–0 | 0.00–0.00 |  |  |
|  | June | 161 | 0–11 | 0.00–6.88 | 0–0 | 0.00–0.00 |  |  |
|  | (e) General beds |  |  |  |  |  |  |  |
|  | January | 16 | 0–1 | 0.00–3.68 | 0–0 | 0.00–0.00 |  |  |
|  | February | 16 | 0–1 | 0.00–1.57 | 0–0 | 0.00–0.00 |  |  |
|  | March | 16 | 0–1 | 0.00–3.15 | 0–0 | 0.00–0.00 |  |  |
|  | April | 17 | 0–2 | 0.00–9.66 | 0–0 | 0.00–0.00 |  |  |
|  | May | 19 | 0–4 | 0.00–21.12 | 0–0 | 0.00–0.00 |  |  |
|  | June | 16 | 0–1 | 0.00–4.21 | 0–0 | 0.00–0.00 |  |  |
|  | (f) LTCI care beds |  |  |  |  |  |  |  |
|  | January | 460 | 90–156 | 29.32–51.15 | 0–0 | 0.00–0.00 | * |  |
|  | February | 438 | 84–149 | 28.67–51.17 | 0–0 | 0.00–0.00 | * |  |
|  | March | 411 | 67–128 | 23.46–44.83 | 0–0 | 0.00–0.00 | * |  |
|  | April | 152 | 0–0 | 0.00–0.00 | 91–152 | 29.90–49.95 |  | ** |
|  | May | 480 | 92–162 | 28.90–50.92 | 0–0 | 0.00–0.00 | * |  |
|  | June | 521 | 135–200 | 41.96–62.38 | 0–0 | 0.00–0.00 | * |  |
| Hyogo | (1) Average number of outpatients per day at hospitals |  |  |  |  |  |  |  |
|  | General hospitals |  |  |  |  |  |  |  |
|  | January | 50084 | 0–0 | 0.00–0.00 | 0–1859 | 0.00–3.58 |  |  |
|  | February | 50033 | 0–0 | 0.00–0.00 | 0–2711 | 0.00–5.14 |  |  |
|  | March | 48603 | 0–0 | 0.00–0.00 | 1444–4986 | 2.69–9.30 |  | ** |
|  | April | 42711 | 0–0 | 0.00–0.00 | 6093–9606 | 11.65–18.36 |  | ** |
|  | May | 37758 | 0–0 | 0.00–0.00 | 11132–14746 | 21.20–28.08 |  | ** |
|  | June | 48276 | 0–0 | 0.00–0.00 | 885–4449 | 1.68–8.44 |  | ** |
|  | Psychiatric hospitals |  |  |  |  |  |  |  |
|  | January | 1586 | 0–0 | 0.00–0.00 | 0–90 | 0.00–5.32 |  |  |
|  | February | 1667 | 0–0 | 0.00–0.00 | 0–41 | 0.00–2.39 |  |  |
|  | March | 1630 | 0–0 | 0.00–0.00 | 0–4 | 0.00–0.19 |  |  |
|  | April | 1565 | 0–0 | 0.00–0.00 | 59–170 | 3.40–9.75 |  | ** |
|  | May | 1381 | 0–0 | 0.00–0.00 | 246–367 | 14.07–20.99 |  | ** |
|  | June | 1614 | 0–0 | 0.00–0.00 | 30–140 | 1.71–7.94 |  | ** |
|  | (2) Average number of hospitalized patients per day |  |  |  |  |  |  |  |
|  | (b) Psychiatric care beds |  |  |  |  |  |  |  |
|  | January | 9982 | 0–0 | 0.00–0.00 | 0–72 | 0.00–0.71 |  |  |
|  | February | 9999 | 0–0 | 0.00–0.00 | 0–63 | 0.00–0.62 |  |  |
|  | March | 9927 | 0–0 | 0.00–0.00 | 0–131 | 0.00–1.30 |  |  |
|  | April | 9789 | 0–0 | 0.00–0.00 | 79–275 | 0.78–2.73 |  | ** |
|  | May | 9793 | 0–0 | 0.00–0.00 | 85–281 | 0.84–2.78 |  | ** |
|  | June | 9866 | 0–0 | 0.00–0.00 | 44–240 | 0.44–2.37 |  | ** |
|  | (c) Tuberculosis care beds |  |  |  |  |  |  |  |
|  | January | 71 | 0–0 | 0.00–0.00 | 0–5 | 0.00–5.45 |  |  |
|  | February | 69 | 0–0 | 0.00–0.00 | 0–4 | 0.00–5.19 |  |  |
|  | March | 60 | 0–0 | 0.00–0.00 | 0–11 | 0.00–15.05 |  |  |
|  | April | 68 | 0–0 | 0.00–0.00 | 0–3 | 0.00–3.58 |  |  |
|  | May | 60 | 0–0 | 0.00–0.00 | 0–12 | 0.00–16.33 |  |  |
|  | June | 59 | 0–0 | 0.00–0.00 | 0–15 | 0.00–19.90 |  |  |
|  | (d) Long-term care beds |  |  |  |  |  |  |  |
|  | January | 11646 | 0–0 | 0.00–0.00 | 266–592 | 2.17–4.84 |  | ** |
|  | February | 11650 | 0–0 | 0.00–0.00 | 217–558 | 1.78–4.56 |  | ** |
|  | March | 11544 | 0–0 | 0.00–0.00 | 254–608 | 2.09–5.00 |  | ** |
|  | April | 11321 | 0–0 | 0.00–0.00 | 324–700 | 2.70–5.82 |  | ** |
|  | May | 11165 | 0–0 | 0.00–0.00 | 370–743 | 3.11–6.24 |  | ** |
|  | June | 11132 | 0–0 | 0.00–0.00 | 340–721 | 2.87–6.08 |  | ** |
|  | (e) General beds |  |  |  |  |  |  |  |
|  | January | 30457 | 0–0 | 0.00–0.00 | 0–649 | 0.00–2.09 |  |  |
|  | February | 30930 | 0–0 | 0.00–0.00 | 0–586 | 0.00–1.86 |  |  |
|  | March | 29338 | 0–0 | 0.00–0.00 | 1202–2104 | 3.82–6.69 |  | ** |
|  | April | 27248 | 0–0 | 0.00–0.00 | 2410–3490 | 7.84–11.35 |  | ** |
|  | May | 25398 | 0–0 | 0.00–0.00 | 4089–4917 | 13.49–16.22 |  | ** |
|  | June | 26916 | 0–0 | 0.00–0.00 | 2399–3292 | 7.94–10.90 |  | ** |
|  | (f) LTCI care beds |  |  |  |  |  |  |  |
|  | January | 353 | 0–0 | 0.00–0.00 | 279–451 | 34.74–56.04 |  | ** |
|  | February | 306 | 0–0 | 0.00–0.00 | 233–438 | 31.35–58.83 |  | ** |
|  | March | 307 | 0–0 | 0.00–0.00 | 156–376 | 22.85–55.02 |  | ** |
|  | April | 246 | 0–0 | 0.00–0.00 | 190–405 | 29.22–62.16 |  | ** |
|  | May | 249 | 0–0 | 0.00–0.00 | 131–350 | 21.90–58.37 |  | ** |
|  | June | 249 | 0–0 | 0.00–0.00 | 55–291 | 10.20–53.84 |  | ** |
|  | (3) Average length of hospital stays per patient |  |  |  |  |  |  |  |
|  | (b) Psychiatric care beds |  |  |  |  |  |  |  |
|  | January | 276 | 0–29 | 0.00–11.48 | 0–0 | 0.00–0.00 |  |  |
|  | February | 269 | 0–27 | 0.00–10.78 | 0–0 | 0.00–0.00 |  |  |
|  | March | 280 | 13–44 | 5.33–18.26 | 0–0 | 0.00–0.00 | * |  |
|  | April | 300 | 31–62 | 12.62–25.86 | 0–0 | 0.00–0.00 | * |  |
|  | May | 294 | 27–58 | 11.07–24.31 | 0–0 | 0.00–0.00 | * |  |
|  | June | 256 | 0–22 | 0.00–9.12 | 0–0 | 0.00–0.00 |  |  |
|  | (c) Tuberculosis care beds |  |  |  |  |  |  |  |
|  | January | 75 | 0–0 | 0.00–0.00 | 0–13 | 0.00–14.34 |  |  |
|  | February | 65 | 0–0 | 0.00–0.00 | 2–22 | 1.50–25.31 |  | ** |
|  | March | 57 | 0–0 | 0.00–0.00 | 7–27 | 8.11–31.82 |  | ** |
|  | April | 53 | 0–0 | 0.00–0.00 | 14–34 | 15.40–38.98 |  | ** |
|  | May | 59 | 0–0 | 0.00–0.00 | 6–27 | 7.02–31.02 |  | ** |
|  | June | 64 | 0–0 | 0.00–0.00 | 3–23 | 2.89–26.59 |  | ** |
|  | (d) Long-term care beds |  |  |  |  |  |  |  |
|  | January | 141 | 0–8 | 0.00–5.76 | 0–0 | 0.00–0.00 |  |  |
|  | February | 135 | 0–4 | 0.00–2.67 | 0–0 | 0.00–0.00 |  |  |
|  | March | 135 | 0–6 | 0.00–4.56 | 0–0 | 0.00–0.00 |  |  |
|  | April | 148 | 0–15 | 0.00–10.50 | 0–0 | 0.00–0.00 |  |  |
|  | May | 160 | 1–24 | 0.07–17.47 | 0–0 | 0.00–0.00 | * |  |
|  | June | 142 | 0–4 | 0.00–2.66 | 0–0 | 0.00–0.00 |  |  |
|  | (e) General beds |  |  |  |  |  |  |  |
|  | January | 17 | 0–1 | 0.00–3.00 | 0–0 | 0.00–0.00 |  |  |
|  | February | 16 | 0–0 | 0.00–0.00 | 0–1 | 0.00–0.20 |  |  |
|  | March | 16 | 0–1 | 0.00–1.75 | 0–0 | 0.00–0.00 |  |  |
|  | April | 17 | 0–2 | 0.00–8.37 | 0–0 | 0.00–0.00 |  |  |
|  | May | 19 | 0–4 | 0.00–20.43 | 0–0 | 0.00–0.00 |  |  |
|  | June | 16 | 0–1 | 0.00–3.62 | 0–0 | 0.00–0.00 |  |  |
|  | (f) LTCI care beds |  |  |  |  |  |  |  |
|  | January | 215 | 0–0 | 0.00–0.00 | 44–147 | 12.01–40.60 |  | ** |
|  | February | 493 | 29–148 | 8.16–42.65 | 0–0 | 0.00–0.00 | * |  |
|  | March | 235 | 0–0 | 0.00–0.00 | 2–96 | 0.60–29.00 |  | ** |
|  | April | 510 | 17–148 | 4.59–40.80 | 0–0 | 0.00–0.00 | * |  |
|  | May | 483 | 0–104 | 0.00–27.39 | 0–0 | 0.00–0.00 |  |  |
|  | June | 623 | 75–208 | 18.05–49.92 | 0–0 | 0.00–0.00 | * |  |
| Nara | (1) Average number of outpatients per day at hospitals |  |  |  |  |  |  |  |
|  | General hospitals |  |  |  |  |  |  |  |
|  | January | 14264 | 0–0 | 0.00–0.00 | 0–442 | 0.00–3.00 |  |  |
|  | February | 14080 | 0–0 | 0.00–0.00 | 0–862 | 0.00–5.76 |  |  |
|  | March | 13986 | 0–0 | 0.00–0.00 | 199–1199 | 1.31–7.89 |  | ** |
|  | April | 12511 | 0–0 | 0.00–0.00 | 1396–2352 | 9.39–15.82 |  | ** |
|  | May | 10992 | 0–0 | 0.00–0.00 | 2982–3999 | 19.89–26.67 |  | ** |
|  | June | 13723 | 0–0 | 0.00–0.00 | 381–1387 | 2.52–9.17 |  | ** |
|  | Psychiatric hospitals |  |  |  |  |  |  |  |
|  | January | 139 | 0–0 | 0.00–0.00 | 0–16 | 0.00–9.92 |  |  |
|  | February | 150 | 0–0 | 0.00–0.00 | 0–8 | 0.00–4.96 |  |  |
|  | March | 148 | 0–0 | 0.00–0.00 | 0–15 | 0.00–9.13 |  |  |
|  | April | 147 | 0–0 | 0.00–0.00 | 0–12 | 0.00–7.11 |  |  |
|  | May | 128 | 0–0 | 0.00–0.00 | 7–31 | 4.42–19.22 |  | ** |
|  | June | 146 | 0–0 | 0.00–0.00 | 0–12 | 0.00–7.39 |  |  |
|  | (2) Average number of hospitalized patients per day |  |  |  |  |  |  |  |
|  | (b) Psychiatric care beds |  |  |  |  |  |  |  |
|  | January | 2510 | 0–2 | 0.00–0.05 | 0–0 | 0.00–0.00 |  |  |
|  | February | 2508 | 0–1 | 0.00–0.03 | 0–0 | 0.00–0.00 |  |  |
|  | March | 2507 | 0–0 | 0.00–0.00 | 0–2 | 0.00–0.07 |  |  |
|  | April | 2467 | 0–0 | 0.00–0.00 | 0–44 | 0.00–1.74 |  |  |
|  | May | 2431 | 0–0 | 0.00–0.00 | 0–88 | 0.00–3.47 |  |  |
|  | June | 2450 | 0–0 | 0.00–0.00 | 0–79 | 0.00–3.10 |  |  |
|  | (c) Tuberculosis care beds |  |  |  |  |  |  |  |
|  | January | 23 | 0–0 | 0.00–0.00 | 0–1 | 0.00–0.02 |  |  |
|  | February | 24 | 0–2 | 0.00–7.67 | 0–0 | 0.00–0.00 |  |  |
|  | March | 22 | 0–0 | 0.00–0.00 | 0–1 | 0.00–3.07 |  |  |
|  | April | 21 | 0–0 | 0.00–0.00 | 0–2 | 0.00–8.56 |  |  |
|  | May | 21 | 0–0 | 0.00–0.00 | 0–3 | 0.00–10.54 |  |  |
|  | June | 21 | 0–0 | 0.00–0.00 | 0–3 | 0.00–12.06 |  |  |
|  | (d) Long-term care beds |  |  |  |  |  |  |  |
|  | January | 2364 | 0–0 | 0.00–0.00 | 0–126 | 0.00–5.06 |  |  |
|  | February | 2409 | 0–0 | 0.00–0.00 | 0–78 | 0.00–3.12 |  |  |
|  | March | 2418 | 0–0 | 0.00–0.00 | 0–71 | 0.00–2.82 |  |  |
|  | April | 2384 | 0–0 | 0.00–0.00 | 0–85 | 0.00–3.41 |  |  |
|  | May | 2337 | 0–0 | 0.00–0.00 | 0–115 | 0.00–4.67 |  |  |
|  | June | 2316 | 0–0 | 0.00–0.00 | 0–105 | 0.00–4.31 |  |  |
|  | (e) General beds |  |  |  |  |  |  |  |
|  | January | 8019 | 0–0 | 0.00–0.00 | 0–243 | 0.00–2.94 |  |  |
|  | February | 8148 | 0–0 | 0.00–0.00 | 0–216 | 0.00–2.57 |  |  |
|  | March | 7729 | 0–0 | 0.00–0.00 | 343–608 | 4.11–7.29 |  | ** |
|  | April | 7163 | 0–0 | 0.00–0.00 | 653–984 | 8.02–12.07 |  | ** |
|  | May | 6425 | 0–0 | 0.00–0.00 | 1326–1604 | 16.52–19.97 |  | ** |
|  | June | 6710 | 0–0 | 0.00–0.00 | 1012–1284 | 12.66–16.06 |  | ** |
|  | (f) LTCI care beds |  |  |  |  |  |  |  |
|  | January | 21 | 0–0 | 0.00–0.00 | 45–204 | 20.03–90.65 |  | ** |
|  | February | 22 | 0–0 | 0.00–0.00 | 33–188 | 15.78–89.48 |  | ** |
|  | March | 21 | 0–0 | 0.00–0.00 | 23–172 | 11.93–89.10 |  | ** |
|  | April | 21 | 0–0 | 0.00–0.00 | 14–156 | 7.94–88.09 |  | ** |
|  | May | 22 | 0–0 | 0.00–0.00 | 6–139 | 3.74–86.28 |  | ** |
|  | June | 21 | 0–0 | 0.00–0.00 | 1–128 | 0.67–85.85 |  | ** |
|  | (3) Average length of hospital stays per patient |  |  |  |  |  |  |  |
|  | (b) Psychiatric care beds |  |  |  |  |  |  |  |
|  | January | 247 | 0–0 | 0.00–0.00 | 0–9 | 0.00–3.18 |  |  |
|  | February | 241 | 0–0 | 0.00–0.00 | 0–9 | 0.00–3.58 |  |  |
|  | March | 246 | 0–1 | 0.00–0.38 | 0–0 | 0.00–0.00 |  |  |
|  | April | 259 | 0–17 | 0.00–6.81 | 0–0 | 0.00–0.00 |  |  |
|  | May | 278 | 8–39 | 3.18–16.32 | 0–0 | 0.00–0.00 | * |  |
|  | June | 245 | 0–7 | 0.00–2.51 | 0–0 | 0.00–0.00 |  |  |
|  | (c) Tuberculosis care beds |  |  |  |  |  |  |  |
|  | January | 158 | 18–64 | 18.91–67.68 | 0–0 | 0.00–0.00 | * |  |
|  | February | 152 | 19–67 | 21.35–78.57 | 0–0 | 0.00–0.00 | * |  |
|  | March | 113 | 0–32 | 0.00–38.07 | 0–0 | 0.00–0.00 |  |  |
|  | April | 82 | 0–0 | 0.00–0.00 | 0–6 | 0.00–6.06 |  |  |
|  | May | 107 | 0–19 | 0.00–21.20 | 0–0 | 0.00–0.00 |  |  |
|  | June | 66 | 0–0 | 0.00–0.00 | 0–19 | 0.00–21.42 |  |  |
|  | (d) Long-term care beds |  |  |  |  |  |  |  |
|  | January | 104 | 0–1 | 0.00–0.05 | 0–0 | 0.00–0.00 |  |  |
|  | February | 101 | 0–0 | 0.00–0.00 | 0–2 | 0.00–1.89 |  |  |
|  | March | 96 | 0–0 | 0.00–0.00 | 0–3 | 0.00–2.96 |  |  |
|  | April | 99 | 0–0 | 0.00–0.00 | 0–1 | 0.00–0.76 |  |  |
|  | May | 107 | 0–6 | 0.00–5.36 | 0–0 | 0.00–0.00 |  |  |
|  | June | 103 | 0–2 | 0.00–1.29 | 0–0 | 0.00–0.00 |  |  |
|  | (e) General beds |  |  |  |  |  |  |  |
|  | January | 17 | 0–1 | 0.00–3.41 | 0–0 | 0.00–0.00 |  |  |
|  | February | 17 | 0–1 | 0.00–1.82 | 0–0 | 0.00–0.00 |  |  |
|  | March | 17 | 0–1 | 0.00–0.02 | 0–0 | 0.00–0.00 |  |  |
|  | April | 17 | 0–1 | 0.00–3.54 | 0–0 | 0.00–0.00 |  |  |
|  | May | 19 | 0–3 | 0.00–13.72 | 0–0 | 0.00–0.00 |  |  |
|  | June | 16 | 0–1 | 0.00–0.07 | 0–0 | 0.00–0.00 |  |  |
|  | (f) LTCI care beds |  |  |  |  |  |  |  |
|  | January | 330 | 0–59 | 0.00–21.37 | 0–0 | 0.00–0.00 |  |  |
|  | February | 0 | NA | NA | NA | NA |  |  |
|  | March | 0 | NA | NA | NA | NA |  |  |
|  | April | 0 | NA | NA | NA | NA |  |  |
|  | May | 0 | NA | NA | NA | NA |  |  |
|  | June | 1232 | 852–989 | 349.75–405.73 | 0–0 | 0.00–0.00 | * |  |
| Wakayama | (1) Average number of outpatients per day at hospitals |  |  |  |  |  |  |  |
|  | General hospitals |  |  |  |  |  |  |  |
|  | January | 10488 | 0–0 | 0.00–0.00 | 0–206 | 0.00–1.92 |  |  |
|  | February | 10088 | 0–0 | 0.00–0.00 | 25–765 | 0.23–7.05 |  | ** |
|  | March | 10284 | 0–0 | 0.00–0.00 | 0–695 | 0.00–6.33 |  |  |
|  | April | 9676 | 0–0 | 0.00–0.00 | 366–1054 | 3.41–9.82 |  | ** |
|  | May | 8481 | 0–0 | 0.00–0.00 | 1617–2345 | 14.94–21.66 |  | ** |
|  | June | 10444 | 0–0 | 0.00–0.00 | 0–484 | 0.00–4.42 |  |  |
|  | Psychiatric hospitals |  |  |  |  |  |  |  |
|  | January | 342 | 0–0 | 0.00–0.00 | 0–16 | 0.00–4.42 |  |  |
|  | February | 352 | 0–0 | 0.00–0.00 | 0–11 | 0.00–2.98 |  |  |
|  | March | 366 | 0–0 | 0.00–0.00 | 0–8 | 0.00–2.07 |  |  |
|  | April | 368 | 0–3 | 0.00–0.59 | 0–0 | 0.00–0.00 |  |  |
|  | May | 319 | 0–0 | 0.00–0.00 | 11–48 | 3.00–12.88 |  | ** |
|  | June | 374 | 0–9 | 0.00–2.43 | 0–0 | 0.00–0.00 |  |  |
|  | (2) Average number of hospitalized patients per day |  |  |  |  |  |  |  |
|  | (b) Psychiatric care beds |  |  |  |  |  |  |  |
|  | January | 1514 | 0–0 | 0.00–0.00 | 0–5 | 0.00–0.27 |  |  |
|  | February | 1505 | 0–0 | 0.00–0.00 | 0–12 | 0.00–0.77 |  |  |
|  | March | 1504 | 0–0 | 0.00–0.00 | 0–12 | 0.00–0.74 |  |  |
|  | April | 1482 | 0–0 | 0.00–0.00 | 0–32 | 0.00–2.11 |  |  |
|  | May | 1467 | 0–0 | 0.00–0.00 | 0–47 | 0.00–3.08 |  |  |
|  | June | 1474 | 0–0 | 0.00–0.00 | 0–41 | 0.00–2.71 |  |  |
|  | (c) Tuberculosis care beds |  |  |  |  |  |  |  |
|  | January | 7 | 0–0 | 0.00–0.00 | 0–1 | 0.00–8.68 |  |  |
|  | February | 9 | 0–3 | 0.00–32.18 | 0–0 | 0.00–0.00 |  |  |
|  | March | 9 | 0–2 | 0.00–25.76 | 0–0 | 0.00–0.00 |  |  |
|  | April | 8 | 0–0 | 0.00–0.00 | 0–1 | 0.00–1.71 |  |  |
|  | May | 6 | 0–0 | 0.00–0.00 | 0–4 | 0.00–34.35 |  |  |
|  | June | 5 | 0–0 | 0.00–0.00 | 0–5 | 0.00–45.80 |  |  |
|  | (d) Long-term care beds |  |  |  |  |  |  |  |
|  | January | 2017 | 0–0 | 0.00–0.00 | 69–191 | 3.13–8.61 |  | ** |
|  | February | 2022 | 0–0 | 0.00–0.00 | 76–195 | 3.43–8.78 |  | ** |
|  | March | 1942 | 0–0 | 0.00–0.00 | 139–262 | 6.31–11.85 |  | ** |
|  | April | 1820 | 0–0 | 0.00–0.00 | 186–325 | 8.67–15.14 |  | ** |
|  | May | 1796 | 0–0 | 0.00–0.00 | 148–296 | 7.08–14.12 |  | ** |
|  | June | 1789 | 0–0 | 0.00–0.00 | 119–267 | 5.79–12.98 |  | ** |
|  | (e) General beds |  |  |  |  |  |  |  |
|  | January | 6950 | 0–132 | 0.00–1.93 | 0–0 | 0.00–0.00 |  |  |
|  | February | 6944 | 0–26 | 0.00–0.36 | 0–0 | 0.00–0.00 |  |  |
|  | March | 6649 | 0–0 | 0.00–0.00 | 27–258 | 0.39–3.73 |  | ** |
|  | April | 6268 | 0–0 | 0.00–0.00 | 220–485 | 3.26–7.18 |  | ** |
|  | May | 5848 | 0–0 | 0.00–0.00 | 657–852 | 9.81–12.71 |  | ** |
|  | June | 6119 | 0–0 | 0.00–0.00 | 359–587 | 5.35–8.74 |  | ** |
|  | (f) LTCI care beds |  |  |  |  |  |  |  |
|  | January | 238 | 0–0 | 0.00–0.00 | 94–131 | 25.53–35.35 |  | ** |
|  | February | 196 | 0–0 | 0.00–0.00 | 125–161 | 35.02–45.10 |  | ** |
|  | March | 194 | 0–0 | 0.00–0.00 | 117–154 | 33.68–44.15 |  | ** |
|  | April | 107 | 0–0 | 0.00–0.00 | 177–222 | 53.80–67.48 |  | ** |
|  | May | 108 | 0–0 | 0.00–0.00 | 153–204 | 49.05–65.38 |  | ** |
|  | June | 106 | 0–0 | 0.00–0.00 | 144–199 | 47.24–65.22 |  | ** |
|  | (3) Average length of hospital stays per patient |  |  |  |  |  |  |  |
|  | (b) Psychiatric care beds |  |  |  |  |  |  |  |
|  | January | 286 | 0–0 | 0.00–0.00 | 0–7 | 0.00–2.06 |  |  |
|  | February | 282 | 0–0 | 0.00–0.00 | 0–2 | 0.00–0.40 |  |  |
|  | March | 303 | 0–34 | 0.00–12.36 | 0–0 | 0.00–0.00 |  |  |
|  | April | 288 | 0–19 | 0.00–6.92 | 0–0 | 0.00–0.00 |  |  |
|  | May | 323 | 20–55 | 7.27–20.18 | 0–0 | 0.00–0.00 | * |  |
|  | June | 270 | 0–1 | 0.00–0.01 | 0–0 | 0.00–0.00 |  |  |
|  | (c) Tuberculosis care beds |  |  |  |  |  |  |  |
|  | January | 406 | 26–212 | 13.34–108.34 | 0–0 | 0.00–0.00 | * |  |
|  | February | 89 | 0–0 | 0.00–0.00 | 2–126 | 0.93–58.49 |  | ** |
|  | March | 574 | 89–313 | 33.98–119.15 | 0–0 | 0.00–0.00 | * |  |
|  | April | 167 | 0–0 | 0.00–0.00 | 0–110 | 0.00–39.50 |  |  |
|  | May | 53 | 0–0 | 0.00–0.00 | 49–196 | 19.50–78.81 |  | ** |
|  | June | 290 | 0–88 | 0.00–43.01 | 0–0 | 0.00–0.00 |  |  |
|  | (d) Long-term care beds |  |  |  |  |  |  |  |
|  | January | 119 | 0–6 | 0.00–4.54 | 0–0 | 0.00–0.00 |  |  |
|  | February | 109 | 0–0 | 0.00–0.00 | 0–3 | 0.00–1.86 |  |  |
|  | March | 101 | 0–0 | 0.00–0.00 | 0–9 | 0.00–7.68 |  |  |
|  | April | 108 | 0–0 | 0.00–0.00 | 0–6 | 0.00–5.08 |  |  |
|  | May | 124 | 0–10 | 0.00–8.47 | 0–0 | 0.00–0.00 |  |  |
|  | June | 105 | 0–0 | 0.00–0.00 | 0–11 | 0.00–8.79 |  |  |
|  | (e) General beds |  |  |  |  |  |  |  |
|  | January | 20 | 0–2 | 0.00–6.38 | 0–0 | 0.00–0.00 |  |  |
|  | February | 20 | 0–2 | 0.00–6.28 | 0–0 | 0.00–0.00 |  |  |
|  | March | 20 | 0–2 | 0.00–7.40 | 0–0 | 0.00–0.00 |  |  |
|  | April | 20 | 0–1 | 0.00–3.65 | 0–0 | 0.00–0.00 |  |  |
|  | May | 22 | 0–3 | 0.00–15.70 | 0–0 | 0.00–0.00 |  |  |
|  | June | 19 | 0–1 | 0.00–2.06 | 0–0 | 0.00–0.00 |  |  |
|  | (f) LTCI care beds |  |  |  |  |  |  |  |
|  | January | 242 | 0–0 | 0.00–0.00 | 0–19 | 0.00–6.94 |  |  |
|  | February | 134 | 0–0 | 0.00–0.00 | 57–108 | 23.38–44.63 |  | ** |
|  | March | 79 | 0–0 | 0.00–0.00 | 112–166 | 45.81–67.62 |  | ** |
|  | April | 402 | 90–153 | 35.65–60.90 | 0–0 | 0.00–0.00 | * |  |
|  | May | 743 | 419–480 | 158.80–182.23 | 0–0 | 0.00–0.00 | * |  |
|  | June | 531 | 205–272 | 78.90–104.55 | 0–0 | 0.00–0.00 | * |  |
| Tottori | (1) Average number of outpatients per day at hospitals |  |  |  |  |  |  |  |
|  | General hospitals |  |  |  |  |  |  |  |
|  | January | 5959 | 0–0 | 0.00–0.00 | 0–35 | 0.00–0.57 |  |  |
|  | February | 5741 | 0–0 | 0.00–0.00 | 0–335 | 0.00–5.51 |  |  |
|  | March | 6028 | 0–0 | 0.00–0.00 | 0–108 | 0.00–1.76 |  |  |
|  | April | 5715 | 0–0 | 0.00–0.00 | 0–332 | 0.00–5.49 |  |  |
|  | May | 4885 | 0–0 | 0.00–0.00 | 733–1175 | 12.10–19.39 |  | ** |
|  | June | 5922 | 0–0 | 0.00–0.00 | 0–174 | 0.00–2.84 |  |  |
|  | Psychiatric hospitals |  |  |  |  |  |  |  |
|  | January | 222 | 0–4 | 0.00–1.59 | 0–0 | 0.00–0.00 |  |  |
|  | February | 226 | 0–4 | 0.00–1.77 | 0–0 | 0.00–0.00 |  |  |
|  | March | 236 | 0–6 | 0.00–2.39 | 0–0 | 0.00–0.00 |  |  |
|  | April | 208 | 0–0 | 0.00–0.00 | 0–20 | 0.00–8.60 |  |  |
|  | May | 185 | 0–0 | 0.00–0.00 | 16–46 | 6.96–19.57 |  | ** |
|  | June | 217 | 0–0 | 0.00–0.00 | 0–16 | 0.00–6.56 |  |  |
|  | (2) Average number of hospitalized patients per day |  |  |  |  |  |  |  |
|  | (b) Psychiatric care beds |  |  |  |  |  |  |  |
|  | January | 1443 | 0–0 | 0.00–0.00 | 24–101 | 1.56–6.50 |  | ** |
|  | February | 1438 | 0–0 | 0.00–0.00 | 18–94 | 1.17–6.13 |  | ** |
|  | March | 1452 | 0–0 | 0.00–0.00 | 0–58 | 0.00–3.80 |  |  |
|  | April | 1431 | 0–0 | 0.00–0.00 | 0–61 | 0.00–4.05 |  |  |
|  | May | 1427 | 0–0 | 0.00–0.00 | 0–50 | 0.00–3.38 |  |  |
|  | June | 1421 | 0–0 | 0.00–0.00 | 0–52 | 0.00–3.47 |  |  |
|  | (c) Tuberculosis care beds |  |  |  |  |  |  |  |
|  | January | 1 | 0–0 | 0.00–0.00 | 0–2 | 0.00–66.04 |  |  |
|  | February | 3 | 0–1 | 0.00–15.48 | 0–0 | 0.00–0.00 |  |  |
|  | March | 3 | 0–1 | 0.00–12.55 | 0–0 | 0.00–0.00 |  |  |
|  | April | 1 | 0–0 | 0.00–0.00 | 0–2 | 0.00–65.59 |  |  |
|  | May | 1 | 0–0 | 0.00–0.00 | 0–3 | 0.00–66.68 |  |  |
|  | June | 1 | 0–0 | 0.00–0.00 | 0–2 | 0.00–66.28 |  |  |
|  | (d) Long-term care beds |  |  |  |  |  |  |  |
|  | January | 1382 | 0–0 | 0.00–0.00 | 86–162 | 5.57–10.46 |  | ** |
|  | February | 1415 | 0–0 | 0.00–0.00 | 55–133 | 3.55–8.57 |  | ** |
|  | March | 1396 | 0–0 | 0.00–0.00 | 54–138 | 3.52–8.94 |  | ** |
|  | April | 1354 | 0–0 | 0.00–0.00 | 64–149 | 4.26–9.89 |  | ** |
|  | May | 1321 | 0–0 | 0.00–0.00 | 75–152 | 5.09–10.31 |  | ** |
|  | June | 1316 | 0–0 | 0.00–0.00 | 57–138 | 3.92–9.48 |  | ** |
|  | (e) General beds |  |  |  |  |  |  |  |
|  | January | 3917 | 0–0 | 0.00–0.00 | 0–26 | 0.00–0.64 |  |  |
|  | February | 3982 | 0–0 | 0.00–0.00 | 0–4 | 0.00–0.08 |  |  |
|  | March | 3799 | 0–0 | 0.00–0.00 | 71–194 | 1.78–4.85 |  | ** |
|  | April | 3595 | 0–0 | 0.00–0.00 | 145–299 | 3.72–7.66 |  | ** |
|  | May | 3460 | 0–0 | 0.00–0.00 | 248–372 | 6.47–9.69 |  | ** |
|  | June | 3660 | 0–0 | 0.00–0.00 | 32–152 | 0.84–3.98 |  | ** |
|  | (f) LTCI care beds |  |  |  |  |  |  |  |
|  | January | 61 | 0–0 | 0.00–0.00 | 98–124 | 53.18–66.90 |  | ** |
|  | February | 57 | 0–0 | 0.00–0.00 | 101–128 | 54.86–69.04 |  | ** |
|  | March | 57 | 0–0 | 0.00–0.00 | 94–119 | 53.42–67.61 |  | ** |
|  | April | 49 | 0–0 | 0.00–0.00 | 65–102 | 43.19–67.44 |  | ** |
|  | May | 47 | 0–0 | 0.00–0.00 | 48–87 | 35.99–64.76 |  | ** |
|  | June | 44 | 0–0 | 0.00–0.00 | 45–86 | 34.87–65.90 |  | ** |
|  | (3) Average length of hospital stays per patient |  |  |  |  |  |  |  |
|  | (b) Psychiatric care beds |  |  |  |  |  |  |  |
|  | January | 265 | 0–11 | 0.00–4.32 | 0–0 | 0.00–0.00 |  |  |
|  | February | 257 | 0–11 | 0.00–4.27 | 0–0 | 0.00–0.00 |  |  |
|  | March | 256 | 0–16 | 0.00–6.65 | 0–0 | 0.00–0.00 |  |  |
|  | April | 263 | 0–24 | 0.00–9.72 | 0–0 | 0.00–0.00 |  |  |
|  | May | 272 | 1–36 | 0.13–14.95 | 0–0 | 0.00–0.00 | * |  |
|  | June | 247 | 0–15 | 0.00–6.06 | 0–0 | 0.00–0.00 |  |  |
|  | (c) Tuberculosis care beds |  |  |  |  |  |  |  |
|  | January | 64 | 0–0 | 0.00–0.00 | 0–62 | 0.00–48.91 |  |  |
|  | February | 41 | 0–0 | 0.00–0.00 | 0–69 | 0.00–62.68 |  |  |
|  | March | 196 | 0–70 | 0.00–55.28 | 0–0 | 0.00–0.00 |  |  |
|  | April | 13 | 0–0 | 0.00–0.00 | 18–95 | 16.43–87.66 |  | ** |
|  | May | 0 | NA | NA | NA | NA |  |  |
|  | June | 0 | NA | NA | NA | NA |  |  |
|  | (d) Long-term care beds |  |  |  |  |  |  |  |
|  | January | 103 | 0–2 | 0.00–1.97 | 0–0 | 0.00–0.00 |  |  |
|  | February | 106 | 0–5 | 0.00–4.95 | 0–0 | 0.00–0.00 |  |  |
|  | March | 98 | 0–2 | 0.00–1.49 | 0–0 | 0.00–0.00 |  |  |
|  | April | 97 | 0–0 | 0.00–0.00 | 0–1 | 0.00–0.46 |  |  |
|  | May | 105 | 0–9 | 0.00–8.83 | 0–0 | 0.00–0.00 |  |  |
|  | June | 98 | 0–2 | 0.00–1.20 | 0–0 | 0.00–0.00 |  |  |
|  | (e) General beds |  |  |  |  |  |  |  |
|  | January | 18 | 0–1 | 0.00–0.62 | 0–0 | 0.00–0.00 |  |  |
|  | February | 18 | 0–0 | 0.00–0.00 | 0–1 | 0.00–2.25 |  |  |
|  | March | 17 | 0–0 | 0.00–0.00 | 0–1 | 0.00–4.37 |  |  |
|  | April | 17 | 0–0 | 0.00–0.00 | 0–1 | 0.00–4.60 |  |  |
|  | May | 18 | 0–2 | 0.00–6.62 | 0–0 | 0.00–0.00 |  |  |
|  | June | 17 | 0–0 | 0.00–0.00 | 0–1 | 0.00–1.68 |  |  |
|  | (f) LTCI care beds |  |  |  |  |  |  |  |
|  | January | 60 | 0–0 | 0.00–0.00 | 8–29 | 8.55–32.06 |  | ** |
|  | February | 64 | 0–0 | 0.00–0.00 | 5–26 | 4.68–28.85 |  | ** |
|  | March | 55 | 0–0 | 0.00–0.00 | 0–23 | 0.00–28.76 |  |  |
|  | April | 48 | 0–0 | 0.00–0.00 | 3–25 | 3.97–34.13 |  | ** |
|  | May | 61 | 0–0 | 0.00–0.00 | 0–12 | 0.00–15.48 |  |  |
|  | June | 41 | 0–0 | 0.00–0.00 | 5–26 | 7.01–38.41 |  | ** |
| Shimane | (1) Average number of outpatients per day at hospitals |  |  |  |  |  |  |  |
|  | General hospitals |  |  |  |  |  |  |  |
|  | January | 6466 | 0–0 | 0.00–0.00 | 0–168 | 0.00–2.53 |  |  |
|  | February | 6389 | 0–0 | 0.00–0.00 | 0–345 | 0.00–5.11 |  |  |
|  | March | 6578 | 0–0 | 0.00–0.00 | 0–264 | 0.00–3.85 |  |  |
|  | April | 6151 | 0–0 | 0.00–0.00 | 131–566 | 1.95–8.42 |  | ** |
|  | May | 5187 | 0–0 | 0.00–0.00 | 1093–1575 | 16.17–23.29 |  | ** |
|  | June | 6474 | 0–0 | 0.00–0.00 | 0–373 | 0.00–5.45 |  |  |
|  | Psychiatric hospitals |  |  |  |  |  |  |  |
|  | January | 408 | 0–19 | 0.00–4.77 | 0–0 | 0.00–0.00 |  |  |
|  | February | 423 | 0–19 | 0.00–4.54 | 0–0 | 0.00–0.00 |  |  |
|  | March | 438 | 0–16 | 0.00–3.70 | 0–0 | 0.00–0.00 |  |  |
|  | April | 428 | 0–6 | 0.00–1.42 | 0–0 | 0.00–0.00 |  |  |
|  | May | 374 | 0–0 | 0.00–0.00 | 13–55 | 3.04–12.62 |  | ** |
|  | June | 436 | 0–5 | 0.00–1.06 | 0–0 | 0.00–0.00 |  |  |
|  | (2) Average number of hospitalized patients per day |  |  |  |  |  |  |  |
|  | (b) Psychiatric care beds |  |  |  |  |  |  |  |
|  | January | 1957 | 0–38 | 0.00–1.98 | 0–0 | 0.00–0.00 |  |  |
|  | February | 1952 | 0–29 | 0.00–1.49 | 0–0 | 0.00–0.00 |  |  |
|  | March | 1983 | 0–61 | 0.00–3.14 | 0–0 | 0.00–0.00 |  |  |
|  | April | 1950 | 0–29 | 0.00–1.49 | 0–0 | 0.00–0.00 |  |  |
|  | May | 1908 | 0–0 | 0.00–0.00 | 0–15 | 0.00–0.74 |  |  |
|  | June | 1914 | 0–0 | 0.00–0.00 | 0–13 | 0.00–0.64 |  |  |
|  | (c) Tuberculosis care beds |  |  |  |  |  |  |  |
|  | January | 5 | 0–2 | 0.00–26.50 | 0–0 | 0.00–0.00 |  |  |
|  | February | 7 | 0–3 | 0.00–60.76 | 0–0 | 0.00–0.00 |  |  |
|  | March | 9 | 1–6 | 29.79–168.14 | 0–0 | 0.00–0.00 | * |  |
|  | April | 9 | 2–7 | 81.23–265.52 | 0–0 | 0.00–0.00 | * |  |
|  | May | 7 | 1–6 | 50.26–251.79 | 0–0 | 0.00–0.00 | * |  |
|  | June | 5 | 0–3 | 0.00–114.39 | 0–0 | 0.00–0.00 |  |  |
|  | (d) Long-term care beds |  |  |  |  |  |  |  |
|  | January | 1647 | 0–0 | 0.00–0.00 | 0–53 | 0.00–3.08 |  |  |
|  | February | 1595 | 0–0 | 0.00–0.00 | 33–113 | 1.93–6.60 |  | ** |
|  | March | 1558 | 0–0 | 0.00–0.00 | 59–140 | 3.48–8.19 |  | ** |
|  | April | 1565 | 0–0 | 0.00–0.00 | 33–113 | 1.97–6.68 |  | ** |
|  | May | 1517 | 0–0 | 0.00–0.00 | 69–149 | 4.14–8.89 |  | ** |
|  | June | 1545 | 0–0 | 0.00–0.00 | 41–120 | 2.46–7.20 |  | ** |
|  | (e) General beds |  |  |  |  |  |  |  |
|  | January | 4639 | 0–0 | 0.00–0.00 | 0–92 | 0.00–1.94 |  |  |
|  | February | 4693 | 0–0 | 0.00–0.00 | 0–91 | 0.00–1.89 |  |  |
|  | March | 4586 | 0–0 | 0.00–0.00 | 11–172 | 0.23–3.60 |  | ** |
|  | April | 4247 | 0–0 | 0.00–0.00 | 230–407 | 4.94–8.73 |  | ** |
|  | May | 3927 | 0–0 | 0.00–0.00 | 544–686 | 11.79–14.86 |  | ** |
|  | June | 4125 | 0–0 | 0.00–0.00 | 327–480 | 7.10–10.40 |  | ** |
|  | (f) LTCI care beds |  |  |  |  |  |  |  |
|  | January | 110 | 0–0 | 0.00–0.00 | 0–14 | 0.00–10.87 |  |  |
|  | February | 63 | 0–0 | 0.00–0.00 | 33–58 | 27.36–47.77 |  | ** |
|  | March | 40 | 0–0 | 0.00–0.00 | 53–77 | 45.59–65.59 |  | ** |
|  | April | 39 | 0–0 | 0.00–0.00 | 51–73 | 45.59–65.14 |  | ** |
|  | May | 38 | 0–0 | 0.00–0.00 | 49–71 | 45.36–64.82 |  | ** |
|  | June | 37 | 0–0 | 0.00–0.00 | 49–69 | 46.51–64.88 |  | ** |
|  | (3) Average length of hospital stays per patient |  |  |  |  |  |  |  |
|  | (b) Psychiatric care beds |  |  |  |  |  |  |  |
|  | January | 276 | 0–5 | 0.00–1.74 | 0–0 | 0.00–0.00 |  |  |
|  | February | 259 | 0–0 | 0.00–0.00 | 0–9 | 0.00–3.33 |  |  |
|  | March | 259 | 0–4 | 0.00–1.34 | 0–0 | 0.00–0.00 |  |  |
|  | April | 229 | 0–0 | 0.00–0.00 | 0–24 | 0.00–9.16 |  |  |
|  | May | 283 | 1–33 | 0.40–13.14 | 0–0 | 0.00–0.00 | * |  |
|  | June | 248 | 0–0 | 0.00–0.00 | 0–1 | 0.00–0.24 |  |  |
|  | (c) Tuberculosis care beds |  |  |  |  |  |  |  |
|  | January | 32 | 0–0 | 0.00–0.00 | 0–28 | 0.00–46.21 |  |  |
|  | February | 107 | 0–43 | 0.00–65.53 | 0–0 | 0.00–0.00 |  |  |
|  | March | 81 | 0–32 | 0.00–62.13 | 0–0 | 0.00–0.00 |  |  |
|  | April | 67 | 0–26 | 0.00–61.45 | 0–0 | 0.00–0.00 |  |  |
|  | May | 68 | 0–34 | 0.00–97.97 | 0–0 | 0.00–0.00 |  |  |
|  | June | 51 | 0–10 | 0.00–23.29 | 0–0 | 0.00–0.00 |  |  |
|  | (d) Long-term care beds |  |  |  |  |  |  |  |
|  | January | 136 | 0–7 | 0.00–5.39 | 0–0 | 0.00–0.00 |  |  |
|  | February | 131 | 0–2 | 0.00–1.22 | 0–0 | 0.00–0.00 |  |  |
|  | March | 124 | 0–0 | 0.00–0.00 | 0–2 | 0.00–1.52 |  |  |
|  | April | 119 | 0–0 | 0.00–0.00 | 0–8 | 0.00–6.07 |  |  |
|  | May | 138 | 0–12 | 0.00–8.90 | 0–0 | 0.00–0.00 |  |  |
|  | June | 118 | 0–0 | 0.00–0.00 | 0–10 | 0.00–7.39 |  |  |
|  | (e) General beds |  |  |  |  |  |  |  |
|  | January | 18 | 0–1 | 0.00–3.32 | 0–0 | 0.00–0.00 |  |  |
|  | February | 17 | 0–1 | 0.00–0.30 | 0–0 | 0.00–0.00 |  |  |
|  | March | 17 | 0–1 | 0.00–2.31 | 0–0 | 0.00–0.00 |  |  |
|  | April | 17 | 0–1 | 0.00–0.48 | 0–0 | 0.00–0.00 |  |  |
|  | May | 19 | 0–3 | 0.00–14.20 | 0–0 | 0.00–0.00 |  |  |
|  | June | 16 | 0–0 | 0.00–0.00 | 0–1 | 0.00–2.09 |  |  |
|  | (f) LTCI care beds |  |  |  |  |  |  |  |
|  | January | 296 | 59–108 | 31.21–57.43 | 0–0 | 0.00–0.00 | * |  |
|  | February | 69 | 0–0 | 0.00–0.00 | 81–125 | 41.74–64.35 |  | ** |
|  | March | 79 | 0–0 | 0.00–0.00 | 66–112 | 34.36–58.41 |  | ** |
|  | April | 293 | 37–95 | 18.60–47.32 | 0–0 | 0.00–0.00 | * |  |
|  | May | 212 | 0–19 | 0.00–9.75 | 0–0 | 0.00–0.00 |  |  |
|  | June | 274 | 5–74 | 2.49–36.40 | 0–0 | 0.00–0.00 | * |  |
| Okayama | (1) Average number of outpatients per day at hospitals |  |  |  |  |  |  |  |
|  | General hospitals |  |  |  |  |  |  |  |
|  | January | 23160 | 0–0 | 0.00–0.00 | 0–709 | 0.00–2.97 |  |  |
|  | February | 23009 | 0–0 | 0.00–0.00 | 0–1066 | 0.00–4.43 |  |  |
|  | March | 22699 | 0–0 | 0.00–0.00 | 0–1651 | 0.00–6.78 |  |  |
|  | April | 21046 | 0–0 | 0.00–0.00 | 1135–2732 | 4.77–11.49 |  | ** |
|  | May | 18327 | 0–0 | 0.00–0.00 | 4002–5606 | 16.72–23.42 |  | ** |
|  | June | 22362 | 0–0 | 0.00–0.00 | 122–1832 | 0.50–7.57 |  | ** |
|  | Psychiatric hospitals |  |  |  |  |  |  |  |
|  | January | 1113 | 0–0 | 0.00–0.00 | 5–100 | 0.41–8.21 |  | ** |
|  | February | 1154 | 0–0 | 0.00–0.00 | 0–79 | 0.00–6.36 |  |  |
|  | March | 1160 | 0–0 | 0.00–0.00 | 8–101 | 0.63–7.99 |  | ** |
|  | April | 1176 | 0–0 | 0.00–0.00 | 0–58 | 0.00–4.64 |  |  |
|  | May | 1027 | 0–0 | 0.00–0.00 | 113–208 | 9.15–16.80 |  | ** |
|  | June | 1194 | 0–0 | 0.00–0.00 | 0–40 | 0.00–3.23 |  |  |
|  | (2) Average number of hospitalized patients per day |  |  |  |  |  |  |  |
|  | (b) Psychiatric care beds |  |  |  |  |  |  |  |
|  | January | 4251 | 0–12 | 0.00–0.27 | 0–0 | 0.00–0.00 |  |  |
|  | February | 4262 | 0–16 | 0.00–0.37 | 0–0 | 0.00–0.00 |  |  |
|  | March | 3997 | 0–0 | 0.00–0.00 | 139–267 | 3.26–6.25 |  | ** |
|  | April | 4168 | 0–0 | 0.00–0.00 | 0–103 | 0.00–2.39 |  |  |
|  | May | 4176 | 0–0 | 0.00–0.00 | 0–107 | 0.00–2.49 |  |  |
|  | June | 4228 | 0–0 | 0.00–0.00 | 0–66 | 0.00–1.53 |  |  |
|  | (c) Tuberculosis care beds |  |  |  |  |  |  |  |
|  | January | 50 | 0–0 | 0.00–0.00 | 0–1 | 0.00–0.14 |  |  |
|  | February | 44 | 0–0 | 0.00–0.00 | 0–7 | 0.00–12.20 |  |  |
|  | March | 38 | 0–0 | 0.00–0.00 | 0–12 | 0.00–23.70 |  |  |
|  | April | 39 | 0–0 | 0.00–0.00 | 0–8 | 0.00–17.02 |  |  |
|  | May | 41 | 0–0 | 0.00–0.00 | 0–4 | 0.00–8.33 |  |  |
|  | June | 42 | 0–0 | 0.00–0.00 | 0–2 | 0.00–3.03 |  |  |
|  | (d) Long-term care beds |  |  |  |  |  |  |  |
|  | January | 3730 | 0–0 | 0.00–0.00 | 19–148 | 0.49–3.80 |  | ** |
|  | February | 3717 | 0–0 | 0.00–0.00 | 65–187 | 1.67–4.77 |  | ** |
|  | March | 3657 | 0–0 | 0.00–0.00 | 120–243 | 3.08–6.22 |  | ** |
|  | April | 3583 | 0–0 | 0.00–0.00 | 149–281 | 3.86–7.26 |  | ** |
|  | May | 3521 | 0–0 | 0.00–0.00 | 178–304 | 4.65–7.93 |  | ** |
|  | June | 3498 | 0–0 | 0.00–0.00 | 178–306 | 4.68–8.04 |  | ** |
|  | (e) General beds |  |  |  |  |  |  |  |
|  | January | 12929 | 0–0 | 0.00–0.00 | 0–397 | 0.00–2.98 |  |  |
|  | February | 13172 | 0–0 | 0.00–0.00 | 0–322 | 0.00–2.38 |  |  |
|  | March | 12718 | 0–0 | 0.00–0.00 | 322–763 | 2.39–5.65 |  | ** |
|  | April | 11892 | 0–0 | 0.00–0.00 | 790–1282 | 6.00–9.73 |  | ** |
|  | May | 11327 | 0–0 | 0.00–0.00 | 1315–1711 | 10.09–13.12 |  | ** |
|  | June | 11780 | 0–0 | 0.00–0.00 | 863–1281 | 6.61–9.81 |  | ** |
|  | (f) LTCI care beds |  |  |  |  |  |  |  |
|  | January | 323 | 0–0 | 0.00–0.00 | 0–37 | 0.00–10.23 |  |  |
|  | February | 320 | 0–0 | 0.00–0.00 | 0–35 | 0.00–9.78 |  |  |
|  | March | 314 | 0–0 | 0.00–0.00 | 0–30 | 0.00–8.51 |  |  |
|  | April | 281 | 0–0 | 0.00–0.00 | 13–51 | 3.92–15.28 |  | ** |
|  | May | 241 | 0–0 | 0.00–0.00 | 44–81 | 13.68–25.09 |  | ** |
|  | June | 236 | 0–0 | 0.00–0.00 | 46–83 | 14.44–25.92 |  | ** |
|  | (3) Average length of hospital stays per patient |  |  |  |  |  |  |  |
|  | (b) Psychiatric care beds |  |  |  |  |  |  |  |
|  | January | 257 | 0–18 | 0.00–7.17 | 0–0 | 0.00–0.00 |  |  |
|  | February | 241 | 0–7 | 0.00–2.68 | 0–0 | 0.00–0.00 |  |  |
|  | March | 282 | 25–55 | 10.65–24.17 | 0–0 | 0.00–0.00 | * |  |
|  | April | 230 | 0–1 | 0.00–0.30 | 0–0 | 0.00–0.00 |  |  |
|  | May | 267 | 5–36 | 2.12–15.51 | 0–0 | 0.00–0.00 | * |  |
|  | June | 236 | 0–7 | 0.00–2.87 | 0–0 | 0.00–0.00 |  |  |
|  | (c) Tuberculosis care beds |  |  |  |  |  |  |  |
|  | January | 87 | 0–0 | 0.00–0.00 | 0–18 | 0.00–16.87 |  |  |
|  | February | 69 | 0–0 | 0.00–0.00 | 4–38 | 3.55–35.41 |  | ** |
|  | March | 95 | 0–0 | 0.00–0.00 | 0–10 | 0.00–9.10 |  |  |
|  | April | 45 | 0–0 | 0.00–0.00 | 32–65 | 28.31–59.12 |  | ** |
|  | May | 83 | 0–0 | 0.00–0.00 | 0–16 | 0.00–16.09 |  |  |
|  | June | 113 | 0–14 | 0.00–13.60 | 0–0 | 0.00–0.00 |  |  |
|  | (d) Long-term care beds |  |  |  |  |  |  |  |
|  | January | 116 | 0–5 | 0.00–3.59 | 0–0 | 0.00–0.00 |  |  |
|  | February | 118 | 0–7 | 0.00–6.20 | 0–0 | 0.00–0.00 |  |  |
|  | March | 117 | 0–7 | 0.00–6.18 | 0–0 | 0.00–0.00 |  |  |
|  | April | 119 | 0–4 | 0.00–3.20 | 0–0 | 0.00–0.00 |  |  |
|  | May | 133 | 0–16 | 0.00–13.50 | 0–0 | 0.00–0.00 |  |  |
|  | June | 120 | 0–1 | 0.00–0.17 | 0–0 | 0.00–0.00 |  |  |
|  | (e) General beds |  |  |  |  |  |  |  |
|  | January | 18 | 0–1 | 0.00–3.50 | 0–0 | 0.00–0.00 |  |  |
|  | February | 17 | 0–0 | 0.00–0.00 | 0–1 | 0.00–0.61 |  |  |
|  | March | 17 | 0–1 | 0.00–1.67 | 0–0 | 0.00–0.00 |  |  |
|  | April | 18 | 0–1 | 0.00–2.90 | 0–0 | 0.00–0.00 |  |  |
|  | May | 19 | 0–3 | 0.00–13.34 | 0–0 | 0.00–0.00 |  |  |
|  | June | 17 | 0–1 | 0.00–1.47 | 0–0 | 0.00–0.00 |  |  |
|  | (f) LTCI care beds |  |  |  |  |  |  |  |
|  | January | 231 | 0–28 | 0.00–13.65 | 0–0 | 0.00–0.00 |  |  |
|  | February | 221 | 0–17 | 0.00–8.17 | 0–0 | 0.00–0.00 |  |  |
|  | March | 221 | 0–35 | 0.00–18.19 | 0–0 | 0.00–0.00 |  |  |
|  | April | 198 | 0–12 | 0.00–6.25 | 0–0 | 0.00–0.00 |  |  |
|  | May | 213 | 0–33 | 0.00–17.86 | 0–0 | 0.00–0.00 |  |  |
|  | June | 228 | 0–44 | 0.00–23.52 | 0–0 | 0.00–0.00 |  |  |
| Hiroshima | (1) Average number of outpatients per day at hospitals |  |  |  |  |  |  |  |
|  | General hospitals |  |  |  |  |  |  |  |
|  | January | 26737 | 0–0 | 0.00–0.00 | 0–1144 | 0.00–4.10 |  |  |
|  | February | 26802 | 0–0 | 0.00–0.00 | 0–1472 | 0.00–5.20 |  |  |
|  | March | 26867 | 0–0 | 0.00–0.00 | 0–1784 | 0.00–6.22 |  |  |
|  | April | 24258 | 0–0 | 0.00–0.00 | 1772–3686 | 6.34–13.19 |  | ** |
|  | May | 20905 | 0–0 | 0.00–0.00 | 5237–7215 | 18.62–25.66 |  | ** |
|  | June | 26290 | 0–0 | 0.00–0.00 | 8–1978 | 0.03–7.00 |  | ** |
|  | Psychiatric hospitals |  |  |  |  |  |  |  |
|  | January | 1827 | 0–0 | 0.00–0.00 | 0–7 | 0.00–0.36 |  |  |
|  | February | 1921 | 0–63 | 0.00–3.38 | 0–0 | 0.00–0.00 |  |  |
|  | March | 1889 | 0–0 | 0.00–0.00 | 0–34 | 0.00–1.72 |  |  |
|  | April | 1816 | 0–0 | 0.00–0.00 | 0–68 | 0.00–3.56 |  |  |
|  | May | 1623 | 0–0 | 0.00–0.00 | 141–267 | 7.46–14.10 |  | ** |
|  | June | 1913 | 0–27 | 0.00–1.43 | 0–0 | 0.00–0.00 |  |  |
|  | (2) Average number of hospitalized patients per day |  |  |  |  |  |  |  |
|  | (b) Psychiatric care beds |  |  |  |  |  |  |  |
|  | January | 7634 | 0–0 | 0.00–0.00 | 0–91 | 0.00–1.17 |  |  |
|  | February | 7649 | 0–0 | 0.00–0.00 | 0–87 | 0.00–1.12 |  |  |
|  | March | 7658 | 0–0 | 0.00–0.00 | 0–83 | 0.00–1.06 |  |  |
|  | April | 7602 | 0–0 | 0.00–0.00 | 0–122 | 0.00–1.58 |  |  |
|  | May | 7576 | 0–0 | 0.00–0.00 | 0–144 | 0.00–1.86 |  |  |
|  | June | 7654 | 0–0 | 0.00–0.00 | 0–79 | 0.00–1.01 |  |  |
|  | (c) Tuberculosis care beds |  |  |  |  |  |  |  |
|  | January | 22 | 0–0 | 0.00–0.00 | 0–2 | 0.00–6.38 |  |  |
|  | February | 19 | 0–0 | 0.00–0.00 | 0–6 | 0.00–23.48 |  |  |
|  | March | 25 | 0–0 | 0.00–0.00 | 0–1 | 0.00–2.58 |  |  |
|  | April | 27 | 0–1 | 0.00–2.69 | 0–0 | 0.00–0.00 |  |  |
|  | May | 25 | 0–0 | 0.00–0.00 | 0–3 | 0.00–7.54 |  |  |
|  | June | 28 | 0–1 | 0.00–3.62 | 0–0 | 0.00–0.00 |  |  |
|  | (d) Long-term care beds |  |  |  |  |  |  |  |
|  | January | 7834 | 0–0 | 0.00–0.00 | 0–202 | 0.00–2.50 |  |  |
|  | February | 7888 | 0–0 | 0.00–0.00 | 0–151 | 0.00–1.87 |  |  |
|  | March | 7799 | 0–0 | 0.00–0.00 | 0–218 | 0.00–2.72 |  |  |
|  | April | 7239 | 0–0 | 0.00–0.00 | 407–712 | 5.12–8.95 |  | ** |
|  | May | 7118 | 0–0 | 0.00–0.00 | 468–769 | 5.93–9.74 |  | ** |
|  | June | 7095 | 0–0 | 0.00–0.00 | 420–723 | 5.37–9.24 |  | ** |
|  | (e) General beds |  |  |  |  |  |  |  |
|  | January | 16281 | 0–0 | 0.00–0.00 | 0–473 | 0.00–2.82 |  |  |
|  | February | 16613 | 0–0 | 0.00–0.00 | 0–244 | 0.00–1.45 |  |  |
|  | March | 16010 | 0–0 | 0.00–0.00 | 268–764 | 1.60–4.55 |  | ** |
|  | April | 15057 | 0–0 | 0.00–0.00 | 783–1345 | 4.77–8.20 |  | ** |
|  | May | 14194 | 0–0 | 0.00–0.00 | 1596–2015 | 9.85–12.43 |  | ** |
|  | June | 14900 | 0–0 | 0.00–0.00 | 862–1331 | 5.31–8.20 |  | ** |
|  | (f) LTCI care beds |  |  |  |  |  |  |  |
|  | January | 1094 | 0–0 | 0.00–0.00 | 27–274 | 1.97–19.98 |  | ** |
|  | February | 1101 | 0–0 | 0.00–0.00 | 0–241 | 0.00–17.94 |  |  |
|  | March | 1076 | 0–0 | 0.00–0.00 | 0–241 | 0.00–18.28 |  |  |
|  | April | 683 | 0–0 | 0.00–0.00 | 357–606 | 27.70–47.00 |  | ** |
|  | May | 681 | 0–0 | 0.00–0.00 | 336–583 | 26.59–46.10 |  | ** |
|  | June | 670 | 0–0 | 0.00–0.00 | 303–549 | 24.87–45.00 |  | ** |
|  | (3) Average length of hospital stays per patient |  |  |  |  |  |  |  |
|  | (b) Psychiatric care beds |  |  |  |  |  |  |  |
|  | January | 324 | 0–16 | 0.00–4.93 | 0–0 | 0.00–0.00 |  |  |
|  | February | 314 | 0–10 | 0.00–3.20 | 0–0 | 0.00–0.00 |  |  |
|  | March | 305 | 0–7 | 0.00–2.26 | 0–0 | 0.00–0.00 |  |  |
|  | April | 339 | 4–40 | 1.30–13.01 | 0–0 | 0.00–0.00 | * |  |
|  | May | 335 | 4–39 | 1.35–12.97 | 0–0 | 0.00–0.00 | * |  |
|  | June | 300 | 0–6 | 0.00–1.94 | 0–0 | 0.00–0.00 |  |  |
|  | (c) Tuberculosis care beds |  |  |  |  |  |  |  |
|  | January | 62 | 0–7 | 0.00–12.72 | 0–0 | 0.00–0.00 |  |  |
|  | February | 48 | 0–0 | 0.00–0.00 | 0–11 | 0.00–17.87 |  |  |
|  | March | 58 | 0–0 | 0.00–0.00 | 0–3 | 0.00–3.84 |  |  |
|  | April | 63 | 0–0 | 0.00–0.00 | 0–2 | 0.00–1.82 |  |  |
|  | May | 49 | 0–0 | 0.00–0.00 | 0–8 | 0.00–13.74 |  |  |
|  | June | 67 | 0–9 | 0.00–14.48 | 0–0 | 0.00–0.00 |  |  |
|  | (d) Long-term care beds |  |  |  |  |  |  |  |
|  | January | 123 | 0–3 | 0.00–2.26 | 0–0 | 0.00–0.00 |  |  |
|  | February | 123 | 0–4 | 0.00–2.60 | 0–0 | 0.00–0.00 |  |  |
|  | March | 111 | 0–0 | 0.00–0.00 | 0–7 | 0.00–5.33 |  |  |
|  | April | 122 | 0–4 | 0.00–2.75 | 0–0 | 0.00–0.00 |  |  |
|  | May | 139 | 0–20 | 0.00–16.08 | 0–0 | 0.00–0.00 |  |  |
|  | June | 121 | 0–0 | 0.00–0.00 | 0–1 | 0.00–0.15 |  |  |
|  | (e) General beds |  |  |  |  |  |  |  |
|  | January | 17 | 0–1 | 0.00–1.89 | 0–0 | 0.00–0.00 |  |  |
|  | February | 17 | 0–0 | 0.00–0.00 | 0–1 | 0.00–0.76 |  |  |
|  | March | 17 | 0–0 | 0.00–0.00 | 0–1 | 0.00–0.40 |  |  |
|  | April | 17 | 0–1 | 0.00–1.48 | 0–0 | 0.00–0.00 |  |  |
|  | May | 19 | 0–3 | 0.00–14.12 | 0–0 | 0.00–0.00 |  |  |
|  | June | 16 | 0–0 | 0.00–0.00 | 0–1 | 0.00–0.06 |  |  |
|  | (f) LTCI care beds |  |  |  |  |  |  |  |
|  | January | 251 | 0–0 | 0.00–0.00 | 0–19 | 0.00–6.83 |  |  |
|  | February | 236 | 0–0 | 0.00–0.00 | 0–26 | 0.00–9.63 |  |  |
|  | March | 95 | 0–0 | 0.00–0.00 | 110–154 | 44.27–61.70 |  | ** |
|  | April | 202 | 0–0 | 0.00–0.00 | 2–48 | 0.80–19.17 |  | ** |
|  | May | 285 | 0–31 | 0.00–12.11 | 0–0 | 0.00–0.00 |  |  |
|  | June | 237 | 0–0 | 0.00–0.00 | 0–24 | 0.00–9.08 |  |  |
| Yamaguchi | (1) Average number of outpatients per day at hospitals |  |  |  |  |  |  |  |
|  | General hospitals |  |  |  |  |  |  |  |
|  | January | 13303 | 0–0 | 0.00–0.00 | 0–388 | 0.00–2.83 |  |  |
|  | February | 13197 | 0–0 | 0.00–0.00 | 0–599 | 0.00–4.34 |  |  |
|  | March | 13226 | 0–0 | 0.00–0.00 | 0–741 | 0.00–5.31 |  |  |
|  | April | 12387 | 0–0 | 0.00–0.00 | 344–1246 | 2.52–9.14 |  | ** |
|  | May | 10570 | 0–0 | 0.00–0.00 | 2198–3167 | 16.00–23.05 |  | ** |
|  | June | 12965 | 0–0 | 0.00–0.00 | 0–940 | 0.00–6.76 |  |  |
|  | Psychiatric hospitals |  |  |  |  |  |  |  |
|  | January | 1085 | 0–0 | 0.00–0.00 | 0–43 | 0.00–3.80 |  |  |
|  | February | 1140 | 0–0 | 0.00–0.00 | 0–9 | 0.00–0.74 |  |  |
|  | March | 1123 | 0–0 | 0.00–0.00 | 0–65 | 0.00–5.47 |  |  |
|  | April | 1087 | 0–0 | 0.00–0.00 | 0–79 | 0.00–6.71 |  |  |
|  | May | 978 | 0–0 | 0.00–0.00 | 106–195 | 9.04–16.61 |  | ** |
|  | June | 1147 | 0–0 | 0.00–0.00 | 0–25 | 0.00–2.11 |  |  |
|  | (2) Average number of hospitalized patients per day |  |  |  |  |  |  |  |
|  | (b) Psychiatric care beds |  |  |  |  |  |  |  |
|  | January | 5222 | 0–0 | 0.00–0.00 | 0–9 | 0.00–0.15 |  |  |
|  | February | 5209 | 0–0 | 0.00–0.00 | 0–17 | 0.00–0.32 |  |  |
|  | March | 5203 | 0–0 | 0.00–0.00 | 0–25 | 0.00–0.47 |  |  |
|  | April | 5190 | 0–0 | 0.00–0.00 | 0–39 | 0.00–0.73 |  |  |
|  | May | 5196 | 0–0 | 0.00–0.00 | 0–38 | 0.00–0.71 |  |  |
|  | June | 5201 | 0–0 | 0.00–0.00 | 0–39 | 0.00–0.73 |  |  |
|  | (c) Tuberculosis care beds |  |  |  |  |  |  |  |
|  | January | 10 | 0–0 | 0.00–0.00 | 0–1 | 0.00–1.87 |  |  |
|  | February | 10 | 0–1 | 0.00–5.74 | 0–0 | 0.00–0.00 |  |  |
|  | March | 9 | 0–1 | 0.00–7.02 | 0–0 | 0.00–0.00 |  |  |
|  | April | 10 | 0–3 | 0.00–25.93 | 0–0 | 0.00–0.00 |  |  |
|  | May | 11 | 0–3 | 0.00–34.80 | 0–0 | 0.00–0.00 |  |  |
|  | June | 11 | 0–2 | 0.00–18.60 | 0–0 | 0.00–0.00 |  |  |
|  | (d) Long-term care beds |  |  |  |  |  |  |  |
|  | January | 7497 | 0–0 | 0.00–0.00 | 78–324 | 1.00–4.14 |  | ** |
|  | February | 7547 | 0–0 | 0.00–0.00 | 34–279 | 0.43–3.56 |  | ** |
|  | March | 7374 | 0–0 | 0.00–0.00 | 221–455 | 2.82–5.81 |  | ** |
|  | April | 7103 | 0–0 | 0.00–0.00 | 367–615 | 4.76–7.97 |  | ** |
|  | May | 6619 | 0–0 | 0.00–0.00 | 812–1050 | 10.59–13.68 |  | ** |
|  | June | 6586 | 0–0 | 0.00–0.00 | 824–1054 | 10.79–13.79 |  | ** |
|  | (e) General beds |  |  |  |  |  |  |  |
|  | January | 8929 | 0–0 | 0.00–0.00 | 0–63 | 0.00–0.69 |  |  |
|  | February | 8996 | 0–0 | 0.00–0.00 | 0–61 | 0.00–0.67 |  |  |
|  | March | 8686 | 0–0 | 0.00–0.00 | 70–335 | 0.78–3.70 |  | ** |
|  | April | 8151 | 0–0 | 0.00–0.00 | 373–665 | 4.23–7.54 |  | ** |
|  | May | 7603 | 0–0 | 0.00–0.00 | 918–1129 | 10.51–12.93 |  | ** |
|  | June | 8017 | 0–0 | 0.00–0.00 | 477–712 | 5.46–8.15 |  | ** |
|  | (f) LTCI care beds |  |  |  |  |  |  |  |
|  | January | 900 | 0–0 | 0.00–0.00 | 0–135 | 0.00–12.98 |  |  |
|  | February | 896 | 0–0 | 0.00–0.00 | 0–113 | 0.00–11.11 |  |  |
|  | March | 746 | 0–0 | 0.00–0.00 | 47–233 | 4.80–23.74 |  | ** |
|  | April | 144 | 0–0 | 0.00–0.00 | 622–808 | 65.36–84.87 |  | ** |
|  | May | 145 | 0–0 | 0.00–0.00 | 595–780 | 64.34–84.32 |  | ** |
|  | June | 146 | 0–0 | 0.00–0.00 | 583–766 | 63.96–83.98 |  | ** |
|  | (3) Average length of hospital stays per patient |  |  |  |  |  |  |  |
|  | (b) Psychiatric care beds |  |  |  |  |  |  |  |
|  | January | 458 | 0–17 | 0.00–3.69 | 0–0 | 0.00–0.00 |  |  |
|  | February | 450 | 0–17 | 0.00–3.88 | 0–0 | 0.00–0.00 |  |  |
|  | March | 443 | 0–13 | 0.00–2.90 | 0–0 | 0.00–0.00 |  |  |
|  | April | 483 | 6–48 | 1.33–10.94 | 0–0 | 0.00–0.00 | * |  |
|  | May | 489 | 3–46 | 0.63–10.21 | 0–0 | 0.00–0.00 | * |  |
|  | June | 428 | 0–0 | 0.00–0.00 | 0–16 | 0.00–3.59 |  |  |
|  | (c) Tuberculosis care beds |  |  |  |  |  |  |  |
|  | January | 69 | 0–0 | 0.00–0.00 | 0–15 | 0.00–17.12 |  |  |
|  | February | 110 | 0–16 | 0.00–16.25 | 0–0 | 0.00–0.00 |  |  |
|  | March | 62 | 0–0 | 0.00–0.00 | 0–21 | 0.00–25.34 |  |  |
|  | April | 144 | 0–58 | 0.00–66.51 | 0–0 | 0.00–0.00 |  |  |
|  | May | 178 | 31–98 | 38.73–122.41 | 0–0 | 0.00–0.00 | * |  |
|  | June | 164 | 8–78 | 9.25–89.70 | 0–0 | 0.00–0.00 | * |  |
|  | (d) Long-term care beds |  |  |  |  |  |  |  |
|  | January | 153 | 0–14 | 0.00–9.52 | 0–0 | 0.00–0.00 |  |  |
|  | February | 143 | 0–5 | 0.00–3.13 | 0–0 | 0.00–0.00 |  |  |
|  | March | 145 | 0–8 | 0.00–5.73 | 0–0 | 0.00–0.00 |  |  |
|  | April | 141 | 0–1 | 0.00–0.11 | 0–0 | 0.00–0.00 |  |  |
|  | May | 162 | 0–19 | 0.00–12.70 | 0–0 | 0.00–0.00 |  |  |
|  | June | 136 | 0–0 | 0.00–0.00 | 0–9 | 0.00–5.79 |  |  |
|  | (e) General beds |  |  |  |  |  |  |  |
|  | January | 18 | 0–1 | 0.00–3.07 | 0–0 | 0.00–0.00 |  |  |
|  | February | 18 | 0–1 | 0.00–0.83 | 0–0 | 0.00–0.00 |  |  |
|  | March | 18 | 0–1 | 0.00–2.24 | 0–0 | 0.00–0.00 |  |  |
|  | April | 18 | 0–1 | 0.00–1.95 | 0–0 | 0.00–0.00 |  |  |
|  | May | 20 | 0–3 | 0.00–14.25 | 0–0 | 0.00–0.00 |  |  |
|  | June | 17 | 0–1 | 0.00–0.39 | 0–0 | 0.00–0.00 |  |  |
|  | (f) LTCI care beds |  |  |  |  |  |  |  |
|  | January | 715 | 50–223 | 9.97–45.19 | 0–0 | 0.00–0.00 | * |  |
|  | February | 244 | 0–0 | 0.00–0.00 | 103–250 | 20.71–50.53 |  | ** |
|  | March | 498 | 0–32 | 0.00–6.73 | 0–0 | 0.00–0.00 |  |  |
|  | April | 145 | 0–0 | 0.00–0.00 | 199–358 | 39.56–71.20 |  | ** |
|  | May | 560 | 0–27 | 0.00–4.98 | 0–0 | 0.00–0.00 |  |  |
|  | June | 417 | 0–0 | 0.00–0.00 | 0–152 | 0.00–26.66 |  |  |
| Tokushima | (1) Average number of outpatients per day at hospitals |  |  |  |  |  |  |  |
|  | General hospitals |  |  |  |  |  |  |  |
|  | January | 9559 | 0–0 | 0.00–0.00 | 1–592 | 0.01–5.83 |  | ** |
|  | February | 9763 | 0–0 | 0.00–0.00 | 0–436 | 0.00–4.27 |  |  |
|  | March | 9590 | 0–0 | 0.00–0.00 | 75–691 | 0.73–6.72 |  | ** |
|  | April | 9283 | 0–0 | 0.00–0.00 | 181–810 | 1.79–8.02 |  | ** |
|  | May | 8206 | 0–0 | 0.00–0.00 | 1328–1928 | 13.11–19.02 |  | ** |
|  | June | 9823 | 0–0 | 0.00–0.00 | 0–368 | 0.00–3.61 |  |  |
|  | Psychiatric hospitals |  |  |  |  |  |  |  |
|  | January | 506 | 0–0 | 0.00–0.00 | 0–19 | 0.00–3.51 |  |  |
|  | February | 534 | 0–6 | 0.00–0.96 | 0–0 | 0.00–0.00 |  |  |
|  | March | 534 | 0–0 | 0.00–0.00 | 0–8 | 0.00–1.38 |  |  |
|  | April | 548 | 0–18 | 0.00–3.22 | 0–0 | 0.00–0.00 |  |  |
|  | May | 496 | 0–0 | 0.00–0.00 | 0–38 | 0.00–7.07 |  |  |
|  | June | 565 | 0–32 | 0.00–5.88 | 0–0 | 0.00–0.00 |  |  |
|  | (2) Average number of hospitalized patients per day |  |  |  |  |  |  |  |
|  | (b) Psychiatric care beds |  |  |  |  |  |  |  |
|  | January | 3078 | 0–3 | 0.00–0.09 | 0–0 | 0.00–0.00 |  |  |
|  | February | 3095 | 0–20 | 0.00–0.64 | 0–0 | 0.00–0.00 |  |  |
|  | March | 3076 | 0–3 | 0.00–0.08 | 0–0 | 0.00–0.00 |  |  |
|  | April | 3049 | 0–0 | 0.00–0.00 | 0–21 | 0.00–0.67 |  |  |
|  | May | 3048 | 0–0 | 0.00–0.00 | 0–23 | 0.00–0.74 |  |  |
|  | June | 3070 | 0–0 | 0.00–0.00 | 0–8 | 0.00–0.23 |  |  |
|  | (c) Tuberculosis care beds |  |  |  |  |  |  |  |
|  | January | 14 | 0–0 | 0.00–0.00 | 0–3 | 0.00–16.93 |  |  |
|  | February | 15 | 0–0 | 0.00–0.00 | 0–1 | 0.00–5.74 |  |  |
|  | March | 15 | 0–0 | 0.00–0.00 | 0–1 | 0.00–3.94 |  |  |
|  | April | 13 | 0–0 | 0.00–0.00 | 0–4 | 0.00–19.43 |  |  |
|  | May | 13 | 0–0 | 0.00–0.00 | 0–4 | 0.00–19.57 |  |  |
|  | June | 15 | 0–0 | 0.00–0.00 | 0–1 | 0.00–3.74 |  |  |
|  | (d) Long-term care beds |  |  |  |  |  |  |  |
|  | January | 3310 | 0–0 | 0.00–0.00 | 46–161 | 1.33–4.63 |  | ** |
|  | February | 3332 | 0–0 | 0.00–0.00 | 29–145 | 0.83–4.14 |  | ** |
|  | March | 3316 | 0–0 | 0.00–0.00 | 35–150 | 1.01–4.31 |  | ** |
|  | April | 3161 | 0–0 | 0.00–0.00 | 164–279 | 4.77–8.09 |  | ** |
|  | May | 3142 | 0–0 | 0.00–0.00 | 156–270 | 4.57–7.89 |  | ** |
|  | June | 3137 | 0–0 | 0.00–0.00 | 141–255 | 4.16–7.51 |  | ** |
|  | (e) General beds |  |  |  |  |  |  |  |
|  | January | 4892 | 0–0 | 0.00–0.00 | 0–71 | 0.00–1.42 |  |  |
|  | February | 4962 | 0–0 | 0.00–0.00 | 0–47 | 0.00–0.92 |  |  |
|  | March | 4840 | 0–0 | 0.00–0.00 | 26–168 | 0.52–3.35 |  | ** |
|  | April | 4641 | 0–0 | 0.00–0.00 | 80–239 | 1.64–4.89 |  | ** |
|  | May | 4441 | 0–0 | 0.00–0.00 | 261–396 | 5.40–8.18 |  | ** |
|  | June | 4664 | 0–0 | 0.00–0.00 | 61–198 | 1.25–4.07 |  | ** |
|  | (f) LTCI care beds |  |  |  |  |  |  |  |
|  | January | 513 | 0–0 | 0.00–0.00 | 96–145 | 14.60–22.00 |  | ** |
|  | February | 506 | 0–0 | 0.00–0.00 | 101–151 | 15.39–22.88 |  | ** |
|  | March | 505 | 0–0 | 0.00–0.00 | 98–148 | 15.02–22.60 |  | ** |
|  | April | 407 | 0–0 | 0.00–0.00 | 192–242 | 29.62–37.21 |  | ** |
|  | May | 403 | 0–0 | 0.00–0.00 | 191–240 | 29.74–37.26 |  | ** |
|  | June | 396 | 0–0 | 0.00–0.00 | 192–240 | 30.19–37.72 |  | ** |
|  | (3) Average length of hospital stays per patient |  |  |  |  |  |  |  |
|  | (b) Psychiatric care beds |  |  |  |  |  |  |  |
|  | January | 353 | 0–12 | 0.00–3.33 | 0–0 | 0.00–0.00 |  |  |
|  | February | 326 | 0–0 | 0.00–0.00 | 0–9 | 0.00–2.56 |  |  |
|  | March | 338 | 0–14 | 0.00–4.12 | 0–0 | 0.00–0.00 |  |  |
|  | April | 339 | 0–11 | 0.00–3.33 | 0–0 | 0.00–0.00 |  |  |
|  | May | 383 | 26–66 | 8.08–20.80 | 0–0 | 0.00–0.00 | * |  |
|  | June | 322 | 0–4 | 0.00–1.19 | 0–0 | 0.00–0.00 |  |  |
|  | (c) Tuberculosis care beds |  |  |  |  |  |  |  |
|  | January | 66 | 0–0 | 0.00–0.00 | 0–18 | 0.00–21.34 |  |  |
|  | February | 96 | 0–20 | 0.00–24.62 | 0–0 | 0.00–0.00 |  |  |
|  | March | 93 | 0–24 | 0.00–34.90 | 0–0 | 0.00–0.00 |  |  |
|  | April | 134 | 21–65 | 29.01–91.91 | 0–0 | 0.00–0.00 | * |  |
|  | May | 80 | 0–11 | 0.00–14.23 | 0–0 | 0.00–0.00 |  |  |
|  | June | 73 | 0–0 | 0.00–0.00 | 0–3 | 0.00–3.65 |  |  |
|  | (d) Long-term care beds |  |  |  |  |  |  |  |
|  | January | 110 | 0–0 | 0.00–0.00 | 0–3 | 0.00–1.86 |  |  |
|  | February | 109 | 0–0 | 0.00–0.00 | 0–5 | 0.00–3.69 |  |  |
|  | March | 108 | 0–0 | 0.00–0.00 | 0–3 | 0.00–2.61 |  |  |
|  | April | 105 | 0–0 | 0.00–0.00 | 0–8 | 0.00–6.28 |  |  |
|  | May | 123 | 0–11 | 0.00–9.62 | 0–0 | 0.00–0.00 |  |  |
|  | June | 110 | 0–0 | 0.00–0.00 | 0–2 | 0.00–1.23 |  |  |
|  | (e) General beds |  |  |  |  |  |  |  |
|  | January | 19 | 0–1 | 0.00–4.42 | 0–0 | 0.00–0.00 |  |  |
|  | February | 18 | 0–1 | 0.00–1.37 | 0–0 | 0.00–0.00 |  |  |
|  | March | 18 | 0–1 | 0.00–1.44 | 0–0 | 0.00–0.00 |  |  |
|  | April | 18 | 0–1 | 0.00–2.83 | 0–0 | 0.00–0.00 |  |  |
|  | May | 20 | 0–3 | 0.00–11.83 | 0–0 | 0.00–0.00 |  |  |
|  | June | 18 | 0–1 | 0.00–1.31 | 0–0 | 0.00–0.00 |  |  |
|  | (f) LTCI care beds |  |  |  |  |  |  |  |
|  | January | 295 | 0–0 | 0.00–0.00 | 0–34 | 0.00–10.18 |  |  |
|  | February | 377 | 0–50 | 0.00–15.11 | 0–0 | 0.00–0.00 |  |  |
|  | March | 344 | 0–34 | 0.00–10.66 | 0–0 | 0.00–0.00 |  |  |
|  | April | 176 | 0–0 | 0.00–0.00 | 60–128 | 19.65–42.04 |  | ** |
|  | May | 520 | 112–200 | 34.71–62.25 | 0–0 | 0.00–0.00 | * |  |
|  | June | 495 | 85–170 | 25.86–51.81 | 0–0 | 0.00–0.00 | * |  |
| Kagawa | (1) Average number of outpatients per day at hospitals |  |  |  |  |  |  |  |
|  | General hospitals |  |  |  |  |  |  |  |
|  | January | 12436 | 0–0 | 0.00–0.00 | 0–403 | 0.00–3.14 |  |  |
|  | February | 12450 | 0–0 | 0.00–0.00 | 0–500 | 0.00–3.86 |  |  |
|  | March | 12282 | 0–0 | 0.00–0.00 | 0–808 | 0.00–6.17 |  |  |
|  | April | 11379 | 0–0 | 0.00–0.00 | 613–1396 | 4.80–10.93 |  | ** |
|  | May | 9829 | 0–0 | 0.00–0.00 | 2191–3036 | 17.03–23.60 |  | ** |
|  | June | 12140 | 0–0 | 0.00–0.00 | 0–870 | 0.00–6.68 |  |  |
|  | Psychiatric hospitals |  |  |  |  |  |  |  |
|  | January | 813 | 0–0 | 0.00–0.00 | 0–22 | 0.00–2.61 |  |  |
|  | February | 847 | 0–1 | 0.00–0.01 | 0–0 | 0.00–0.00 |  |  |
|  | March | 859 | 0–0 | 0.00–0.00 | 0–16 | 0.00–1.76 |  |  |
|  | April | 828 | 0–0 | 0.00–0.00 | 0–38 | 0.00–4.30 |  |  |
|  | May | 701 | 0–0 | 0.00–0.00 | 119–183 | 13.48–20.62 |  | ** |
|  | June | 852 | 0–0 | 0.00–0.00 | 0–36 | 0.00–4.03 |  |  |
|  | (2) Average number of hospitalized patients per day |  |  |  |  |  |  |  |
|  | (b) Psychiatric care beds |  |  |  |  |  |  |  |
|  | January | 2884 | 0–0 | 0.00–0.00 | 0–36 | 0.00–1.22 |  |  |
|  | February | 2900 | 0–0 | 0.00–0.00 | 0–25 | 0.00–0.83 |  |  |
|  | March | 2862 | 0–0 | 0.00–0.00 | 0–68 | 0.00–2.31 |  |  |
|  | April | 2854 | 0–0 | 0.00–0.00 | 0–75 | 0.00–2.53 |  |  |
|  | May | 2830 | 0–0 | 0.00–0.00 | 0–98 | 0.00–3.33 |  |  |
|  | June | 2800 | 0–0 | 0.00–0.00 | 28–134 | 0.95–4.54 |  | ** |
|  | (c) Tuberculosis care beds |  |  |  |  |  |  |  |
|  | January | 7 | 0–0 | 0.00–0.00 | 0–3 | 0.00–26.58 |  |  |
|  | February | 7 | 0–0 | 0.00–0.00 | 0–3 | 0.00–28.19 |  |  |
|  | March | 8 | 0–0 | 0.00–0.00 | 0–3 | 0.00–23.53 |  |  |
|  | April | 9 | 0–0 | 0.00–0.00 | 0–3 | 0.00–21.33 |  |  |
|  | May | 11 | 0–0 | 0.00–0.00 | 0–1 | 0.00–4.51 |  |  |
|  | June | 14 | 0–3 | 0.00–18.18 | 0–0 | 0.00–0.00 |  |  |
|  | (d) Long-term care beds |  |  |  |  |  |  |  |
|  | January | 1839 | 0–0 | 0.00–0.00 | 0–9 | 0.00–0.46 |  |  |
|  | February | 1856 | 0–3 | 0.00–0.12 | 0–0 | 0.00–0.00 |  |  |
|  | March | 1848 | 0–0 | 0.00–0.00 | 0–6 | 0.00–0.30 |  |  |
|  | April | 1644 | 0–0 | 0.00–0.00 | 105–189 | 5.73–10.27 |  | ** |
|  | May | 1634 | 0–0 | 0.00–0.00 | 96–179 | 5.30–9.86 |  | ** |
|  | June | 1630 | 0–0 | 0.00–0.00 | 88–171 | 4.89–9.47 |  | ** |
|  | (e) General beds |  |  |  |  |  |  |  |
|  | January | 6560 | 0–0 | 0.00–0.00 | 0–95 | 0.00–1.42 |  |  |
|  | February | 6641 | 0–0 | 0.00–0.00 | 0–79 | 0.00–1.16 |  |  |
|  | March | 6457 | 0–0 | 0.00–0.00 | 99–271 | 1.47–4.02 |  | ** |
|  | April | 6010 | 0–0 | 0.00–0.00 | 328–559 | 4.99–8.50 |  | ** |
|  | May | 5584 | 0–0 | 0.00–0.00 | 739–905 | 11.39–13.94 |  | ** |
|  | June | 5927 | 0–0 | 0.00–0.00 | 383–540 | 5.92–8.34 |  | ** |
|  | (f) LTCI care beds |  |  |  |  |  |  |  |
|  | January | 315 | 0–0 | 0.00–0.00 | 0–51 | 0.00–13.77 |  |  |
|  | February | 319 | 0–0 | 0.00–0.00 | 0–42 | 0.00–11.61 |  |  |
|  | March | 320 | 0–0 | 0.00–0.00 | 0–36 | 0.00–9.91 |  |  |
|  | April | 240 | 0–0 | 0.00–0.00 | 21–103 | 6.12–30.00 |  | ** |
|  | May | 237 | 0–0 | 0.00–0.00 | 15–94 | 4.54–28.25 |  | ** |
|  | June | 224 | 0–0 | 0.00–0.00 | 18–94 | 5.67–29.43 |  | ** |
|  | (3) Average length of hospital stays per patient |  |  |  |  |  |  |  |
|  | (b) Psychiatric care beds |  |  |  |  |  |  |  |
|  | January | 292 | 0–3 | 0.00–0.92 | 0–0 | 0.00–0.00 |  |  |
|  | February | 291 | 0–2 | 0.00–0.43 | 0–0 | 0.00–0.00 |  |  |
|  | March | 304 | 0–11 | 0.00–3.50 | 0–0 | 0.00–0.00 |  |  |
|  | April | 371 | 35–71 | 11.54–23.32 | 0–0 | 0.00–0.00 | * |  |
|  | May | 352 | 19–54 | 6.15–17.80 | 0–0 | 0.00–0.00 | * |  |
|  | June | 212 | 0–0 | 0.00–0.00 | 58–90 | 18.93–29.74 |  | ** |
|  | (c) Tuberculosis care beds |  |  |  |  |  |  |  |
|  | January | 74 | 0–12 | 0.00–17.94 | 0–0 | 0.00–0.00 |  |  |
|  | February | 71 | 0–0 | 0.00–0.00 | 0–11 | 0.00–12.53 |  |  |
|  | March | 239 | 88–151 | 99.03–168.95 | 0–0 | 0.00–0.00 | * |  |
|  | April | 185 | 44–101 | 51.22–119.21 | 0–0 | 0.00–0.00 | * |  |
|  | May | 52 | 0–0 | 0.00–0.00 | 0–37 | 0.00–41.20 |  |  |
|  | June | 141 | 0–51 | 0.00–56.12 | 0–0 | 0.00–0.00 |  |  |
|  | (d) Long-term care beds |  |  |  |  |  |  |  |
|  | January | 150 | 0–0 | 0.00–0.00 | 0–7 | 0.00–4.23 |  |  |
|  | February | 172 | 0–16 | 0.00–10.08 | 0–0 | 0.00–0.00 |  |  |
|  | March | 164 | 0–11 | 0.00–6.97 | 0–0 | 0.00–0.00 |  |  |
|  | April | 143 | 0–0 | 0.00–0.00 | 0–13 | 0.00–8.17 |  |  |
|  | May | 157 | 0–0 | 0.00–0.00 | 0–3 | 0.00–1.55 |  |  |
|  | June | 148 | 0–0 | 0.00–0.00 | 0–16 | 0.00–9.66 |  |  |
|  | (e) General beds |  |  |  |  |  |  |  |
|  | January | 17 | 0–1 | 0.00–2.33 | 0–0 | 0.00–0.00 |  |  |
|  | February | 17 | 0–1 | 0.00–0.62 | 0–0 | 0.00–0.00 |  |  |
|  | March | 17 | 0–0 | 0.00–0.00 | 0–1 | 0.00–0.68 |  |  |
|  | April | 17 | 0–1 | 0.00–1.95 | 0–0 | 0.00–0.00 |  |  |
|  | May | 19 | 0–3 | 0.00–14.78 | 0–0 | 0.00–0.00 |  |  |
|  | June | 17 | 0–1 | 0.00–1.64 | 0–0 | 0.00–0.00 |  |  |
|  | (f) LTCI care beds |  |  |  |  |  |  |  |
|  | January | 261 | 0–28 | 0.00–11.68 | 0–0 | 0.00–0.00 |  |  |
|  | February | 180 | 0–0 | 0.00–0.00 | 0–68 | 0.00–27.21 |  |  |
|  | March | 258 | 0–21 | 0.00–8.50 | 0–0 | 0.00–0.00 |  |  |
|  | April | 306 | 0–62 | 0.00–25.09 | 0–0 | 0.00–0.00 |  |  |
|  | May | 312 | 0–65 | 0.00–25.80 | 0–0 | 0.00–0.00 |  |  |
|  | June | 305 | 0–44 | 0.00–16.79 | 0–0 | 0.00–0.00 |  |  |
| Ehime | (1) Average number of outpatients per day at hospitals |  |  |  |  |  |  |  |
|  | General hospitals |  |  |  |  |  |  |  |
|  | January | 16493 | 0–0 | 0.00–0.00 | 0–467 | 0.00–2.75 |  |  |
|  | February | 16487 | 0–0 | 0.00–0.00 | 0–662 | 0.00–3.86 |  |  |
|  | March | 16224 | 0–0 | 0.00–0.00 | 54–1173 | 0.31–6.74 |  | ** |
|  | April | 15219 | 0–0 | 0.00–0.00 | 724–1788 | 4.26–10.51 |  | ** |
|  | May | 13212 | 0–0 | 0.00–0.00 | 2770–3903 | 16.19–22.80 |  | ** |
|  | June | 16083 | 0–0 | 0.00–0.00 | 29–1162 | 0.17–6.73 |  | ** |
|  | Psychiatric hospitals |  |  |  |  |  |  |  |
|  | January | 741 | 0–0 | 0.00–0.00 | 0–44 | 0.00–5.54 |  |  |
|  | February | 774 | 0–0 | 0.00–0.00 | 0–20 | 0.00–2.41 |  |  |
|  | March | 746 | 0–0 | 0.00–0.00 | 0–63 | 0.00–7.78 |  |  |
|  | April | 738 | 0–0 | 0.00–0.00 | 0–57 | 0.00–7.07 |  |  |
|  | May | 629 | 0–0 | 0.00–0.00 | 98–171 | 12.26–21.31 |  | ** |
|  | June | 752 | 0–0 | 0.00–0.00 | 0–50 | 0.00–6.18 |  |  |
|  | (2) Average number of hospitalized patients per day |  |  |  |  |  |  |  |
|  | (b) Psychiatric care beds |  |  |  |  |  |  |  |
|  | January | 3515 | 0–0 | 0.00–0.00 | 0–52 | 0.00–1.44 |  |  |
|  | February | 3550 | 0–0 | 0.00–0.00 | 0–13 | 0.00–0.34 |  |  |
|  | March | 3580 | 0–19 | 0.00–0.53 | 0–0 | 0.00–0.00 |  |  |
|  | April | 3548 | 0–0 | 0.00–0.00 | 0–11 | 0.00–0.28 |  |  |
|  | May | 3503 | 0–0 | 0.00–0.00 | 0–57 | 0.00–1.59 |  |  |
|  | June | 3511 | 0–0 | 0.00–0.00 | 0–51 | 0.00–1.43 |  |  |
|  | (c) Tuberculosis care beds |  |  |  |  |  |  |  |
|  | January | 8 | 0–0 | 0.00–0.00 | 0–2 | 0.00–11.35 |  |  |
|  | February | 12 | 0–3 | 0.00–26.08 | 0–0 | 0.00–0.00 |  |  |
|  | March | 12 | 0–2 | 0.00–18.65 | 0–0 | 0.00–0.00 |  |  |
|  | April | 14 | 0–4 | 0.00–37.35 | 0–0 | 0.00–0.00 |  |  |
|  | May | 9 | 0–0 | 0.00–0.00 | 0–2 | 0.00–11.33 |  |  |
|  | June | 10 | 0–1 | 0.00–1.43 | 0–0 | 0.00–0.00 |  |  |
|  | (d) Long-term care beds |  |  |  |  |  |  |  |
|  | January | 3854 | 0–0 | 0.00–0.00 | 134–259 | 3.26–6.28 |  | ** |
|  | February | 3969 | 0–0 | 0.00–0.00 | 20–145 | 0.49–3.52 |  | ** |
|  | March | 3950 | 0–0 | 0.00–0.00 | 22–147 | 0.54–3.58 |  | ** |
|  | April | 3843 | 0–0 | 0.00–0.00 | 89–213 | 2.19–5.25 |  | ** |
|  | May | 3828 | 0–0 | 0.00–0.00 | 63–186 | 1.57–4.63 |  | ** |
|  | June | 3774 | 0–0 | 0.00–0.00 | 95–218 | 2.38–5.46 |  | ** |
|  | (e) General beds |  |  |  |  |  |  |  |
|  | January | 8721 | 0–0 | 0.00–0.00 | 0–192 | 0.00–2.14 |  |  |
|  | February | 8889 | 0–0 | 0.00–0.00 | 0–104 | 0.00–1.15 |  |  |
|  | March | 8521 | 0–0 | 0.00–0.00 | 203–470 | 2.26–5.22 |  | ** |
|  | April | 8103 | 0–0 | 0.00–0.00 | 409–698 | 4.65–7.93 |  | ** |
|  | May | 7701 | 0–0 | 0.00–0.00 | 787–1023 | 9.02–11.72 |  | ** |
|  | June | 7899 | 0–0 | 0.00–0.00 | 593–833 | 6.79–9.53 |  | ** |
|  | (f) LTCI care beds |  |  |  |  |  |  |  |
|  | January | 332 | 0–0 | 0.00–0.00 | 14–56 | 3.62–14.21 |  | ** |
|  | February | 333 | 0–0 | 0.00–0.00 | 0–36 | 0.00–9.73 |  |  |
|  | March | 336 | 0–0 | 0.00–0.00 | 0–26 | 0.00–7.13 |  |  |
|  | April | 296 | 0–0 | 0.00–0.00 | 23–61 | 6.44–17.06 |  | ** |
|  | May | 296 | 0–0 | 0.00–0.00 | 19–55 | 5.42–15.52 |  | ** |
|  | June | 241 | 0–0 | 0.00–0.00 | 72–109 | 20.62–30.97 |  | ** |
|  | (3) Average length of hospital stays per patient |  |  |  |  |  |  |  |
|  | (b) Psychiatric care beds |  |  |  |  |  |  |  |
|  | January | 326 | 0–32 | 0.00–10.67 | 0–0 | 0.00–0.00 |  |  |
|  | February | 297 | 0–7 | 0.00–2.42 | 0–0 | 0.00–0.00 |  |  |
|  | March | 333 | 14–48 | 4.84–16.74 | 0–0 | 0.00–0.00 | * |  |
|  | April | 318 | 4–38 | 1.14–13.41 | 0–0 | 0.00–0.00 | * |  |
|  | May | 349 | 37–71 | 12.97–25.15 | 0–0 | 0.00–0.00 | * |  |
|  | June | 318 | 10–43 | 3.53–15.63 | 0–0 | 0.00–0.00 | * |  |
|  | (c) Tuberculosis care beds |  |  |  |  |  |  |  |
|  | January | 86 | 0–12 | 0.00–14.72 | 0–0 | 0.00–0.00 |  |  |
|  | February | 86 | 0–9 | 0.00–11.21 | 0–0 | 0.00–0.00 |  |  |
|  | March | 35 | 0–0 | 0.00–0.00 | 0–43 | 0.00–54.98 |  |  |
|  | April | 34 | 0–0 | 0.00–0.00 | 2–50 | 2.41–59.06 |  | ** |
|  | May | 60 | 0–0 | 0.00–0.00 | 0–29 | 0.00–31.86 |  |  |
|  | June | 39 | 0–0 | 0.00–0.00 | 0–42 | 0.00–51.19 |  |  |
|  | (d) Long-term care beds |  |  |  |  |  |  |  |
|  | January | 112 | 0–5 | 0.00–3.87 | 0–0 | 0.00–0.00 |  |  |
|  | February | 110 | 0–3 | 0.00–2.75 | 0–0 | 0.00–0.00 |  |  |
|  | March | 112 | 0–9 | 0.00–8.31 | 0–0 | 0.00–0.00 |  |  |
|  | April | 107 | 0–3 | 0.00–2.43 | 0–0 | 0.00–0.00 |  |  |
|  | May | 122 | 0–17 | 0.00–15.12 | 0–0 | 0.00–0.00 |  |  |
|  | June | 107 | 0–0 | 0.00–0.00 | 0–1 | 0.00–0.00 |  |  |
|  | (e) General beds |  |  |  |  |  |  |  |
|  | January | 18 | 0–1 | 0.00–3.07 | 0–0 | 0.00–0.00 |  |  |
|  | February | 18 | 0–1 | 0.00–0.06 | 0–0 | 0.00–0.00 |  |  |
|  | March | 17 | 0–1 | 0.00–1.03 | 0–0 | 0.00–0.00 |  |  |
|  | April | 18 | 0–1 | 0.00–2.48 | 0–0 | 0.00–0.00 |  |  |
|  | May | 20 | 0–3 | 0.00–13.95 | 0–0 | 0.00–0.00 |  |  |
|  | June | 17 | 0–1 | 0.00–0.28 | 0–0 | 0.00–0.00 |  |  |
|  | (f) LTCI care beds |  |  |  |  |  |  |  |
|  | January | 174 | 0–0 | 0.00–0.00 | 17–65 | 6.95–26.94 |  | ** |
|  | February | 188 | 0–0 | 0.00–0.00 | 0–42 | 0.00–18.28 |  |  |
|  | March | 191 | 0–0 | 0.00–0.00 | 0–22 | 0.00–10.03 |  |  |
|  | April | 152 | 0–0 | 0.00–0.00 | 23–68 | 10.17–30.78 |  | ** |
|  | May | 163 | 0–0 | 0.00–0.00 | 13–56 | 5.69–25.45 |  | ** |
|  | June | 148 | 0–0 | 0.00–0.00 | 41–84 | 17.48–36.12 |  | ** |
| Kochi | (1) Average number of outpatients per day at hospitals |  |  |  |  |  |  |  |
|  | General hospitals |  |  |  |  |  |  |  |
|  | January | 11062 | 0–0 | 0.00–0.00 | 0–497 | 0.00–4.29 |  |  |
|  | February | 11081 | 0–0 | 0.00–0.00 | 0–557 | 0.00–4.78 |  |  |
|  | March | 10625 | 0–0 | 0.00–0.00 | 321–1046 | 2.75–8.96 |  | ** |
|  | April | 9969 | 0–0 | 0.00–0.00 | 736–1433 | 6.46–12.56 |  | ** |
|  | May | 9109 | 0–0 | 0.00–0.00 | 1635–2330 | 14.29–20.37 |  | ** |
|  | June | 10985 | 0–0 | 0.00–0.00 | 0–530 | 0.00–4.60 |  |  |
|  | Psychiatric hospitals |  |  |  |  |  |  |  |
|  | January | 461 | 0–0 | 0.00–0.00 | 0–18 | 0.00–3.75 |  |  |
|  | February | 475 | 0–0 | 0.00–0.00 | 0–11 | 0.00–2.21 |  |  |
|  | March | 459 | 0–0 | 0.00–0.00 | 0–42 | 0.00–8.24 |  |  |
|  | April | 470 | 0–0 | 0.00–0.00 | 0–20 | 0.00–3.98 |  |  |
|  | May | 405 | 0–0 | 0.00–0.00 | 46–88 | 9.33–17.84 |  | ** |
|  | June | 474 | 0–0 | 0.00–0.00 | 0–18 | 0.00–3.60 |  |  |
|  | (2) Average number of hospitalized patients per day |  |  |  |  |  |  |  |
|  | (b) Psychiatric care beds |  |  |  |  |  |  |  |
|  | January | 2957 | 0–0 | 0.00–0.00 | 0–8 | 0.00–0.27 |  |  |
|  | February | 2980 | 0–4 | 0.00–0.12 | 0–0 | 0.00–0.00 |  |  |
|  | March | 2963 | 0–0 | 0.00–0.00 | 0–19 | 0.00–0.60 |  |  |
|  | April | 2947 | 0–0 | 0.00–0.00 | 0–34 | 0.00–1.13 |  |  |
|  | May | 2949 | 0–0 | 0.00–0.00 | 0–32 | 0.00–1.04 |  |  |
|  | June | 2979 | 0–0 | 0.00–0.00 | 0–11 | 0.00–0.35 |  |  |
|  | (c) Tuberculosis care beds |  |  |  |  |  |  |  |
|  | January | 6 | 0–0 | 0.00–0.00 | 0–1 | 0.00–6.24 |  |  |
|  | February | 7 | 0–1 | 0.00–3.35 | 0–0 | 0.00–0.00 |  |  |
|  | March | 12 | 0–5 | 0.00–66.82 | 0–0 | 0.00–0.00 |  |  |
|  | April | 14 | 0–7 | 0.00–91.99 | 0–0 | 0.00–0.00 |  |  |
|  | May | 11 | 0–3 | 0.00–36.70 | 0–0 | 0.00–0.00 |  |  |
|  | June | 10 | 0–2 | 0.00–21.91 | 0–0 | 0.00–0.00 |  |  |
|  | (d) Long-term care beds |  |  |  |  |  |  |  |
|  | January | 5305 | 0–0 | 0.00–0.00 | 189–336 | 3.35–5.95 |  | ** |
|  | February | 5298 | 0–0 | 0.00–0.00 | 155–302 | 2.77–5.38 |  | ** |
|  | March | 5187 | 0–0 | 0.00–0.00 | 226–372 | 4.07–6.68 |  | ** |
|  | April | 4301 | 0–0 | 0.00–0.00 | 1056–1201 | 19.20–21.82 |  | ** |
|  | May | 4262 | 0–0 | 0.00–0.00 | 1045–1189 | 19.17–21.81 |  | ** |
|  | June | 4196 | 0–0 | 0.00–0.00 | 1083–1227 | 19.97–22.61 |  | ** |
|  | (e) General beds |  |  |  |  |  |  |  |
|  | January | 6162 | 0–0 | 0.00–0.00 | 0–174 | 0.00–2.74 |  |  |
|  | February | 6278 | 0–0 | 0.00–0.00 | 0–139 | 0.00–2.16 |  |  |
|  | March | 5992 | 0–0 | 0.00–0.00 | 204–403 | 3.19–6.29 |  | ** |
|  | April | 5772 | 0–0 | 0.00–0.00 | 257–492 | 4.10–7.84 |  | ** |
|  | May | 5562 | 0–0 | 0.00–0.00 | 444–612 | 7.19–9.90 |  | ** |
|  | June | 5739 | 0–0 | 0.00–0.00 | 240–420 | 3.90–6.81 |  | ** |
|  | (f) LTCI care beds |  |  |  |  |  |  |  |
|  | January | 1313 | 0–0 | 0.00–0.00 | 226–305 | 13.97–18.81 |  | ** |
|  | February | 1298 | 0–0 | 0.00–0.00 | 225–303 | 14.06–18.91 |  | ** |
|  | March | 1246 | 0–0 | 0.00–0.00 | 234–320 | 14.95–20.42 |  | ** |
|  | April | 382 | 0–0 | 0.00–0.00 | 1058–1157 | 68.77–75.17 |  | ** |
|  | May | 379 | 0–0 | 0.00–0.00 | 1011–1118 | 67.57–74.67 |  | ** |
|  | June | 332 | 0–0 | 0.00–0.00 | 1042–1152 | 70.26–77.61 |  | ** |
|  | (3) Average length of hospital stays per patient |  |  |  |  |  |  |  |
|  | (b) Psychiatric care beds |  |  |  |  |  |  |  |
|  | January | 265 | 0–22 | 0.00–8.68 | 0–0 | 0.00–0.00 |  |  |
|  | February | 240 | 0–6 | 0.00–2.47 | 0–0 | 0.00–0.00 |  |  |
|  | March | 250 | 0–16 | 0.00–6.70 | 0–0 | 0.00–0.00 |  |  |
|  | April | 266 | 0–31 | 0.00–12.75 | 0–0 | 0.00–0.00 |  |  |
|  | May | 289 | 26–57 | 10.86–24.19 | 0–0 | 0.00–0.00 | * |  |
|  | June | 231 | 0–1 | 0.00–0.38 | 0–0 | 0.00–0.00 |  |  |
|  | (c) Tuberculosis care beds |  |  |  |  |  |  |  |
|  | January | 26 | 0–0 | 0.00–0.00 | 0–6 | 0.00–16.48 |  |  |
|  | February | 60 | 0–31 | 0.00–103.53 | 0–0 | 0.00–0.00 |  |  |
|  | March | 27 | 0–0 | 0.00–0.00 | 0–15 | 0.00–34.62 |  |  |
|  | April | 35 | 0–0 | 0.00–0.00 | 0–2 | 0.00–4.07 |  |  |
|  | May | 30 | 0–0 | 0.00–0.00 | 0–9 | 0.00–22.38 |  |  |
|  | June | 51 | 0–18 | 0.00–52.97 | 0–0 | 0.00–0.00 |  |  |
|  | (d) Long-term care beds |  |  |  |  |  |  |  |
|  | January | 187 | 0–10 | 0.00–5.65 | 0–0 | 0.00–0.00 |  |  |
|  | February | 175 | 0–0 | 0.00–0.00 | 0–1 | 0.00–0.12 |  |  |
|  | March | 149 | 0–0 | 0.00–0.00 | 0–21 | 0.00–12.22 |  |  |
|  | April | 130 | 0–0 | 0.00–0.00 | 20–45 | 11.34–25.45 |  | ** |
|  | May | 161 | 0–0 | 0.00–0.00 | 0–19 | 0.00–10.43 |  |  |
|  | June | 149 | 0–0 | 0.00–0.00 | 7–32 | 3.50–17.35 |  | ** |
|  | (e) General beds |  |  |  |  |  |  |  |
|  | January | 22 | 0–0 | 0.00–0.00 | 0–1 | 0.00–1.10 |  |  |
|  | February | 22 | 0–0 | 0.00–0.00 | 0–1 | 0.00–1.69 |  |  |
|  | March | 22 | 0–1 | 0.00–2.23 | 0–0 | 0.00–0.00 |  |  |
|  | April | 22 | 0–1 | 0.00–2.34 | 0–0 | 0.00–0.00 |  |  |
|  | May | 24 | 0–3 | 0.00–13.35 | 0–0 | 0.00–0.00 |  |  |
|  | June | 21 | 0–0 | 0.00–0.00 | 0–1 | 0.00–0.73 |  |  |
|  | (f) LTCI care beds |  |  |  |  |  |  |  |
|  | January | 577 | 114–206 | 30.49–55.27 | 0–0 | 0.00–0.00 | * |  |
|  | February | 476 | 8–98 | 1.95–25.60 | 0–0 | 0.00–0.00 | * |  |
|  | March | 123 | 0–0 | 0.00–0.00 | 176–249 | 47.36–66.81 |  | ** |
|  | April | 84 | 0–0 | 0.00–0.00 | 233–311 | 59.02–78.72 |  | ** |
|  | May | 290 | 0–0 | 0.00–0.00 | 34–114 | 8.31–28.16 |  | ** |
|  | June | 486 | 0–91 | 0.00–22.76 | 0–0 | 0.00–0.00 |  |  |
| Fukuoka | (1) Average number of outpatients per day at hospitals |  |  |  |  |  |  |  |
|  | General hospitals |  |  |  |  |  |  |  |
|  | January | 49765 | 0–0 | 0.00–0.00 | 0–1951 | 0.00–3.77 |  |  |
|  | February | 49930 | 0–0 | 0.00–0.00 | 0–2431 | 0.00–4.64 |  |  |
|  | March | 48996 | 0–0 | 0.00–0.00 | 379–3510 | 0.72–6.68 |  | ** |
|  | April | 40363 | 0–0 | 0.00–0.00 | 7844–10906 | 15.30–21.27 |  | ** |
|  | May | 36410 | 0–0 | 0.00–0.00 | 11986–15058 | 23.29–29.26 |  | ** |
|  | June | 46323 | 0–0 | 0.00–0.00 | 2344–5506 | 4.52–10.62 |  | ** |
|  | Psychiatric hospitals |  |  |  |  |  |  |  |
|  | January | 3137 | 0–0 | 0.00–0.00 | 0–222 | 0.00–6.60 |  |  |
|  | February | 3281 | 0–0 | 0.00–0.00 | 0–142 | 0.00–4.12 |  |  |
|  | March | 3297 | 0–0 | 0.00–0.00 | 0–201 | 0.00–5.73 |  |  |
|  | April | 3090 | 0–0 | 0.00–0.00 | 124–357 | 3.60–10.35 |  | ** |
|  | May | 2708 | 0–0 | 0.00–0.00 | 529–767 | 15.23–22.06 |  | ** |
|  | June | 3266 | 0–0 | 0.00–0.00 | 0–216 | 0.00–6.20 |  |  |
|  | (2) Average number of hospitalized patients per day |  |  |  |  |  |  |  |
|  | (b) Psychiatric care beds |  |  |  |  |  |  |  |
|  | January | 18360 | 0–0 | 0.00–0.00 | 0–36 | 0.00–0.19 |  |  |
|  | February | 18362 | 0–0 | 0.00–0.00 | 0–66 | 0.00–0.36 |  |  |
|  | March | 18391 | 0–0 | 0.00–0.00 | 0–57 | 0.00–0.31 |  |  |
|  | April | 18197 | 0–0 | 0.00–0.00 | 0–232 | 0.00–1.26 |  |  |
|  | May | 18138 | 0–0 | 0.00–0.00 | 34–300 | 0.18–1.63 |  | ** |
|  | June | 18272 | 0–0 | 0.00–0.00 | 0–202 | 0.00–1.09 |  |  |
|  | (c) Tuberculosis care beds |  |  |  |  |  |  |  |
|  | January | 77 | 0–0 | 0.00–0.00 | 0–14 | 0.00–14.52 |  |  |
|  | February | 72 | 0–0 | 0.00–0.00 | 1–19 | 1.11–20.21 |  | ** |
|  | March | 77 | 0–0 | 0.00–0.00 | 0–13 | 0.00–14.30 |  |  |
|  | April | 88 | 0–0 | 0.00–0.00 | 0–1 | 0.00–0.16 |  |  |
|  | May | 77 | 0–0 | 0.00–0.00 | 0–11 | 0.00–12.11 |  |  |
|  | June | 65 | 0–0 | 0.00–0.00 | 6–24 | 6.79–26.39 |  | ** |
|  | (d) Long-term care beds |  |  |  |  |  |  |  |
|  | January | 16184 | 0–0 | 0.00–0.00 | 699–1251 | 4.01–7.17 |  | ** |
|  | February | 16158 | 0–0 | 0.00–0.00 | 519–1161 | 3.00–6.70 |  | ** |
|  | March | 15979 | 0–0 | 0.00–0.00 | 578–1237 | 3.36–7.18 |  | ** |
|  | April | 15715 | 0–0 | 0.00–0.00 | 578–1275 | 3.40–7.50 |  | ** |
|  | May | 15485 | 0–0 | 0.00–0.00 | 592–1299 | 3.53–7.74 |  | ** |
|  | June | 15441 | 0–0 | 0.00–0.00 | 436–1173 | 2.62–7.06 |  | ** |
|  | (e) General beds |  |  |  |  |  |  |  |
|  | January | 34732 | 0–0 | 0.00–0.00 | 0–765 | 0.00–2.15 |  |  |
|  | February | 35708 | 0–0 | 0.00–0.00 | 0–255 | 0.00–0.71 |  |  |
|  | March | 34534 | 0–0 | 0.00–0.00 | 511–1420 | 1.42–3.95 |  | ** |
|  | April | 31365 | 0–0 | 0.00–0.00 | 2829–3914 | 8.02–11.09 |  | ** |
|  | May | 29369 | 0–0 | 0.00–0.00 | 4825–5628 | 13.79–16.08 |  | ** |
|  | June | 30883 | 0–0 | 0.00–0.00 | 3284–4128 | 9.38–11.79 |  | ** |
|  | (f) LTCI care beds |  |  |  |  |  |  |  |
|  | January | 1092 | 0–0 | 0.00–0.00 | 962–1163 | 42.66–51.57 |  | ** |
|  | February | 932 | 0–0 | 0.00–0.00 | 1062–1274 | 48.14–57.75 |  | ** |
|  | March | 874 | 0–0 | 0.00–0.00 | 822–1154 | 40.54–56.89 |  | ** |
|  | April | 821 | 0–0 | 0.00–0.00 | 771–1124 | 39.65–57.78 |  | ** |
|  | May | 817 | 0–0 | 0.00–0.00 | 727–1086 | 38.22–57.05 |  | ** |
|  | June | 829 | 0–0 | 0.00–0.00 | 616–1009 | 33.52–54.89 |  | ** |
|  | (3) Average length of hospital stays per patient |  |  |  |  |  |  |  |
|  | (b) Psychiatric care beds |  |  |  |  |  |  |  |
|  | January | 296 | 0–10 | 0.00–3.29 | 0–0 | 0.00–0.00 |  |  |
|  | February | 298 | 0–15 | 0.00–5.20 | 0–0 | 0.00–0.00 |  |  |
|  | March | 297 | 0–23 | 0.00–8.16 | 0–0 | 0.00–0.00 |  |  |
|  | April | 333 | 24–58 | 8.48–20.77 | 0–0 | 0.00–0.00 | * |  |
|  | May | 357 | 50–84 | 18.16–30.33 | 0–0 | 0.00–0.00 | * |  |
|  | June | 296 | 0–24 | 0.00–8.49 | 0–0 | 0.00–0.00 |  |  |
|  | (c) Tuberculosis care beds |  |  |  |  |  |  |  |
|  | January | 53 | 0–6 | 0.00–10.73 | 0–0 | 0.00–0.00 |  |  |
|  | February | 51 | 0–4 | 0.00–8.38 | 0–0 | 0.00–0.00 |  |  |
|  | March | 67 | 2–19 | 3.35–39.59 | 0–0 | 0.00–0.00 | * |  |
|  | April | 36 | 0–0 | 0.00–0.00 | 1–18 | 1.31–31.96 |  | ** |
|  | May | 50 | 0–0 | 0.00–0.00 | 0–4 | 0.00–5.73 |  |  |
|  | June | 42 | 0–0 | 0.00–0.00 | 0–13 | 0.00–22.70 |  |  |
|  | (d) Long-term care beds |  |  |  |  |  |  |  |
|  | January | 132 | 0–4 | 0.00–2.60 | 0–0 | 0.00–0.00 |  |  |
|  | February | 128 | 0–0 | 0.00–0.00 | 0–1 | 0.00–0.21 |  |  |
|  | March | 129 | 0–5 | 0.00–3.70 | 0–0 | 0.00–0.00 |  |  |
|  | April | 135 | 0–9 | 0.00–6.43 | 0–0 | 0.00–0.00 |  |  |
|  | May | 153 | 3–26 | 1.88–20.15 | 0–0 | 0.00–0.00 | * |  |
|  | June | 135 | 0–6 | 0.00–4.47 | 0–0 | 0.00–0.00 |  |  |
|  | (e) General beds |  |  |  |  |  |  |  |
|  | January | 19 | 0–1 | 0.00–1.98 | 0–0 | 0.00–0.00 |  |  |
|  | February | 18 | 0–1 | 0.00–0.62 | 0–0 | 0.00–0.00 |  |  |
|  | March | 18 | 0–1 | 0.00–2.39 | 0–0 | 0.00–0.00 |  |  |
|  | April | 20 | 0–3 | 0.00–12.23 | 0–0 | 0.00–0.00 |  |  |
|  | May | 22 | 0–4 | 0.00–21.65 | 0–0 | 0.00–0.00 |  |  |
|  | June | 18 | 0–1 | 0.00–3.43 | 0–0 | 0.00–0.00 |  |  |
|  | (f) LTCI care beds |  |  |  |  |  |  |  |
|  | January | 241 | 0–0 | 0.00–0.00 | 67–134 | 17.66–35.65 |  | ** |
|  | February | 249 | 0–0 | 0.00–0.00 | 59–125 | 15.77–33.30 |  | ** |
|  | March | 390 | 0–62 | 0.00–18.71 | 0–0 | 0.00–0.00 |  |  |
|  | April | 337 | 0–14 | 0.00–4.32 | 0–0 | 0.00–0.00 |  |  |
|  | May | 402 | 0–61 | 0.00–17.56 | 0–0 | 0.00–0.00 |  |  |
|  | June | 404 | 0–56 | 0.00–16.00 | 0–0 | 0.00–0.00 |  |  |
| Saga | (1) Average number of outpatients per day at hospitals |  |  |  |  |  |  |  |
|  | General hospitals |  |  |  |  |  |  |  |
|  | January | 8965 | 0–0 | 0.00–0.00 | 0–261 | 0.00–2.82 |  |  |
|  | February | 8943 | 0–0 | 0.00–0.00 | 0–360 | 0.00–3.86 |  |  |
|  | March | 8905 | 0–0 | 0.00–0.00 | 0–378 | 0.00–4.07 |  |  |
|  | April | 8037 | 0–0 | 0.00–0.00 | 498–1032 | 5.49–11.37 |  | ** |
|  | May | 6992 | 0–0 | 0.00–0.00 | 1565–2096 | 17.22–23.06 |  | ** |
|  | June | 8739 | 0–0 | 0.00–0.00 | 0–418 | 0.00–4.56 |  |  |
|  | Psychiatric hospitals |  |  |  |  |  |  |  |
|  | January | 709 | 0–0 | 0.00–0.00 | 0–26 | 0.00–3.45 |  |  |
|  | February | 757 | 0–14 | 0.00–1.80 | 0–0 | 0.00–0.00 |  |  |
|  | March | 746 | 0–0 | 0.00–0.00 | 0–17 | 0.00–2.17 |  |  |
|  | April | 733 | 0–0 | 0.00–0.00 | 0–27 | 0.00–3.49 |  |  |
|  | May | 641 | 0–0 | 0.00–0.00 | 68–122 | 8.92–15.91 |  | ** |
|  | June | 746 | 0–0 | 0.00–0.00 | 0–17 | 0.00–2.18 |  |  |
|  | (2) Average number of hospitalized patients per day |  |  |  |  |  |  |  |
|  | (b) Psychiatric care beds |  |  |  |  |  |  |  |
|  | January | 3669 | 0–0 | 0.00–0.00 | 0–55 | 0.00–1.46 |  |  |
|  | February | 3653 | 0–0 | 0.00–0.00 | 0–81 | 0.00–2.14 |  |  |
|  | March | 3652 | 0–0 | 0.00–0.00 | 0–85 | 0.00–2.27 |  |  |
|  | April | 3636 | 0–0 | 0.00–0.00 | 0–103 | 0.00–2.75 |  |  |
|  | May | 3606 | 0–0 | 0.00–0.00 | 18–138 | 0.48–3.66 |  | ** |
|  | June | 3641 | 0–0 | 0.00–0.00 | 0–105 | 0.00–2.80 |  |  |
|  | (c) Tuberculosis care beds |  |  |  |  |  |  |  |
|  | January | 16 | 0–6 | 0.00–46.61 | 0–0 | 0.00–0.00 |  |  |
|  | February | 15 | 0–5 | 0.00–46.49 | 0–0 | 0.00–0.00 |  |  |
|  | March | 22 | 4–12 | 38.69–112.82 | 0–0 | 0.00–0.00 | * |  |
|  | April | 21 | 2–10 | 17.53–84.10 | 0–0 | 0.00–0.00 | * |  |
|  | May | 21 | 1–9 | 8.08–69.60 | 0–0 | 0.00–0.00 | * |  |
|  | June | 16 | 0–3 | 0.00–18.86 | 0–0 | 0.00–0.00 |  |  |
|  | (d) Long-term care beds |  |  |  |  |  |  |  |
|  | January | 3585 | 0–0 | 0.00–0.00 | 48–167 | 1.28–4.45 |  | ** |
|  | February | 3592 | 0–0 | 0.00–0.00 | 42–162 | 1.12–4.30 |  | ** |
|  | March | 3579 | 0–0 | 0.00–0.00 | 38–158 | 1.02–4.21 |  | ** |
|  | April | 3539 | 0–0 | 0.00–0.00 | 43–162 | 1.16–4.35 |  | ** |
|  | May | 3452 | 0–0 | 0.00–0.00 | 93–211 | 2.54–5.74 |  | ** |
|  | June | 3453 | 0–0 | 0.00–0.00 | 65–183 | 1.79–5.01 |  | ** |
|  | (e) General beds |  |  |  |  |  |  |  |
|  | January | 5098 | 0–0 | 0.00–0.00 | 0–100 | 0.00–1.91 |  |  |
|  | February | 5156 | 0–0 | 0.00–0.00 | 0–38 | 0.00–0.71 |  |  |
|  | March | 4974 | 0–0 | 0.00–0.00 | 33–208 | 0.64–4.01 |  | ** |
|  | April | 4604 | 0–0 | 0.00–0.00 | 299–477 | 5.89–9.38 |  | ** |
|  | May | 4402 | 0–0 | 0.00–0.00 | 462–600 | 9.24–11.98 |  | ** |
|  | June | 4649 | 0–0 | 0.00–0.00 | 193–351 | 3.86–7.01 |  | ** |
|  | (f) LTCI care beds |  |  |  |  |  |  |  |
|  | January | 356 | 0–0 | 0.00–0.00 | 118–163 | 22.78–31.28 |  | ** |
|  | February | 357 | 0–0 | 0.00–0.00 | 111–155 | 21.72–30.14 |  | ** |
|  | March | 354 | 0–0 | 0.00–0.00 | 107–150 | 21.24–29.73 |  | ** |
|  | April | 324 | 0–0 | 0.00–0.00 | 128–171 | 25.90–34.45 |  | ** |
|  | May | 317 | 0–0 | 0.00–0.00 | 131–173 | 26.74–35.29 |  | ** |
|  | June | 313 | 0–0 | 0.00–0.00 | 128–171 | 26.49–35.22 |  | ** |
|  | (3) Average length of hospital stays per patient |  |  |  |  |  |  |  |
|  | (b) Psychiatric care beds |  |  |  |  |  |  |  |
|  | January | 304 | 0–5 | 0.00–1.57 | 0–0 | 0.00–0.00 |  |  |
|  | February | 296 | 0–3 | 0.00–0.89 | 0–0 | 0.00–0.00 |  |  |
|  | March | 299 | 0–17 | 0.00–5.85 | 0–0 | 0.00–0.00 |  |  |
|  | April | 320 | 1–36 | 0.14–12.31 | 0–0 | 0.00–0.00 | * |  |
|  | May | 332 | 18–52 | 6.31–18.34 | 0–0 | 0.00–0.00 | * |  |
|  | June | 290 | 0–13 | 0.00–4.45 | 0–0 | 0.00–0.00 |  |  |
|  | (c) Tuberculosis care beds |  |  |  |  |  |  |  |
|  | January | 161 | 47–93 | 67.57–135.42 | 0–0 | 0.00–0.00 | * |  |
|  | February | 88 | 0–19 | 0.00–26.52 | 0–0 | 0.00–0.00 |  |  |
|  | March | 112 | 0–43 | 0.00–62.30 | 0–0 | 0.00–0.00 |  |  |
|  | April | 78 | 0–12 | 0.00–16.72 | 0–0 | 0.00–0.00 |  |  |
|  | May | 106 | 0–41 | 0.00–60.53 | 0–0 | 0.00–0.00 |  |  |
|  | June | 108 | 0–37 | 0.00–51.56 | 0–0 | 0.00–0.00 |  |  |
|  | (d) Long-term care beds |  |  |  |  |  |  |  |
|  | January | 113 | 0–8 | 0.00–7.16 | 0–0 | 0.00–0.00 |  |  |
|  | February | 106 | 0–1 | 0.00–0.95 | 0–0 | 0.00–0.00 |  |  |
|  | March | 108 | 0–5 | 0.00–3.92 | 0–0 | 0.00–0.00 |  |  |
|  | April | 110 | 0–4 | 0.00–3.40 | 0–0 | 0.00–0.00 |  |  |
|  | May | 127 | 0–20 | 0.00–18.38 | 0–0 | 0.00–0.00 |  |  |
|  | June | 111 | 0–5 | 0.00–3.86 | 0–0 | 0.00–0.00 |  |  |
|  | (e) General beds |  |  |  |  |  |  |  |
|  | January | 19 | 0–1 | 0.00–2.95 | 0–0 | 0.00–0.00 |  |  |
|  | February | 19 | 0–1 | 0.00–1.73 | 0–0 | 0.00–0.00 |  |  |
|  | March | 19 | 0–1 | 0.00–1.24 | 0–0 | 0.00–0.00 |  |  |
|  | April | 20 | 0–1 | 0.00–4.73 | 0–0 | 0.00–0.00 |  |  |
|  | May | 22 | 0–4 | 0.00–19.19 | 0–0 | 0.00–0.00 |  |  |
|  | June | 19 | 0–1 | 0.00–1.41 | 0–0 | 0.00–0.00 |  |  |
|  | (f) LTCI care beds |  |  |  |  |  |  |  |
|  | January | 596 | 5–121 | 1.03–25.38 | 0–0 | 0.00–0.00 | * |  |
|  | February | 544 | 0–83 | 0.00–17.74 | 0–0 | 0.00–0.00 |  |  |
|  | March | 498 | 0–4 | 0.00–0.76 | 0–0 | 0.00–0.00 |  |  |
|  | April | 299 | 0–0 | 0.00–0.00 | 96–213 | 18.62–41.55 |  | ** |
|  | May | 457 | 0–0 | 0.00–0.00 | 0–50 | 0.00–9.78 |  |  |
|  | June | 313 | 0–0 | 0.00–0.00 | 60–185 | 11.90–37.14 |  | ** |
| Nagasaki | (1) Average number of outpatients per day at hospitals |  |  |  |  |  |  |  |
|  | General hospitals |  |  |  |  |  |  |  |
|  | January | 14513 | 0–0 | 0.00–0.00 | 0–246 | 0.00–1.66 |  |  |
|  | February | 14494 | 0–0 | 0.00–0.00 | 0–433 | 0.00–2.90 |  |  |
|  | March | 14324 | 0–0 | 0.00–0.00 | 0–581 | 0.00–3.89 |  |  |
|  | April | 13316 | 0–0 | 0.00–0.00 | 432–1302 | 2.96–8.90 |  | ** |
|  | May | 11669 | 0–0 | 0.00–0.00 | 2096–3022 | 14.27–20.57 |  | ** |
|  | June | 14117 | 0–0 | 0.00–0.00 | 0–739 | 0.00–4.97 |  |  |
|  | Psychiatric hospitals |  |  |  |  |  |  |  |
|  | January | 1055 | 0–0 | 0.00–0.00 | 0–13 | 0.00–1.18 |  |  |
|  | February | 1088 | 0–3 | 0.00–0.27 | 0–0 | 0.00–0.00 |  |  |
|  | March | 1101 | 0–0 | 0.00–0.00 | 0–8 | 0.00–0.67 |  |  |
|  | April | 1089 | 0–3 | 0.00–0.23 | 0–0 | 0.00–0.00 |  |  |
|  | May | 949 | 0–0 | 0.00–0.00 | 64–141 | 5.87–12.93 |  | ** |
|  | June | 1097 | 0–5 | 0.00–0.44 | 0–0 | 0.00–0.00 |  |  |
|  | (2) Average number of hospitalized patients per day |  |  |  |  |  |  |  |
|  | (b) Psychiatric care beds |  |  |  |  |  |  |  |
|  | January | 6458 | 0–0 | 0.00–0.00 | 0–95 | 0.00–1.45 |  |  |
|  | February | 6436 | 0–0 | 0.00–0.00 | 0–116 | 0.00–1.77 |  |  |
|  | March | 6439 | 0–0 | 0.00–0.00 | 0–104 | 0.00–1.58 |  |  |
|  | April | 6400 | 0–0 | 0.00–0.00 | 0–132 | 0.00–2.01 |  |  |
|  | May | 6389 | 0–0 | 0.00–0.00 | 0–134 | 0.00–2.04 |  |  |
|  | June | 6441 | 0–0 | 0.00–0.00 | 0–80 | 0.00–1.22 |  |  |
|  | (c) Tuberculosis care beds |  |  |  |  |  |  |  |
|  | January | 18 | 0–0 | 0.00–0.00 | 0–3 | 0.00–10.29 |  |  |
|  | February | 16 | 0–0 | 0.00–0.00 | 0–3 | 0.00–14.89 |  |  |
|  | March | 17 | 0–0 | 0.00–0.00 | 0–1 | 0.00–1.16 |  |  |
|  | April | 26 | 2–11 | 12.73–65.48 | 0–0 | 0.00–0.00 | * |  |
|  | May | 23 | 0–8 | 0.00–47.47 | 0–0 | 0.00–0.00 |  |  |
|  | June | 19 | 0–3 | 0.00–18.43 | 0–0 | 0.00–0.00 |  |  |
|  | (d) Long-term care beds |  |  |  |  |  |  |  |
|  | January | 5394 | 0–9 | 0.00–0.16 | 0–0 | 0.00–0.00 |  |  |
|  | February | 5427 | 0–14 | 0.00–0.25 | 0–0 | 0.00–0.00 |  |  |
|  | March | 5409 | 0–0 | 0.00–0.00 | 0–11 | 0.00–0.19 |  |  |
|  | April | 5256 | 0–0 | 0.00–0.00 | 0–127 | 0.00–2.34 |  |  |
|  | May | 5209 | 0–0 | 0.00–0.00 | 0–134 | 0.00–2.49 |  |  |
|  | June | 5165 | 0–0 | 0.00–0.00 | 12–154 | 0.23–2.89 |  | ** |
|  | (e) General beds |  |  |  |  |  |  |  |
|  | January | 9304 | 0–0 | 0.00–0.00 | 0–59 | 0.00–0.62 |  |  |
|  | February | 9565 | 0–68 | 0.00–0.71 | 0–0 | 0.00–0.00 |  |  |
|  | March | 9230 | 0–0 | 0.00–0.00 | 0–217 | 0.00–2.29 |  |  |
|  | April | 8545 | 0–0 | 0.00–0.00 | 369–682 | 4.00–7.38 |  | ** |
|  | May | 8073 | 0–0 | 0.00–0.00 | 843–1077 | 9.21–11.77 |  | ** |
|  | June | 8650 | 0–0 | 0.00–0.00 | 235–513 | 2.56–5.59 |  | ** |
|  | (f) LTCI care beds |  |  |  |  |  |  |  |
|  | January | 187 | 0–9 | 0.00–4.97 | 0–0 | 0.00–0.00 |  |  |
|  | February | 186 | 0–11 | 0.00–6.01 | 0–0 | 0.00–0.00 |  |  |
|  | March | 188 | 0–20 | 0.00–11.25 | 0–0 | 0.00–0.00 |  |  |
|  | April | 139 | 0–0 | 0.00–0.00 | 0–23 | 0.00–13.99 |  |  |
|  | May | 122 | 0–0 | 0.00–0.00 | 0–35 | 0.00–21.81 |  |  |
|  | June | 114 | 0–0 | 0.00–0.00 | 2–40 | 1.31–25.54 |  | ** |
|  | (3) Average length of hospital stays per patient |  |  |  |  |  |  |  |
|  | (b) Psychiatric care beds |  |  |  |  |  |  |  |
|  | January | 389 | 0–22 | 0.00–5.90 | 0–0 | 0.00–0.00 |  |  |
|  | February | 347 | 0–0 | 0.00–0.00 | 0–15 | 0.00–4.07 |  |  |
|  | March | 375 | 0–22 | 0.00–5.96 | 0–0 | 0.00–0.00 |  |  |
|  | April | 409 | 18–56 | 4.96–15.71 | 0–0 | 0.00–0.00 | * |  |
|  | May | 429 | 41–79 | 11.47–22.42 | 0–0 | 0.00–0.00 | * |  |
|  | June | 362 | 0–14 | 0.00–3.72 | 0–0 | 0.00–0.00 |  |  |
|  | (c) Tuberculosis care beds |  |  |  |  |  |  |  |
|  | January | 53 | 0–0 | 0.00–0.00 | 0–14 | 0.00–19.69 |  |  |
|  | February | 59 | 0–9 | 0.00–16.86 | 0–0 | 0.00–0.00 |  |  |
|  | March | 35 | 0–0 | 0.00–0.00 | 0–16 | 0.00–30.71 |  |  |
|  | April | 40 | 0–0 | 0.00–0.00 | 0–10 | 0.00–19.55 |  |  |
|  | May | 55 | 0–6 | 0.00–11.78 | 0–0 | 0.00–0.00 |  |  |
|  | June | 49 | 0–3 | 0.00–5.70 | 0–0 | 0.00–0.00 |  |  |
|  | (d) Long-term care beds |  |  |  |  |  |  |  |
|  | January | 89 | 0–3 | 0.00–2.52 | 0–0 | 0.00–0.00 |  |  |
|  | February | 90 | 0–5 | 0.00–5.20 | 0–0 | 0.00–0.00 |  |  |
|  | March | 87 | 0–4 | 0.00–3.75 | 0–0 | 0.00–0.00 |  |  |
|  | April | 91 | 0–6 | 0.00–6.44 | 0–0 | 0.00–0.00 |  |  |
|  | May | 101 | 0–15 | 0.00–16.23 | 0–0 | 0.00–0.00 |  |  |
|  | June | 88 | 0–2 | 0.00–1.87 | 0–0 | 0.00–0.00 |  |  |
|  | (e) General beds |  |  |  |  |  |  |  |
|  | January | 18 | 0–1 | 0.00–4.30 | 0–0 | 0.00–0.00 |  |  |
|  | February | 18 | 0–1 | 0.00–3.18 | 0–0 | 0.00–0.00 |  |  |
|  | March | 18 | 0–1 | 0.00–2.39 | 0–0 | 0.00–0.00 |  |  |
|  | April | 18 | 0–1 | 0.00–1.25 | 0–0 | 0.00–0.00 |  |  |
|  | May | 19 | 0–3 | 0.00–12.74 | 0–0 | 0.00–0.00 |  |  |
|  | June | 17 | 0–1 | 0.00–1.24 | 0–0 | 0.00–0.00 |  |  |
|  | (f) LTCI care beds |  |  |  |  |  |  |  |
|  | January | 298 | 0–0 | 0.00–0.00 | 0–148 | 0.00–33.12 |  |  |
|  | February | 433 | 0–35 | 0.00–8.80 | 0–0 | 0.00–0.00 |  |  |
|  | March | 191 | 0–0 | 0.00–0.00 | 37–173 | 10.18–47.47 |  | ** |
|  | April | 199 | 0–0 | 0.00–0.00 | 9–151 | 2.32–43.03 |  | ** |
|  | May | 314 | 0–0 | 0.00–0.00 | 0–115 | 0.00–26.70 |  |  |
|  | June | 327 | 0–0 | 0.00–0.00 | 0–132 | 0.00–28.71 |  |  |
| Kumamoto | (1) Average number of outpatients per day at hospitals |  |  |  |  |  |  |  |
|  | General hospitals |  |  |  |  |  |  |  |
|  | January | 17619 | 0–0 | 0.00–0.00 | 0–891 | 0.00–4.81 |  |  |
|  | February | 17271 | 0–0 | 0.00–0.00 | 168–1407 | 0.90–7.53 |  | ** |
|  | March | 16713 | 0–0 | 0.00–0.00 | 795–1985 | 4.25–10.61 |  | ** |
|  | April | 15686 | 0–0 | 0.00–0.00 | 1429–2617 | 7.81–14.30 |  | ** |
|  | May | 14189 | 0–0 | 0.00–0.00 | 2940–4115 | 16.06–22.48 |  | ** |
|  | June | 17190 | 0–0 | 0.00–0.00 | 129–1399 | 0.69–7.52 |  | ** |
|  | Psychiatric hospitals |  |  |  |  |  |  |  |
|  | January | 1736 | 0–0 | 0.00–0.00 | 0–87 | 0.00–4.74 |  |  |
|  | February | 1770 | 0–0 | 0.00–0.00 | 0–77 | 0.00–4.17 |  |  |
|  | March | 1785 | 0–0 | 0.00–0.00 | 0–104 | 0.00–5.46 |  |  |
|  | April | 1767 | 0–0 | 0.00–0.00 | 0–76 | 0.00–4.09 |  |  |
|  | May | 1531 | 0–0 | 0.00–0.00 | 161–317 | 8.71–17.13 |  | ** |
|  | June | 1818 | 0–0 | 0.00–0.00 | 0–38 | 0.00–2.01 |  |  |
|  | (2) Average number of hospitalized patients per day |  |  |  |  |  |  |  |
|  | (b) Psychiatric care beds |  |  |  |  |  |  |  |
|  | January | 7599 | 0–0 | 0.00–0.00 | 0–116 | 0.00–1.49 |  |  |
|  | February | 7626 | 0–0 | 0.00–0.00 | 0–99 | 0.00–1.27 |  |  |
|  | March | 7632 | 0–0 | 0.00–0.00 | 0–89 | 0.00–1.15 |  |  |
|  | April | 7602 | 0–0 | 0.00–0.00 | 0–95 | 0.00–1.23 |  |  |
|  | May | 7544 | 0–0 | 0.00–0.00 | 0–140 | 0.00–1.81 |  |  |
|  | June | 7625 | 0–0 | 0.00–0.00 | 0–69 | 0.00–0.89 |  |  |
|  | (c) Tuberculosis care beds |  |  |  |  |  |  |  |
|  | January | 19 | 0–3 | 0.00–15.23 | 0–0 | 0.00–0.00 |  |  |
|  | February | 23 | 0–7 | 0.00–43.73 | 0–0 | 0.00–0.00 |  |  |
|  | March | 20 | 0–5 | 0.00–27.90 | 0–0 | 0.00–0.00 |  |  |
|  | April | 20 | 0–5 | 0.00–31.88 | 0–0 | 0.00–0.00 |  |  |
|  | May | 17 | 0–1 | 0.00–2.57 | 0–0 | 0.00–0.00 |  |  |
|  | June | 14 | 0–0 | 0.00–0.00 | 0–4 | 0.00–18.73 |  |  |
|  | (d) Long-term care beds |  |  |  |  |  |  |  |
|  | January | 7044 | 0–0 | 0.00–0.00 | 74–310 | 1.01–4.20 |  | ** |
|  | February | 7086 | 0–0 | 0.00–0.00 | 0–236 | 0.00–3.22 |  |  |
|  | March | 6991 | 0–0 | 0.00–0.00 | 48–285 | 0.66–3.90 |  | ** |
|  | April | 6470 | 0–0 | 0.00–0.00 | 462–704 | 6.44–9.80 |  | ** |
|  | May | 6332 | 0–0 | 0.00–0.00 | 511–749 | 7.22–10.57 |  | ** |
|  | June | 6258 | 0–0 | 0.00–0.00 | 519–757 | 7.40–10.79 |  | ** |
|  | (e) General beds |  |  |  |  |  |  |  |
|  | January | 12954 | 0–75 | 0.00–0.58 | 0–0 | 0.00–0.00 |  |  |
|  | February | 13253 | 0–216 | 0.00–1.66 | 0–0 | 0.00–0.00 |  |  |
|  | March | 12636 | 0–0 | 0.00–0.00 | 6–351 | 0.05–2.70 |  | ** |
|  | April | 12018 | 0–0 | 0.00–0.00 | 365–756 | 2.86–5.92 |  | ** |
|  | May | 11637 | 0–0 | 0.00–0.00 | 751–1018 | 5.93–8.04 |  | ** |
|  | June | 12109 | 0–0 | 0.00–0.00 | 223–559 | 1.76–4.41 |  | ** |
|  | (f) LTCI care beds |  |  |  |  |  |  |  |
|  | January | 948 | 0–0 | 0.00–0.00 | 54–153 | 4.91–13.88 |  | ** |
|  | February | 959 | 0–0 | 0.00–0.00 | 13–115 | 1.21–10.68 |  | ** |
|  | March | 925 | 0–0 | 0.00–0.00 | 9–114 | 0.87–10.95 |  | ** |
|  | April | 564 | 0–0 | 0.00–0.00 | 353–454 | 34.69–44.58 |  | ** |
|  | May | 532 | 0–0 | 0.00–0.00 | 344–451 | 35.02–45.84 |  | ** |
|  | June | 479 | 0–0 | 0.00–0.00 | 378–482 | 39.35–50.14 |  | ** |
|  | (3) Average length of hospital stays per patient |  |  |  |  |  |  |  |
|  | (b) Psychiatric care beds |  |  |  |  |  |  |  |
|  | January | 313 | 0–0 | 0.00–0.00 | 0–4 | 0.00–1.00 |  |  |
|  | February | 299 | 0–0 | 0.00–0.00 | 0–10 | 0.00–3.15 |  |  |
|  | March | 304 | 0–4 | 0.00–1.19 | 0–0 | 0.00–0.00 |  |  |
|  | April | 321 | 0–23 | 0.00–7.64 | 0–0 | 0.00–0.00 |  |  |
|  | May | 359 | 27–63 | 9.00–20.95 | 0–0 | 0.00–0.00 | * |  |
|  | June | 305 | 0–9 | 0.00–2.80 | 0–0 | 0.00–0.00 |  |  |
|  | (c) Tuberculosis care beds |  |  |  |  |  |  |  |
|  | January | 58 | 0–0 | 0.00–0.00 | 0–7 | 0.00–10.26 |  |  |
|  | February | 69 | 0–9 | 0.00–13.45 | 0–0 | 0.00–0.00 |  |  |
|  | March | 40 | 0–0 | 0.00–0.00 | 0–23 | 0.00–36.18 |  |  |
|  | April | 49 | 0–0 | 0.00–0.00 | 0–13 | 0.00–20.26 |  |  |
|  | May | 46 | 0–0 | 0.00–0.00 | 0–20 | 0.00–29.93 |  |  |
|  | June | 45 | 0–0 | 0.00–0.00 | 0–24 | 0.00–34.62 |  |  |
|  | (d) Long-term care beds |  |  |  |  |  |  |  |
|  | January | 132 | 0–12 | 0.00–9.22 | 0–0 | 0.00–0.00 |  |  |
|  | February | 127 | 0–8 | 0.00–6.69 | 0–0 | 0.00–0.00 |  |  |
|  | March | 133 | 0–17 | 0.00–14.62 | 0–0 | 0.00–0.00 |  |  |
|  | April | 118 | 0–0 | 0.00–0.00 | 0–1 | 0.00–0.48 |  |  |
|  | May | 137 | 0–17 | 0.00–13.70 | 0–0 | 0.00–0.00 |  |  |
|  | June | 126 | 0–4 | 0.00–3.19 | 0–0 | 0.00–0.00 |  |  |
|  | (e) General beds |  |  |  |  |  |  |  |
|  | January | 21 | 0–1 | 0.00–3.48 | 0–0 | 0.00–0.00 |  |  |
|  | February | 20 | 0–1 | 0.00–1.81 | 0–0 | 0.00–0.00 |  |  |
|  | March | 20 | 0–1 | 0.00–4.10 | 0–0 | 0.00–0.00 |  |  |
|  | April | 20 | 0–1 | 0.00–3.14 | 0–0 | 0.00–0.00 |  |  |
|  | May | 22 | 0–3 | 0.00–12.76 | 0–0 | 0.00–0.00 |  |  |
|  | June | 19 | 0–0 | 0.00–0.00 | 0–1 | 0.00–0.07 |  |  |
|  | (f) LTCI care beds |  |  |  |  |  |  |  |
|  | January | 259 | 0–33 | 0.00–14.43 | 0–0 | 0.00–0.00 |  |  |
|  | February | 321 | 42–98 | 18.45–43.26 | 0–0 | 0.00–0.00 | * |  |
|  | March | 262 | 0–47 | 0.00–21.68 | 0–0 | 0.00–0.00 |  |  |
|  | April | 94 | 0–0 | 0.00–0.00 | 80–128 | 35.76–57.55 |  | ** |
|  | May | 212 | 0–0 | 0.00–0.00 | 0–6 | 0.00–2.70 |  |  |
|  | June | 170 | 0–0 | 0.00–0.00 | 2–49 | 0.91–22.24 |  | ** |
| Oita | (1) Average number of outpatients per day at hospitals |  |  |  |  |  |  |  |
|  | General hospitals |  |  |  |  |  |  |  |
|  | January | 13164 | 0–0 | 0.00–0.00 | 0–523 | 0.00–3.82 |  |  |
|  | February | 13177 | 0–0 | 0.00–0.00 | 0–613 | 0.00–4.44 |  |  |
|  | March | 12608 | 0–0 | 0.00–0.00 | 418–1178 | 3.03–8.54 |  | ** |
|  | April | 11770 | 0–0 | 0.00–0.00 | 1018–1730 | 7.54–12.81 |  | ** |
|  | May | 10881 | 0–0 | 0.00–0.00 | 1931–2664 | 14.26–19.66 |  | ** |
|  | June | 13025 | 0–0 | 0.00–0.00 | 0–736 | 0.00–5.34 |  |  |
|  | Psychiatric hospitals |  |  |  |  |  |  |  |
|  | January | 1035 | 0–0 | 0.00–0.00 | 0–64 | 0.00–5.76 |  |  |
|  | February | 1099 | 0–0 | 0.00–0.00 | 0–11 | 0.00–0.93 |  |  |
|  | March | 1089 | 0–0 | 0.00–0.00 | 0–52 | 0.00–4.50 |  |  |
|  | April | 1082 | 0–0 | 0.00–0.00 | 0–35 | 0.00–3.08 |  |  |
|  | May | 985 | 0–0 | 0.00–0.00 | 62–143 | 5.50–12.67 |  | ** |
|  | June | 1137 | 0–2 | 0.00–0.10 | 0–0 | 0.00–0.00 |  |  |
|  | (2) Average number of hospitalized patients per day |  |  |  |  |  |  |  |
|  | (b) Psychiatric care beds |  |  |  |  |  |  |  |
|  | January | 4703 | 0–20 | 0.00–0.42 | 0–0 | 0.00–0.00 |  |  |
|  | February | 4685 | 0–2 | 0.00–0.04 | 0–0 | 0.00–0.00 |  |  |
|  | March | 4683 | 0–0 | 0.00–0.00 | 0–3 | 0.00–0.06 |  |  |
|  | April | 4670 | 0–0 | 0.00–0.00 | 0–13 | 0.00–0.27 |  |  |
|  | May | 4652 | 0–0 | 0.00–0.00 | 0–28 | 0.00–0.59 |  |  |
|  | June | 4690 | 0–8 | 0.00–0.16 | 0–0 | 0.00–0.00 |  |  |
|  | (c) Tuberculosis care beds |  |  |  |  |  |  |  |
|  | January | 17 | 0–0 | 0.00–0.00 | 0–7 | 0.00–27.15 |  |  |
|  | February | 17 | 0–0 | 0.00–0.00 | 0–5 | 0.00–20.87 |  |  |
|  | March | 21 | 0–1 | 0.00–1.33 | 0–0 | 0.00–0.00 |  |  |
|  | April | 20 | 0–0 | 0.00–0.00 | 0–1 | 0.00–1.52 |  |  |
|  | May | 23 | 0–3 | 0.00–12.81 | 0–0 | 0.00–0.00 |  |  |
|  | June | 23 | 0–3 | 0.00–14.36 | 0–0 | 0.00–0.00 |  |  |
|  | (d) Long-term care beds |  |  |  |  |  |  |  |
|  | January | 2266 | 0–0 | 0.00–0.00 | 0–115 | 0.00–4.80 |  |  |
|  | February | 2282 | 0–0 | 0.00–0.00 | 0–91 | 0.00–3.83 |  |  |
|  | March | 2285 | 0–0 | 0.00–0.00 | 0–63 | 0.00–2.65 |  |  |
|  | April | 2078 | 0–0 | 0.00–0.00 | 86–235 | 3.72–10.15 |  | ** |
|  | May | 2066 | 0–0 | 0.00–0.00 | 75–219 | 3.28–9.58 |  | ** |
|  | June | 2070 | 0–0 | 0.00–0.00 | 54–199 | 2.38–8.75 |  | ** |
|  | (e) General beds |  |  |  |  |  |  |  |
|  | January | 9532 | 0–0 | 0.00–0.00 | 0–128 | 0.00–1.32 |  |  |
|  | February | 9614 | 0–0 | 0.00–0.00 | 0–157 | 0.00–1.60 |  |  |
|  | March | 9217 | 0–0 | 0.00–0.00 | 309–566 | 3.16–5.78 |  | ** |
|  | April | 8601 | 0–0 | 0.00–0.00 | 714–1000 | 7.44–10.41 |  | ** |
|  | May | 8401 | 0–0 | 0.00–0.00 | 893–1129 | 9.37–11.84 |  | ** |
|  | June | 8777 | 0–0 | 0.00–0.00 | 528–767 | 5.53–8.03 |  | ** |
|  | (f) LTCI care beds |  |  |  |  |  |  |  |
|  | January | 158 | 0–3 | 0.00–1.45 | 0–0 | 0.00–0.00 |  |  |
|  | February | 154 | 0–2 | 0.00–1.00 | 0–0 | 0.00–0.00 |  |  |
|  | March | 154 | 0–4 | 0.00–2.33 | 0–0 | 0.00–0.00 |  |  |
|  | April | 18 | 0–0 | 0.00–0.00 | 101–130 | 68.54–87.78 |  | ** |
|  | May | 18 | 0–0 | 0.00–0.00 | 99–127 | 68.64–87.52 |  | ** |
|  | June | 18 | 0–0 | 0.00–0.00 | 97–124 | 68.40–87.31 |  | ** |
|  | (3) Average length of hospital stays per patient |  |  |  |  |  |  |  |
|  | (b) Psychiatric care beds |  |  |  |  |  |  |  |
|  | January | 429 | 0–17 | 0.00–4.03 | 0–0 | 0.00–0.00 |  |  |
|  | February | 454 | 0–46 | 0.00–11.22 | 0–0 | 0.00–0.00 |  |  |
|  | March | 427 | 0–34 | 0.00–8.51 | 0–0 | 0.00–0.00 |  |  |
|  | April | 448 | 0–46 | 0.00–11.41 | 0–0 | 0.00–0.00 |  |  |
|  | May | 478 | 34–84 | 8.50–21.17 | 0–0 | 0.00–0.00 | * |  |
|  | June | 413 | 0–21 | 0.00–5.13 | 0–0 | 0.00–0.00 |  |  |
|  | (c) Tuberculosis care beds |  |  |  |  |  |  |  |
|  | January | 73 | 0–0 | 0.00–0.00 | 0–30 | 0.00–28.37 |  |  |
|  | February | 112 | 0–4 | 0.00–3.26 | 0–0 | 0.00–0.00 |  |  |
|  | March | 141 | 0–37 | 0.00–35.04 | 0–0 | 0.00–0.00 |  |  |
|  | April | 60 | 0–0 | 0.00–0.00 | 0–43 | 0.00–41.33 |  |  |
|  | May | 117 | 0–23 | 0.00–24.34 | 0–0 | 0.00–0.00 |  |  |
|  | June | 97 | 0–4 | 0.00–3.20 | 0–0 | 0.00–0.00 |  |  |
|  | (d) Long-term care beds |  |  |  |  |  |  |  |
|  | January | 101 | 0–9 | 0.00–9.07 | 0–0 | 0.00–0.00 |  |  |
|  | February | 93 | 0–1 | 0.00–0.05 | 0–0 | 0.00–0.00 |  |  |
|  | March | 94 | 0–5 | 0.00–5.14 | 0–0 | 0.00–0.00 |  |  |
|  | April | 100 | 0–8 | 0.00–8.02 | 0–0 | 0.00–0.00 |  |  |
|  | May | 106 | 0–16 | 0.00–16.46 | 0–0 | 0.00–0.00 |  |  |
|  | June | 96 | 0–6 | 0.00–5.56 | 0–0 | 0.00–0.00 |  |  |
|  | (e) General beds |  |  |  |  |  |  |  |
|  | January | 20 | 0–1 | 0.00–2.49 | 0–0 | 0.00–0.00 |  |  |
|  | February | 20 | 0–0 | 0.00–0.00 | 0–1 | 0.00–0.56 |  |  |
|  | March | 20 | 0–1 | 0.00–2.68 | 0–0 | 0.00–0.00 |  |  |
|  | April | 21 | 0–2 | 0.00–5.94 | 0–0 | 0.00–0.00 |  |  |
|  | May | 21 | 0–3 | 0.00–11.76 | 0–0 | 0.00–0.00 |  |  |
|  | June | 19 | 0–0 | 0.00–0.00 | 0–1 | 0.00–1.02 |  |  |
|  | (f) LTCI care beds |  |  |  |  |  |  |  |
|  | January | 155 | 0–0 | 0.00–0.00 | 0–24 | 0.00–13.29 |  |  |
|  | February | 121 | 0–0 | 0.00–0.00 | 0–44 | 0.00–26.43 |  |  |
|  | March | 87 | 0–0 | 0.00–0.00 | 10–61 | 6.18–41.03 |  | ** |
|  | April | 537 | 327–391 | 223.52–267.06 | 0–0 | 0.00–0.00 | * |  |
|  | May | 0 | NA | NA | NA | NA |  |  |
|  | June | 0 | NA | NA | NA | NA |  |  |
| Miyazaki | (1) Average number of outpatients per day at hospitals |  |  |  |  |  |  |  |
|  | General hospitals |  |  |  |  |  |  |  |
|  | January | 11094 | 0–0 | 0.00–0.00 | 0–496 | 0.00–4.28 |  |  |
|  | February | 11242 | 0–0 | 0.00–0.00 | 0–444 | 0.00–3.79 |  |  |
|  | March | 10809 | 0–0 | 0.00–0.00 | 161–963 | 1.37–8.18 |  | ** |
|  | April | 10374 | 0–0 | 0.00–0.00 | 323–1060 | 2.83–9.26 |  | ** |
|  | May | 9352 | 0–0 | 0.00–0.00 | 1375–2156 | 11.95–18.73 |  | ** |
|  | June | 11422 | 0–0 | 0.00–0.00 | 0–181 | 0.00–1.55 |  |  |
|  | Psychiatric hospitals |  |  |  |  |  |  |  |
|  | January | 884 | 0–0 | 0.00–0.00 | 1–65 | 0.11–6.81 |  | ** |
|  | February | 921 | 0–0 | 0.00–0.00 | 0–38 | 0.00–3.90 |  |  |
|  | March | 923 | 0–0 | 0.00–0.00 | 0–57 | 0.00–5.76 |  |  |
|  | April | 945 | 0–0 | 0.00–0.00 | 0–19 | 0.00–1.88 |  |  |
|  | May | 843 | 0–0 | 0.00–0.00 | 56–125 | 5.79–12.86 |  | ** |
|  | June | 955 | 0–0 | 0.00–0.00 | 0–12 | 0.00–1.20 |  |  |
|  | (2) Average number of hospitalized patients per day |  |  |  |  |  |  |  |
|  | (b) Psychiatric care beds |  |  |  |  |  |  |  |
|  | January | 5107 | 0–75 | 0.00–1.49 | 0–0 | 0.00–0.00 |  |  |
|  | February | 5095 | 0–57 | 0.00–1.13 | 0–0 | 0.00–0.00 |  |  |
|  | March | 5074 | 0–31 | 0.00–0.60 | 0–0 | 0.00–0.00 |  |  |
|  | April | 5061 | 0–25 | 0.00–0.48 | 0–0 | 0.00–0.00 |  |  |
|  | May | 5067 | 0–26 | 0.00–0.50 | 0–0 | 0.00–0.00 |  |  |
|  | June | 5109 | 0–62 | 0.00–1.22 | 0–0 | 0.00–0.00 |  |  |
|  | (c) Tuberculosis care beds |  |  |  |  |  |  |  |
|  | January | 11 | 0–0 | 0.00–0.00 | 0–3 | 0.00–15.66 |  |  |
|  | February | 17 | 0–5 | 0.00–34.96 | 0–0 | 0.00–0.00 |  |  |
|  | March | 13 | 0–1 | 0.00–6.65 | 0–0 | 0.00–0.00 |  |  |
|  | April | 13 | 0–2 | 0.00–8.60 | 0–0 | 0.00–0.00 |  |  |
|  | May | 16 | 0–5 | 0.00–41.68 | 0–0 | 0.00–0.00 |  |  |
|  | June | 17 | 0–7 | 0.00–54.83 | 0–0 | 0.00–0.00 |  |  |
|  | (d) Long-term care beds |  |  |  |  |  |  |  |
|  | January | 3084 | 0–0 | 0.00–0.00 | 0–42 | 0.00–1.34 |  |  |
|  | February | 3132 | 0–0 | 0.00–0.00 | 0–11 | 0.00–0.34 |  |  |
|  | March | 3107 | 0–0 | 0.00–0.00 | 0–39 | 0.00–1.22 |  |  |
|  | April | 3038 | 0–0 | 0.00–0.00 | 0–80 | 0.00–2.55 |  |  |
|  | May | 2957 | 0–0 | 0.00–0.00 | 32–141 | 1.03–4.53 |  | ** |
|  | June | 2923 | 0–0 | 0.00–0.00 | 54–162 | 1.75–5.25 |  | ** |
|  | (e) General beds |  |  |  |  |  |  |  |
|  | January | 6546 | 0–0 | 0.00–0.00 | 0–149 | 0.00–2.22 |  |  |
|  | February | 6796 | 0–12 | 0.00–0.17 | 0–0 | 0.00–0.00 |  |  |
|  | March | 6471 | 0–0 | 0.00–0.00 | 92–289 | 1.36–4.26 |  | ** |
|  | April | 6079 | 0–0 | 0.00–0.00 | 324–543 | 4.89–8.19 |  | ** |
|  | May | 5814 | 0–0 | 0.00–0.00 | 594–759 | 9.04–11.54 |  | ** |
|  | June | 6136 | 0–0 | 0.00–0.00 | 270–460 | 4.09–6.97 |  | ** |
|  | (f) LTCI care beds |  |  |  |  |  |  |  |
|  | January | 521 | 0–0 | 0.00–0.00 | 0–28 | 0.00–5.09 |  |  |
|  | February | 519 | 0–0 | 0.00–0.00 | 0–27 | 0.00–4.83 |  |  |
|  | March | 532 | 0–0 | 0.00–0.00 | 0–10 | 0.00–1.69 |  |  |
|  | April | 491 | 0–0 | 0.00–0.00 | 2–47 | 0.37–8.72 |  | ** |
|  | May | 488 | 0–0 | 0.00–0.00 | 4–49 | 0.75–9.09 |  | ** |
|  | June | 490 | 0–0 | 0.00–0.00 | 0–45 | 0.00–8.31 |  |  |
|  | (3) Average length of hospital stays per patient |  |  |  |  |  |  |  |
|  | (b) Psychiatric care beds |  |  |  |  |  |  |  |
|  | January | 359 | 0–15 | 0.00–4.30 | 0–0 | 0.00–0.00 |  |  |
|  | February | 360 | 0–24 | 0.00–6.86 | 0–0 | 0.00–0.00 |  |  |
|  | March | 353 | 0–23 | 0.00–6.68 | 0–0 | 0.00–0.00 |  |  |
|  | April | 374 | 8–45 | 2.28–13.62 | 0–0 | 0.00–0.00 | * |  |
|  | May | 352 | 0–21 | 0.00–6.35 | 0–0 | 0.00–0.00 |  |  |
|  | June | 346 | 0–17 | 0.00–4.95 | 0–0 | 0.00–0.00 |  |  |
|  | (c) Tuberculosis care beds |  |  |  |  |  |  |  |
|  | January | 42 | 0–4 | 0.00–8.61 | 0–0 | 0.00–0.00 |  |  |
|  | February | 76 | 17–39 | 45.40–105.78 | 0–0 | 0.00–0.00 | * |  |
|  | March | 26 | 0–0 | 0.00–0.00 | 0–15 | 0.00–35.94 |  |  |
|  | April | 25 | 0–0 | 0.00–0.00 | 4–22 | 8.44–45.68 |  | ** |
|  | May | 145 | 74–99 | 159.03–212.44 | 0–0 | 0.00–0.00 | * |  |
|  | June | 90 | 19–46 | 40.41–100.25 | 0–0 | 0.00–0.00 | * |  |
|  | (d) Long-term care beds |  |  |  |  |  |  |  |
|  | January | 112 | 0–8 | 0.00–6.99 | 0–0 | 0.00–0.00 |  |  |
|  | February | 109 | 0–4 | 0.00–3.58 | 0–0 | 0.00–0.00 |  |  |
|  | March | 117 | 0–13 | 0.00–11.49 | 0–0 | 0.00–0.00 |  |  |
|  | April | 114 | 0–6 | 0.00–4.62 | 0–0 | 0.00–0.00 |  |  |
|  | May | 126 | 0–17 | 0.00–15.35 | 0–0 | 0.00–0.00 |  |  |
|  | June | 108 | 0–0 | 0.00–0.00 | 0–3 | 0.00–2.20 |  |  |
|  | (e) General beds |  |  |  |  |  |  |  |
|  | January | 18 | 0–1 | 0.00–3.14 | 0–0 | 0.00–0.00 |  |  |
|  | February | 18 | 0–1 | 0.00–1.13 | 0–0 | 0.00–0.00 |  |  |
|  | March | 17 | 0–1 | 0.00–0.69 | 0–0 | 0.00–0.00 |  |  |
|  | April | 17 | 0–0 | 0.00–0.00 | 0–1 | 0.00–0.54 |  |  |
|  | May | 19 | 0–2 | 0.00–9.77 | 0–0 | 0.00–0.00 |  |  |
|  | June | 17 | 0–0 | 0.00–0.00 | 0–1 | 0.00–1.97 |  |  |
|  | (f) LTCI care beds |  |  |  |  |  |  |  |
|  | January | 482 | 0–74 | 0.00–18.11 | 0–0 | 0.00–0.00 |  |  |
|  | February | 342 | 0–0 | 0.00–0.00 | 0–75 | 0.00–17.98 |  |  |
|  | March | 429 | 0–23 | 0.00–5.54 | 0–0 | 0.00–0.00 |  |  |
|  | April | 409 | 0–0 | 0.00–0.00 | 0–8 | 0.00–1.90 |  |  |
|  | May | 488 | 0–70 | 0.00–16.72 | 0–0 | 0.00–0.00 |  |  |
|  | June | 306 | 0–0 | 0.00–0.00 | 11–123 | 2.50–28.54 |  | ** |
| Kagoshima | (1) Average number of outpatients per day at hospitals |  |  |  |  |  |  |  |
|  | General hospitals |  |  |  |  |  |  |  |
|  | January | 17795 | 0–0 | 0.00–0.00 | 0–815 | 0.00–4.38 |  |  |
|  | February | 17767 | 0–0 | 0.00–0.00 | 0–814 | 0.00–4.38 |  |  |
|  | March | 17561 | 0–0 | 0.00–0.00 | 116–1119 | 0.62–5.99 |  | ** |
|  | April | 16899 | 0–0 | 0.00–0.00 | 438–1450 | 2.39–7.90 |  | ** |
|  | May | 15102 | 0–0 | 0.00–0.00 | 2441–3420 | 13.18–18.46 |  | ** |
|  | June | 17935 | 0–0 | 0.00–0.00 | 0–719 | 0.00–3.85 |  |  |
|  | Psychiatric hospitals |  |  |  |  |  |  |  |
|  | January | 1468 | 0–0 | 0.00–0.00 | 0–40 | 0.00–2.61 |  |  |
|  | February | 1525 | 0–0 | 0.00–0.00 | 0–2 | 0.00–0.07 |  |  |
|  | March | 1554 | 0–0 | 0.00–0.00 | 0–10 | 0.00–0.60 |  |  |
|  | April | 1564 | 0–21 | 0.00–1.35 | 0–0 | 0.00–0.00 |  |  |
|  | May | 1400 | 0–0 | 0.00–0.00 | 43–157 | 2.76–10.06 |  | ** |
|  | June | 1564 | 0–5 | 0.00–0.31 | 0–0 | 0.00–0.00 |  |  |
|  | (2) Average number of hospitalized patients per day |  |  |  |  |  |  |  |
|  | (b) Psychiatric care beds |  |  |  |  |  |  |  |
|  | January | 8354 | 0–0 | 0.00–0.00 | 0–40 | 0.00–0.48 |  |  |
|  | February | 8381 | 0–0 | 0.00–0.00 | 0–7 | 0.00–0.08 |  |  |
|  | March | 8318 | 0–0 | 0.00–0.00 | 0–70 | 0.00–0.83 |  |  |
|  | April | 8292 | 0–0 | 0.00–0.00 | 0–96 | 0.00–1.14 |  |  |
|  | May | 8267 | 0–0 | 0.00–0.00 | 0–120 | 0.00–1.42 |  |  |
|  | June | 8301 | 0–0 | 0.00–0.00 | 0–89 | 0.00–1.06 |  |  |
|  | (c) Tuberculosis care beds |  |  |  |  |  |  |  |
|  | January | 36 | 0–0 | 0.00–0.00 | 0–2 | 0.00–3.32 |  |  |
|  | February | 36 | 0–0 | 0.00–0.00 | 0–1 | 0.00–0.97 |  |  |
|  | March | 38 | 0–1 | 0.00–2.60 | 0–0 | 0.00–0.00 |  |  |
|  | April | 33 | 0–0 | 0.00–0.00 | 0–5 | 0.00–11.24 |  |  |
|  | May | 34 | 0–0 | 0.00–0.00 | 0–4 | 0.00–8.42 |  |  |
|  | June | 37 | 0–2 | 0.00–3.90 | 0–0 | 0.00–0.00 |  |  |
|  | (d) Long-term care beds |  |  |  |  |  |  |  |
|  | January | 6649 | 0–0 | 0.00–0.00 | 162–370 | 2.31–5.26 |  | ** |
|  | February | 6651 | 0–0 | 0.00–0.00 | 200–395 | 2.84–5.60 |  | ** |
|  | March | 6667 | 0–0 | 0.00–0.00 | 175–365 | 2.49–5.19 |  | ** |
|  | April | 6366 | 0–0 | 0.00–0.00 | 393–594 | 5.65–8.53 |  | ** |
|  | May | 6273 | 0–0 | 0.00–0.00 | 418–608 | 6.08–8.83 |  | ** |
|  | June | 6146 | 0–0 | 0.00–0.00 | 488–676 | 7.15–9.90 |  | ** |
|  | (e) General beds |  |  |  |  |  |  |  |
|  | January | 11781 | 0–0 | 0.00–0.00 | 0–334 | 0.00–2.75 |  |  |
|  | February | 12043 | 0–141 | 0.00–1.18 | 0–0 | 0.00–0.00 |  |  |
|  | March | 11675 | 0–0 | 0.00–0.00 | 0–203 | 0.00–1.70 |  |  |
|  | April | 11118 | 0–0 | 0.00–0.00 | 592–973 | 4.90–8.05 |  | ** |
|  | May | 10793 | 0–0 | 0.00–0.00 | 922–1182 | 7.70–9.86 |  | ** |
|  | June | 11229 | 0–0 | 0.00–0.00 | 482–801 | 4.01–6.65 |  | ** |
|  | (f) LTCI care beds |  |  |  |  |  |  |  |
|  | January | 269 | 0–0 | 0.00–0.00 | 133–205 | 28.08–43.21 |  | ** |
|  | February | 284 | 0–0 | 0.00–0.00 | 93–171 | 20.46–37.53 |  | ** |
|  | March | 282 | 0–0 | 0.00–0.00 | 88–165 | 19.69–36.90 |  | ** |
|  | April | 186 | 0–0 | 0.00–0.00 | 160–242 | 37.41–56.51 |  | ** |
|  | May | 187 | 0–0 | 0.00–0.00 | 142–226 | 34.42–54.67 |  | ** |
|  | June | 193 | 0–0 | 0.00–0.00 | 121–205 | 30.41–51.49 |  | ** |
|  | (3) Average length of hospital stays per patient |  |  |  |  |  |  |  |
|  | (b) Psychiatric care beds |  |  |  |  |  |  |  |
|  | January | 356 | 0–0 | 0.00–0.00 | 0–5 | 0.00–1.14 |  |  |
|  | February | 363 | 0–13 | 0.00–3.43 | 0–0 | 0.00–0.00 |  |  |
|  | March | 353 | 0–16 | 0.00–4.74 | 0–0 | 0.00–0.00 |  |  |
|  | April | 355 | 0–19 | 0.00–5.56 | 0–0 | 0.00–0.00 |  |  |
|  | May | 410 | 39–76 | 11.45–22.42 | 0–0 | 0.00–0.00 | * |  |
|  | June | 349 | 0–13 | 0.00–3.72 | 0–0 | 0.00–0.00 |  |  |
|  | (c) Tuberculosis care beds |  |  |  |  |  |  |  |
|  | January | 245 | 46–100 | 31.54–68.28 | 0–0 | 0.00–0.00 | * |  |
|  | February | 130 | 0–0 | 0.00–0.00 | 0–16 | 0.00–10.49 |  |  |
|  | March | 213 | 6–66 | 3.59–44.67 | 0–0 | 0.00–0.00 | * |  |
|  | April | 166 | 0–20 | 0.00–13.28 | 0–0 | 0.00–0.00 |  |  |
|  | May | 176 | 0–11 | 0.00–6.62 | 0–0 | 0.00–0.00 |  |  |
|  | June | 314 | 86–150 | 52.18–90.98 | 0–0 | 0.00–0.00 | * |  |
|  | (d) Long-term care beds |  |  |  |  |  |  |  |
|  | January | 115 | 0–12 | 0.00–10.64 | 0–0 | 0.00–0.00 |  |  |
|  | February | 105 | 0–2 | 0.00–1.47 | 0–0 | 0.00–0.00 |  |  |
|  | March | 114 | 0–13 | 0.00–12.81 | 0–0 | 0.00–0.00 |  |  |
|  | April | 107 | 0–5 | 0.00–4.37 | 0–0 | 0.00–0.00 |  |  |
|  | May | 115 | 0–13 | 0.00–11.69 | 0–0 | 0.00–0.00 |  |  |
|  | June | 108 | 0–5 | 0.00–4.09 | 0–0 | 0.00–0.00 |  |  |
|  | (e) General beds |  |  |  |  |  |  |  |
|  | January | 20 | 0–1 | 0.00–1.54 | 0–0 | 0.00–0.00 |  |  |
|  | February | 20 | 0–0 | 0.00–0.00 | 0–1 | 0.00–0.64 |  |  |
|  | March | 20 | 0–1 | 0.00–0.26 | 0–0 | 0.00–0.00 |  |  |
|  | April | 20 | 0–0 | 0.00–0.00 | 0–1 | 0.00–0.33 |  |  |
|  | May | 21 | 0–2 | 0.00–7.83 | 0–0 | 0.00–0.00 |  |  |
|  | June | 19 | 0–0 | 0.00–0.00 | 0–1 | 0.00–2.48 |  |  |
|  | (f) LTCI care beds |  |  |  |  |  |  |  |
|  | January | 363 | 0–0 | 0.00–0.00 | 0–9 | 0.00–2.27 |  |  |
|  | February | 515 | 48–158 | 13.34–44.17 | 0–0 | 0.00–0.00 | * |  |
|  | March | 486 | 18–130 | 4.83–36.49 | 0–0 | 0.00–0.00 | * |  |
|  | April | 232 | 0–0 | 0.00–0.00 | 55–160 | 13.83–40.71 |  | ** |
|  | May | 413 | 0–1 | 0.00–0.08 | 0–0 | 0.00–0.00 |  |  |
|  | June | 399 | 0–0 | 0.00–0.00 | 0–2 | 0.00–0.45 |  |  |
| Okinawa | (1) Average number of outpatients per day at hospitals |  |  |  |  |  |  |  |
|  | General hospitals |  |  |  |  |  |  |  |
|  | January | 12697 | 0–0 | 0.00–0.00 | 0–658 | 0.00–4.92 |  |  |
|  | February | 12570 | 0–0 | 0.00–0.00 | 92–905 | 0.68–6.72 |  | ** |
|  | March | 12249 | 0–0 | 0.00–0.00 | 497–1292 | 3.67–9.54 |  | ** |
|  | April | 10220 | 0–0 | 0.00–0.00 | 2188–2998 | 16.55–22.68 |  | ** |
|  | May | 8878 | 0–0 | 0.00–0.00 | 3608–4418 | 27.14–33.22 |  | ** |
|  | June | 11722 | 0–0 | 0.00–0.00 | 830–1626 | 6.22–12.18 |  | ** |
|  | Psychiatric hospitals |  |  |  |  |  |  |  |
|  | January | 1188 | 0–0 | 0.00–0.00 | 0–34 | 0.00–2.73 |  |  |
|  | February | 1201 | 0–0 | 0.00–0.00 | 0–43 | 0.00–3.41 |  |  |
|  | March | 1257 | 0–0 | 0.00–0.00 | 0–24 | 0.00–1.80 |  |  |
|  | April | 1140 | 0–0 | 0.00–0.00 | 13–116 | 1.04–9.22 |  | ** |
|  | May | 912 | 0–0 | 0.00–0.00 | 249–360 | 19.58–28.30 |  | ** |
|  | June | 1237 | 0–0 | 0.00–0.00 | 0–38 | 0.00–2.93 |  |  |
|  | (2) Average number of hospitalized patients per day |  |  |  |  |  |  |  |
|  | (b) Psychiatric care beds |  |  |  |  |  |  |  |
|  | January | 4705 | 0–7 | 0.00–0.13 | 0–0 | 0.00–0.00 |  |  |
|  | February | 4689 | 0–0 | 0.00–0.00 | 0–12 | 0.00–0.25 |  |  |
|  | March | 4682 | 0–0 | 0.00–0.00 | 0–25 | 0.00–0.53 |  |  |
|  | April | 4650 | 0–0 | 0.00–0.00 | 0–64 | 0.00–1.34 |  |  |
|  | May | 4676 | 0–0 | 0.00–0.00 | 0–48 | 0.00–1.01 |  |  |
|  | June | 4696 | 0–0 | 0.00–0.00 | 0–34 | 0.00–0.71 |  |  |
|  | (c) Tuberculosis care beds |  |  |  |  |  |  |  |
|  | January | 13 | 0–0 | 0.00–0.00 | 0–2 | 0.00–11.81 |  |  |
|  | February | 21 | 0–8 | 0.00–56.42 | 0–0 | 0.00–0.00 |  |  |
|  | March | 17 | 0–4 | 0.00–30.63 | 0–0 | 0.00–0.00 |  |  |
|  | April | 15 | 0–3 | 0.00–17.83 | 0–0 | 0.00–0.00 |  |  |
|  | May | 16 | 0–5 | 0.00–34.01 | 0–0 | 0.00–0.00 |  |  |
|  | June | 16 | 0–5 | 0.00–43.45 | 0–0 | 0.00–0.00 |  |  |
|  | (d) Long-term care beds |  |  |  |  |  |  |  |
|  | January | 3511 | 0–44 | 0.00–1.24 | 0–0 | 0.00–0.00 |  |  |
|  | February | 3539 | 0–61 | 0.00–1.75 | 0–0 | 0.00–0.00 |  |  |
|  | March | 3537 | 0–47 | 0.00–1.34 | 0–0 | 0.00–0.00 |  |  |
|  | April | 3487 | 0–4 | 0.00–0.11 | 0–0 | 0.00–0.00 |  |  |
|  | May | 3432 | 0–0 | 0.00–0.00 | 0–48 | 0.00–1.35 |  |  |
|  | June | 3492 | 0–18 | 0.00–0.50 | 0–0 | 0.00–0.00 |  |  |
|  | (e) General beds |  |  |  |  |  |  |  |
|  | January | 7788 | 0–0 | 0.00–0.00 | 0–156 | 0.00–1.96 |  |  |
|  | February | 7986 | 0–0 | 0.00–0.00 | 0–93 | 0.00–1.14 |  |  |
|  | March | 7705 | 0–0 | 0.00–0.00 | 150–371 | 1.86–4.58 |  | ** |
|  | April | 7061 | 0–0 | 0.00–0.00 | 686–914 | 8.60–11.45 |  | ** |
|  | May | 6746 | 0–0 | 0.00–0.00 | 1036–1226 | 13.00–15.37 |  | ** |
|  | June | 7334 | 0–0 | 0.00–0.00 | 470–687 | 5.86–8.56 |  | ** |
|  | (f) LTCI care beds |  |  |  |  |  |  |  |
|  | January | 239 | 0–5 | 0.00–1.88 | 0–0 | 0.00–0.00 |  |  |
|  | February | 243 | 0–6 | 0.00–2.51 | 0–0 | 0.00–0.00 |  |  |
|  | March | 245 | 0–3 | 0.00–1.21 | 0–0 | 0.00–0.00 |  |  |
|  | April | 198 | 0–0 | 0.00–0.00 | 13–43 | 5.40–17.78 |  | ** |
|  | May | 197 | 0–0 | 0.00–0.00 | 14–44 | 5.81–18.23 |  | ** |
|  | June | 193 | 0–0 | 0.00–0.00 | 20–50 | 8.26–20.31 |  | ** |
|  | (3) Average length of hospital stays per patient |  |  |  |  |  |  |  |
|  | (b) Psychiatric care beds |  |  |  |  |  |  |  |
|  | January | 239 | 0–4 | 0.00–1.40 | 0–0 | 0.00–0.00 |  |  |
|  | February | 250 | 0–20 | 0.00–8.23 | 0–0 | 0.00–0.00 |  |  |
|  | March | 234 | 0–7 | 0.00–2.85 | 0–0 | 0.00–0.00 |  |  |
|  | April | 246 | 0–16 | 0.00–6.88 | 0–0 | 0.00–0.00 |  |  |
|  | May | 276 | 19–49 | 8.08–21.38 | 0–0 | 0.00–0.00 | * |  |
|  | June | 222 | 0–0 | 0.00–0.00 | 0–4 | 0.00–1.43 |  |  |
|  | (c) Tuberculosis care beds |  |  |  |  |  |  |  |
|  | January | 59 | 0–4 | 0.00–6.81 | 0–0 | 0.00–0.00 |  |  |
|  | February | 82 | 0–34 | 0.00–69.96 | 0–0 | 0.00–0.00 |  |  |
|  | March | 45 | 0–0 | 0.00–0.00 | 0–2 | 0.00–2.86 |  |  |
|  | April | 44 | 0–0 | 0.00–0.00 | 0–3 | 0.00–4.74 |  |  |
|  | May | 57 | 0–17 | 0.00–39.22 | 0–0 | 0.00–0.00 |  |  |
|  | June | 61 | 0–20 | 0.00–45.24 | 0–0 | 0.00–0.00 |  |  |
|  | (d) Long-term care beds |  |  |  |  |  |  |  |
|  | January | 144 | 0–5 | 0.00–3.02 | 0–0 | 0.00–0.00 |  |  |
|  | February | 144 | 0–7 | 0.00–4.62 | 0–0 | 0.00–0.00 |  |  |
|  | March | 132 | 0–0 | 0.00–0.00 | 0–2 | 0.00–0.80 |  |  |
|  | April | 147 | 0–12 | 0.00–8.72 | 0–0 | 0.00–0.00 |  |  |
|  | May | 161 | 2–26 | 1.33–18.75 | 0–0 | 0.00–0.00 | * |  |
|  | June | 145 | 0–11 | 0.00–7.58 | 0–0 | 0.00–0.00 |  |  |
|  | (e) General beds |  |  |  |  |  |  |  |
|  | January | 16 | 0–1 | 0.00–2.95 | 0–0 | 0.00–0.00 |  |  |
|  | February | 16 | 0–1 | 0.00–2.80 | 0–0 | 0.00–0.00 |  |  |
|  | March | 15 | 0–1 | 0.00–3.11 | 0–0 | 0.00–0.00 |  |  |
|  | April | 16 | 0–2 | 0.00–10.54 | 0–0 | 0.00–0.00 |  |  |
|  | May | 18 | 0–4 | 0.00–21.29 | 0–0 | 0.00–0.00 |  |  |
|  | June | 15 | 0–1 | 0.00–2.93 | 0–0 | 0.00–0.00 |  |  |
|  | (f) LTCI care beds |  |  |  |  |  |  |  |
|  | January | 412 | 0–0 | 0.00–0.00 | 0–148 | 0.00–26.36 |  |  |
|  | February | 503 | 0–0 | 0.00–0.00 | 0–40 | 0.00–7.30 |  |  |
|  | March | 178 | 0–0 | 0.00–0.00 | 228–402 | 39.22–69.26 |  | ** |
|  | April | 792 | 0–191 | 0.00–31.75 | 0–0 | 0.00–0.00 |  |  |
|  | May | 643 | 0–5 | 0.00–0.72 | 0–0 | 0.00–0.00 |  |  |
|  | June | 483 | 0–0 | 0.00–0.00 | 0–133 | 0.00–21.48 |  |  |

LTCI care beds: long-term care beds covered by the long-term care insurance. * indicates a month where the observed value exceeded the upper bound of the two-sided 95% prediction interval; ** indicates a month where the observed value falls the lower bound of the two-sided 95% prediction interval.
