# Supplementary material for: Trends in Healthcare Access in Japan during the First Wave of the COVID-19 Pandemic, up to June 2020
Source: Int J Environ Res Public Health. 2021 Mar 22;18(6):3271. doi: 10.3390/ijerph18063271 (PMC8004161; doi:10.3390/ijerph18063271)

# Hokkaido

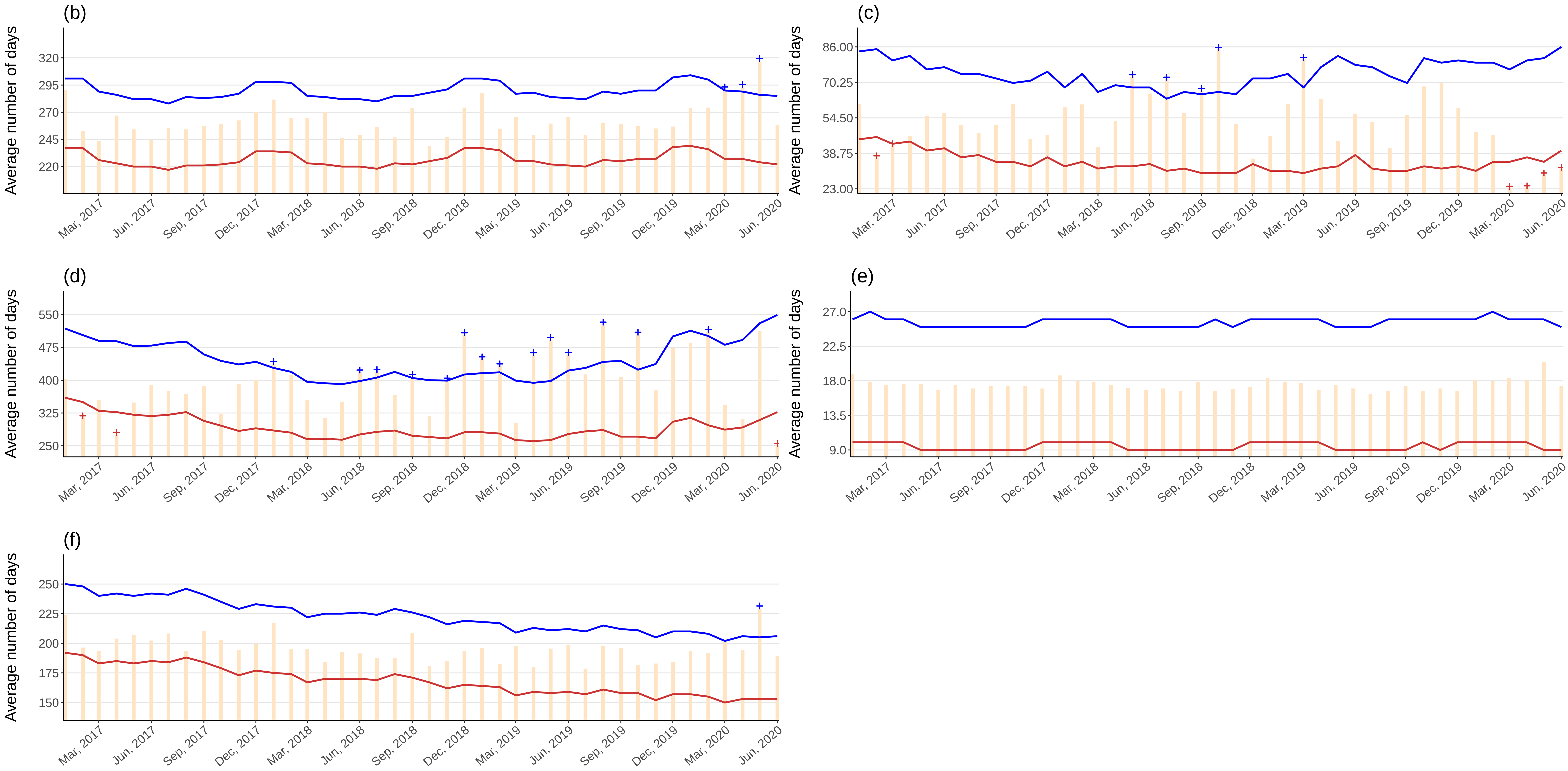


# Aomori

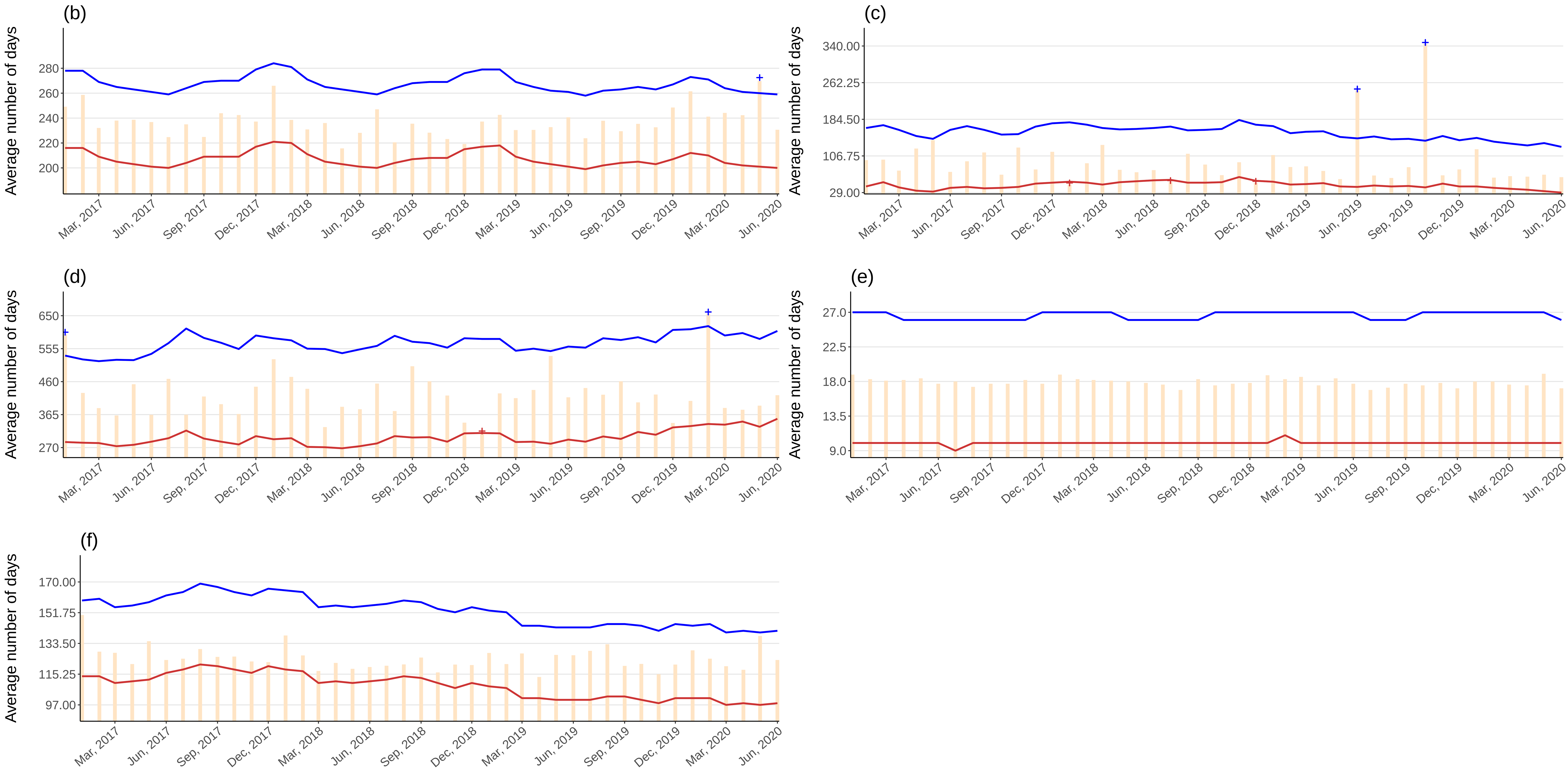


# Iwate

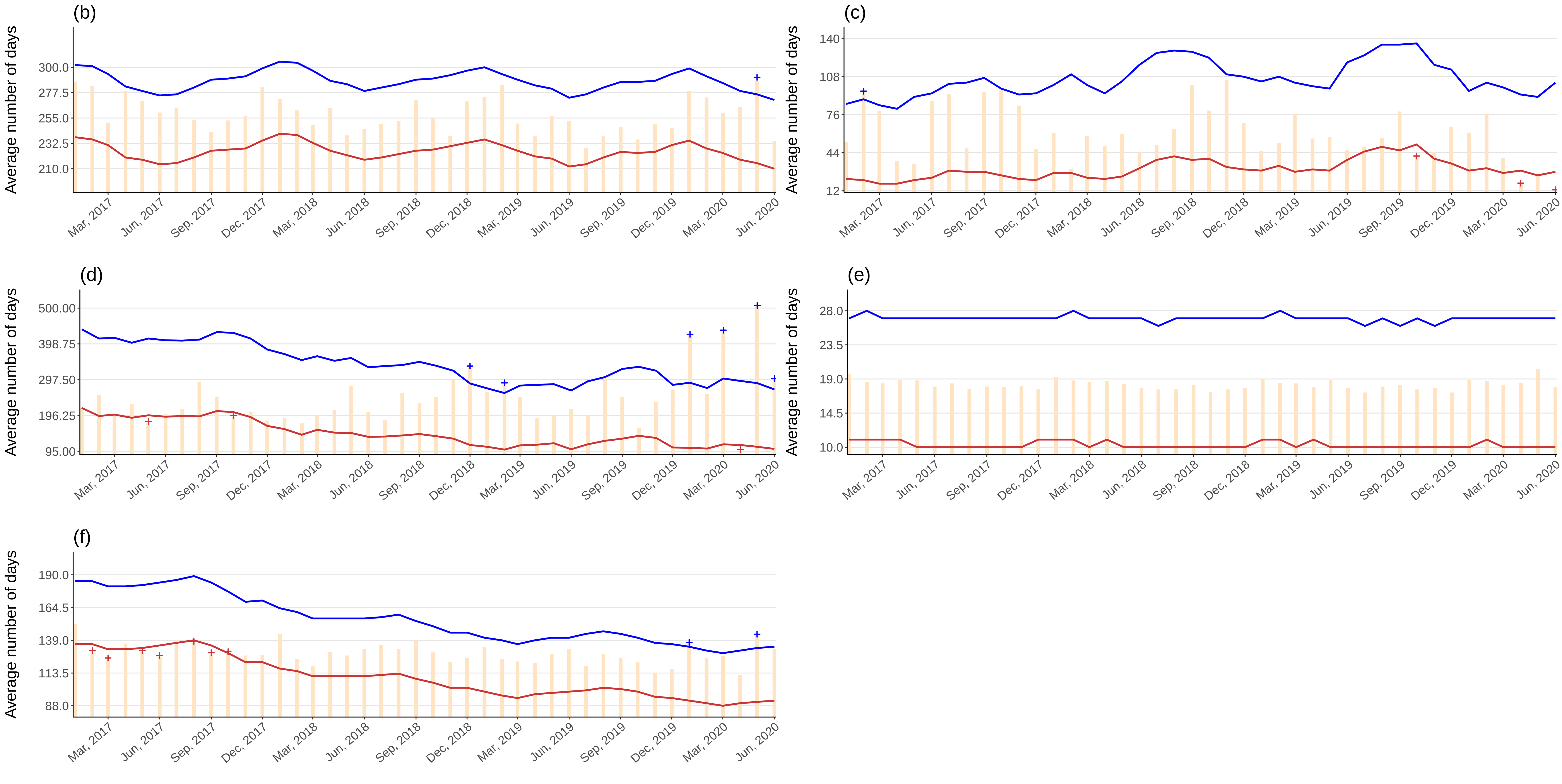

## Miyagi

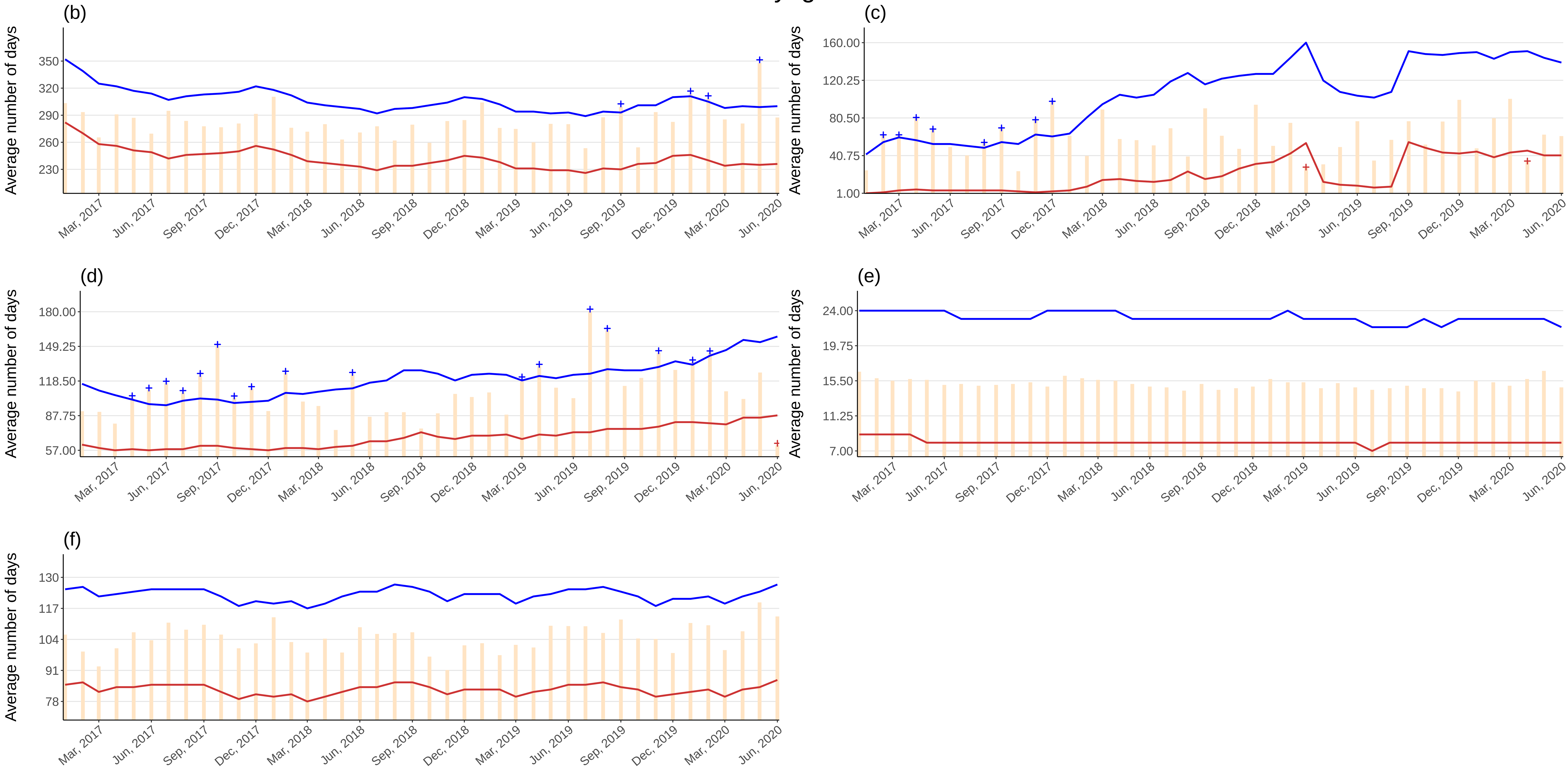


## Akita

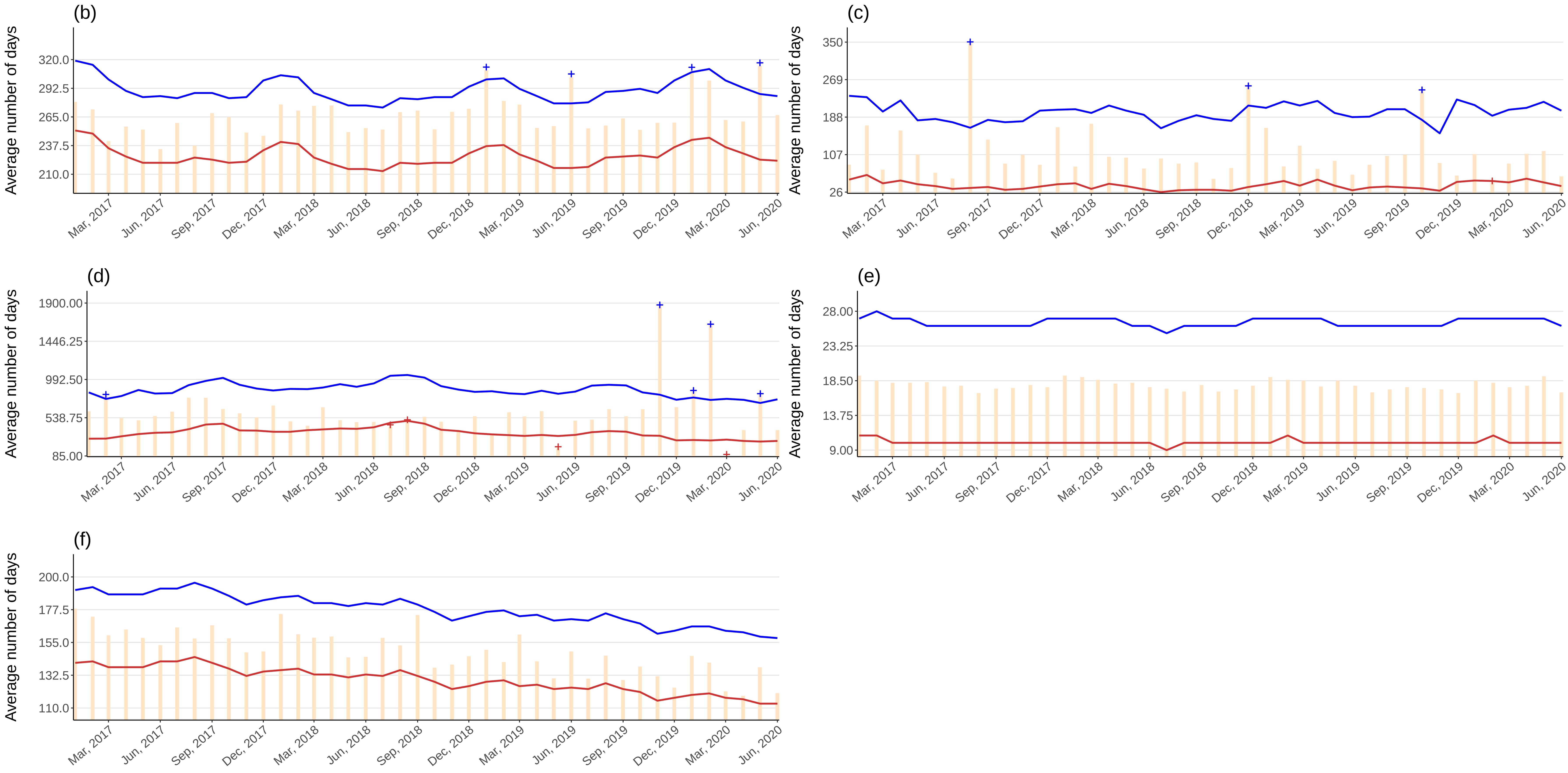


## Yamagata

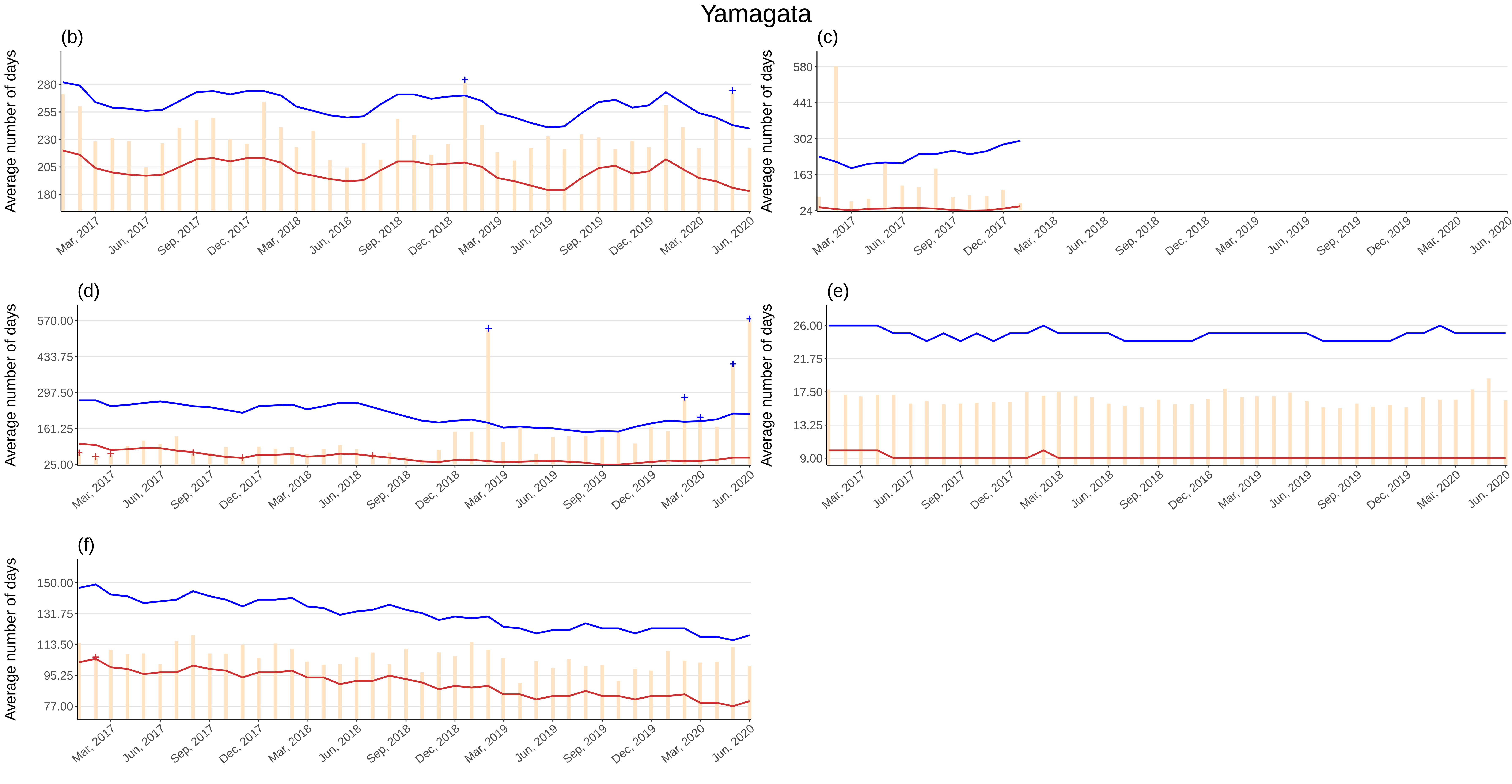

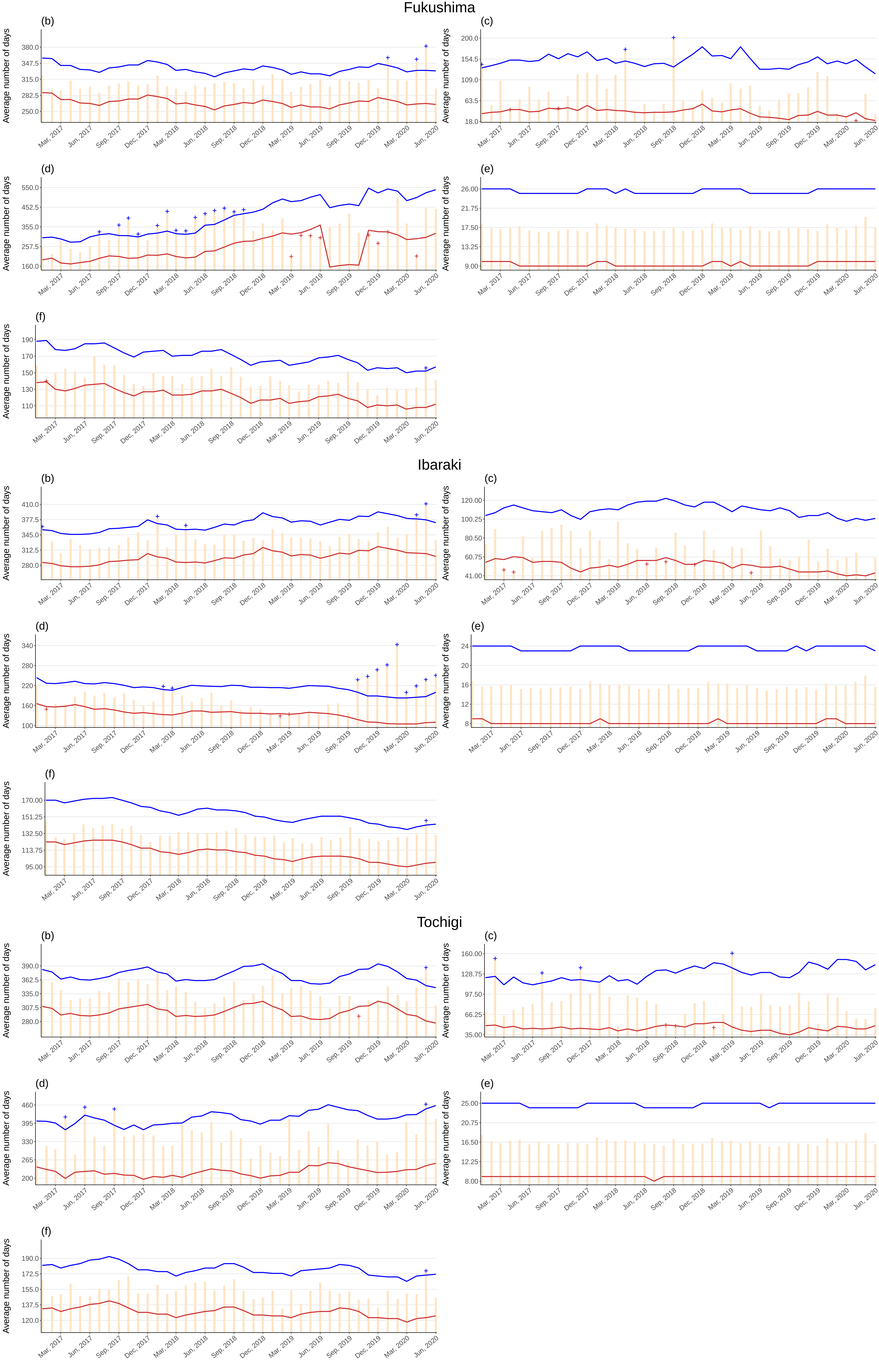
Fukushima
(b)
Average number of days
380.0
347.5
315.0
282.5
250.0
(c)
200.0
154.5
109.0
63.5
18.0
(d)
550.0
452.5
355.0
257.5
160.0
(e)
26.00
21.75
17.50
13.25
9.00
(f)
190
170
150
130
110
Mar, 2017
Jun, 2017
Sep, 2017
Dec, 2017
Mar, 2018
Jun, 2018
Sep, 2018
Dec, 2018
Mar, 2019
Jun, 2019
Sep, 2019
Dec, 2019
Mar, 2020
Jun, 2020
Ibaraki
(b)
410.0
377.5
345.0
312.5
280.0
(c)
120.00
100.25
80.50
60.75
41.00
(d)
340
280
220
160
100
(e)
24
20
16
12
8
(f)
170.00
151.25
132.50
113.75
95.00
Tochigi
(b)
390.0
362.5
335.0
307.5
280.0
(c)
160.00
128.75
97.50
66.25
35.00
(d)
460
395
330
265
200
(e)
25.00
20.75
16.50
12.25
8.00
(f)
190.0
172.5
155.0
137.5
120.0

# Gunma

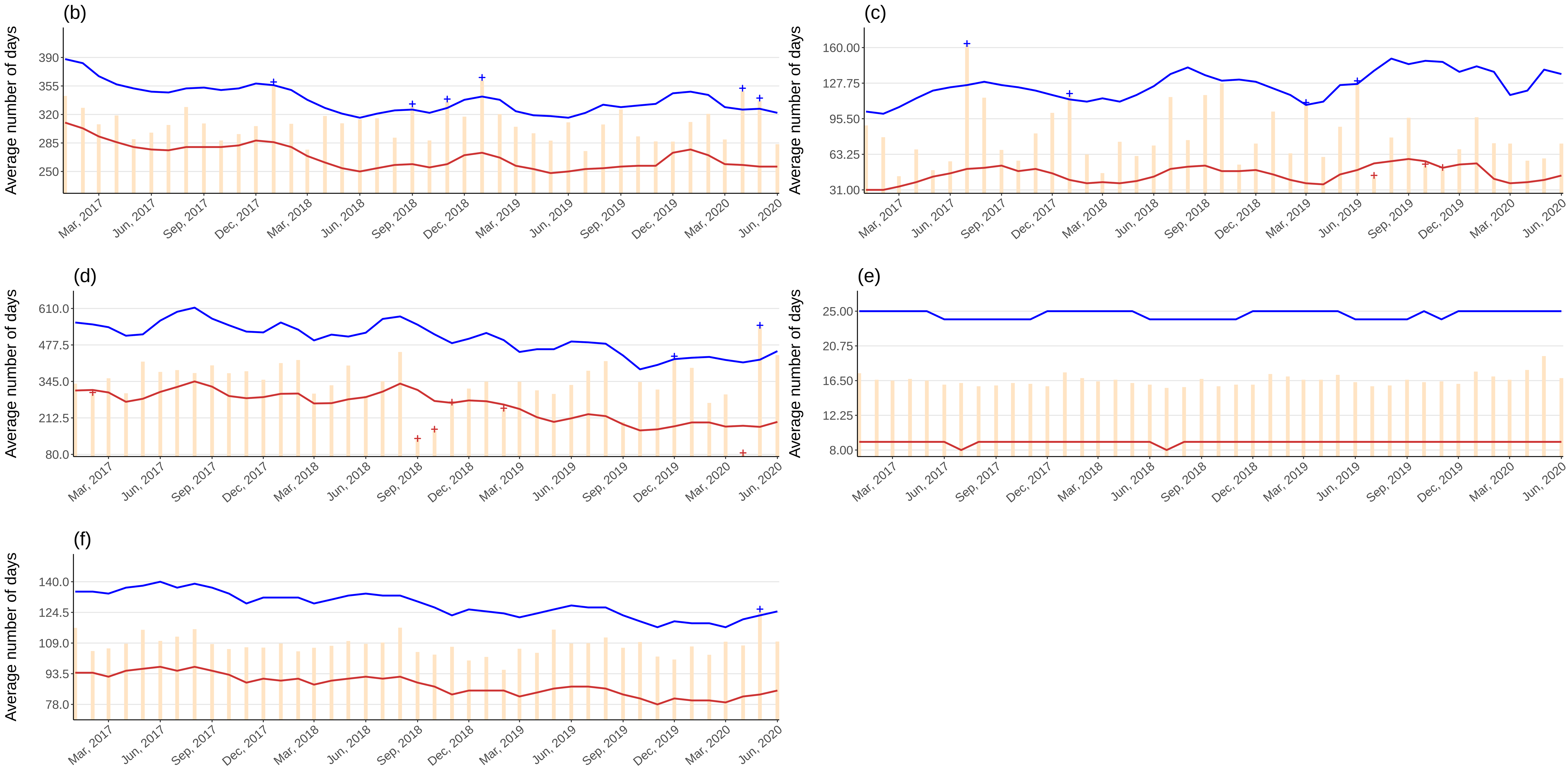


# Saitama

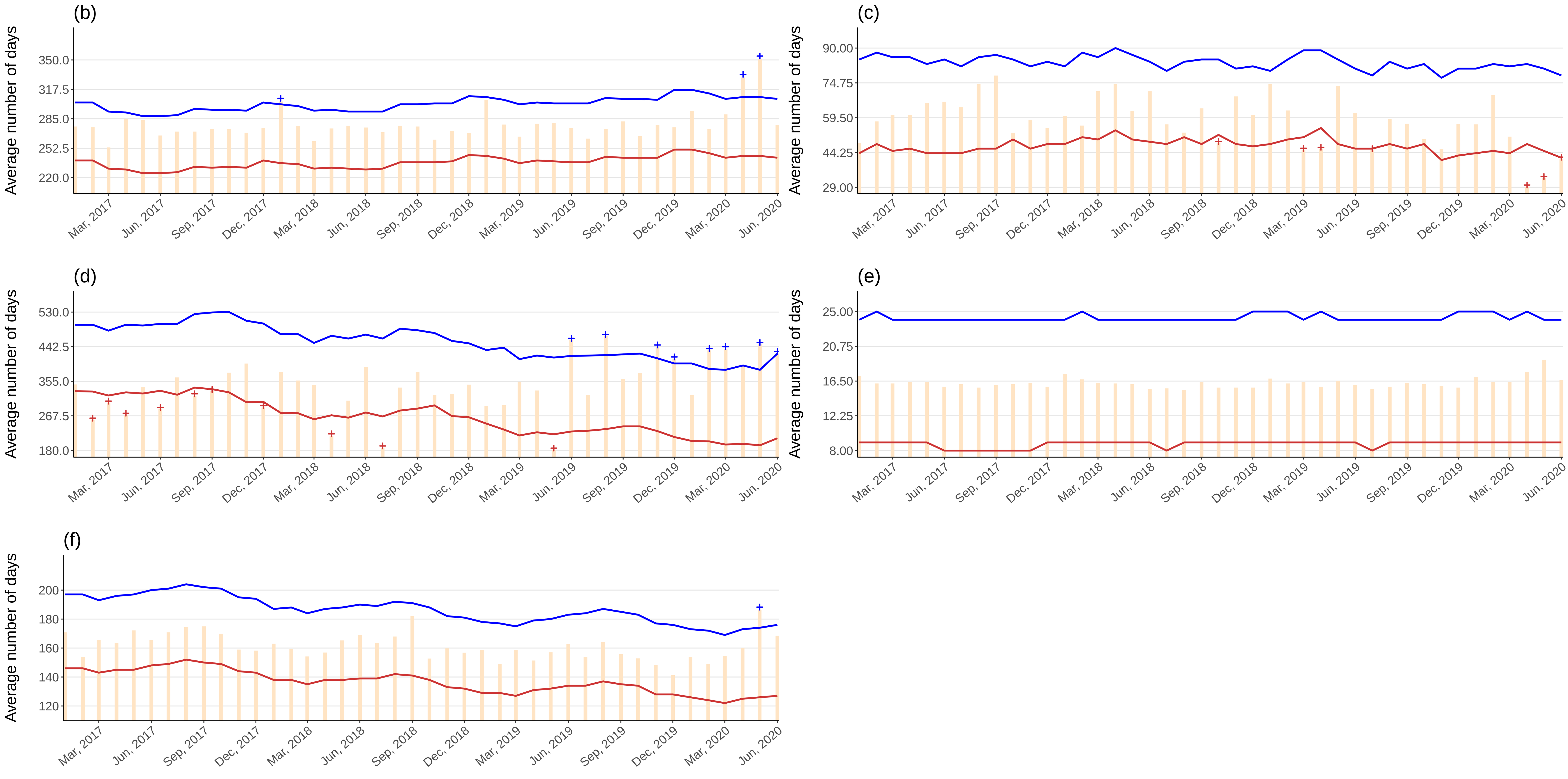


# Chiba

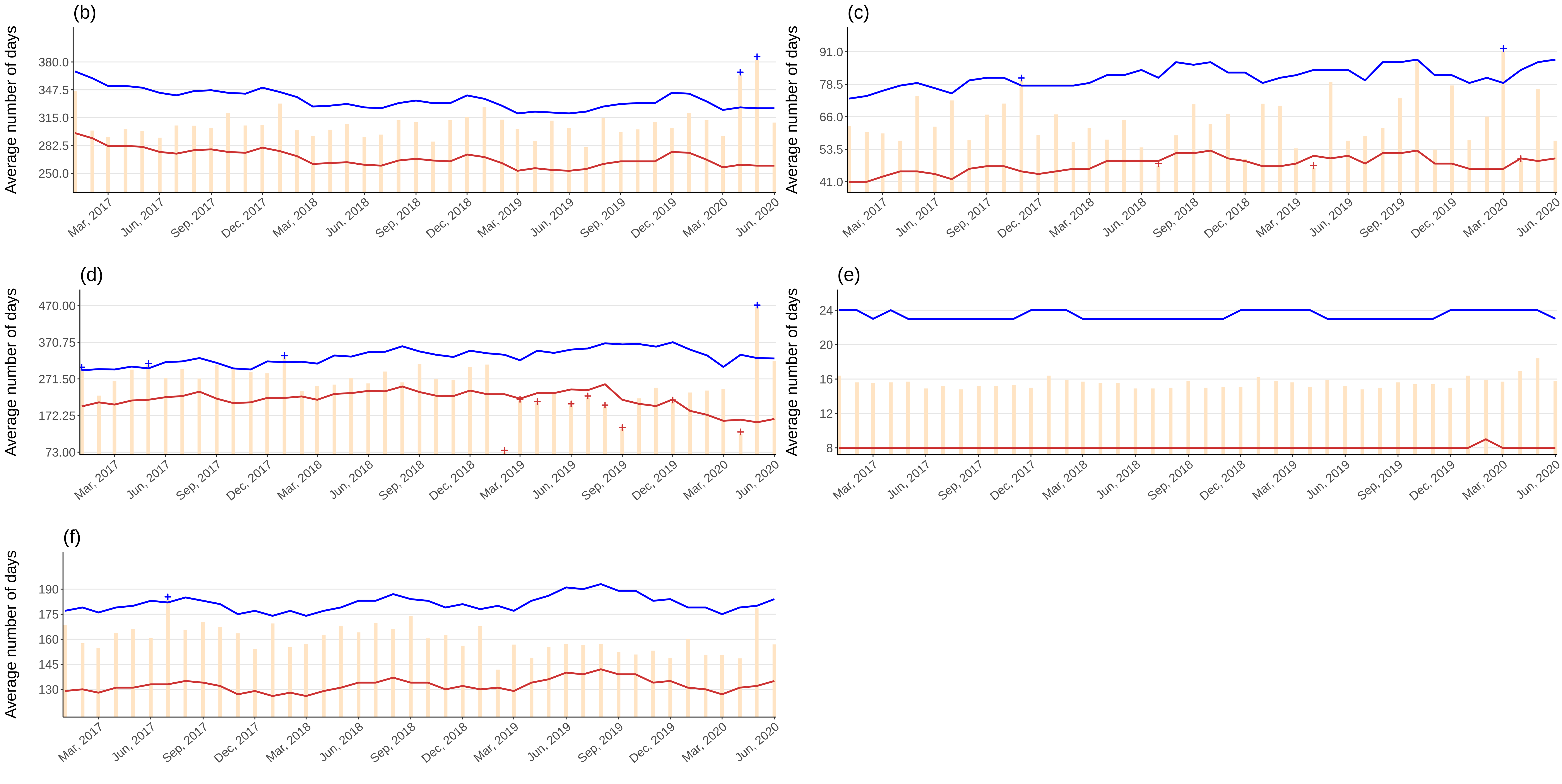

# Tokyo

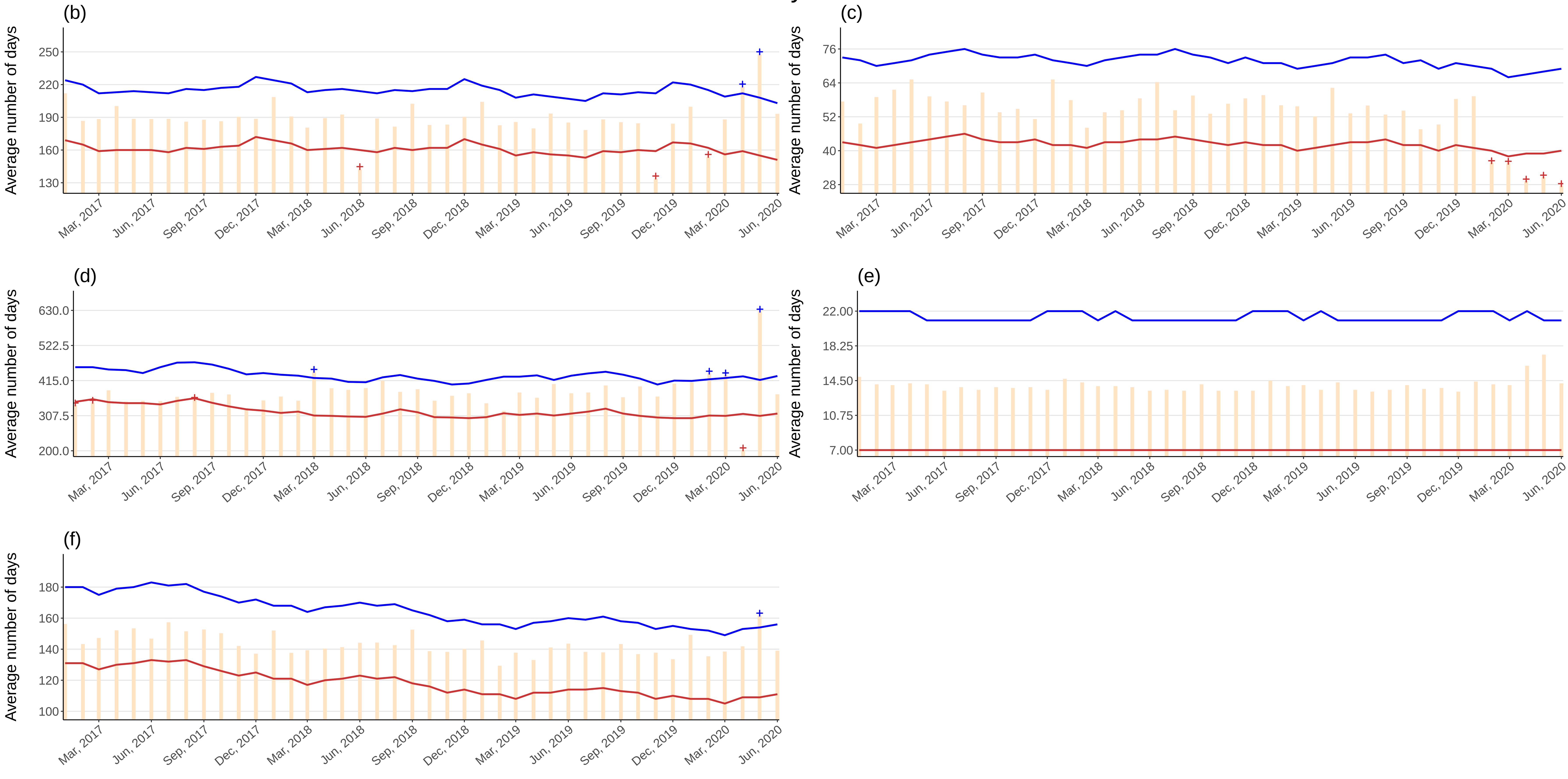


# Kanagawa

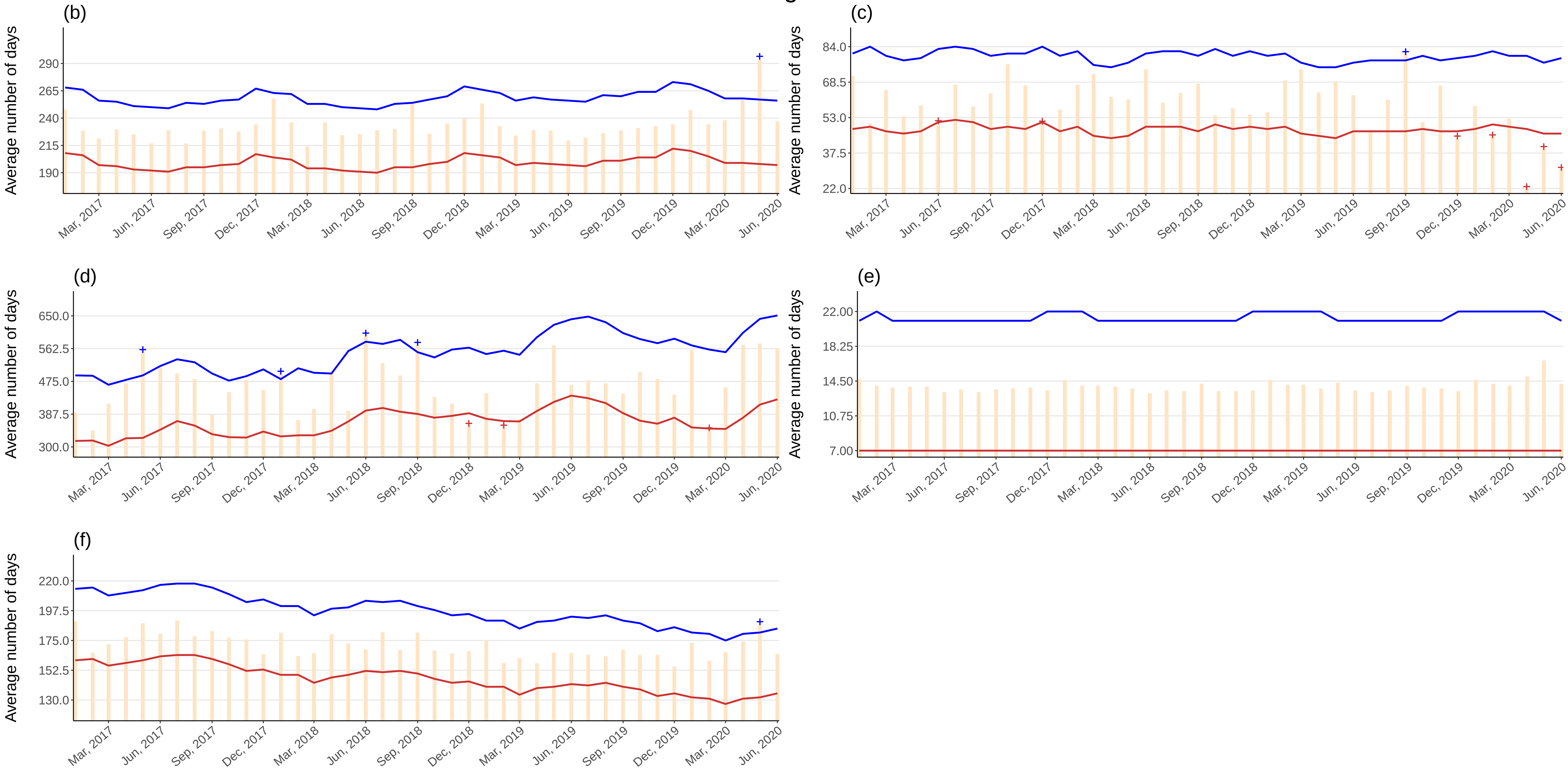


# Niigata

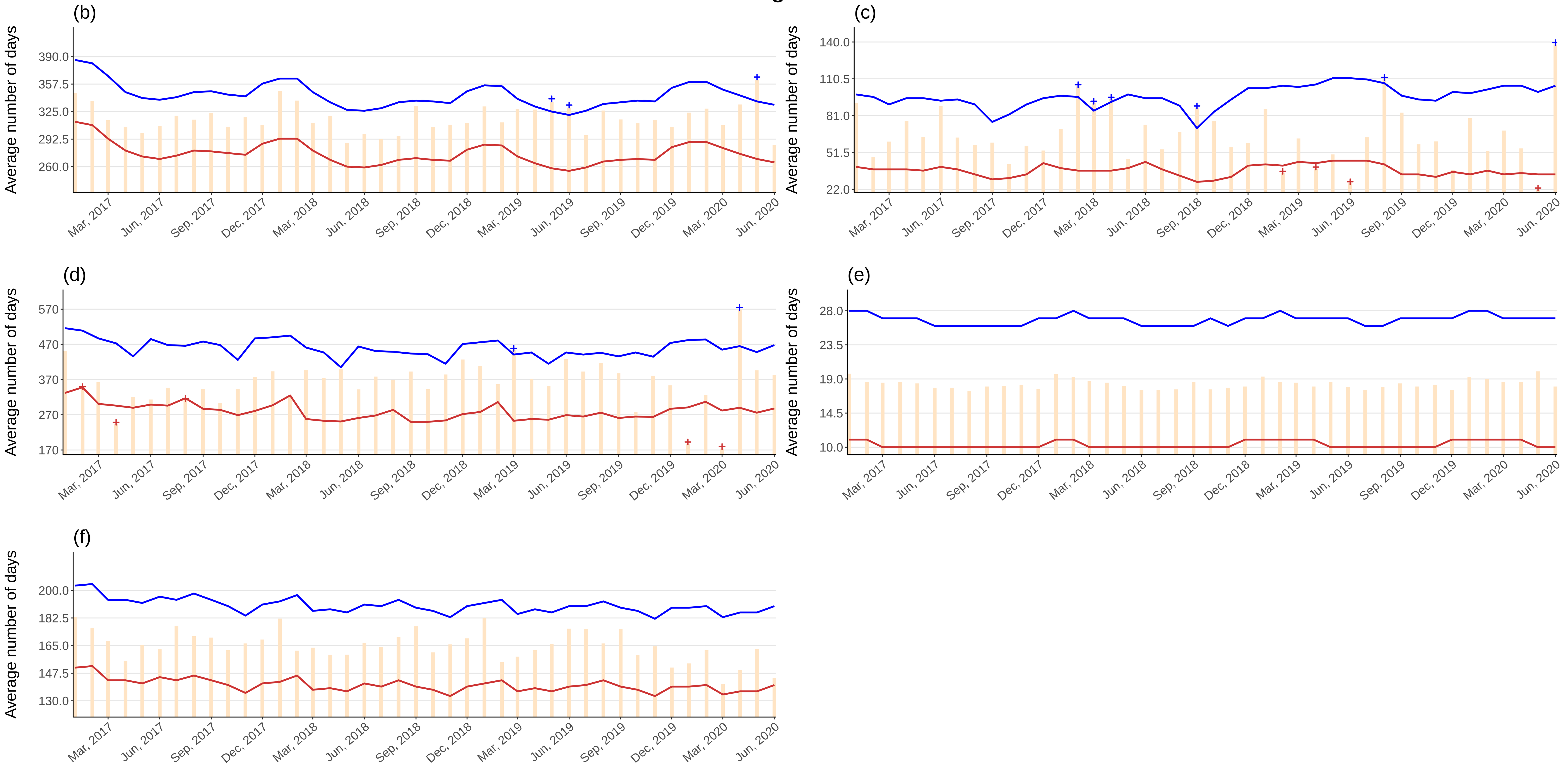

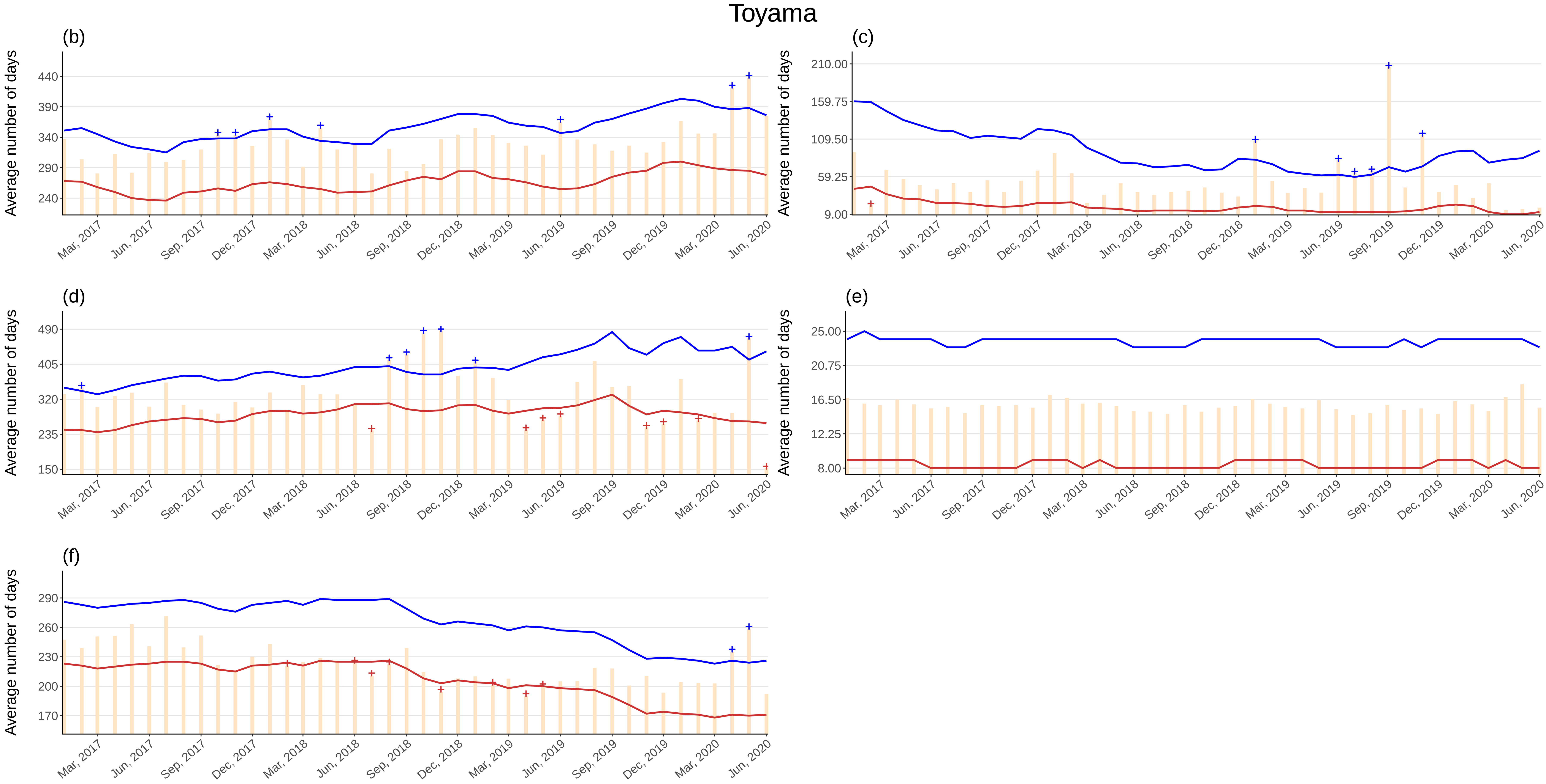

Toyama
(b)
(c)
(d)
(e)
(f)
Average number of days
Mar, 2017
Jun, 2017
Sep, 2017
Dec, 2017
Mar, 2018
Jun, 2018
Sep, 2018
Dec, 2018
Mar, 2019
Jun, 2019
Sep, 2019
Dec, 2019
Mar, 2020
Jun, 2020


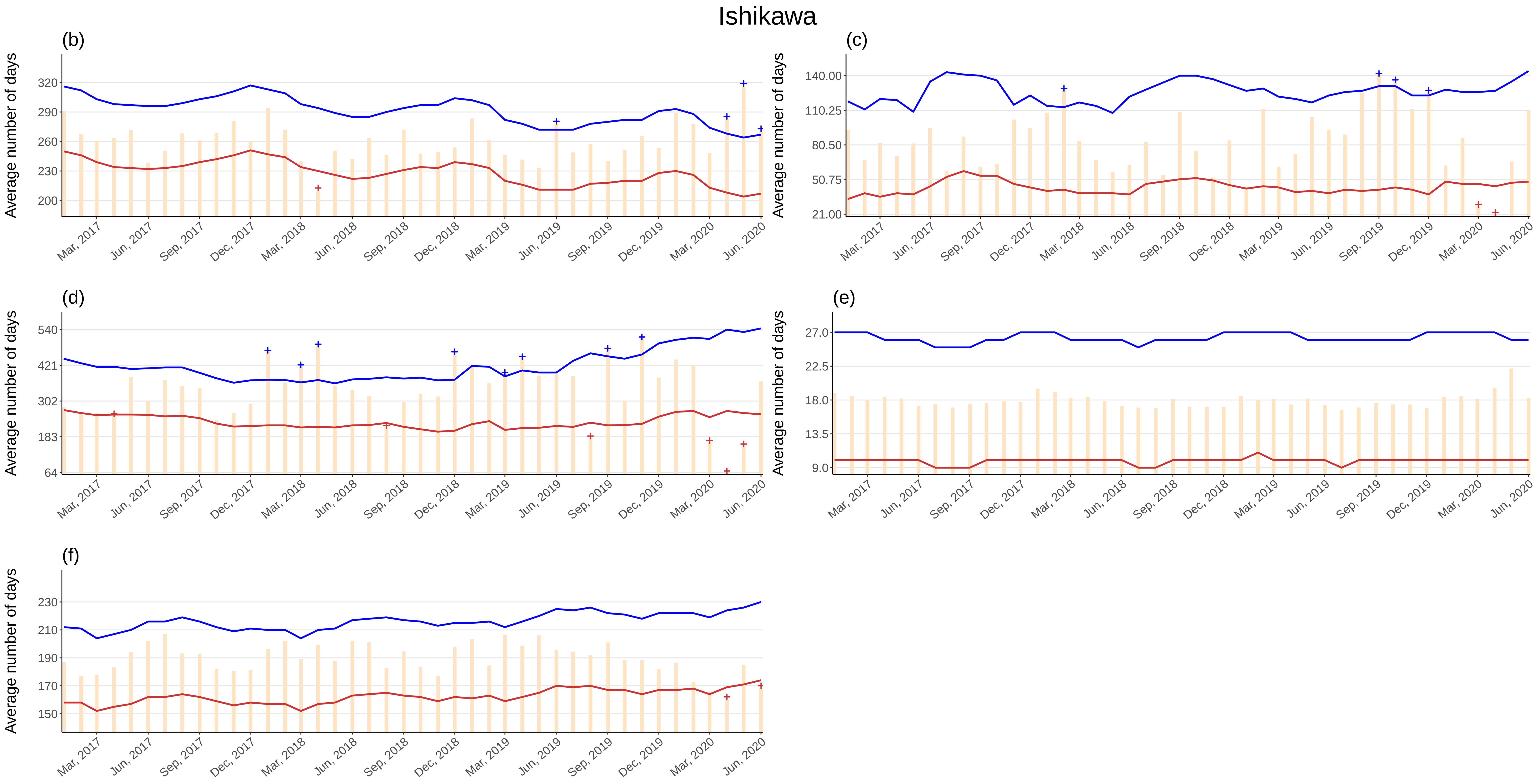

Ishikawa
(b)
(c)
(d)
(e)
(f)
Average number of days
Mar, 2017
Jun, 2017
Sep, 2017
Dec, 2017
Mar, 2018
Jun, 2018
Sep, 2018
Dec, 2018
Mar, 2019
Jun, 2019
Sep, 2019
Dec, 2019
Mar, 2020
Jun, 2020


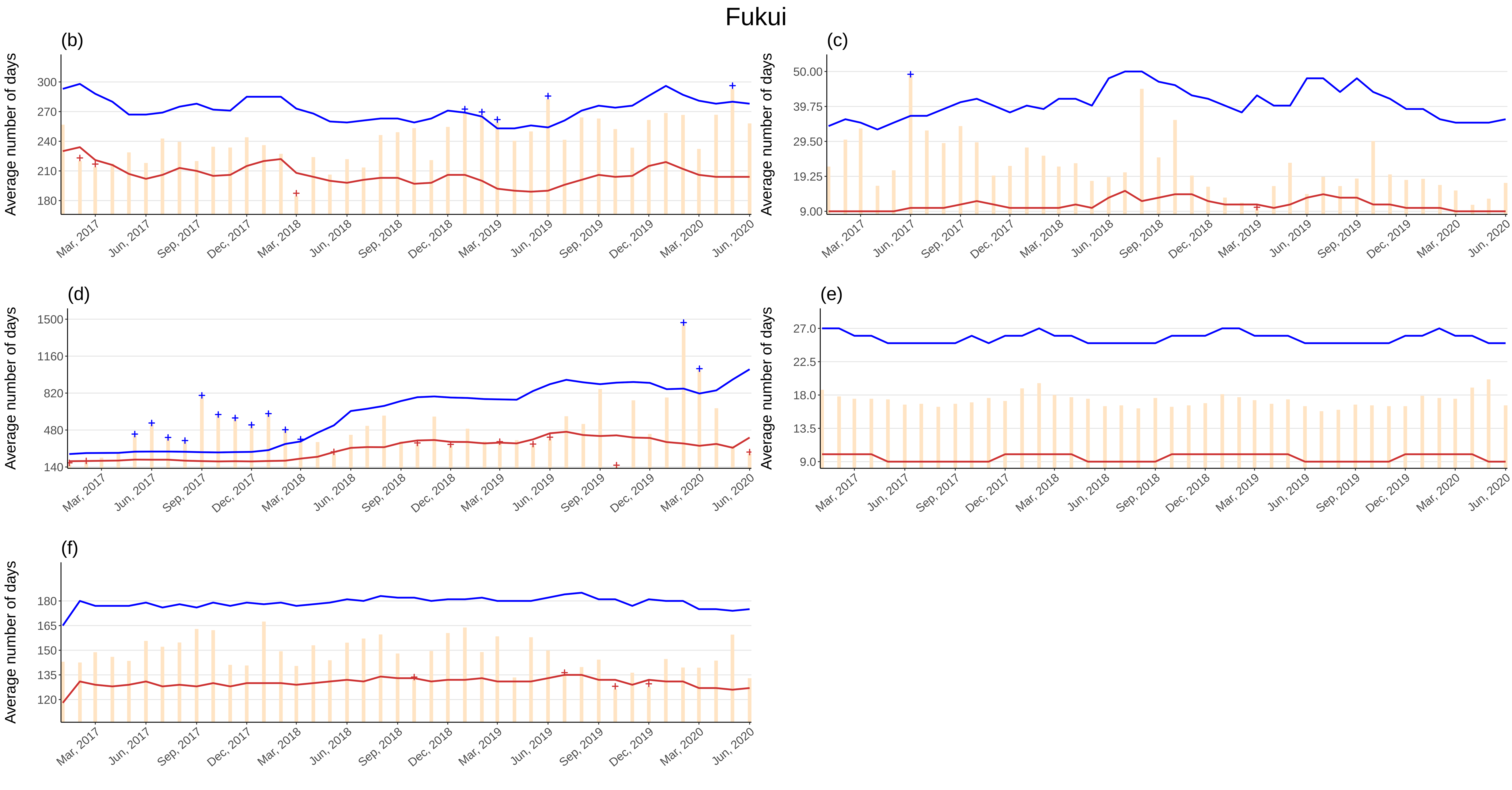

Fukui
(b)
(c)
(d)
(e)
(f)
Average number of days
Mar, 2017
Jun, 2017
Sep, 2017
Dec, 2017
Mar, 2018
Jun, 2018
Sep, 2018
Dec, 2018
Mar, 2019
Jun, 2019
Sep, 2019
Dec, 2019
Mar, 2020
Jun, 2020

## Yamanashi

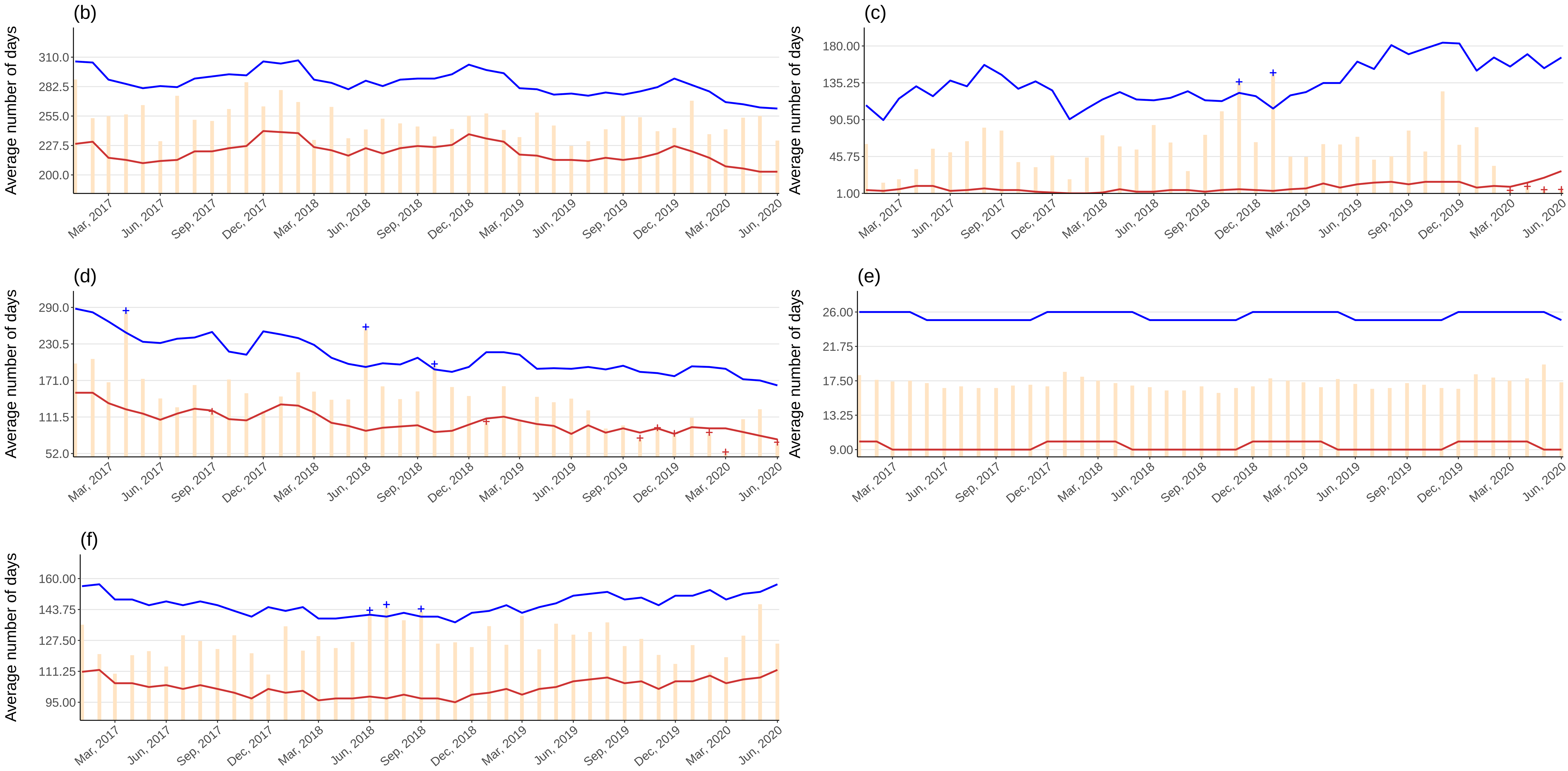


## Nagano

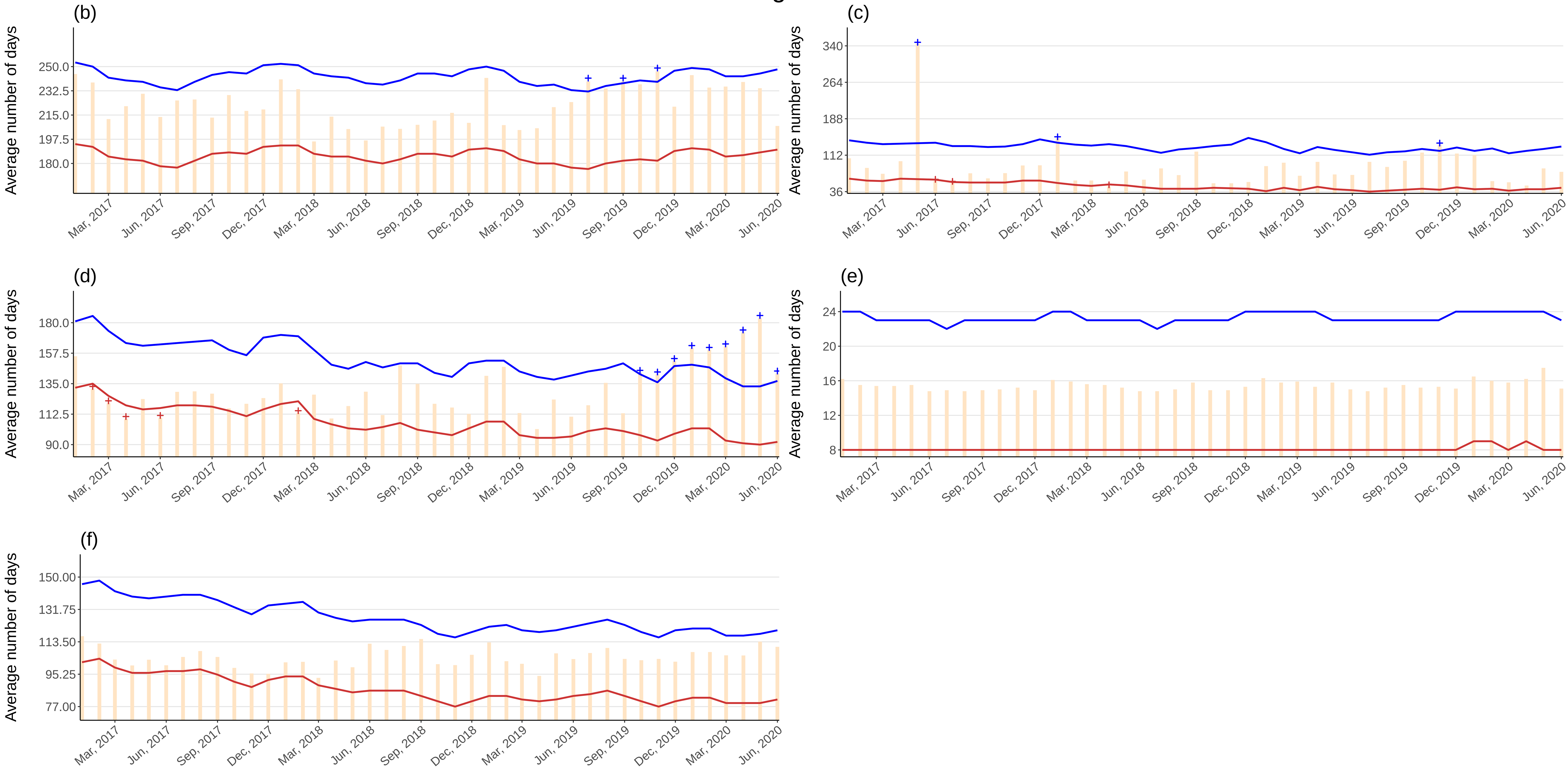


## Gifu

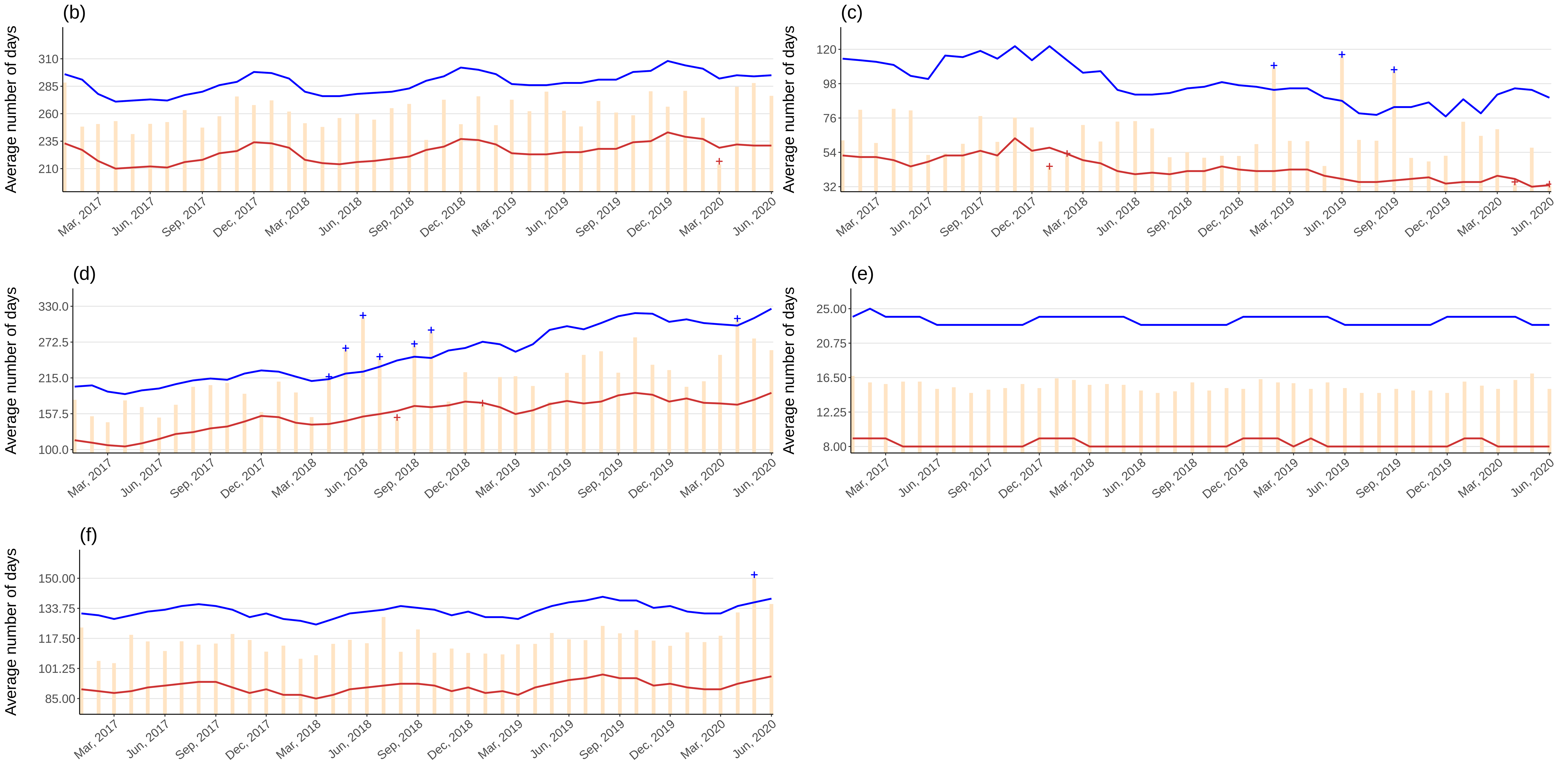

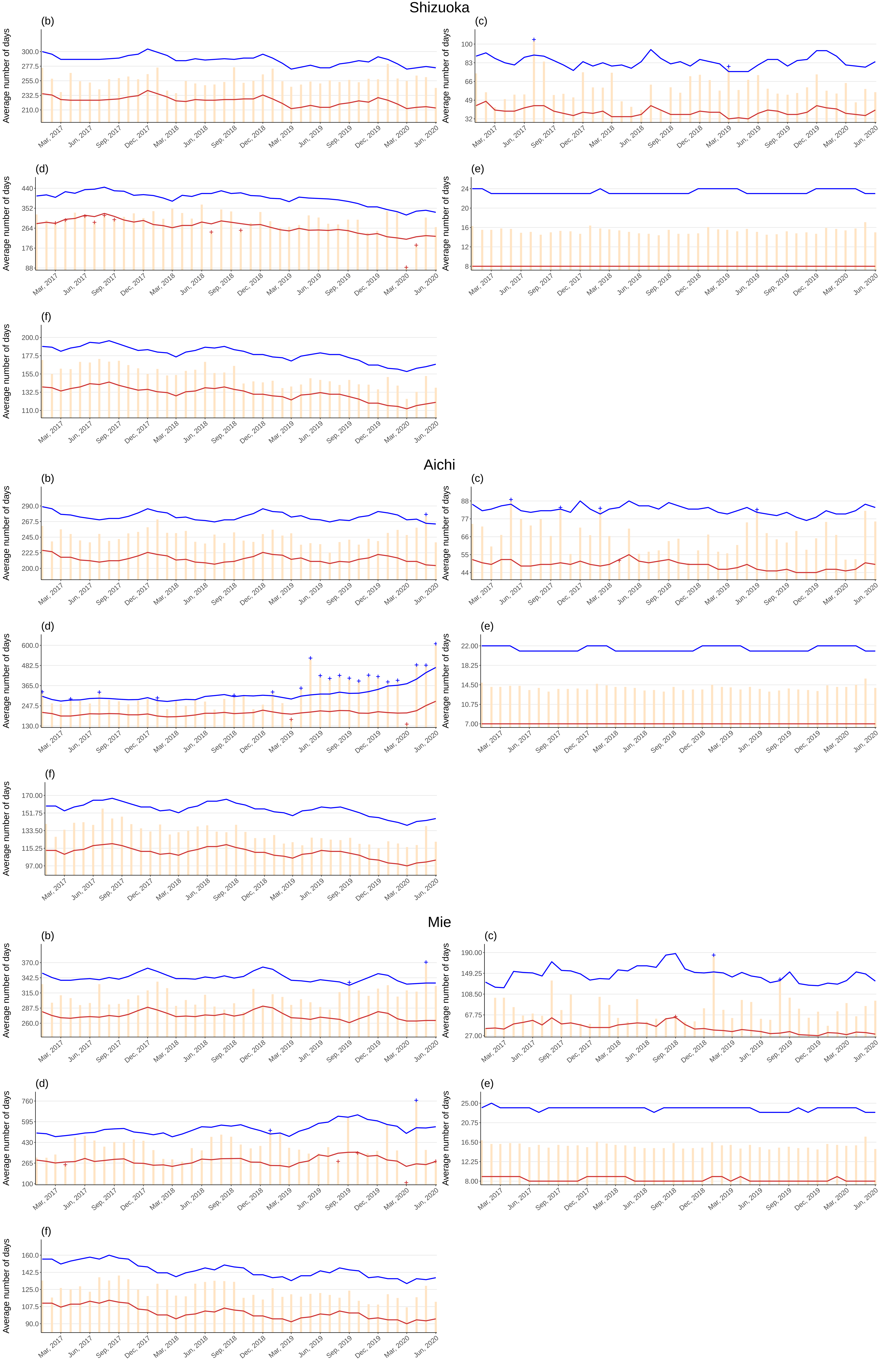

Shizuoka
(b)
(c)
(d)
(e)
(f)
Aichi
(b)
(c)
(d)
(e)
(f)
Mie
(b)
(c)
(d)
(e)
(f)
Average number of days
Mar, 2017
Jun, 2017
Sep, 2017
Dec, 2017
Mar, 2018
Jun, 2018
Sep, 2018
Dec, 2018
Mar, 2019
Jun, 2019
Sep, 2019
Dec, 2019
Mar, 2020
Jun, 2020

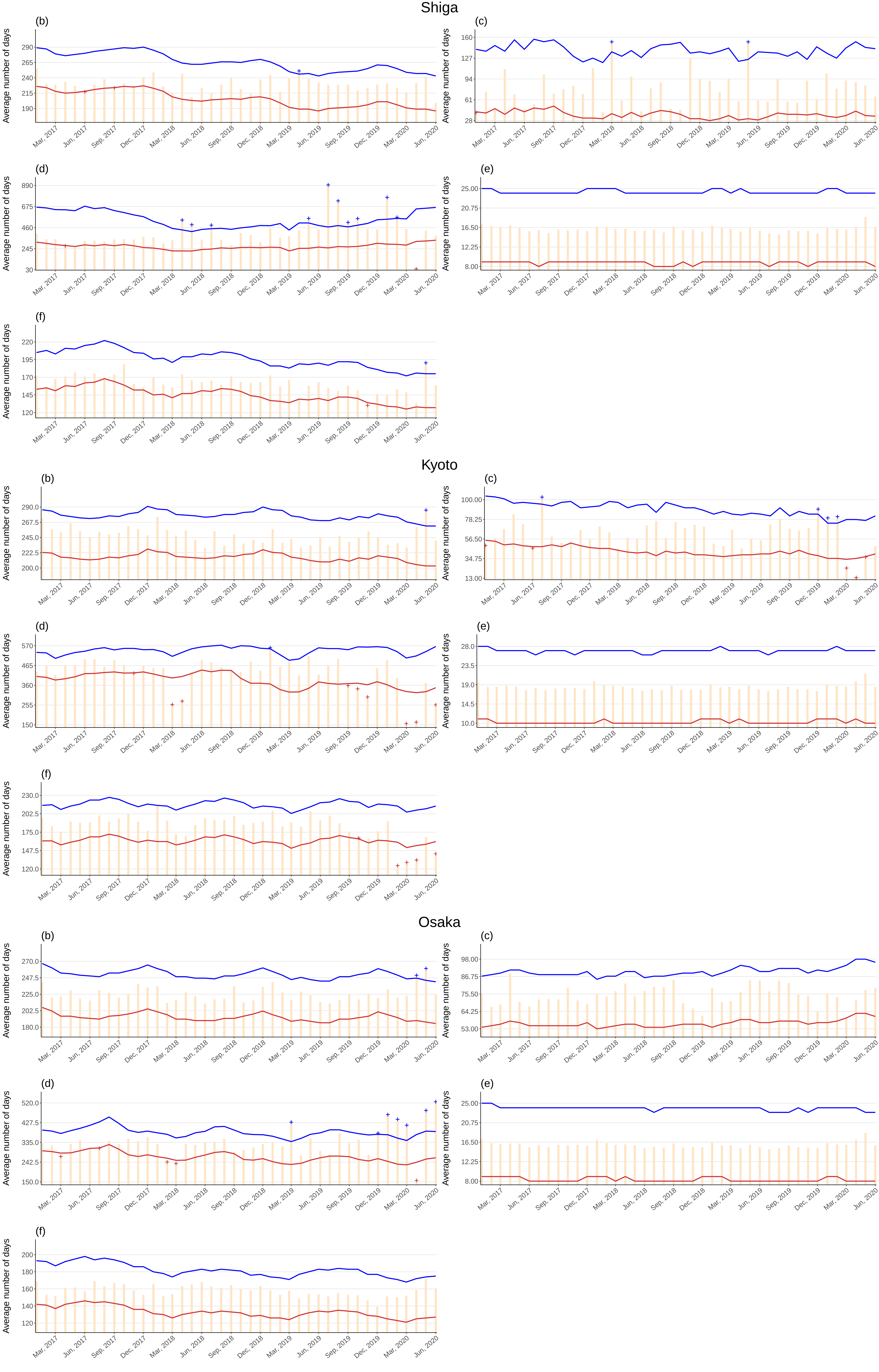

Shiga
(b)
Average number of days
190
215
240
265
290
(c)
Average number of days
28
61
94
127
160
(d)
Average number of days
30
245
460
675
890
(e)
Average number of days
8.00
12.25
16.50
20.75
25.00
(f)
Average number of days
120
145
170
195
220
Kyoto
(b)
Average number of days
200.0
222.5
245.0
267.5
290.0
(c)
Average number of days
13.00
34.75
56.50
78.25
100.00
(d)
Average number of days
150
255
360
465
570
(e)
Average number of days
10.0
14.5
19.0
23.5
28.0
(f)
Average number of days
120.0
147.5
175.0
202.5
230.0
Osaka
(b)
Average number of days
180.0
202.5
225.0
247.5
270.0
(c)
Average number of days
53.00
64.25
75.50
86.75
98.00
(d)
Average number of days
150.0
242.5
335.0
427.5
520.0
(e)
Average number of days
8.00
12.25
16.50
20.75
25.00
(f)
Average number of days
120
140
160
180
200
Mar, 2017
Jun, 2017
Sep, 2017
Dec, 2017
Mar, 2018
Jun, 2018
Sep, 2018
Dec, 2018
Mar, 2019
Jun, 2019
Sep, 2019
Dec, 2019
Mar, 2020
Jun, 2020

# Hyogo

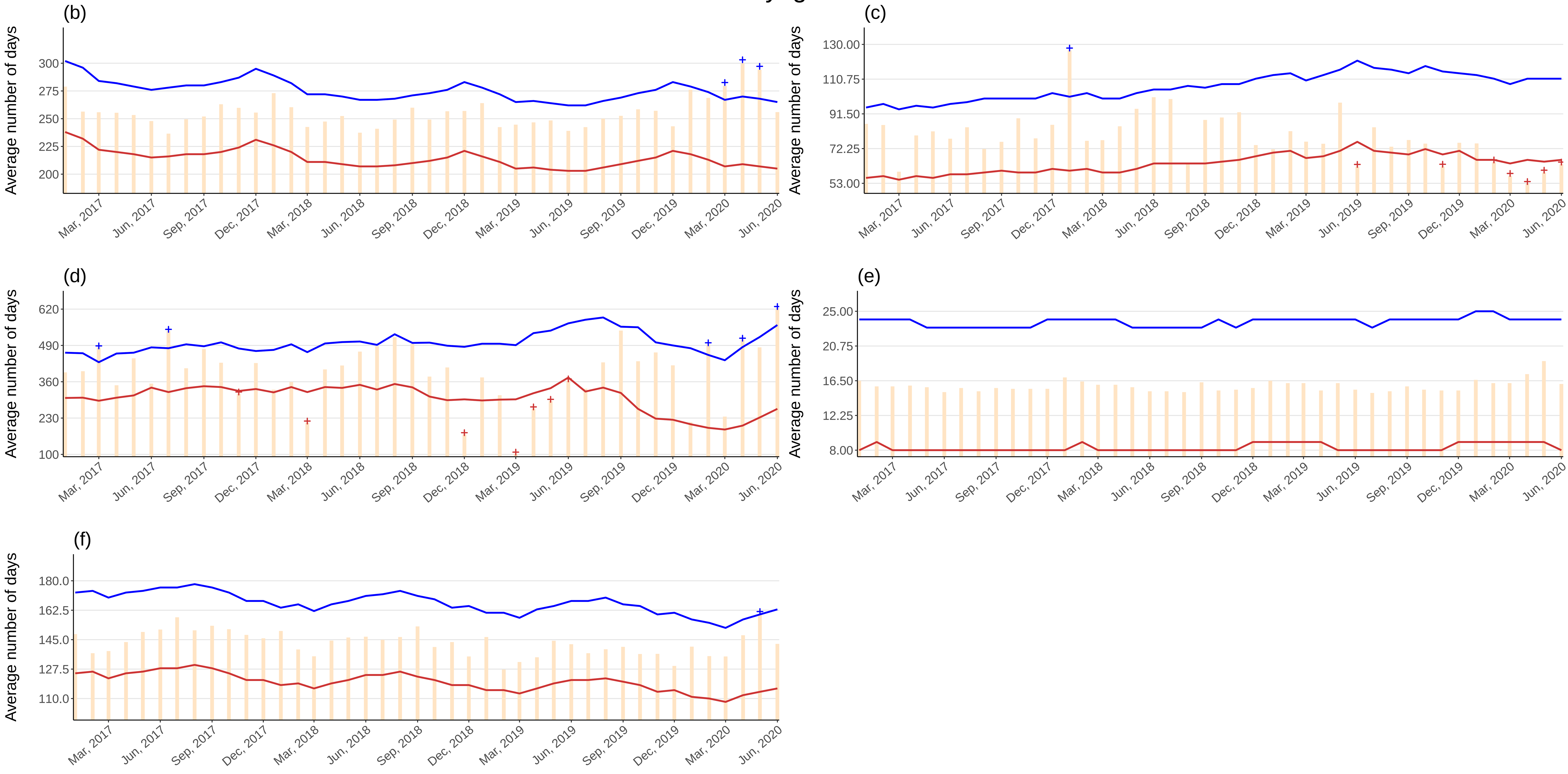


# Nara

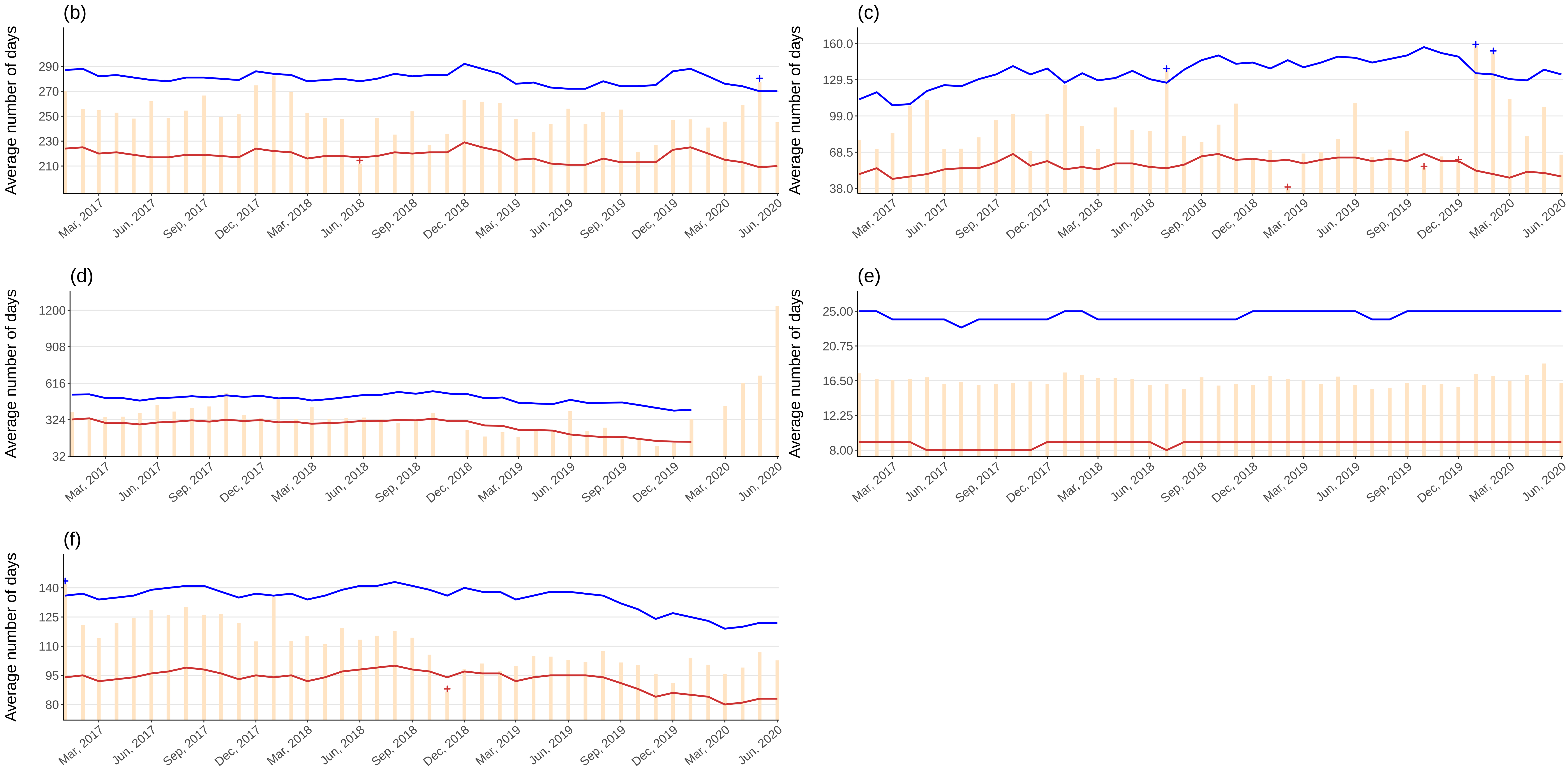


# Wakayama

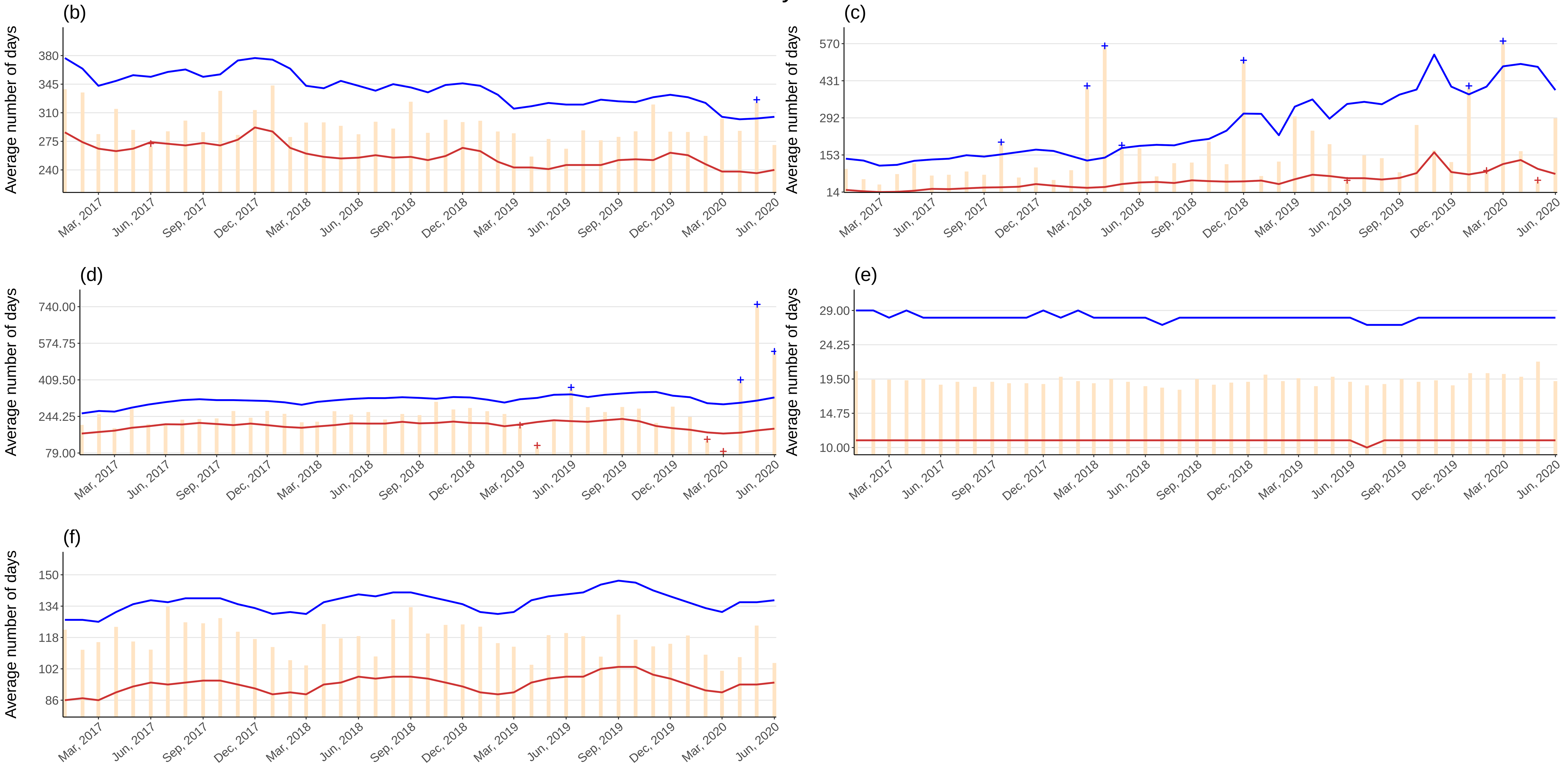

## Tottori

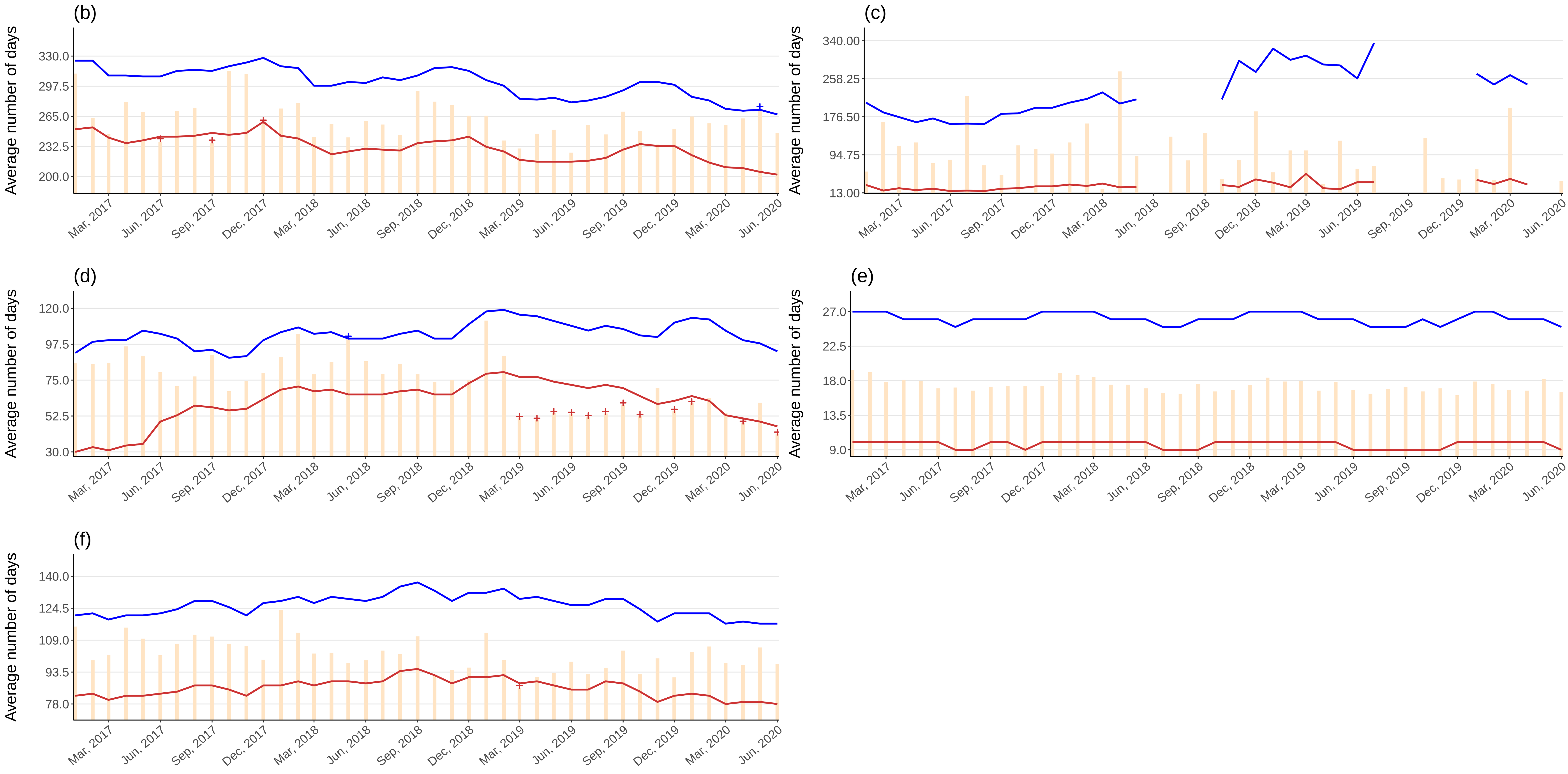


## Shimane

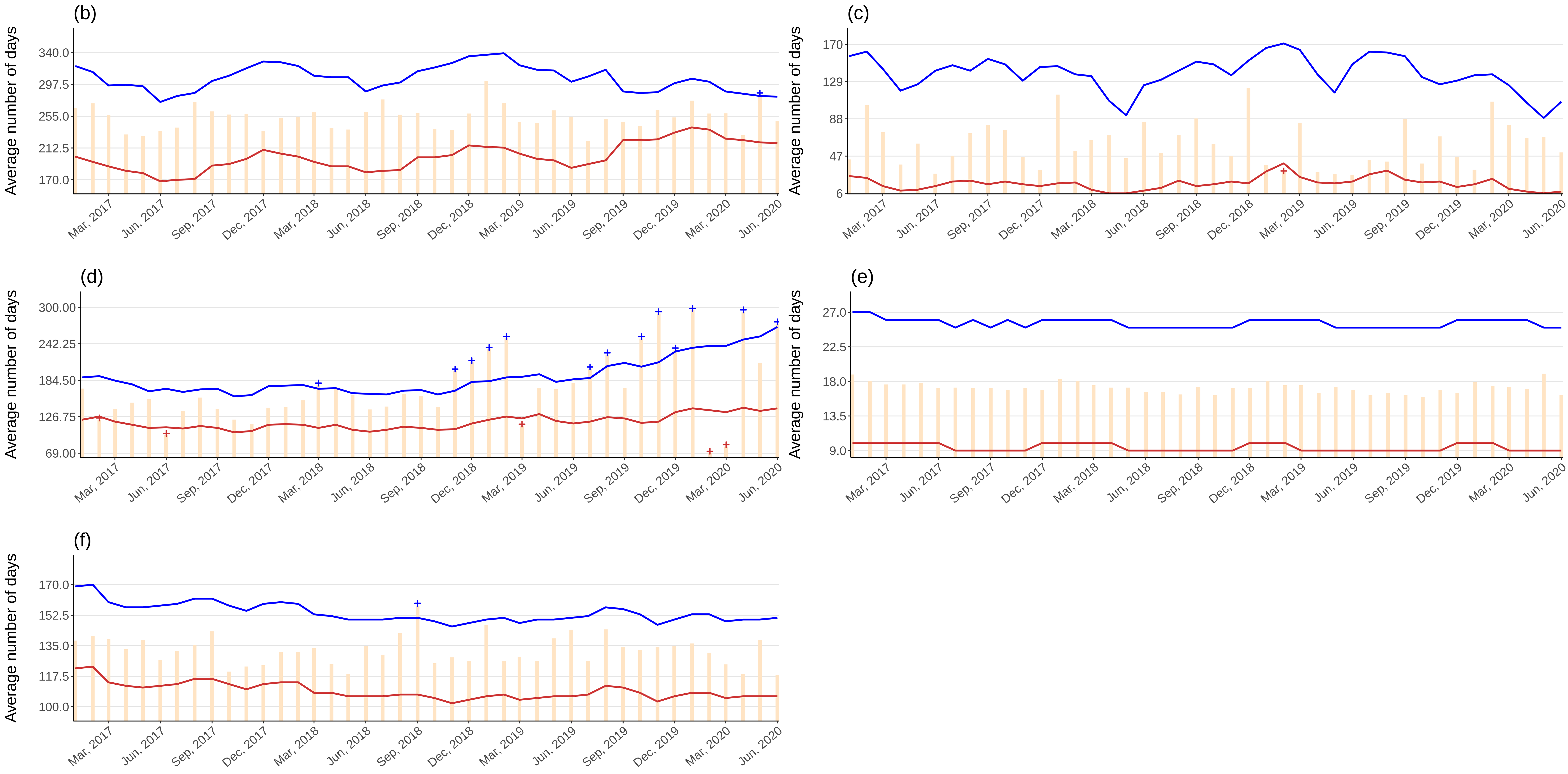


## Okayama

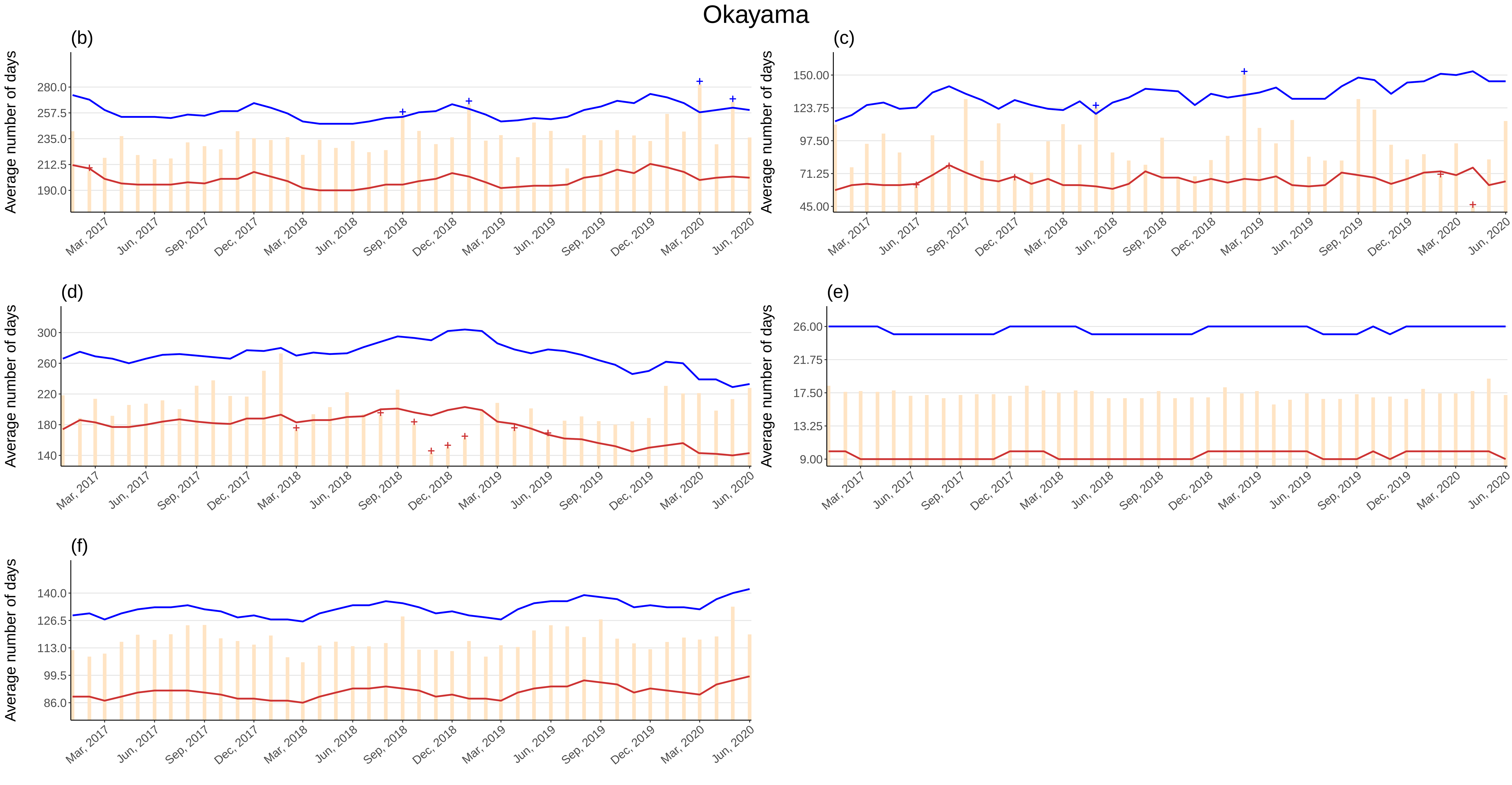

## Hiroshima

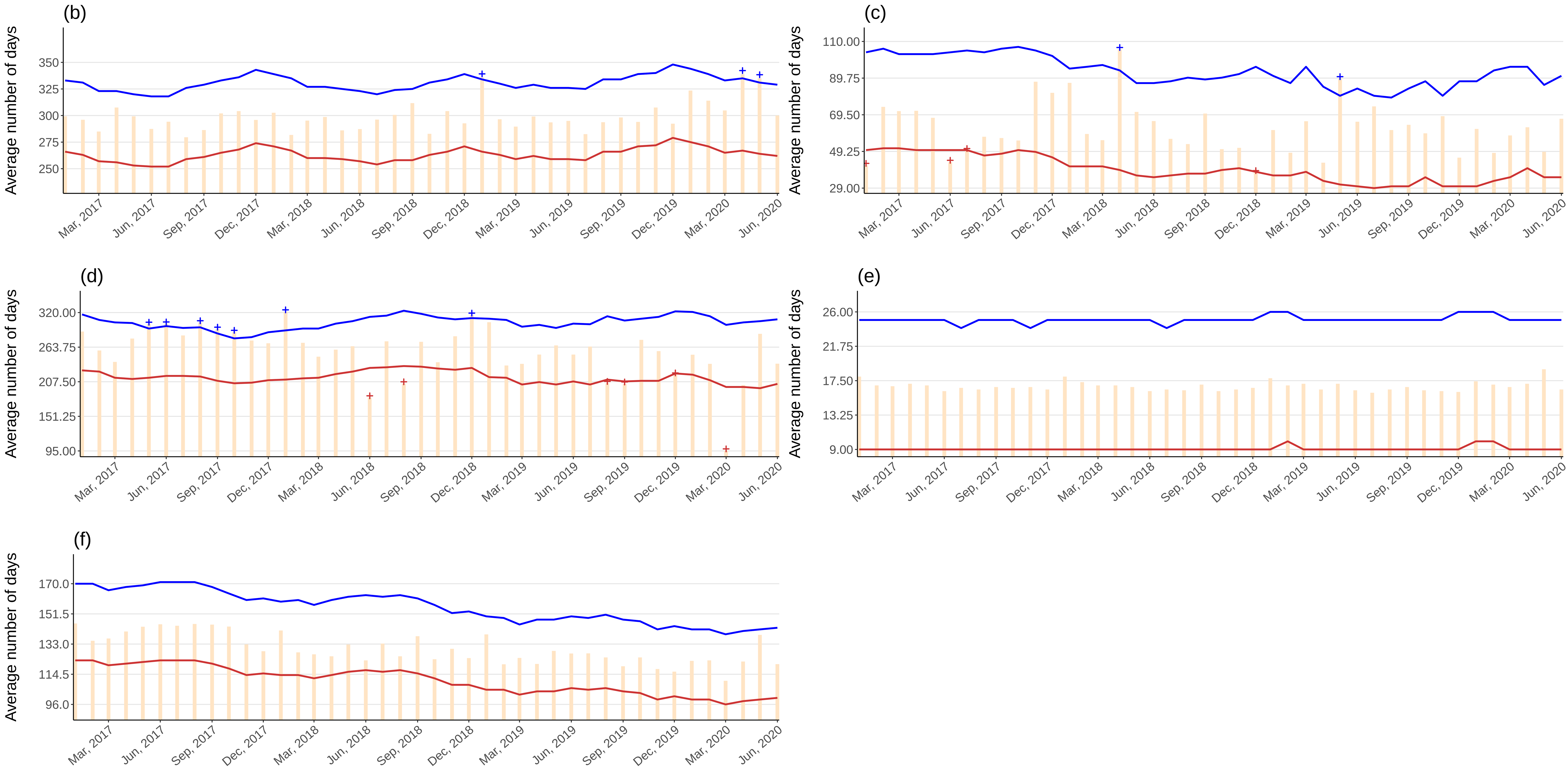


## Yamaguchi

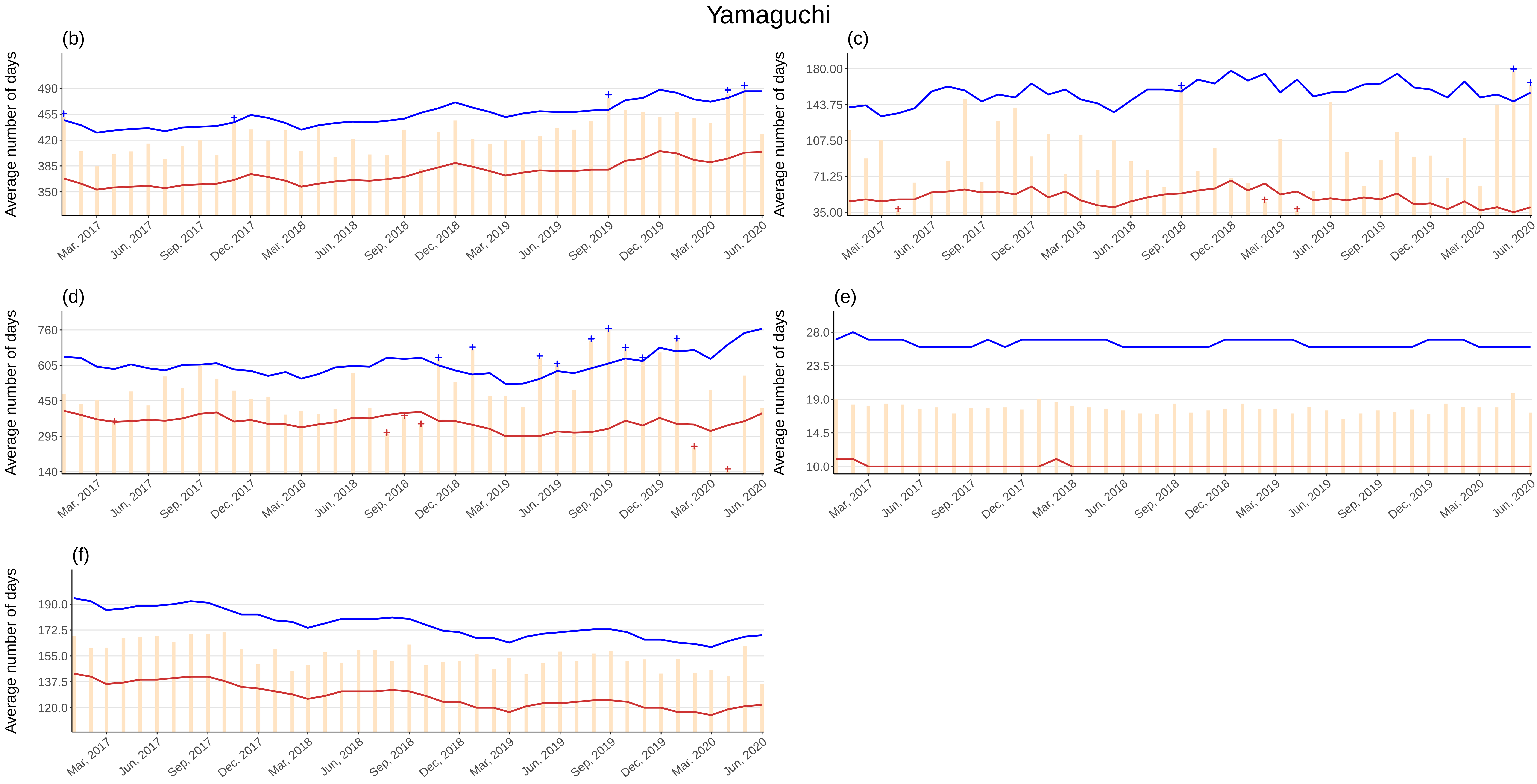


## Tokushima

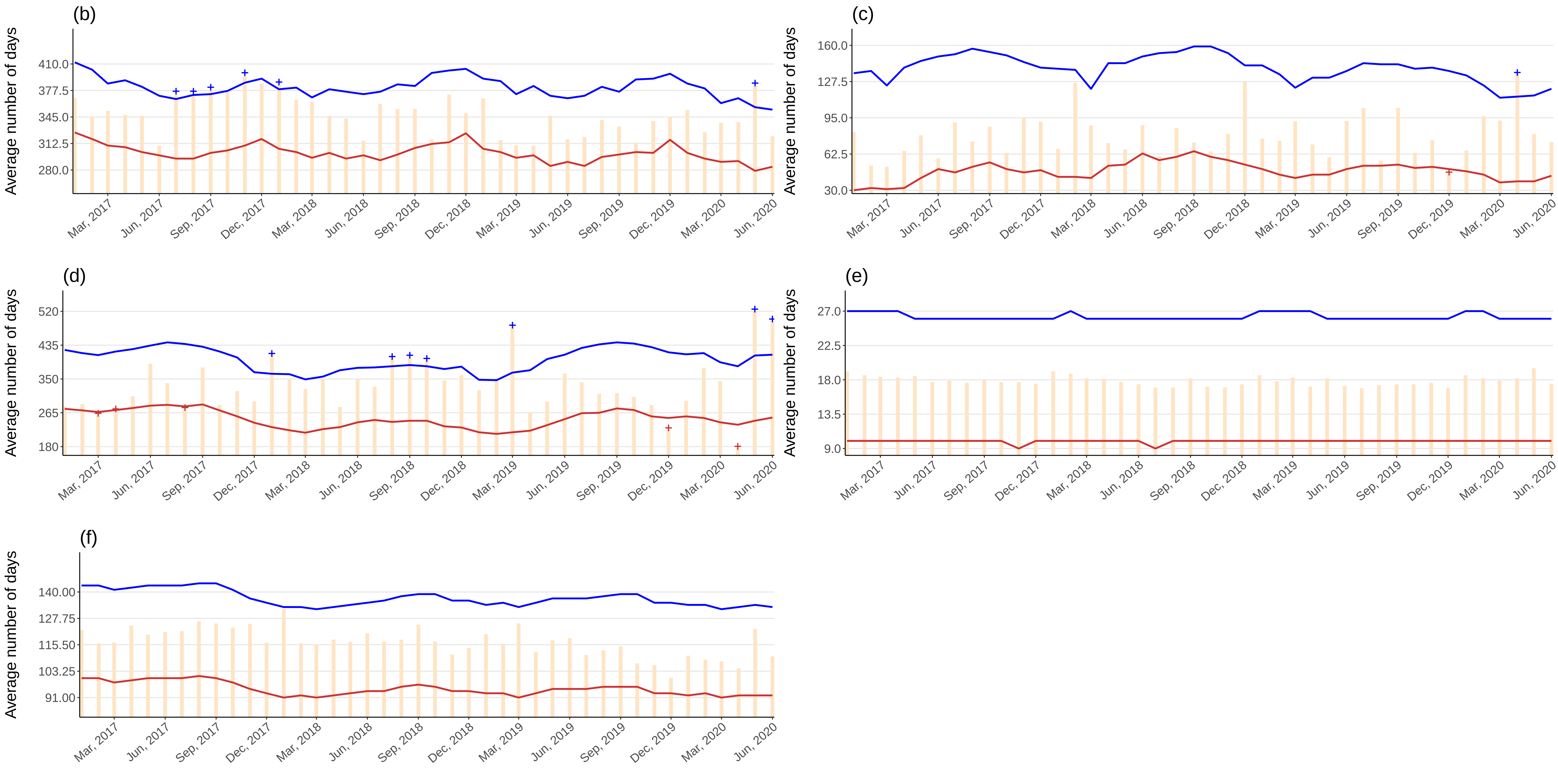

# Kagawa

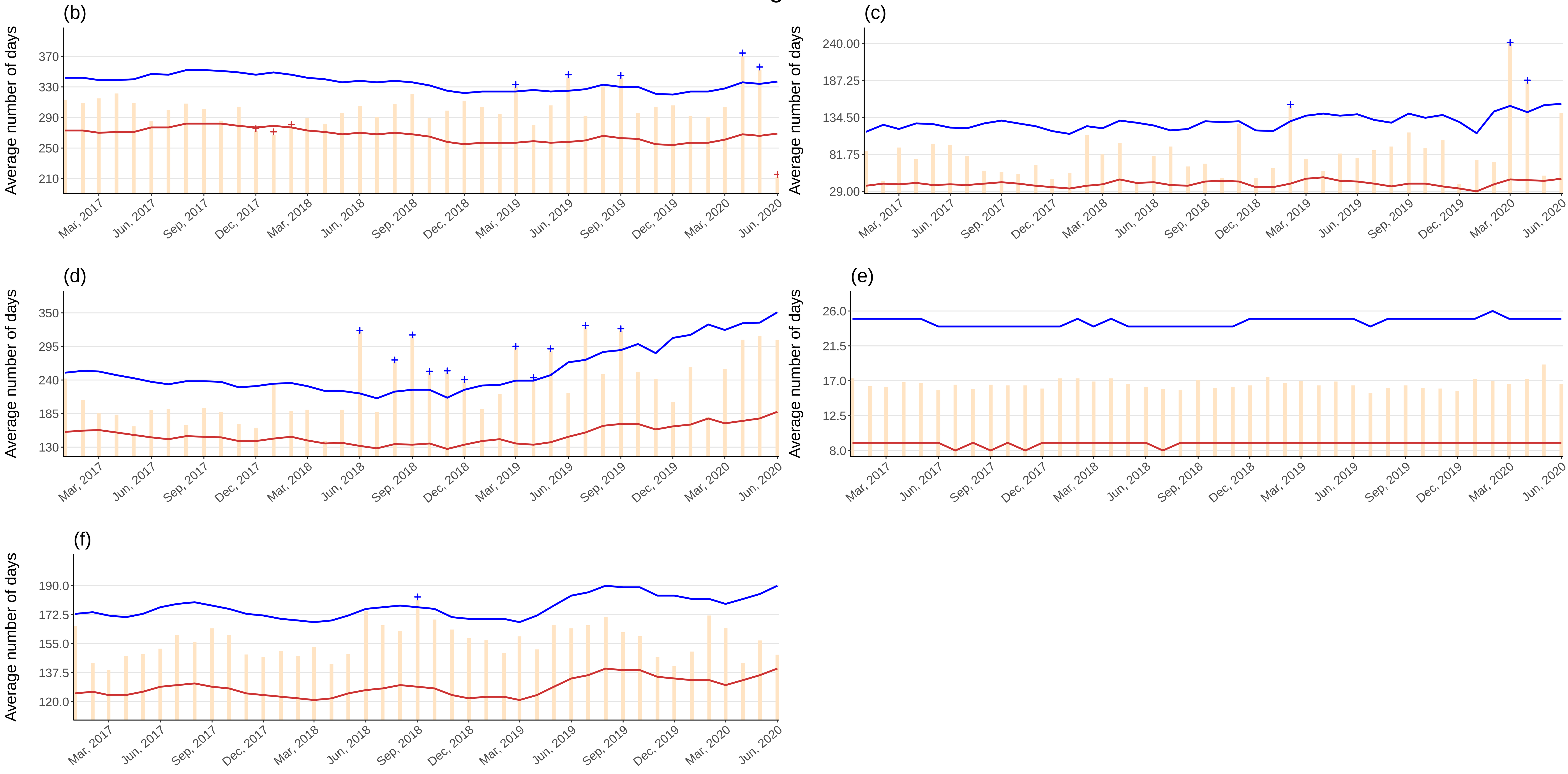


# Ehime

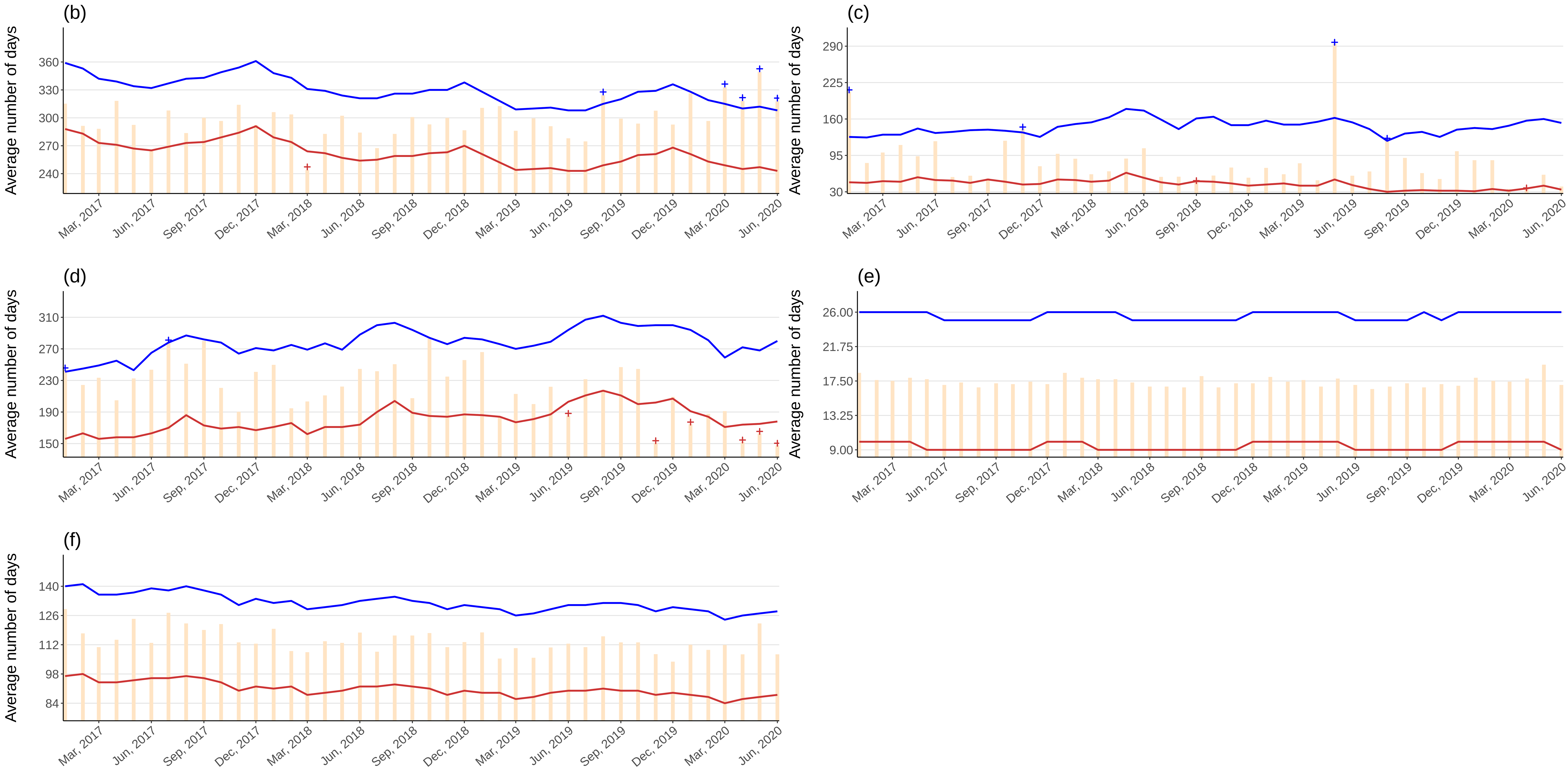


# Kochi

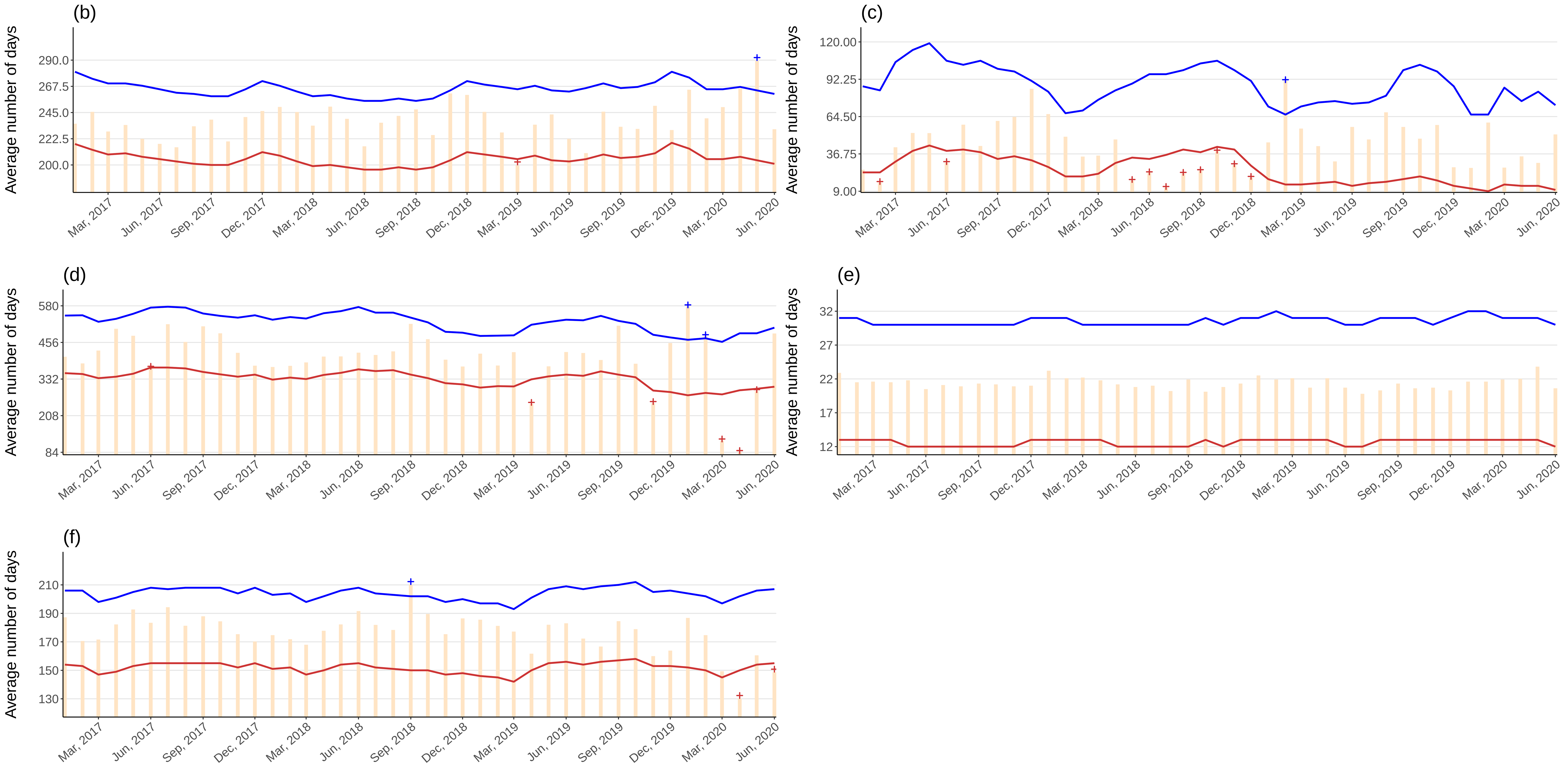

# Fukuoka

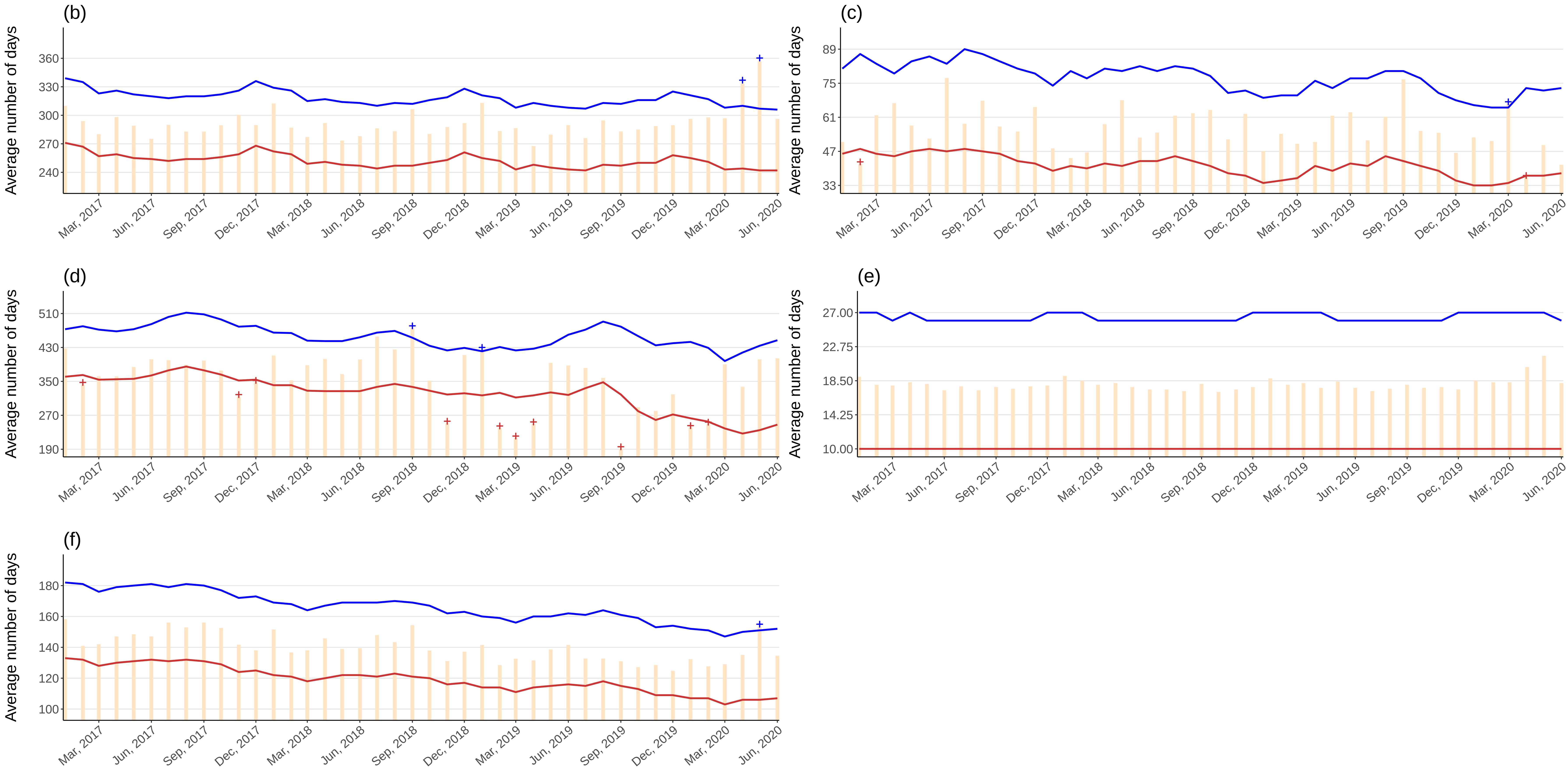


# Saga

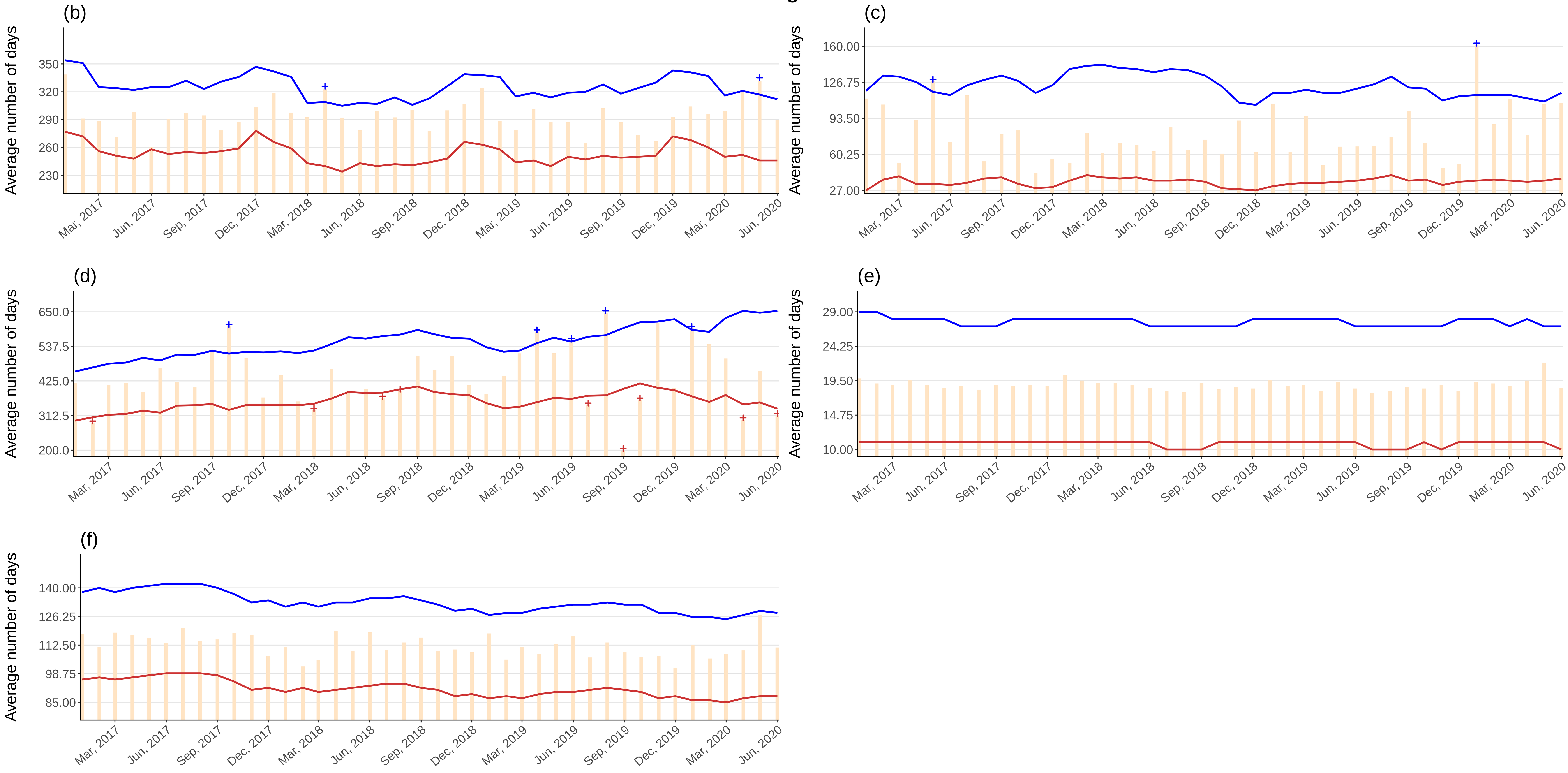


# Nagasaki

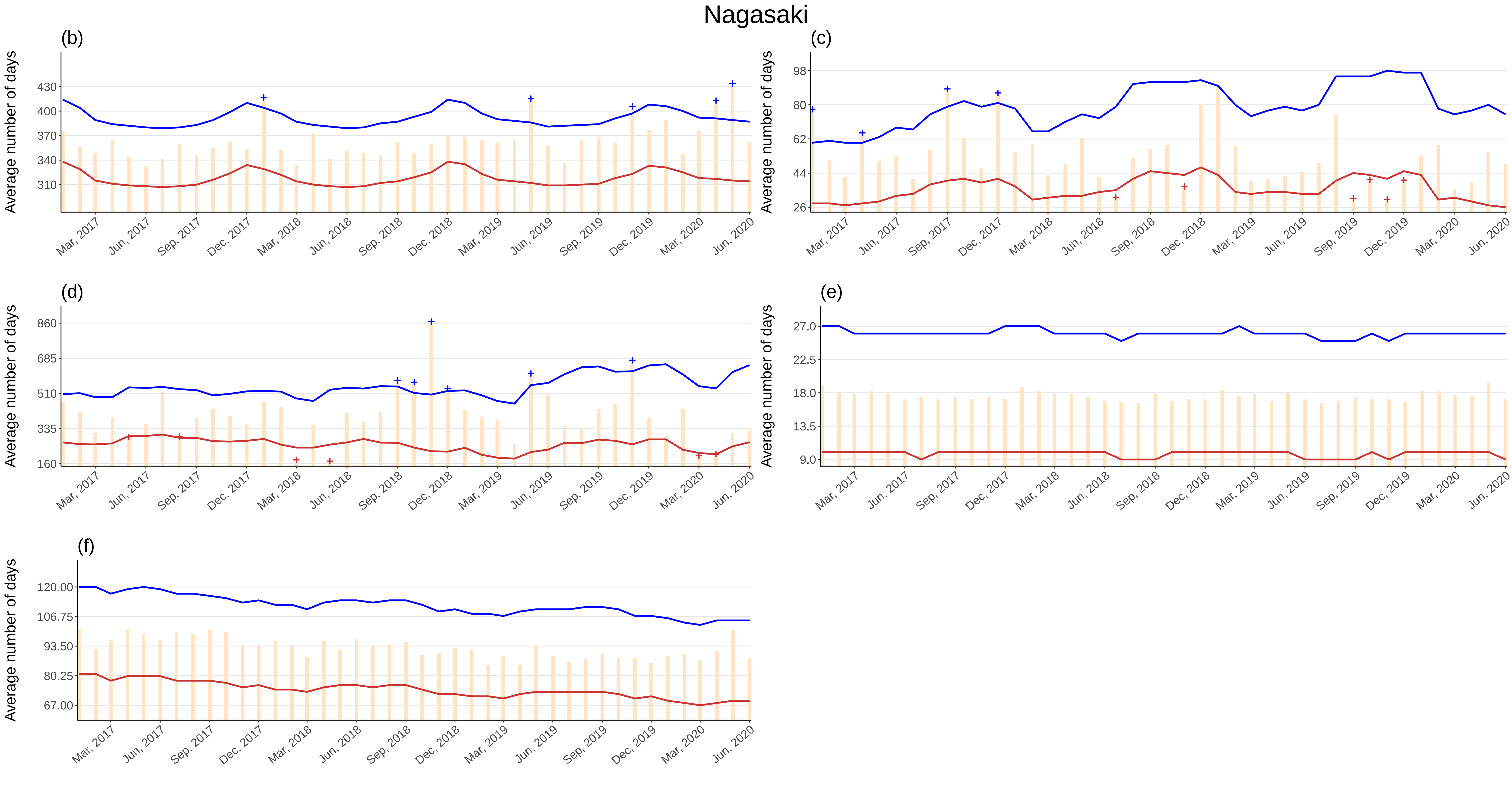

## Kumamoto

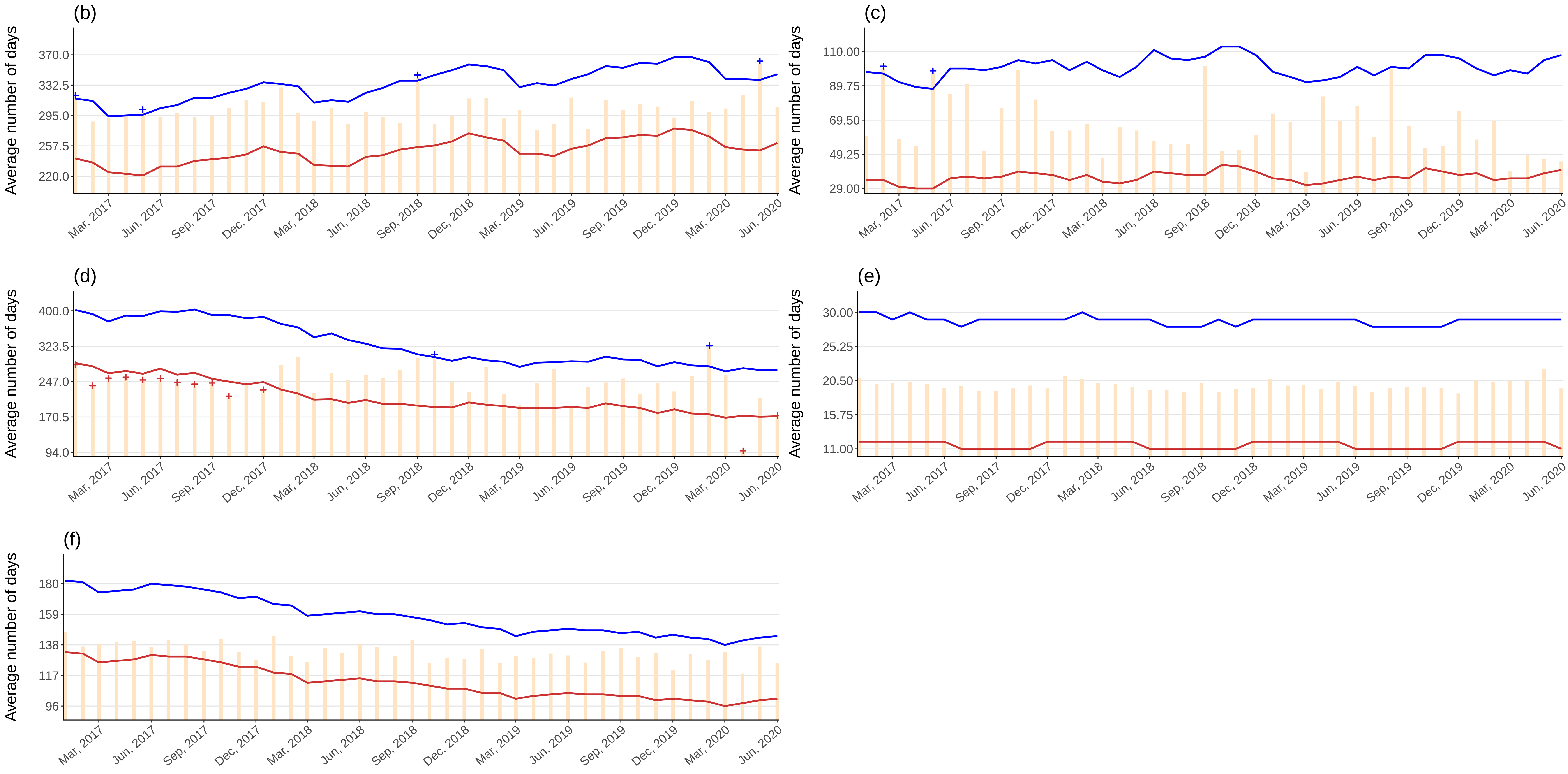


## Oita

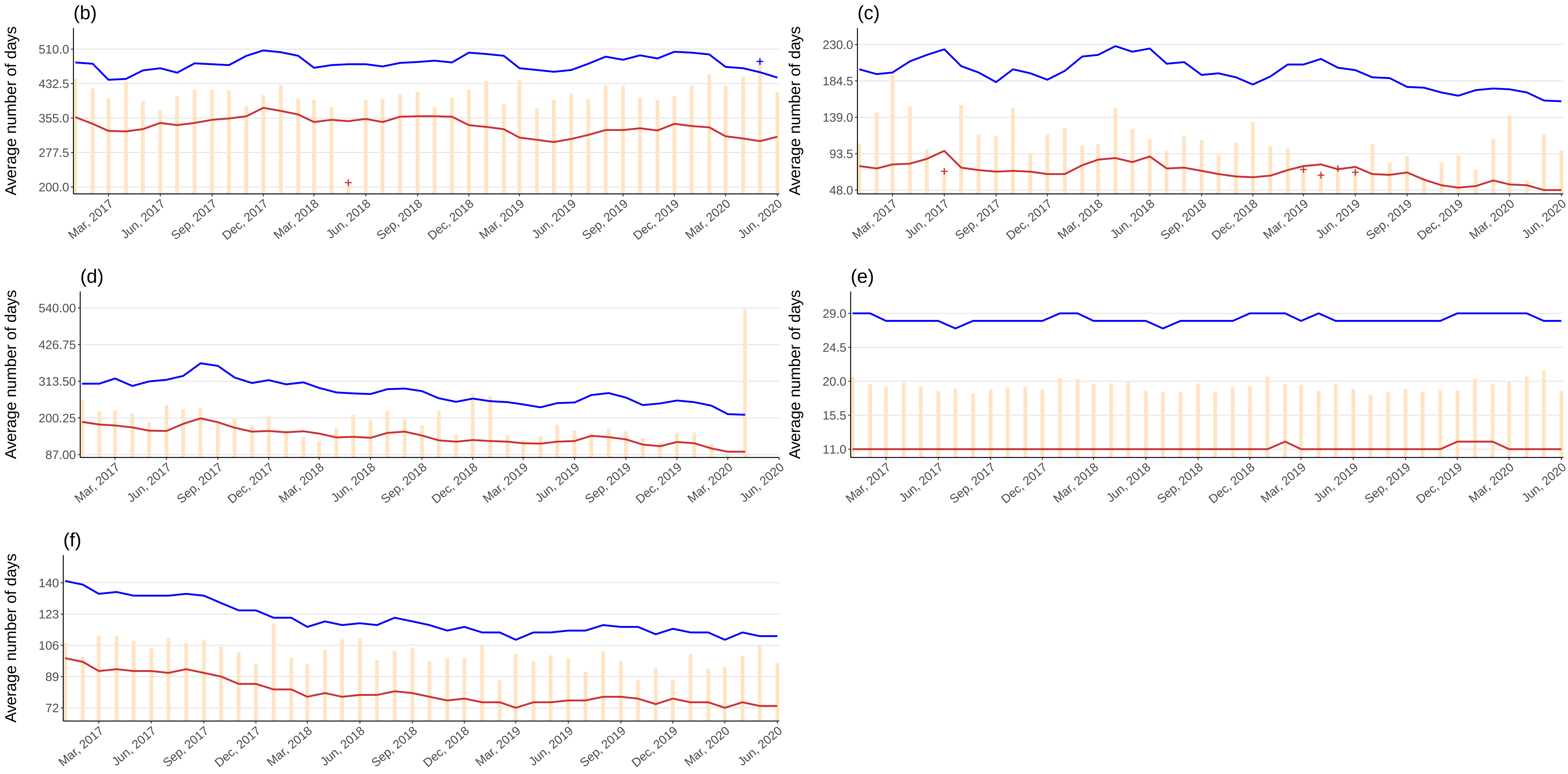


## Miyazaki

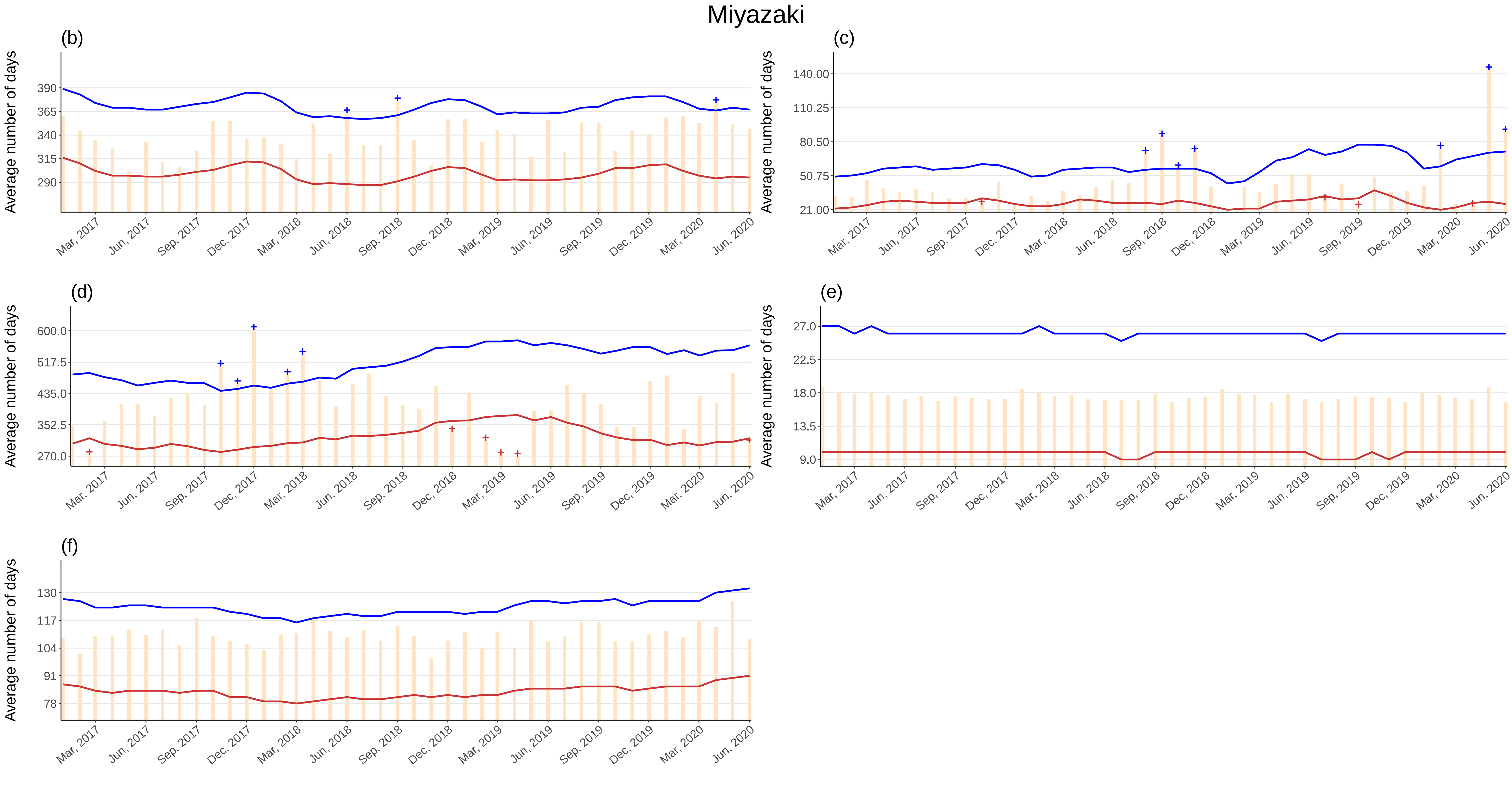

# Kagoshima

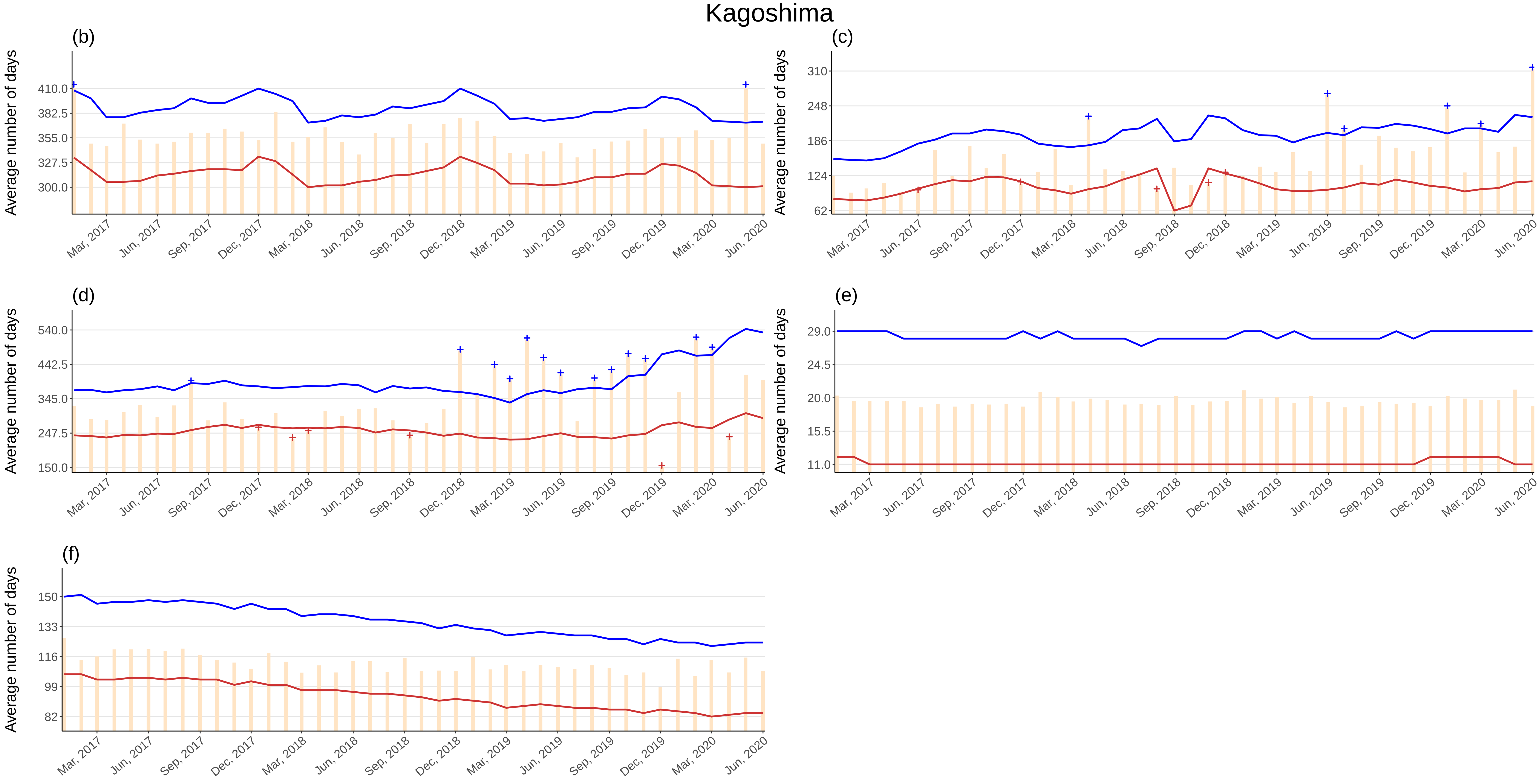


# Okinawa

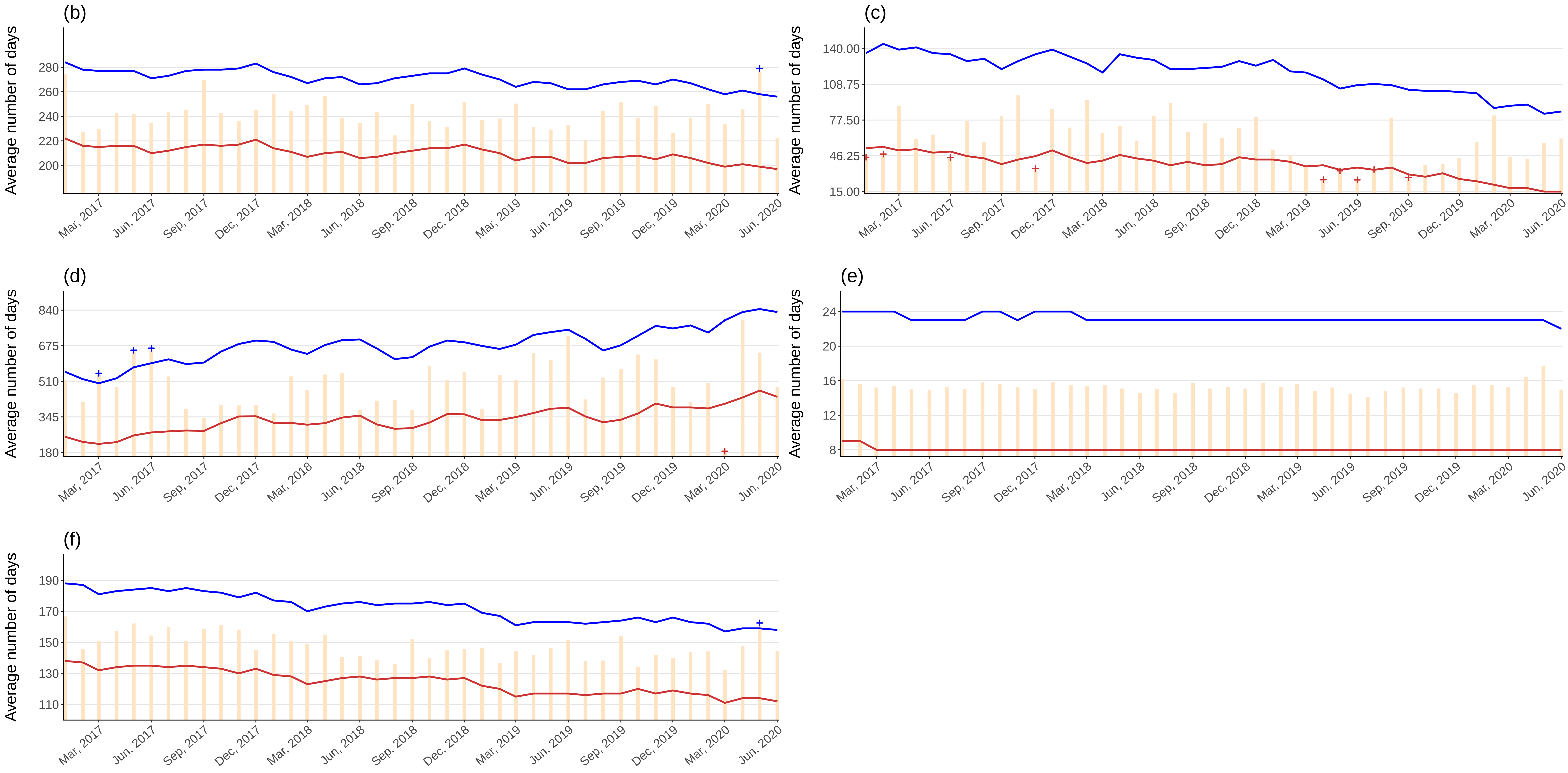

Supplement: Supplementary file 1 [file ijerph-18-03271-s001.zip › Appendix Figure 3.pdf]
