# Supplementary material for: Trends in Healthcare Access in Japan during the First Wave of the COVID-19 Pandemic, up to June 2020
Source: Int J Environ Res Public Health. 2021 Mar 22;18(6):3271. doi: 10.3390/ijerph18063271 (PMC8004161; doi:10.3390/ijerph18063271)

Hokkaido

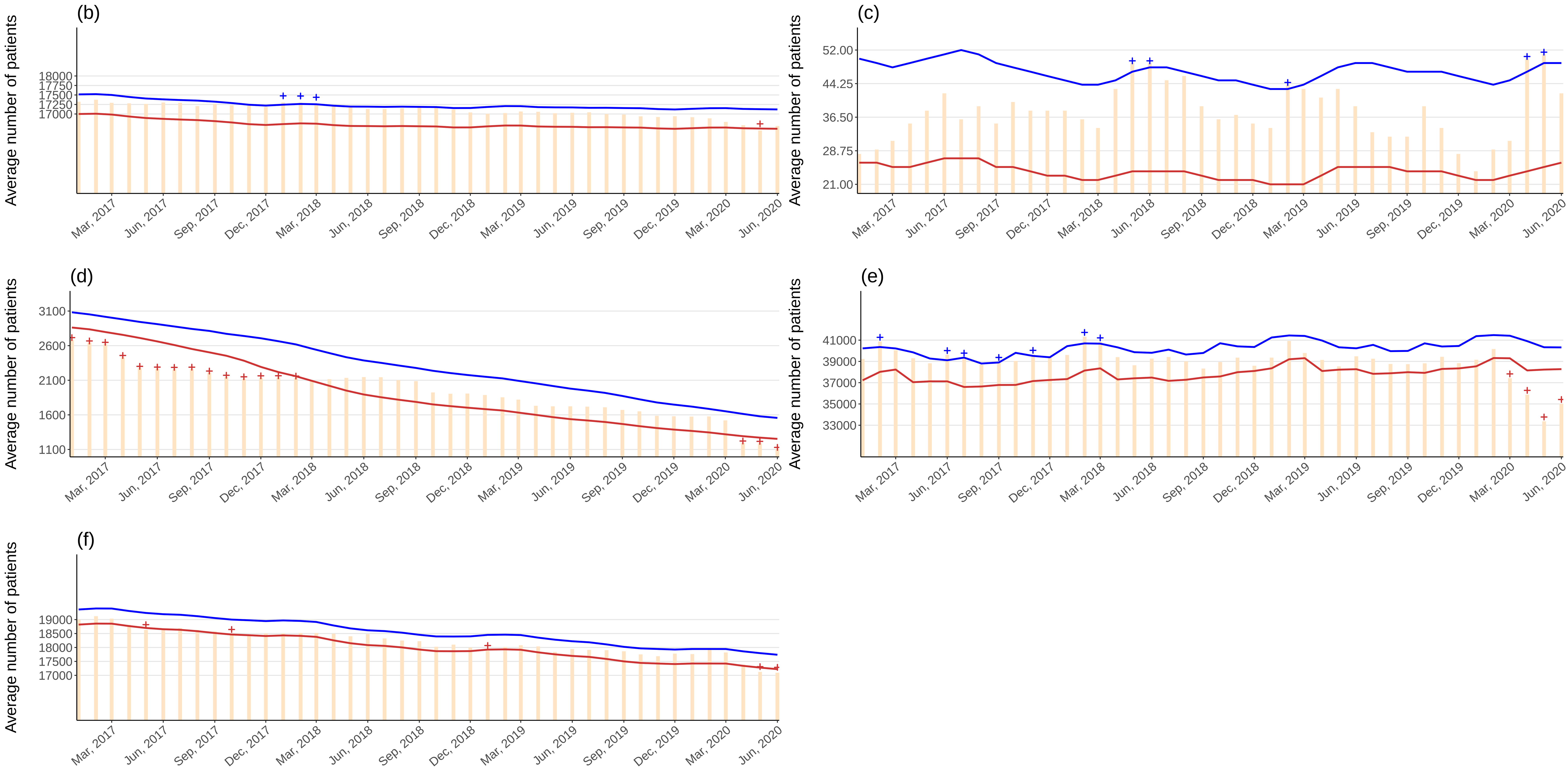

Aomori

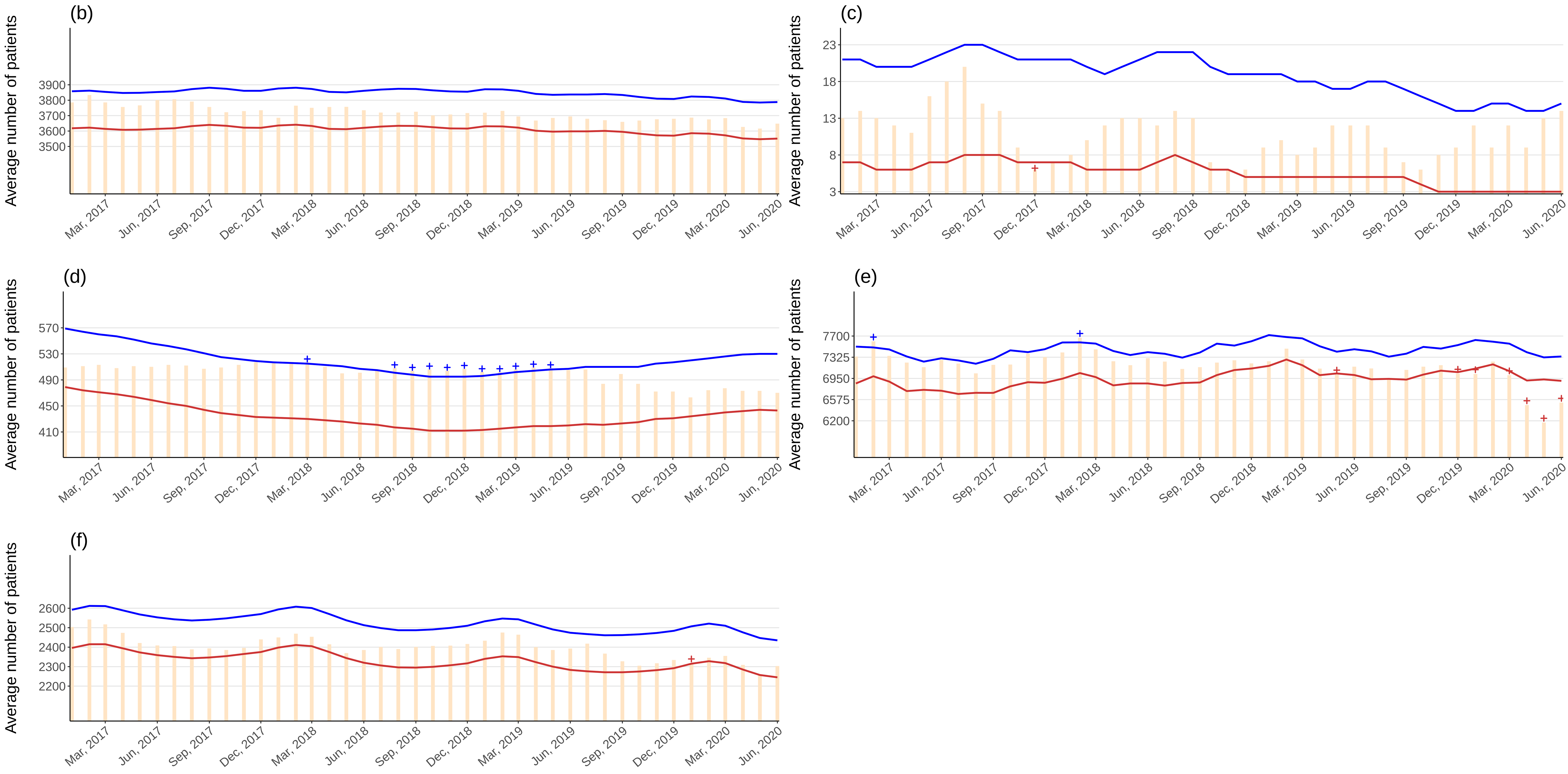

Iwate

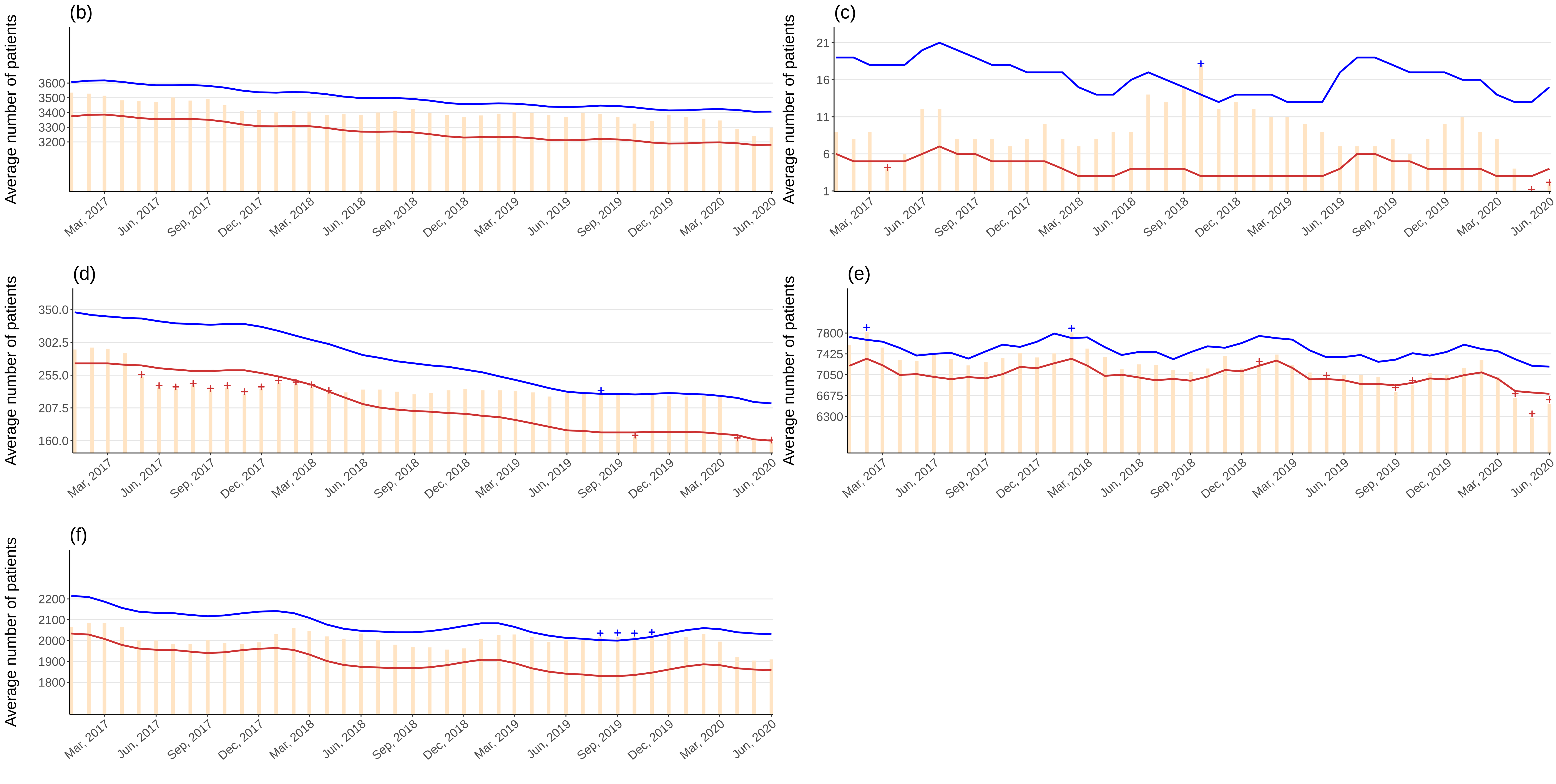

Miyagi

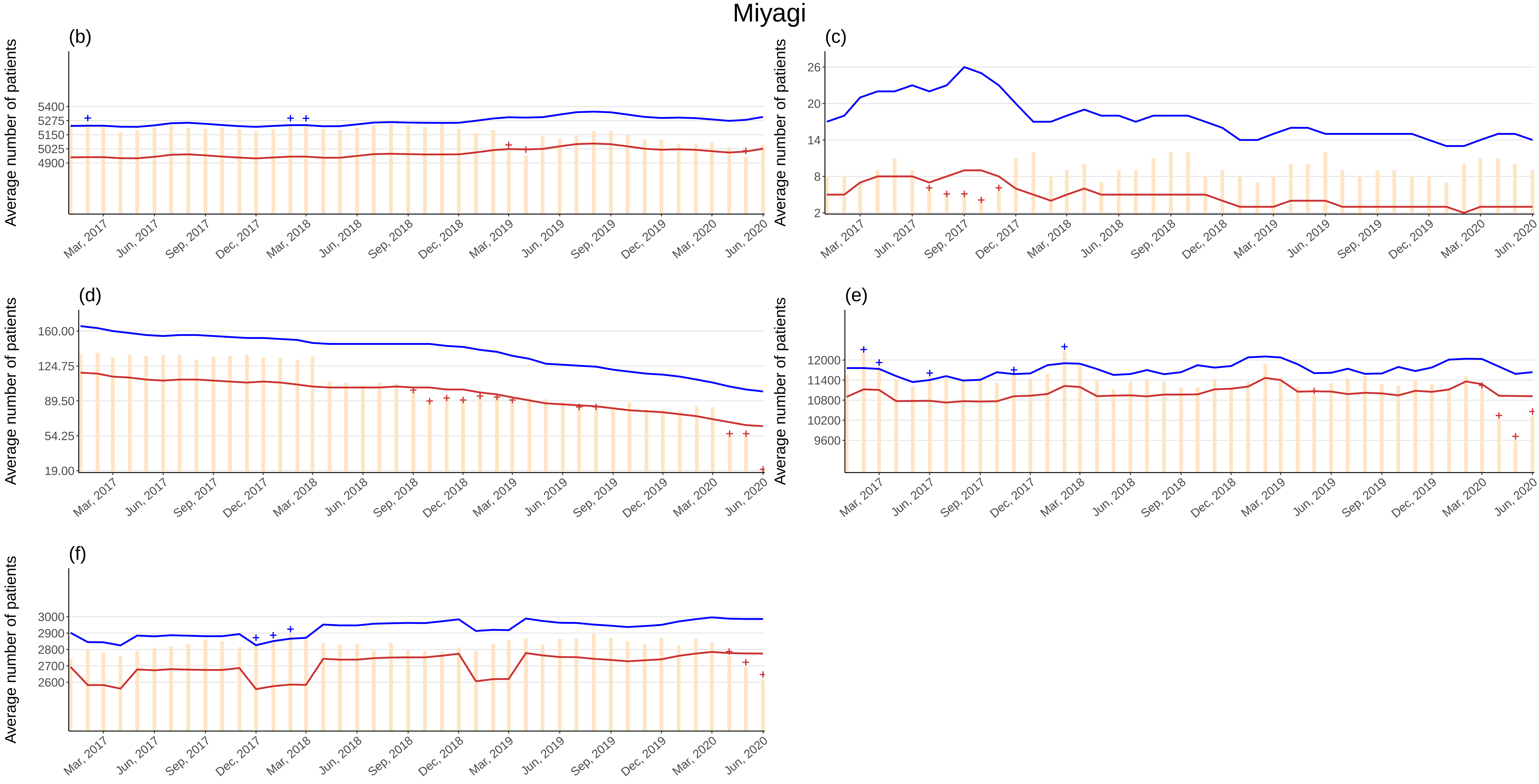

Akita

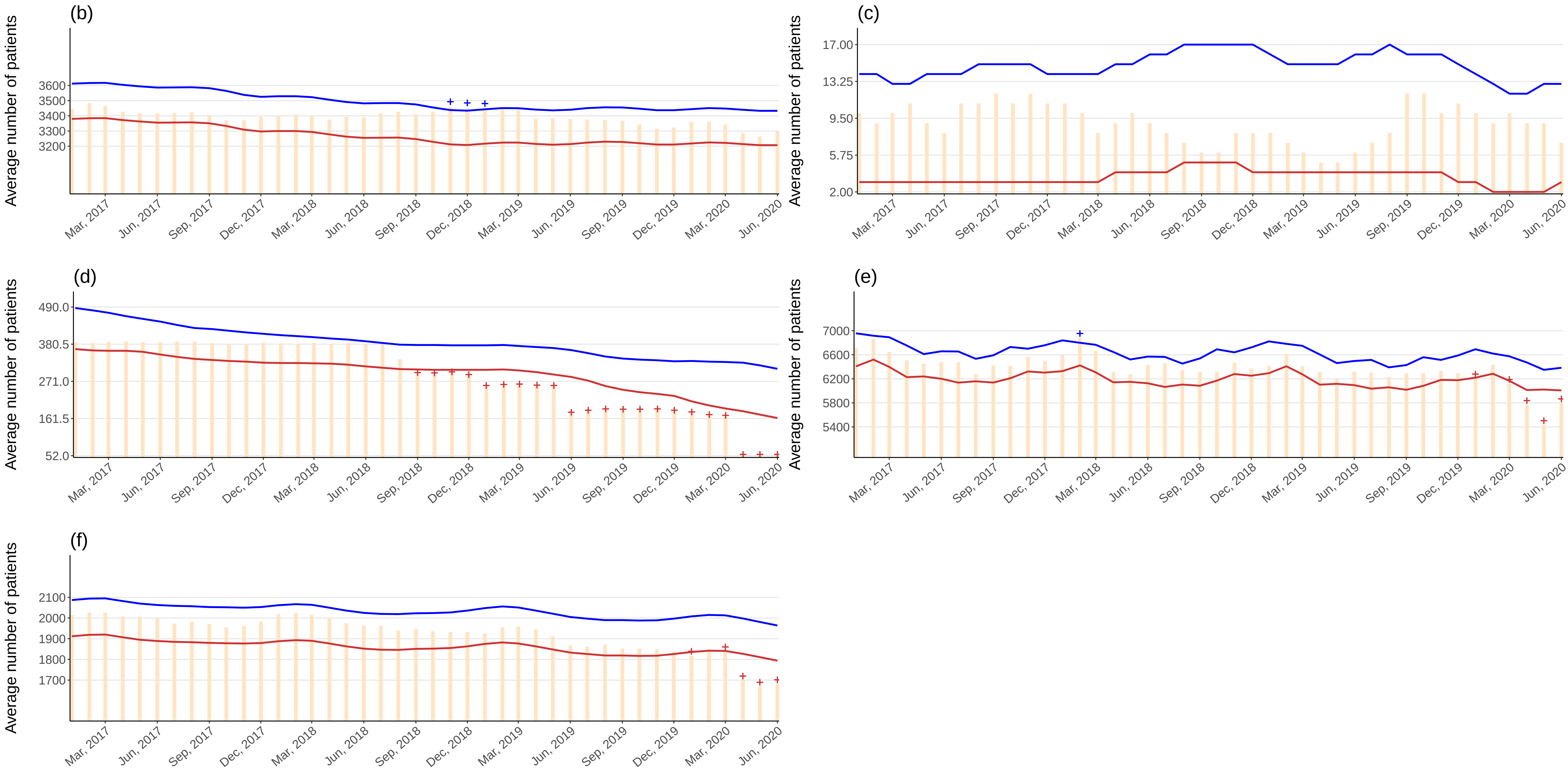

Yamagata

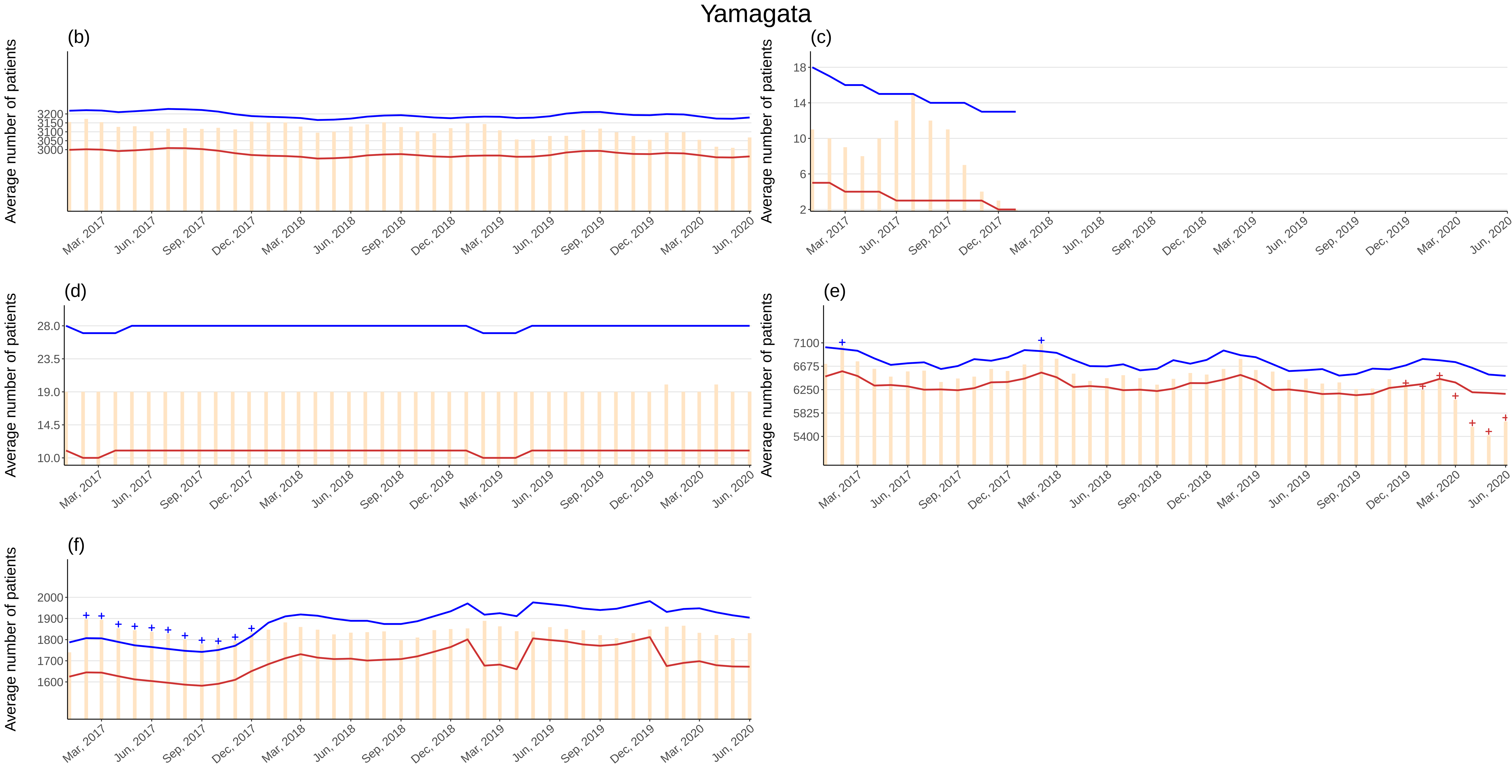

Fukushima

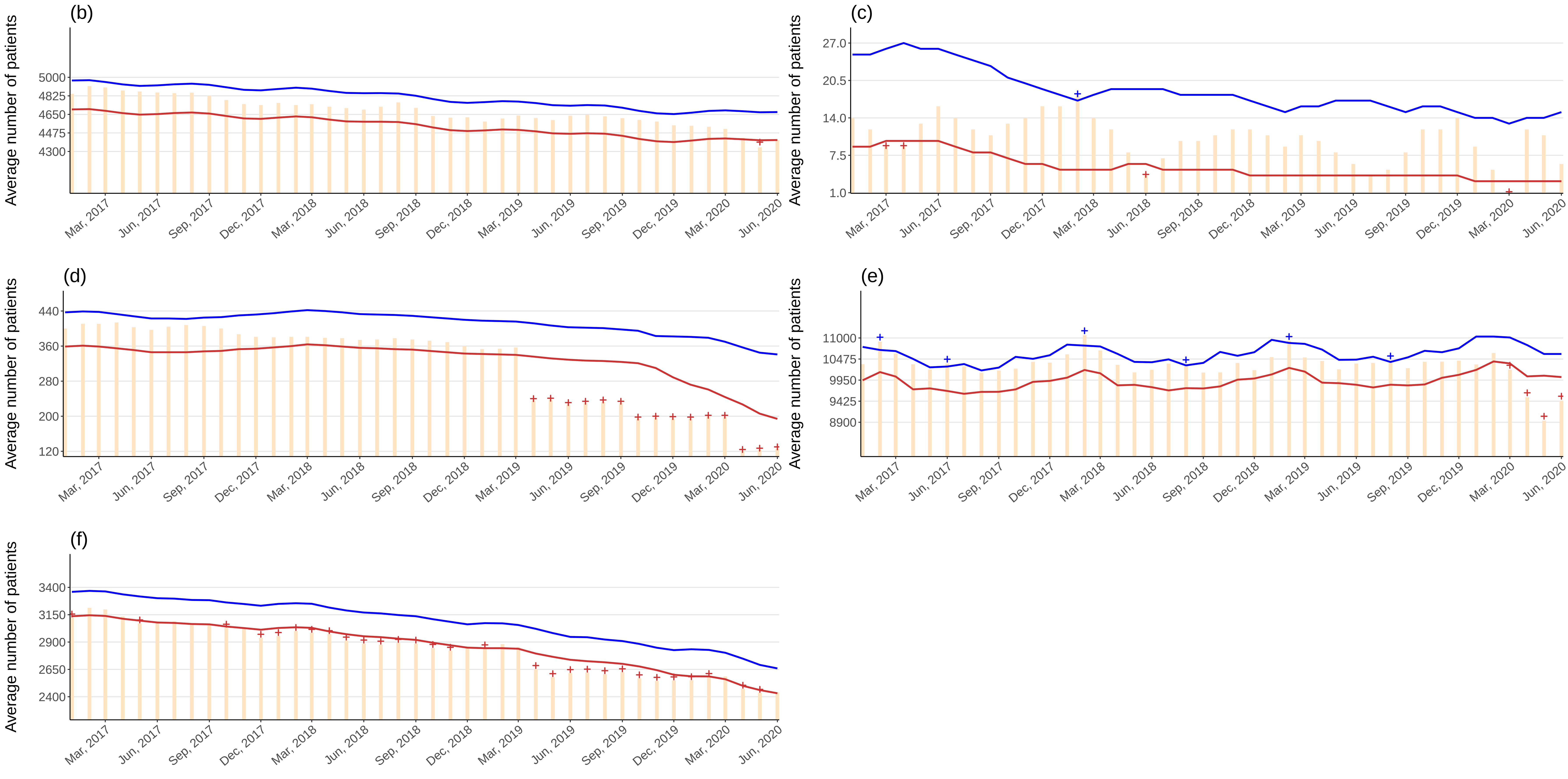

Ibaraki

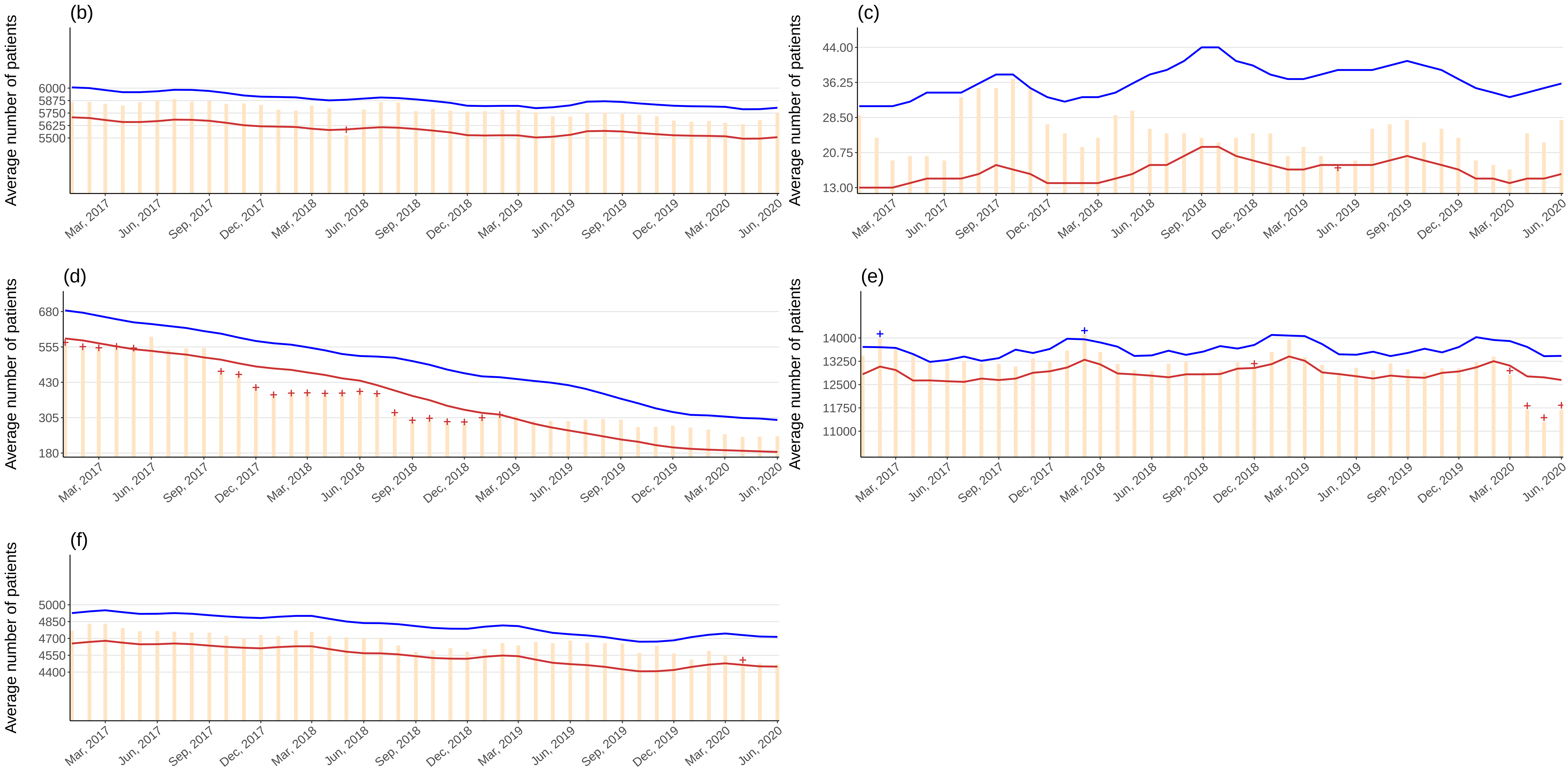

Tochigi

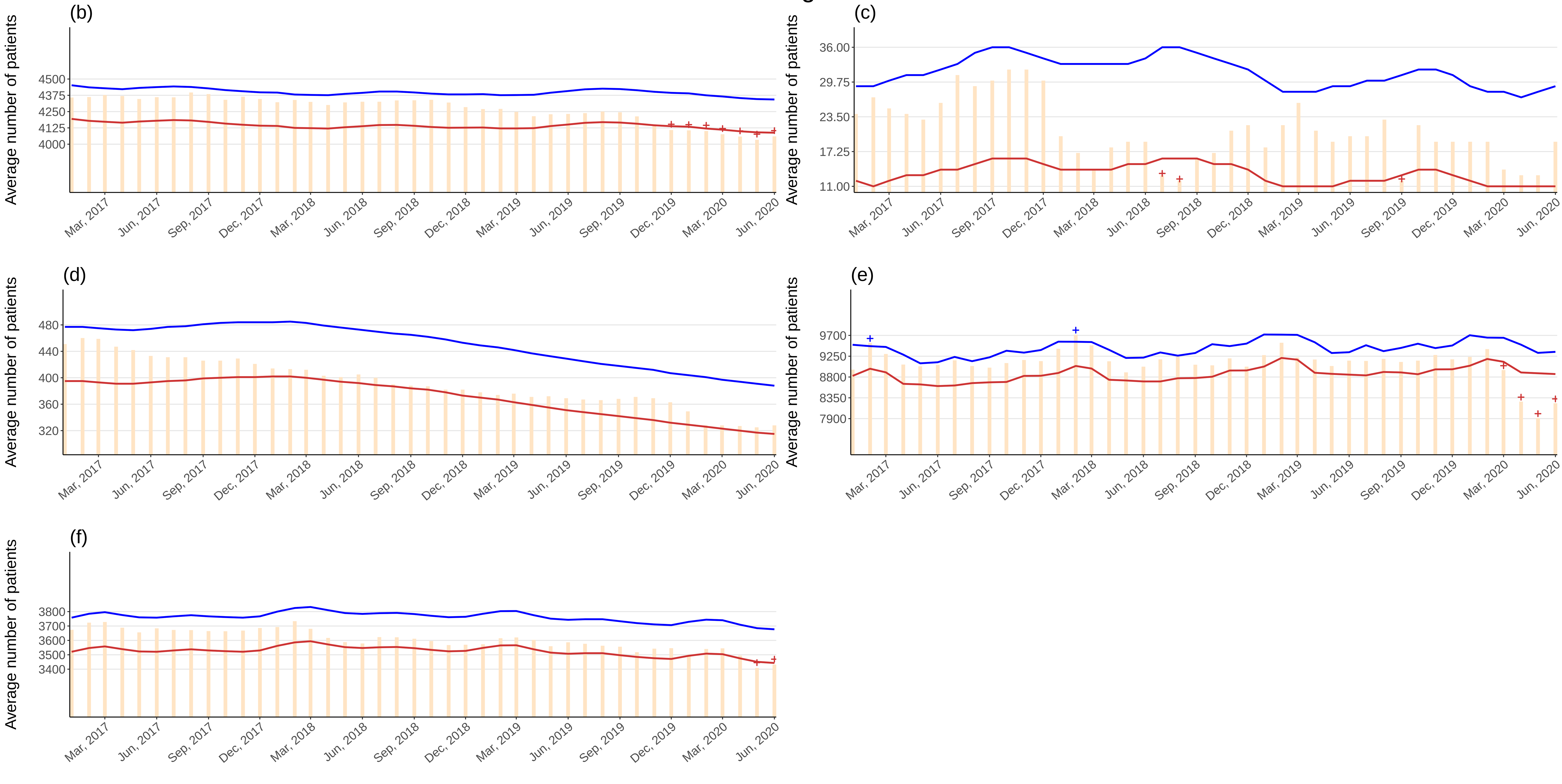

Gunma

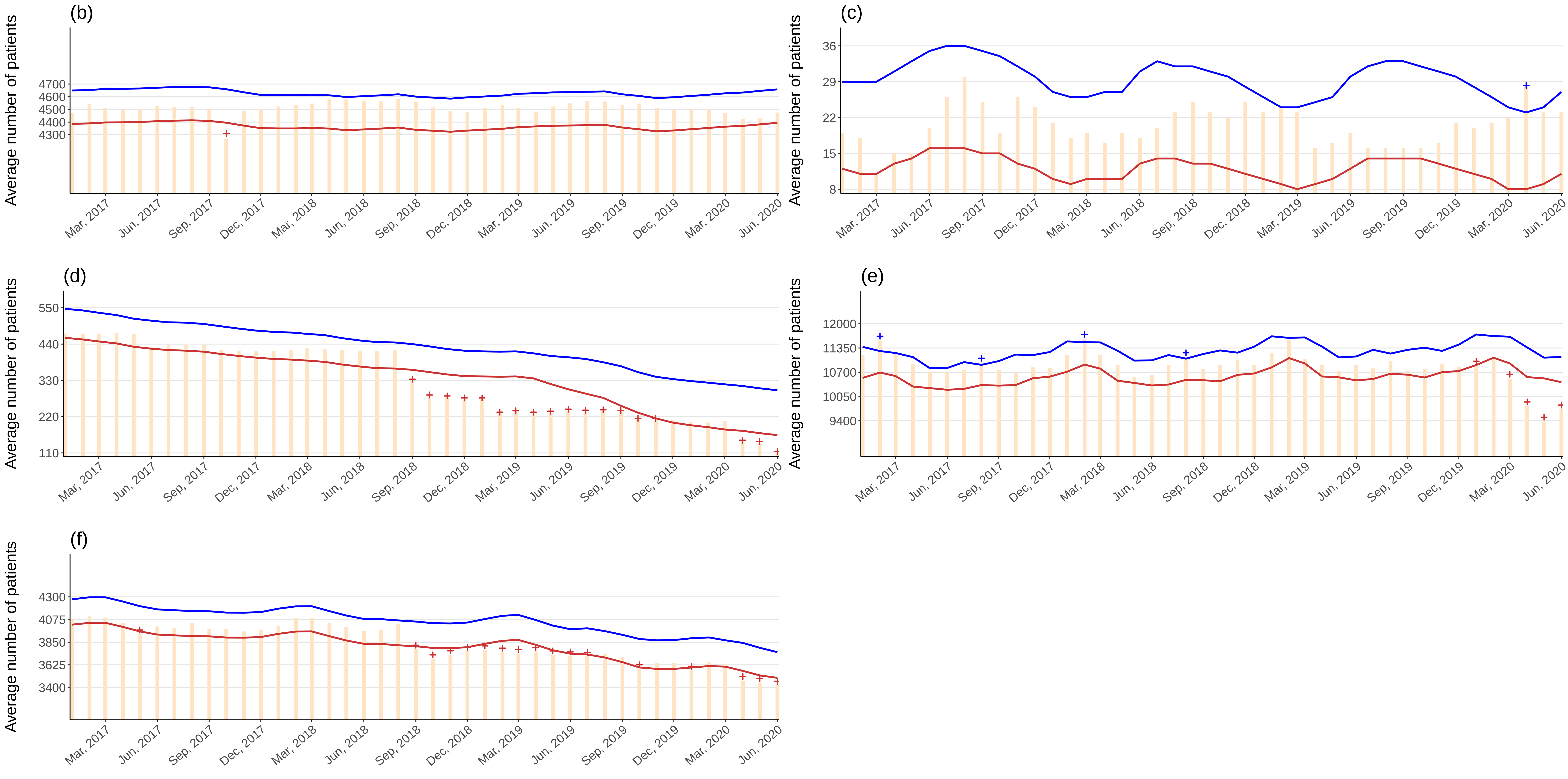

Saitama

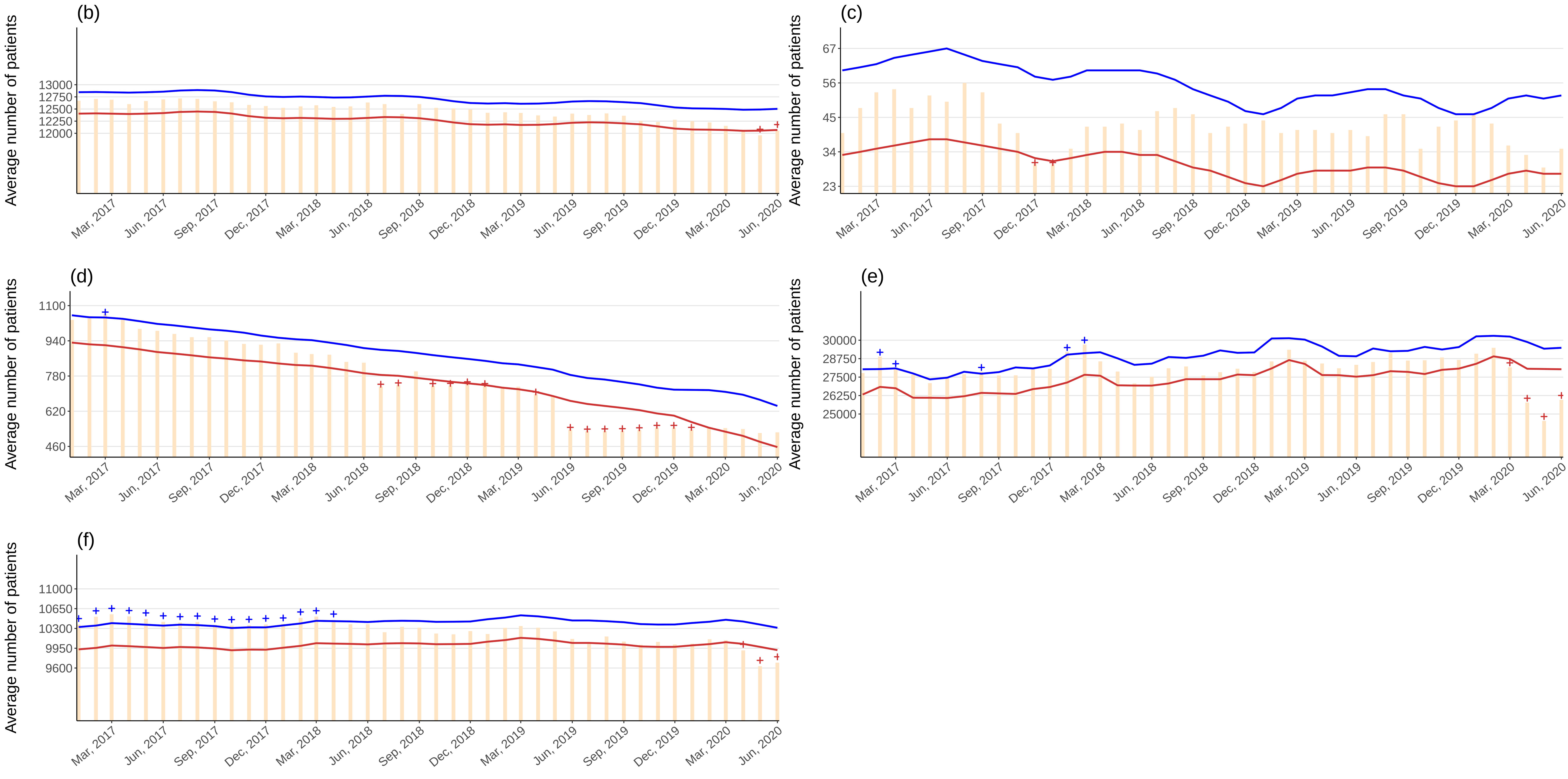

Chiba

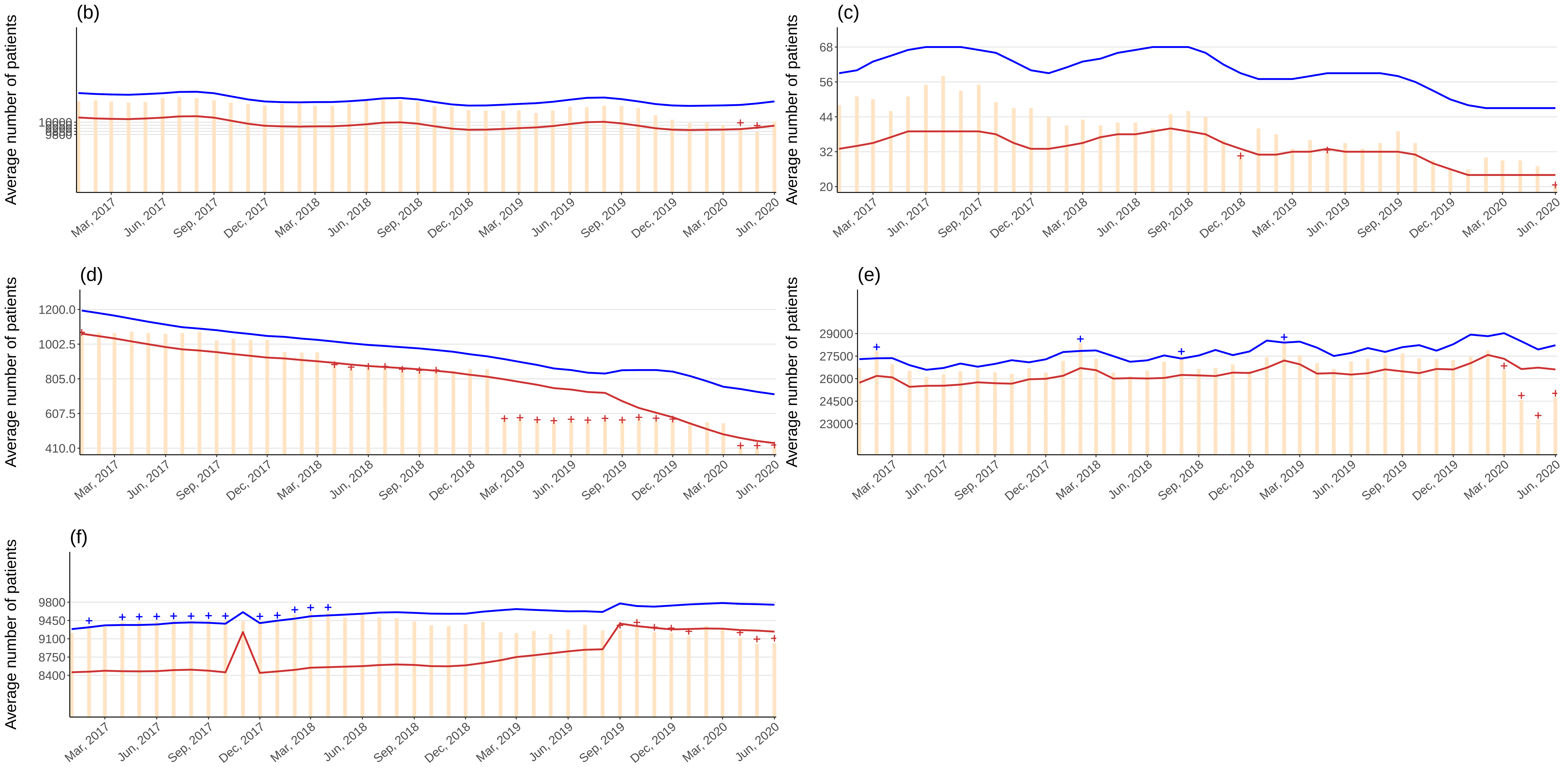

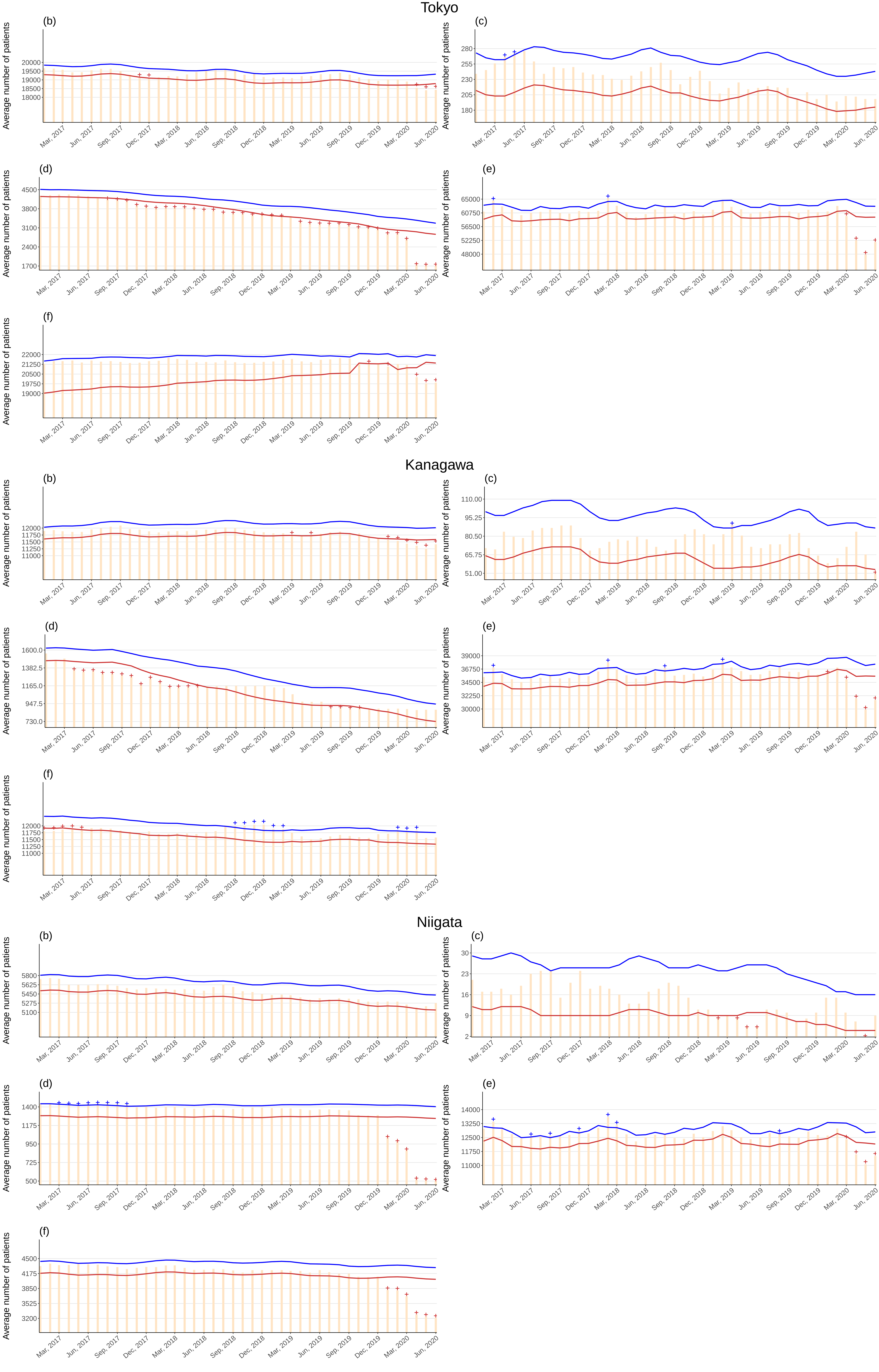
Tokyo
(b)
20000
19500
19000
18500
18000
(c)
280
255
230
205
180
(d)
4500
3800
3100
2400
1700
(e)
65000
60750
56500
52250
48000
(f)
22000
21250
20500
19750
19000
Kanagawa
(b)
12000
11750
11500
11250
11000
(c)
110.00
95.25
80.50
65.75
51.00
(d)
1600.0
1382.5
1165.0
947.5
730.0
(e)
39000
36750
34500
32250
30000
(f)
12000
11750
11500
11250
11000
Niigata
(b)
5800
5625
5450
5275
5100
(c)
30
23
16
9
2
(d)
1400
1175
950
725
500
(e)
14000
13250
12500
11750
11000
(f)
4500
4175
3850
3525
3200
Average number of patients
Mar, 2017
Jun, 2017
Sep, 2017
Dec, 2017
Mar, 2018
Jun, 2018
Sep, 2018
Dec, 2018
Mar, 2019
Jun, 2019
Sep, 2019
Dec, 2019
Mar, 2020
Jun, 2020

Toyama

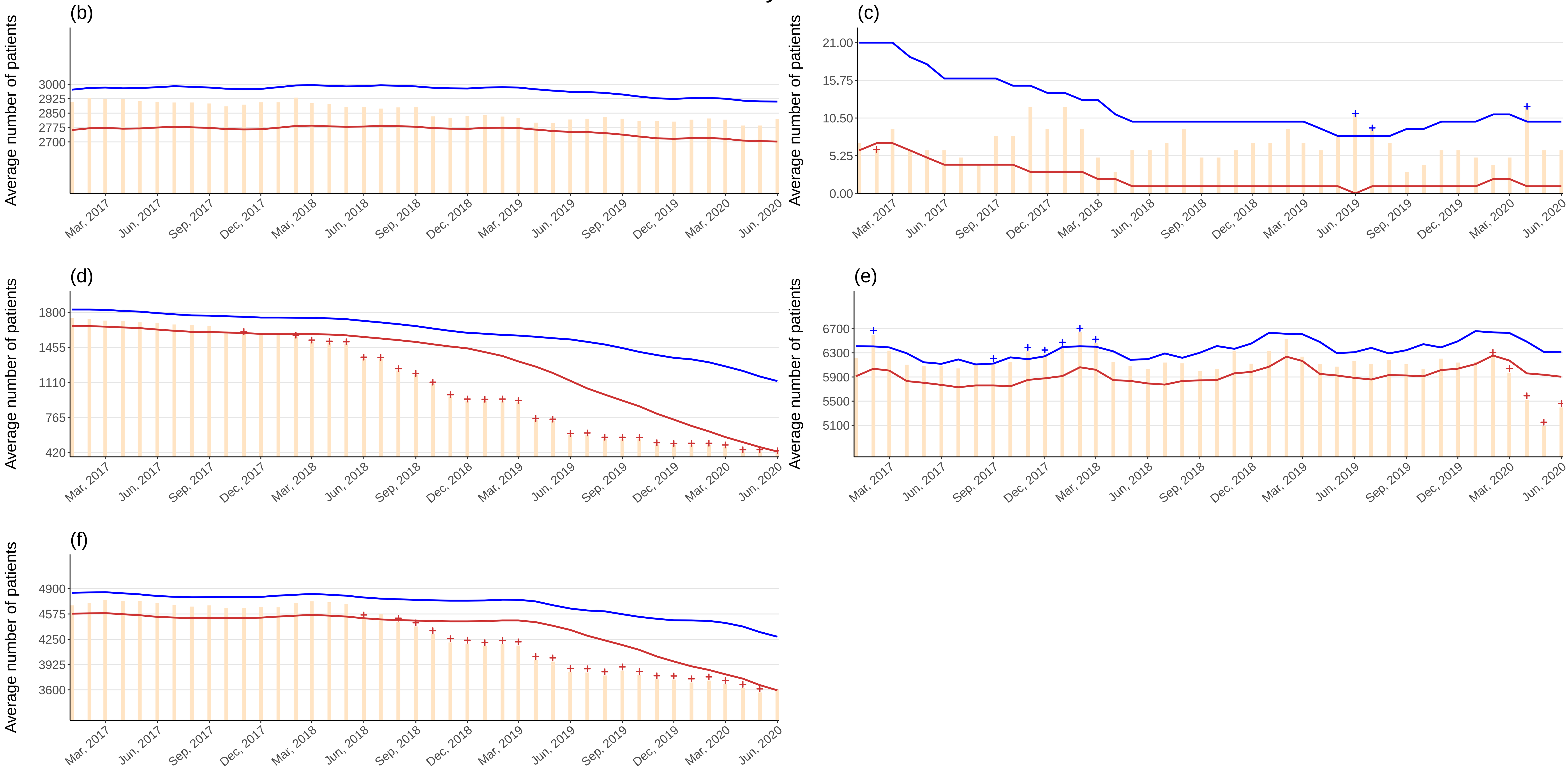

Ishikawa

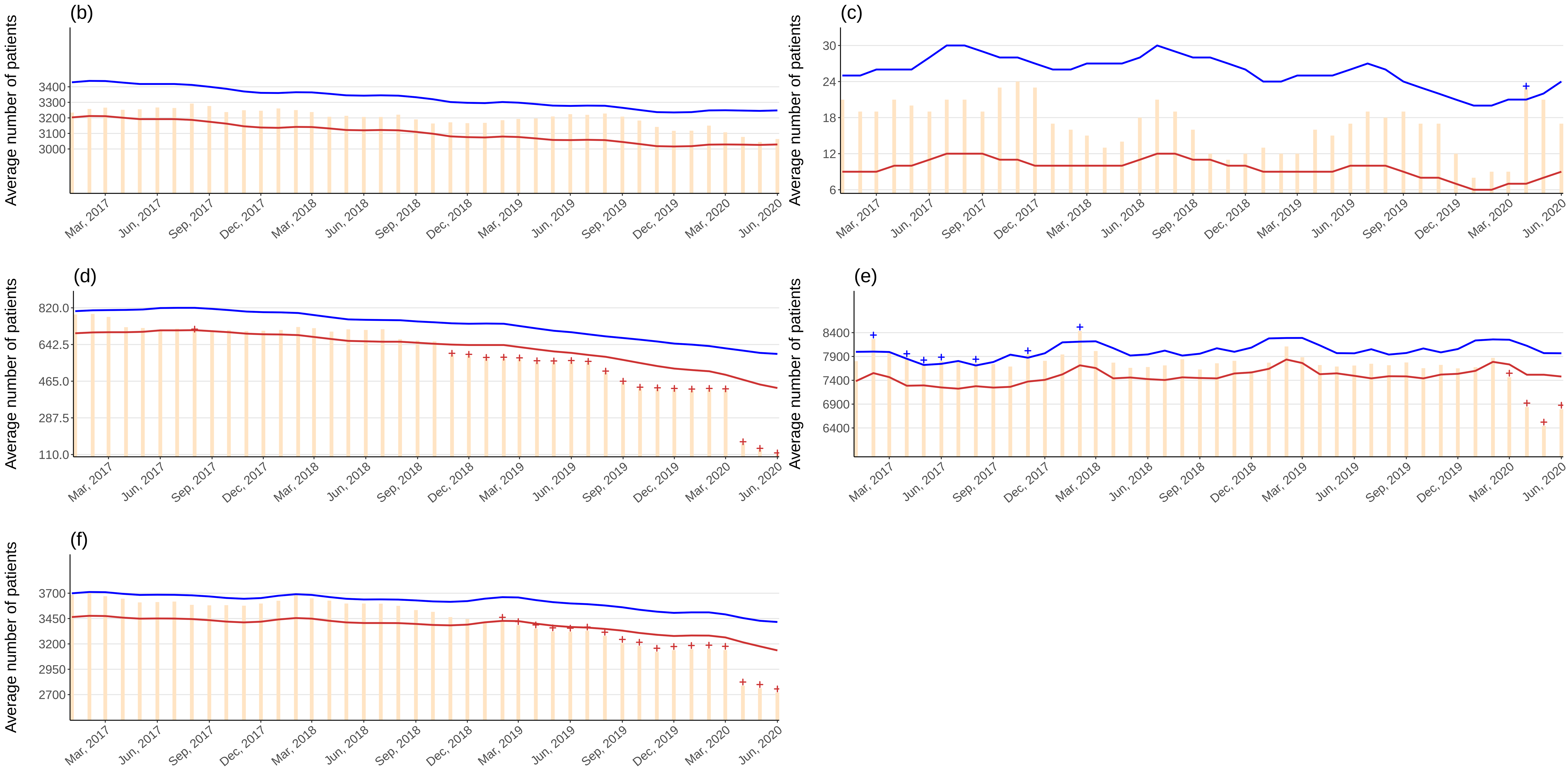

Fukui

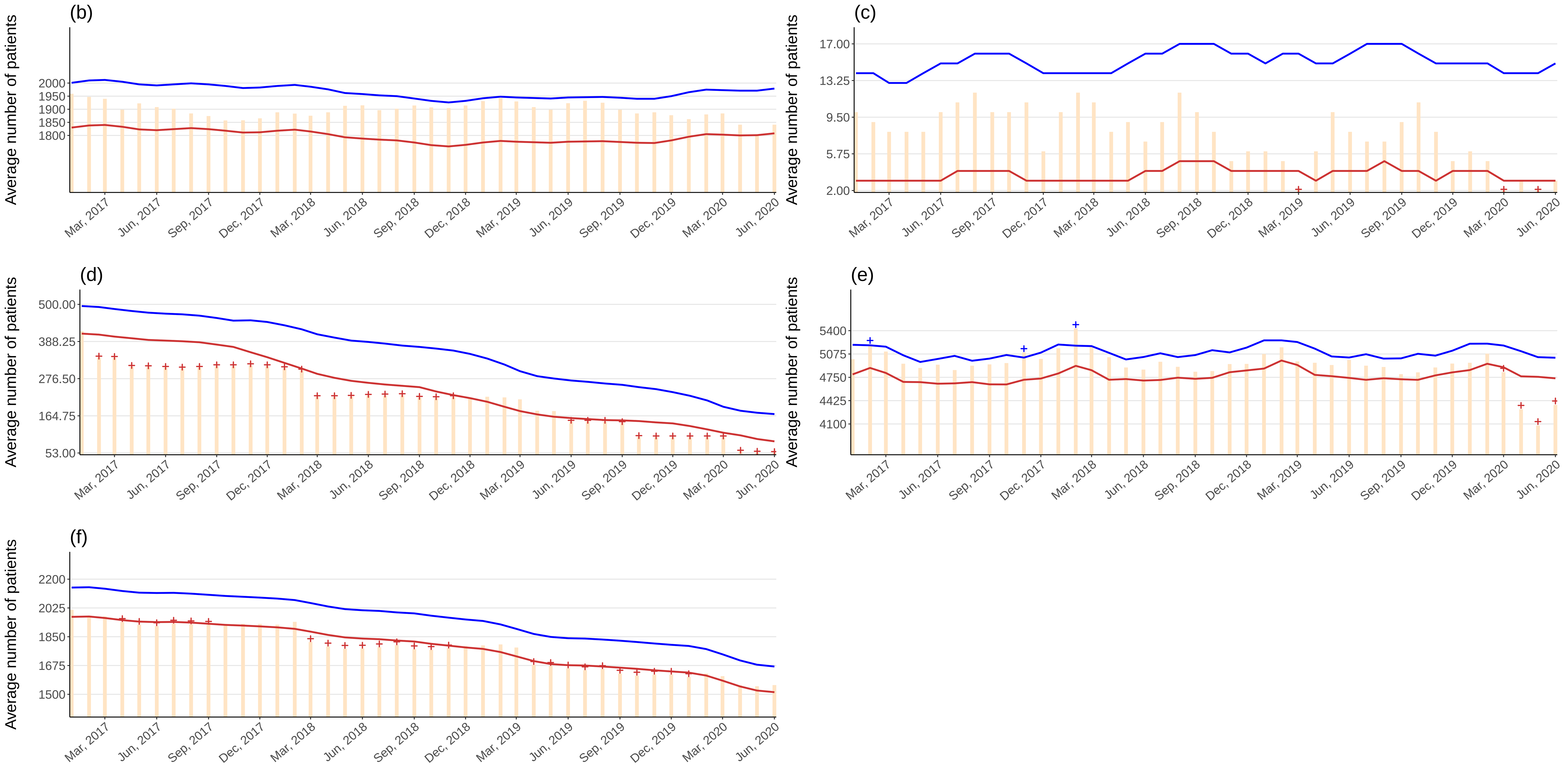

Yamanashi

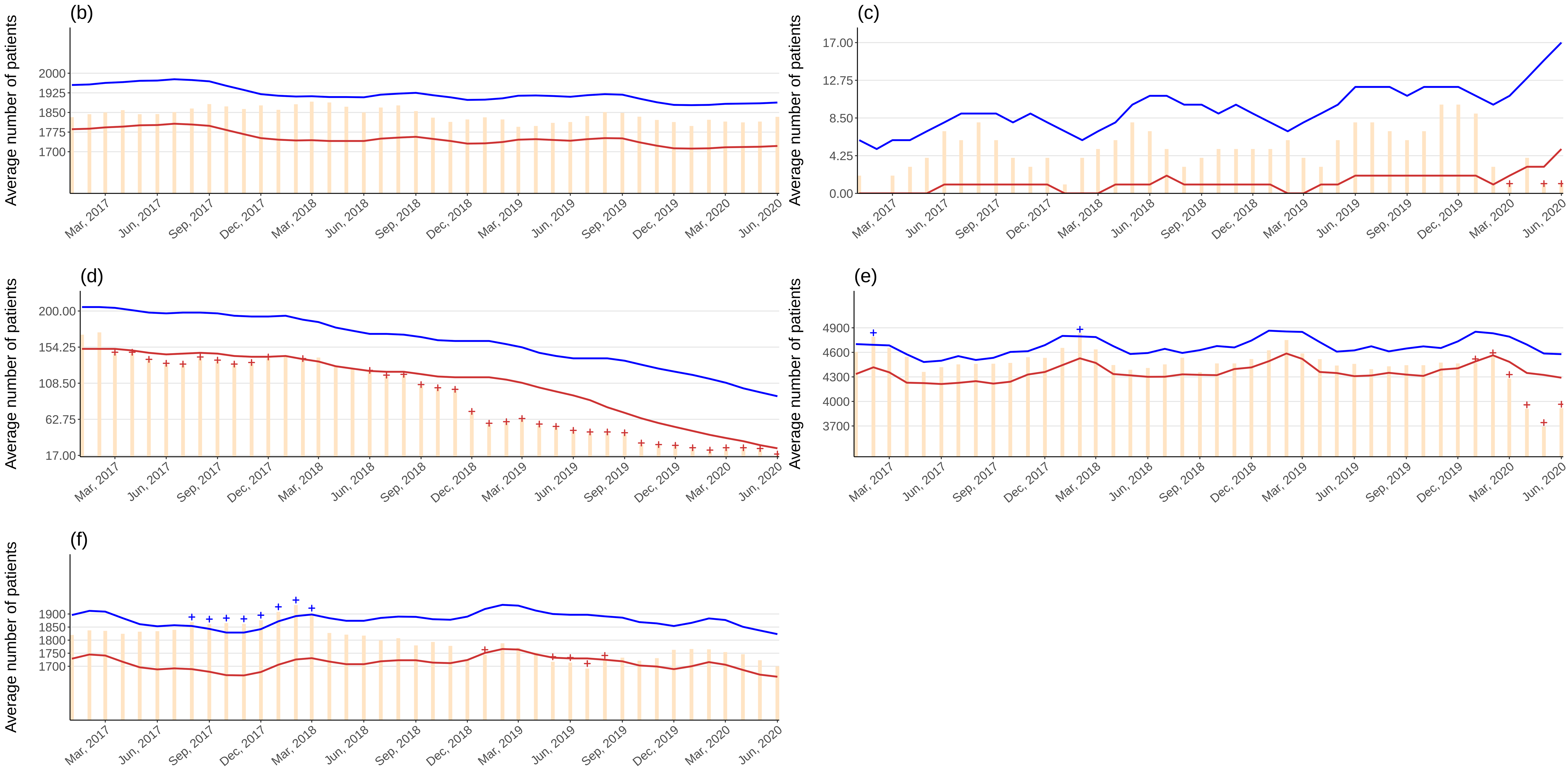

Nagano

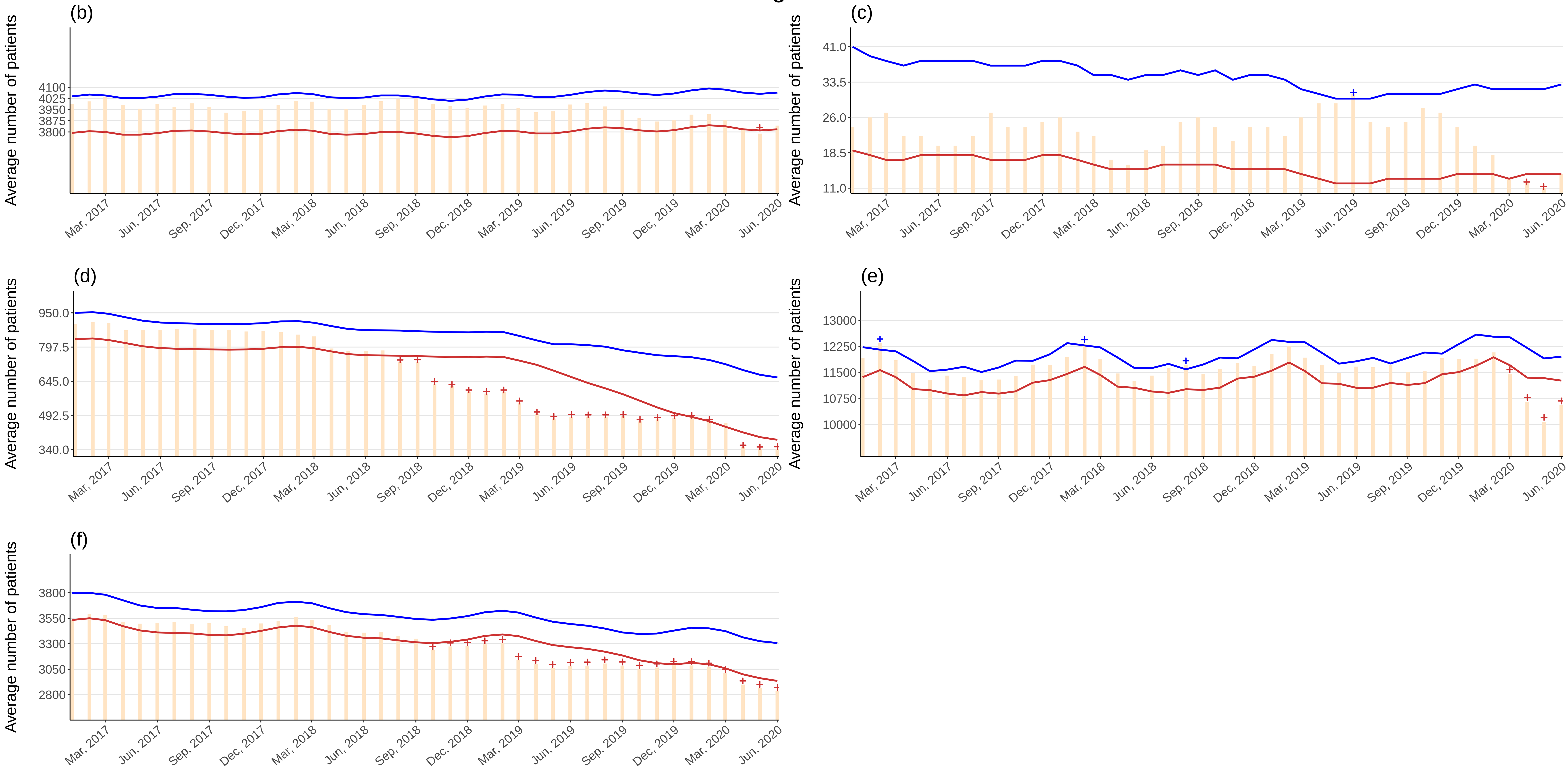

Gifu

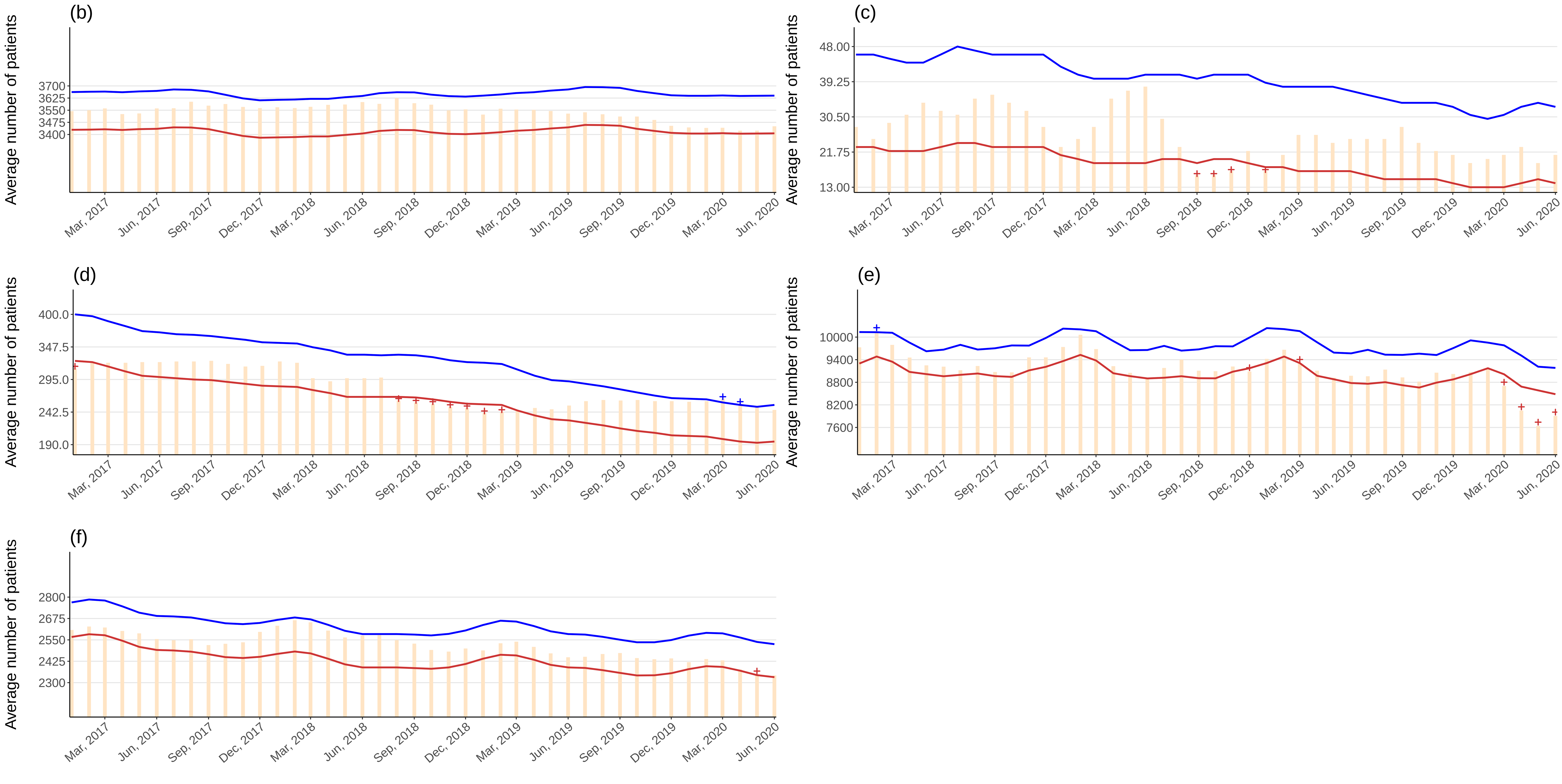

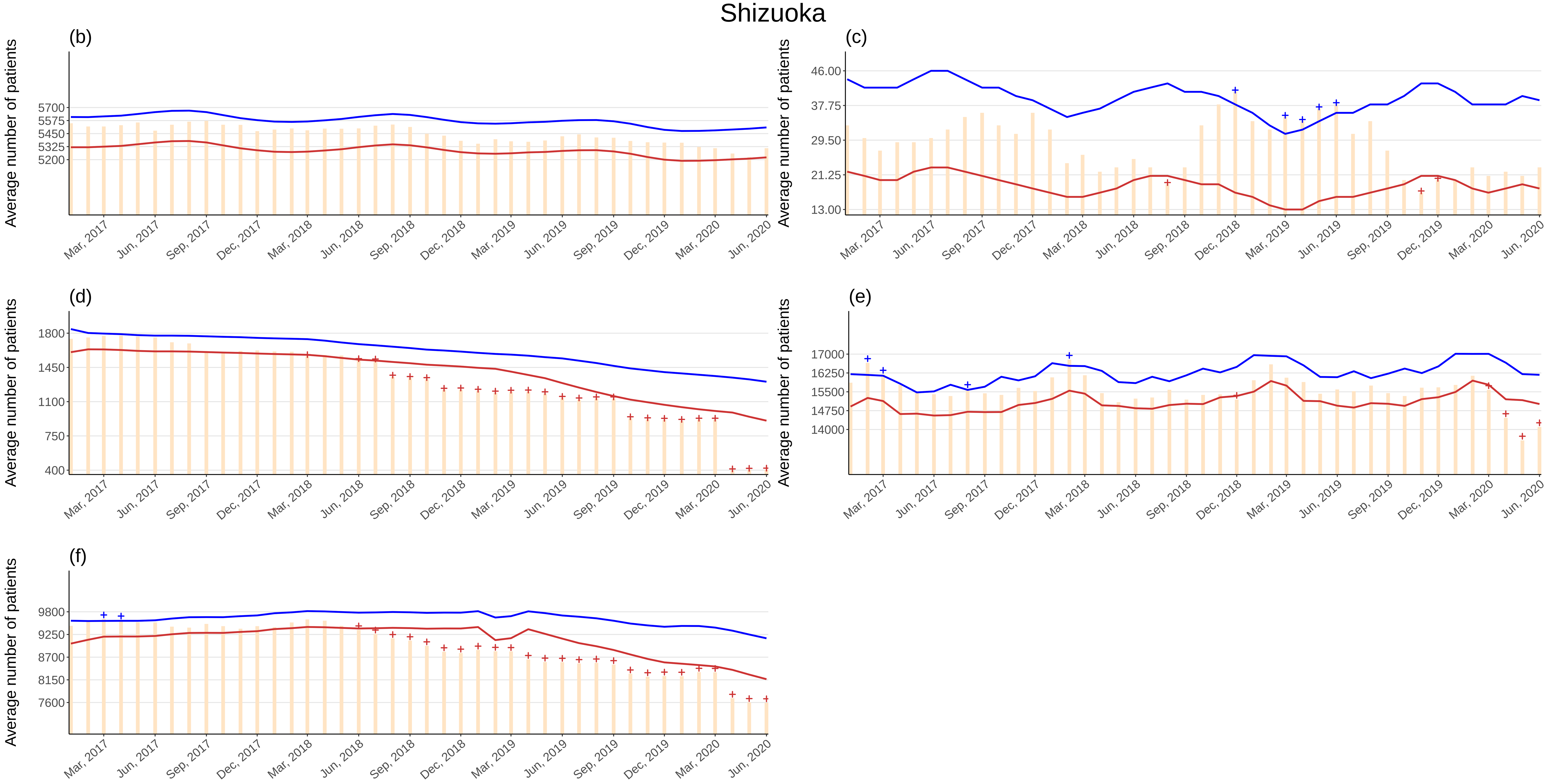
Shizuoka
(b)
5700
5575
5450
5325
5200
(c)
46.00
37.75
29.50
21.25
13.00
(d)
1800
1450
1100
750
400
(e)
17000
16250
15500
14750
14000
(f)
9800
9250
8700
8150
7600
Average number of patients
Mar, 2017
Jun, 2017
Sep, 2017
Dec, 2017
Mar, 2018
Jun, 2018
Sep, 2018
Dec, 2018
Mar, 2019
Jun, 2019
Sep, 2019
Dec, 2019
Mar, 2020
Jun, 2020

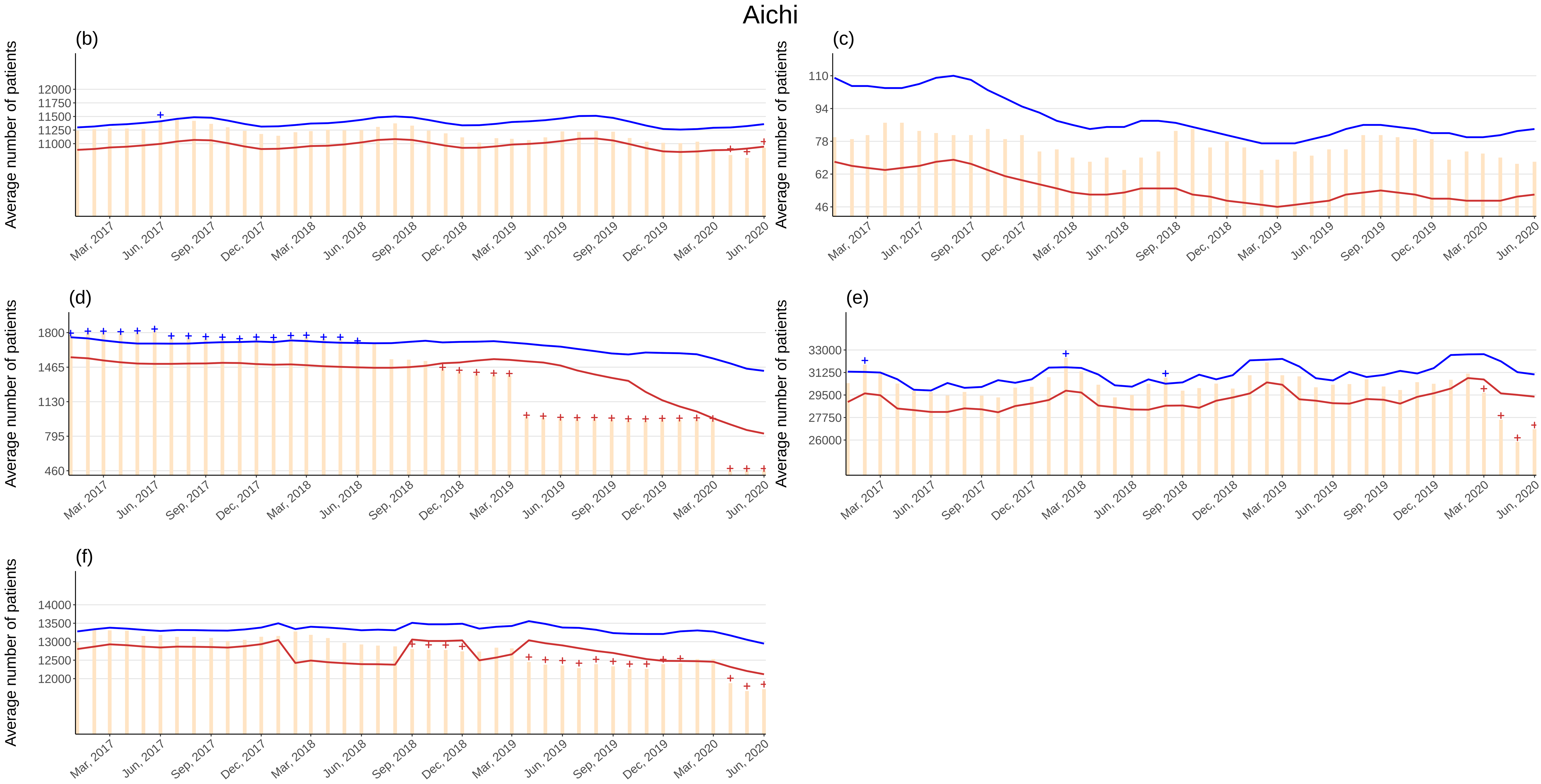
Aichi
(b)
12000
11750
11500
11250
11000
(c)
110
94
78
62
46
(d)
1800
1465
1130
795
460
(e)
33000
31250
29500
27750
26000
(f)
14000
13500
13000
12500
12000
Average number of patients
Mar, 2017
Jun, 2017
Sep, 2017
Dec, 2017
Mar, 2018
Jun, 2018
Sep, 2018
Dec, 2018
Mar, 2019
Jun, 2019
Sep, 2019
Dec, 2019
Mar, 2020
Jun, 2020

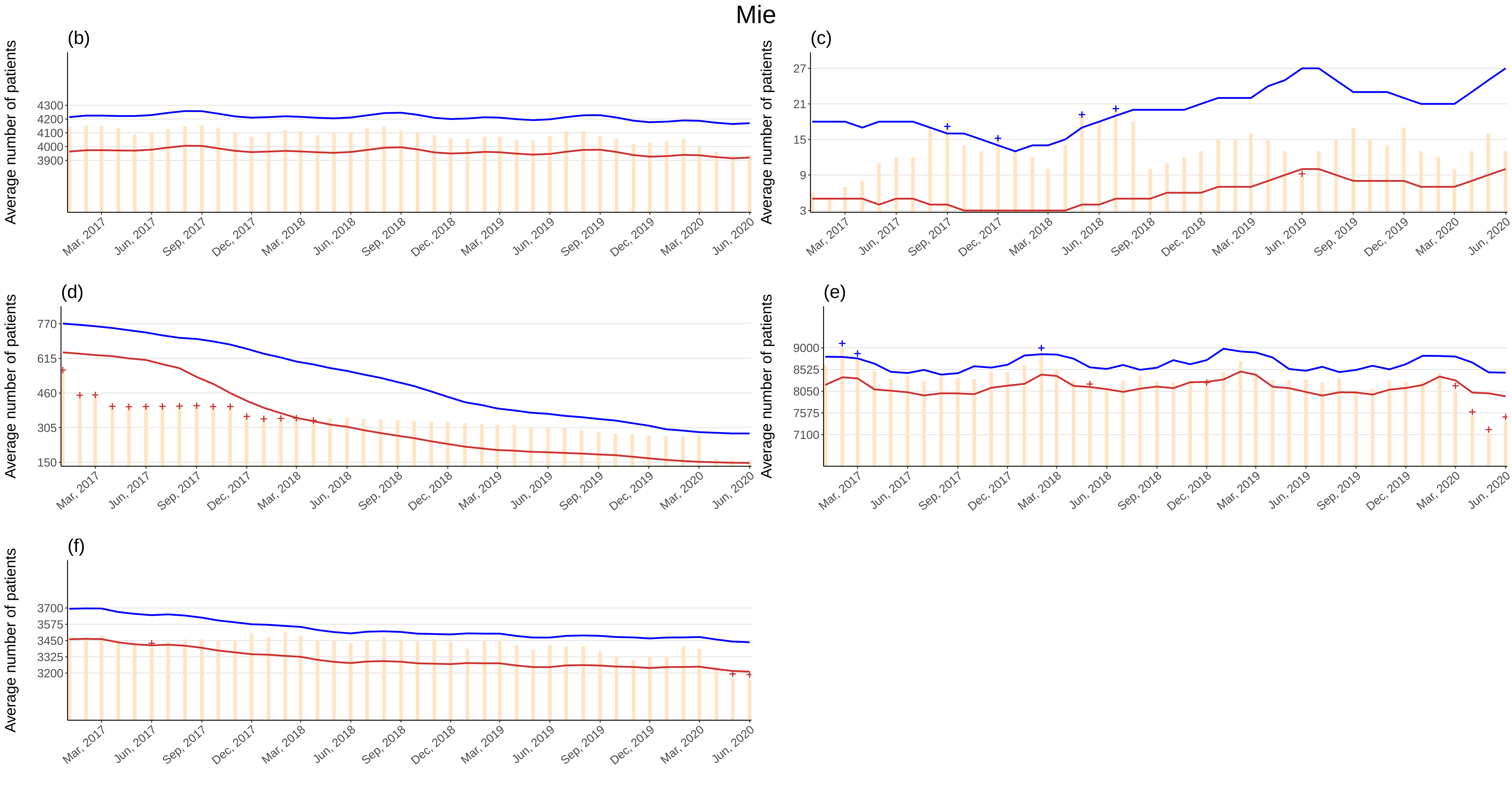
Mie
(b)
4300
4200
4100
4000
3900
(c)
27
21
15
9
3
(d)
770
615
460
305
150
(e)
9000
8525
8050
7575
7100
(f)
3700
3575
3450
3325
3200
Average number of patients
Mar, 2017
Jun, 2017
Sep, 2017
Dec, 2017
Mar, 2018
Jun, 2018
Sep, 2018
Dec, 2018
Mar, 2019
Jun, 2019
Sep, 2019
Dec, 2019
Mar, 2020
Jun, 2020

Shiga

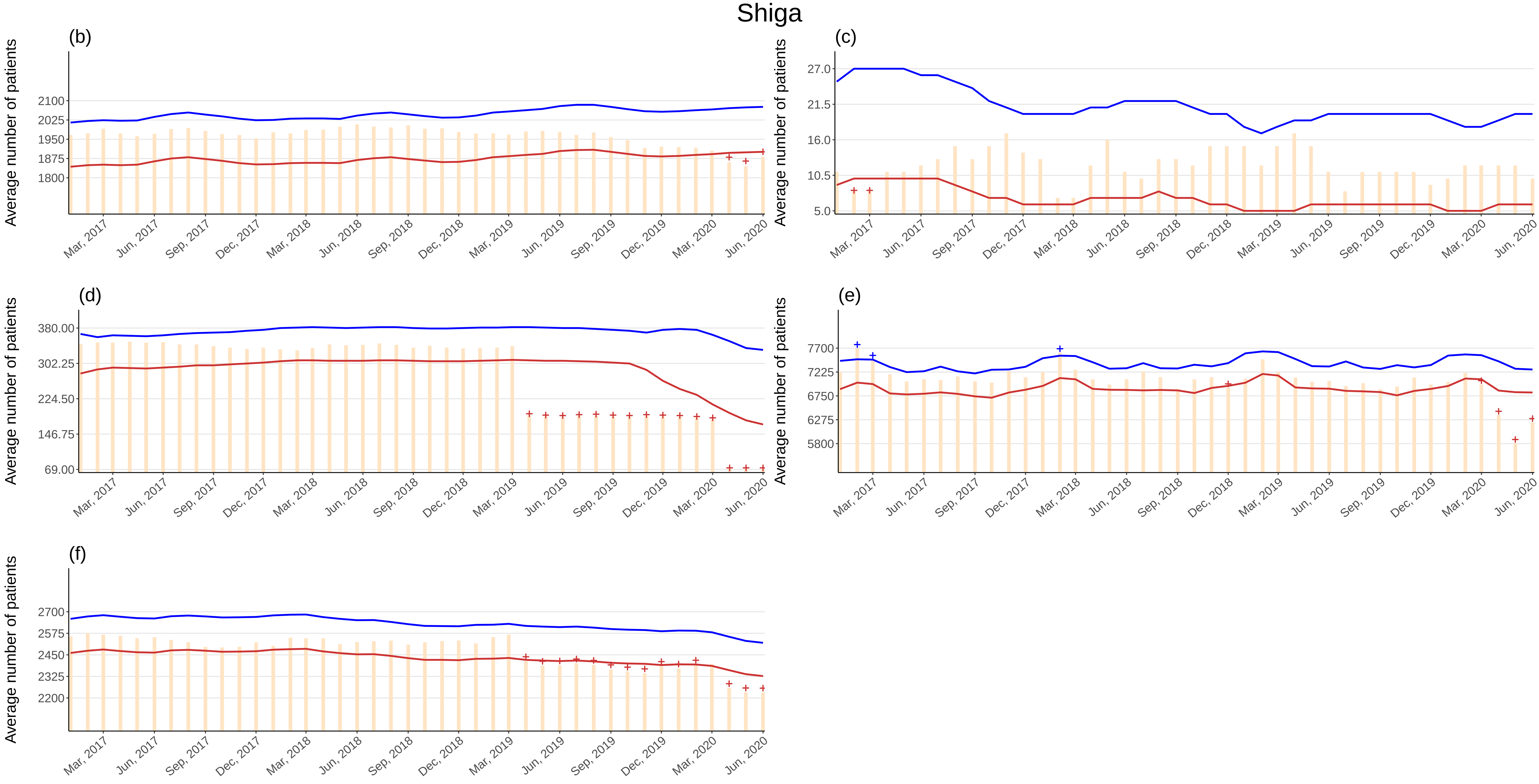

Kyoto

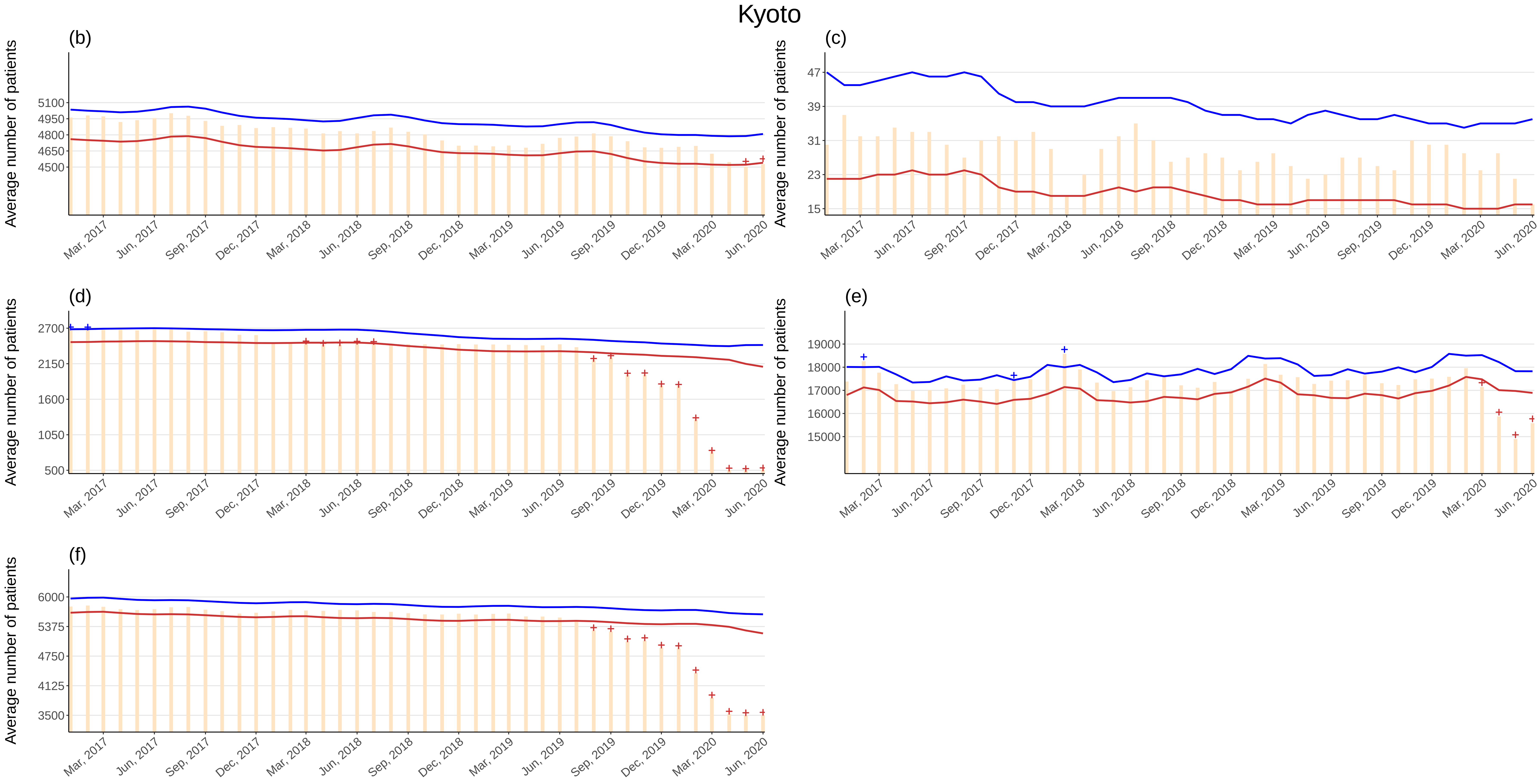

Osaka

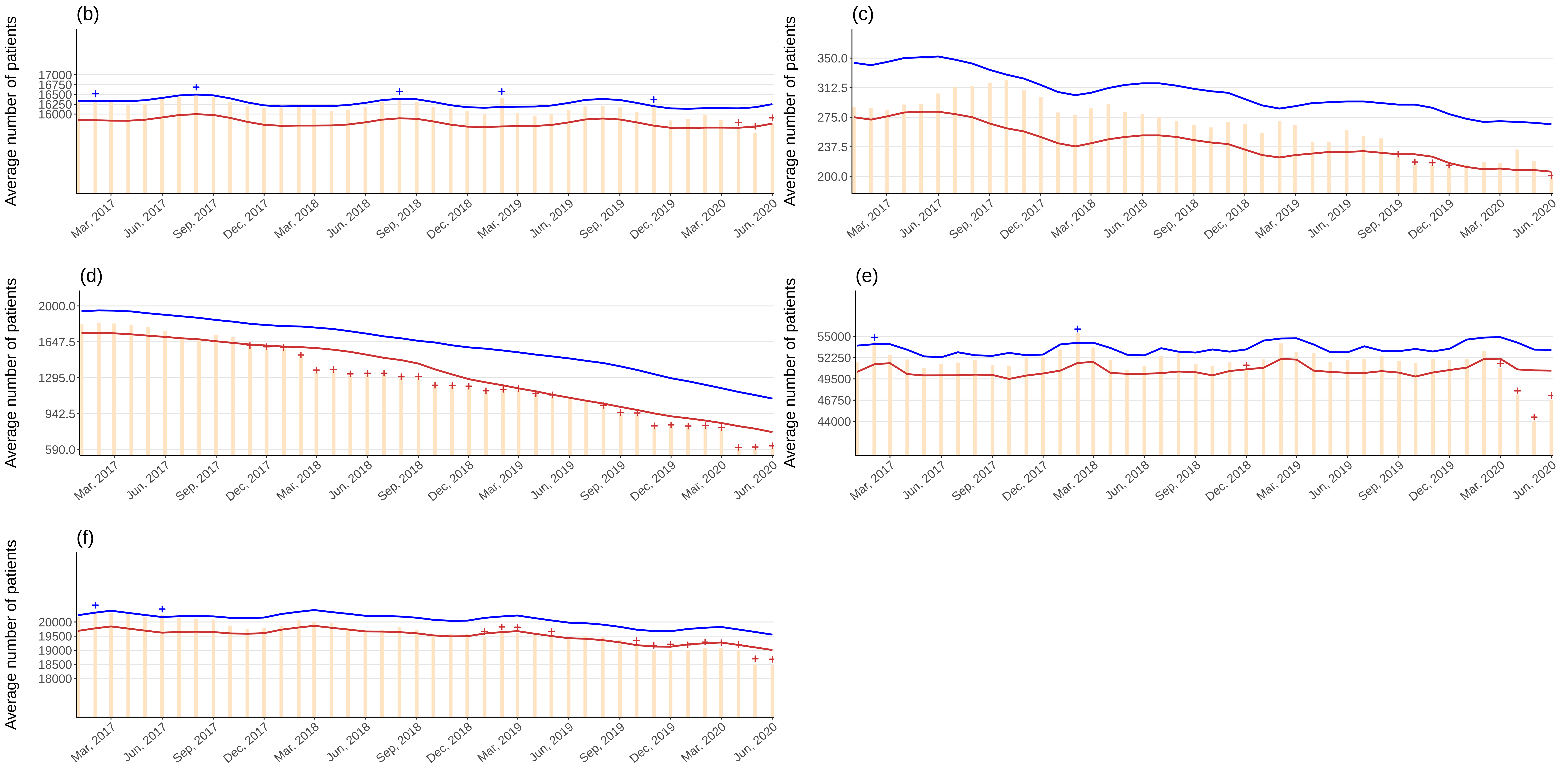

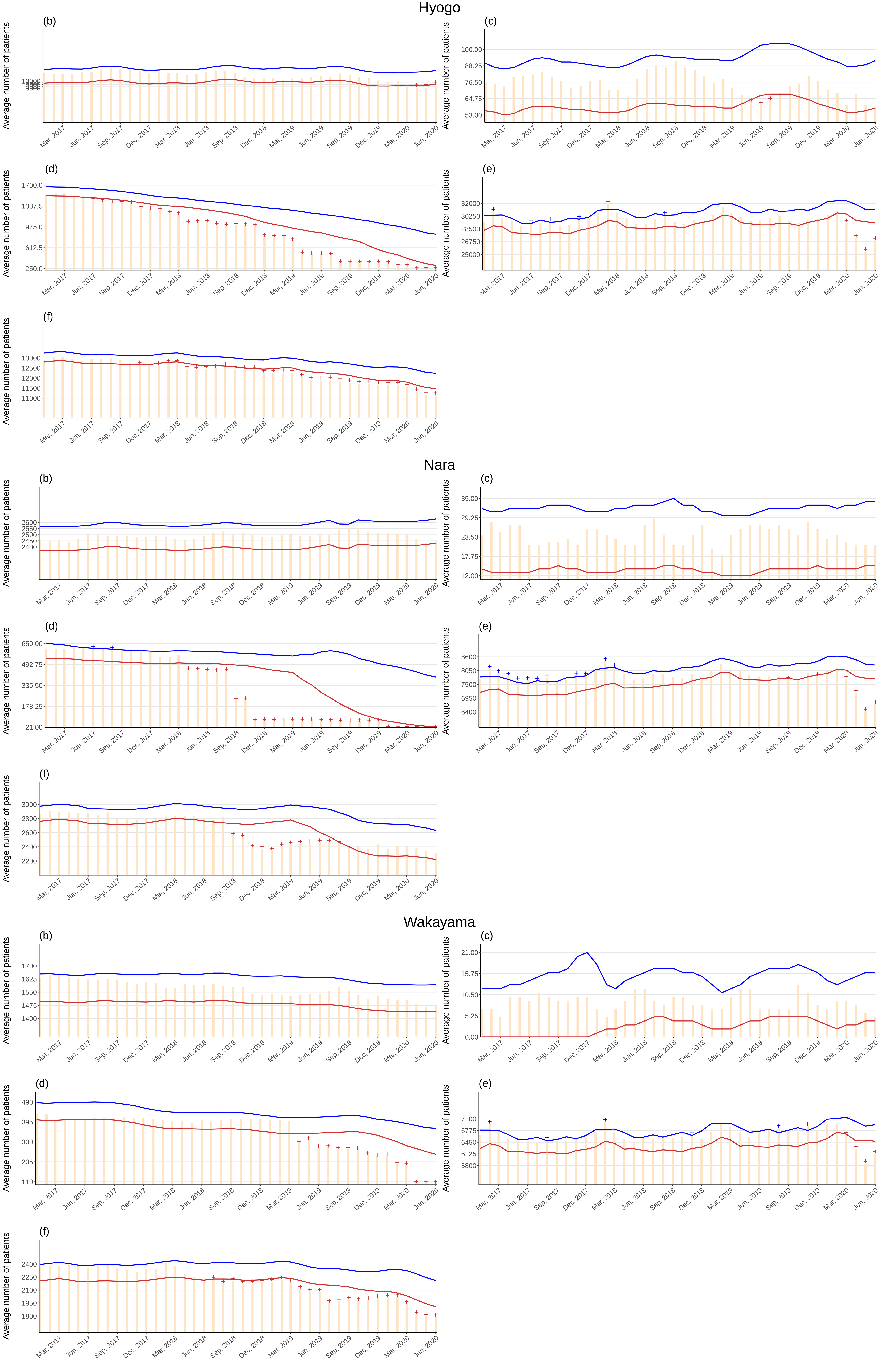
Hyogo
(b)
(c)
(d)
(e)
(f)
Nara
(b)
(c)
(d)
(e)
(f)
Wakayama
(b)
(c)
(d)
(e)
(f)
Average number of patients
Mar, 2017
Jun, 2017
Sep, 2017
Dec, 2017
Mar, 2018
Jun, 2018
Sep, 2018
Dec, 2018
Mar, 2019
Jun, 2019
Sep, 2019
Dec, 2019
Mar, 2020
Jun, 2020

Tottori

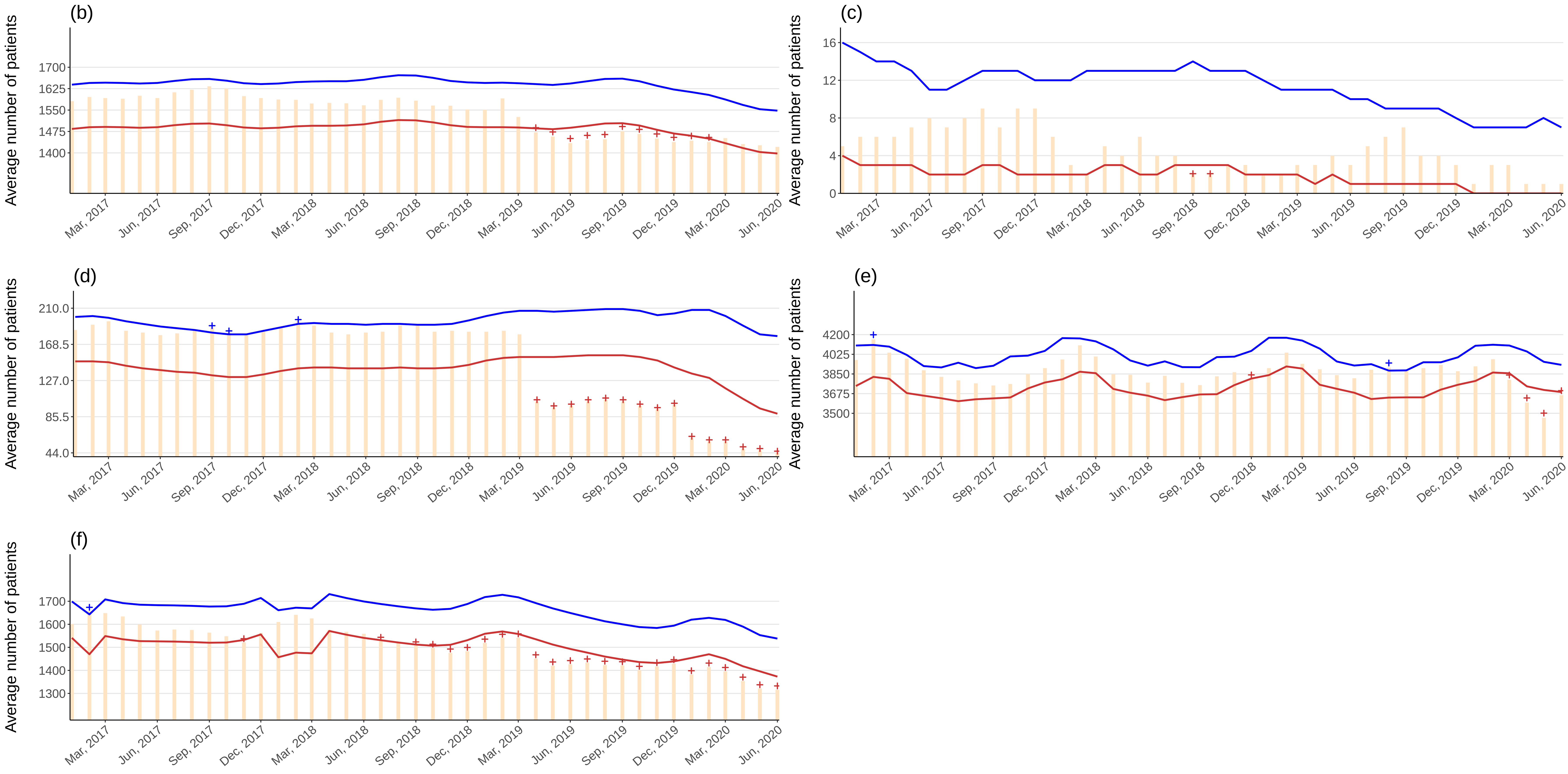

Shimane

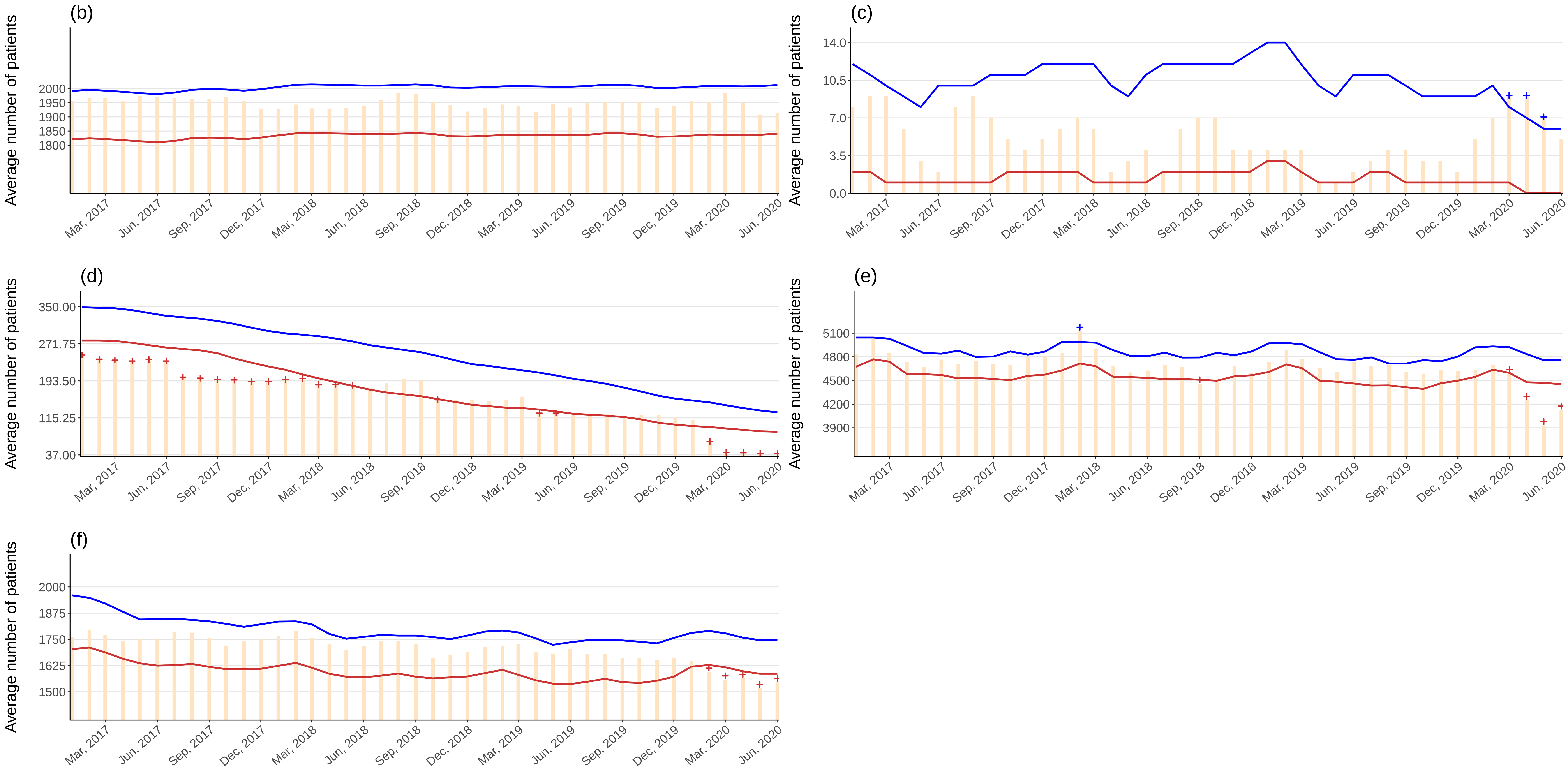

Okayama

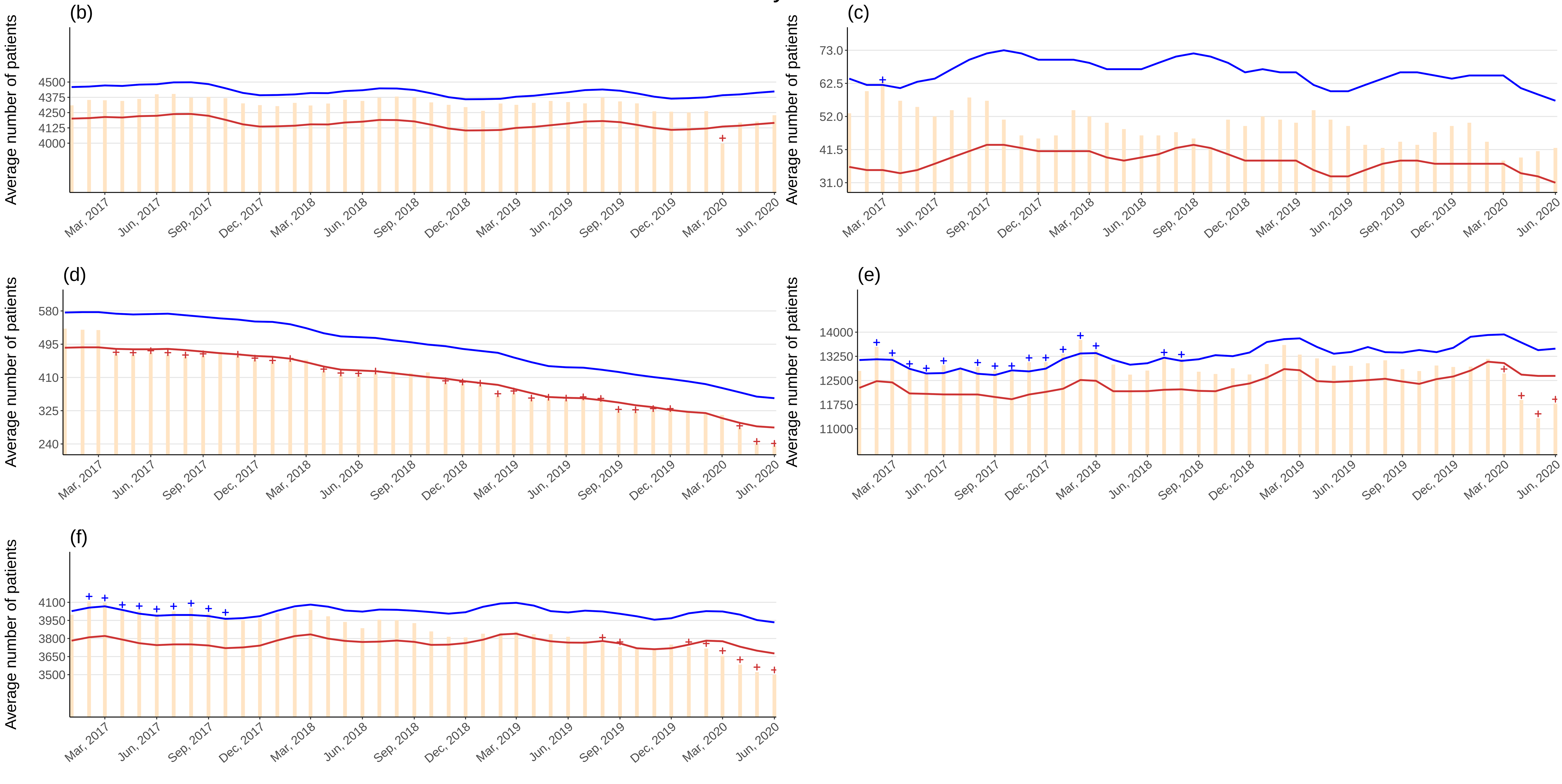

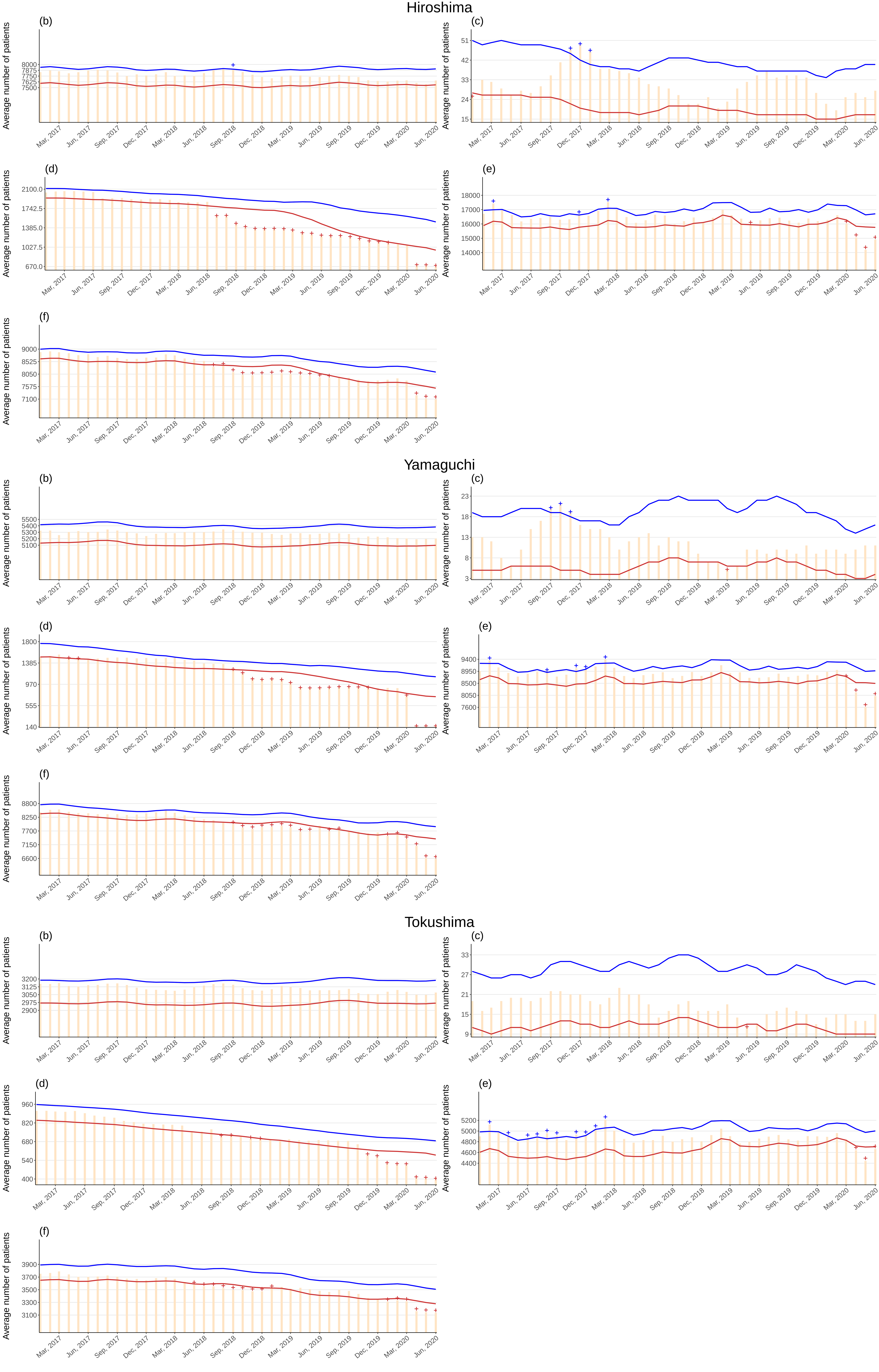

Hiroshima
(b)
(c)
(d)
(e)
(f)
Yamaguchi
(b)
(c)
(d)
(e)
(f)
Tokushima
(b)
(c)
(d)
(e)
(f)
Average number of patients
Mar, 2017
Jun, 2017
Sep, 2017
Dec, 2017
Mar, 2018
Jun, 2018
Sep, 2018
Dec, 2018
Mar, 2019
Jun, 2019
Sep, 2019
Dec, 2019
Mar, 2020
Jun, 2020

Kagawa

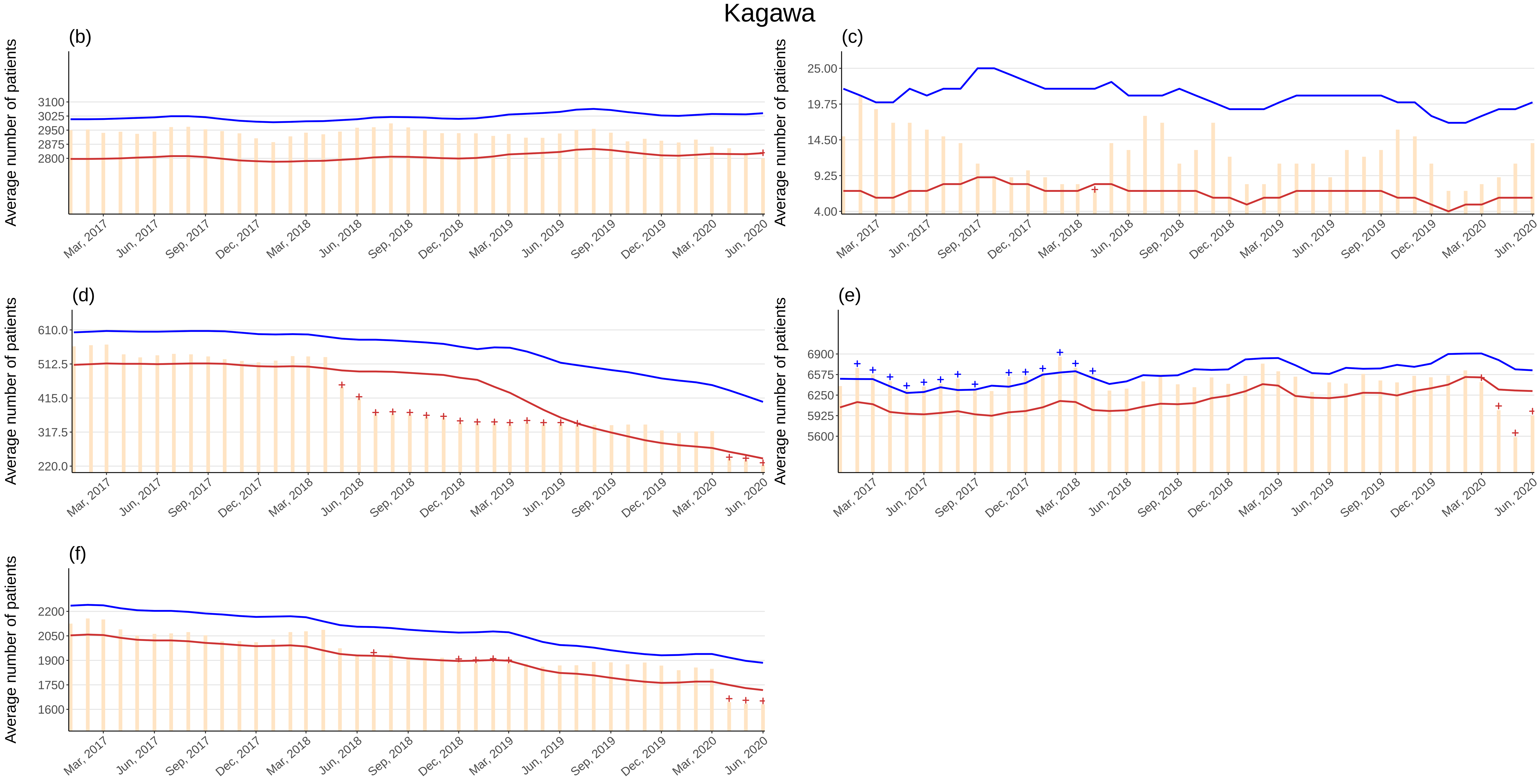

Ehime

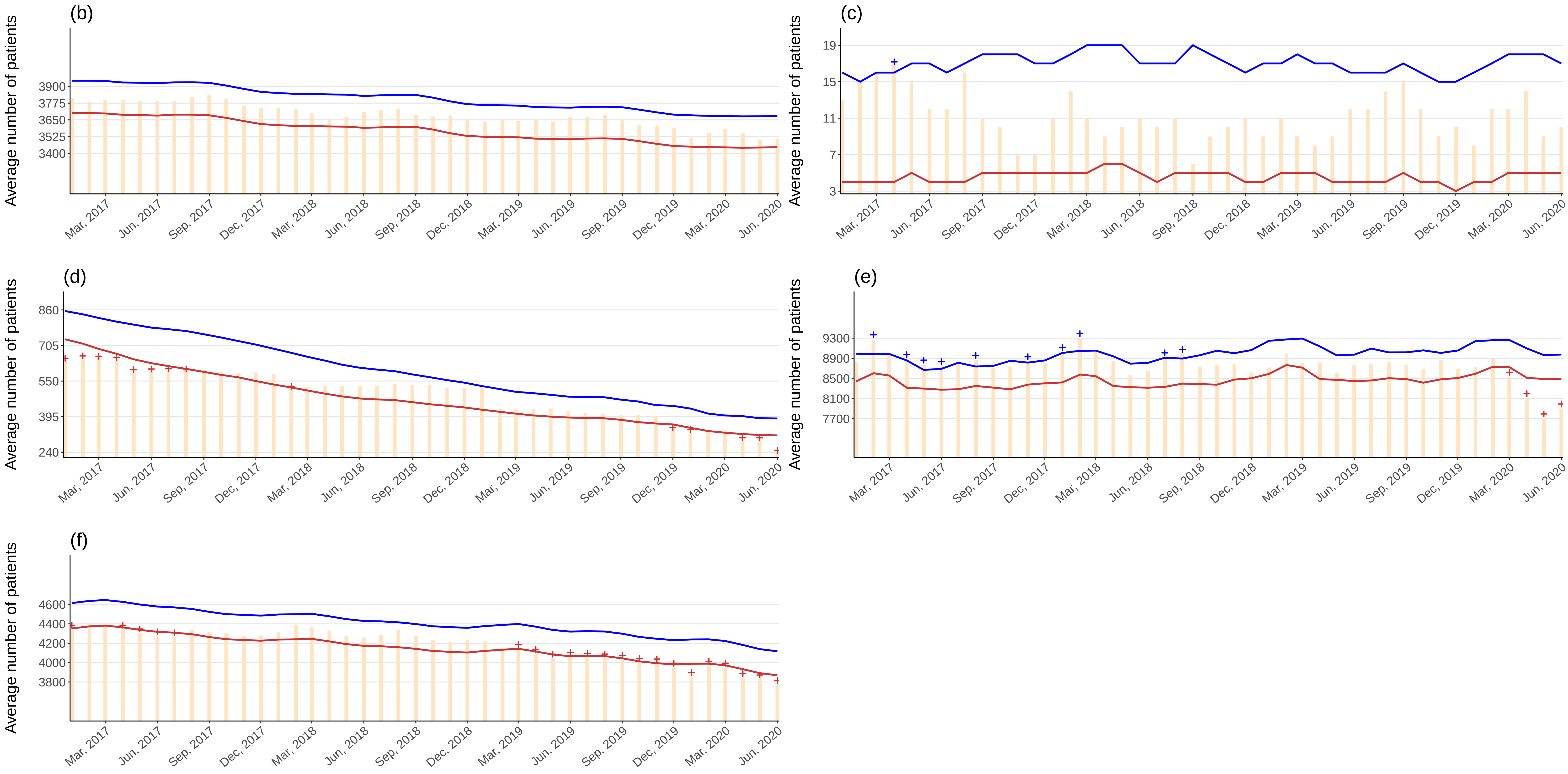

Kochi

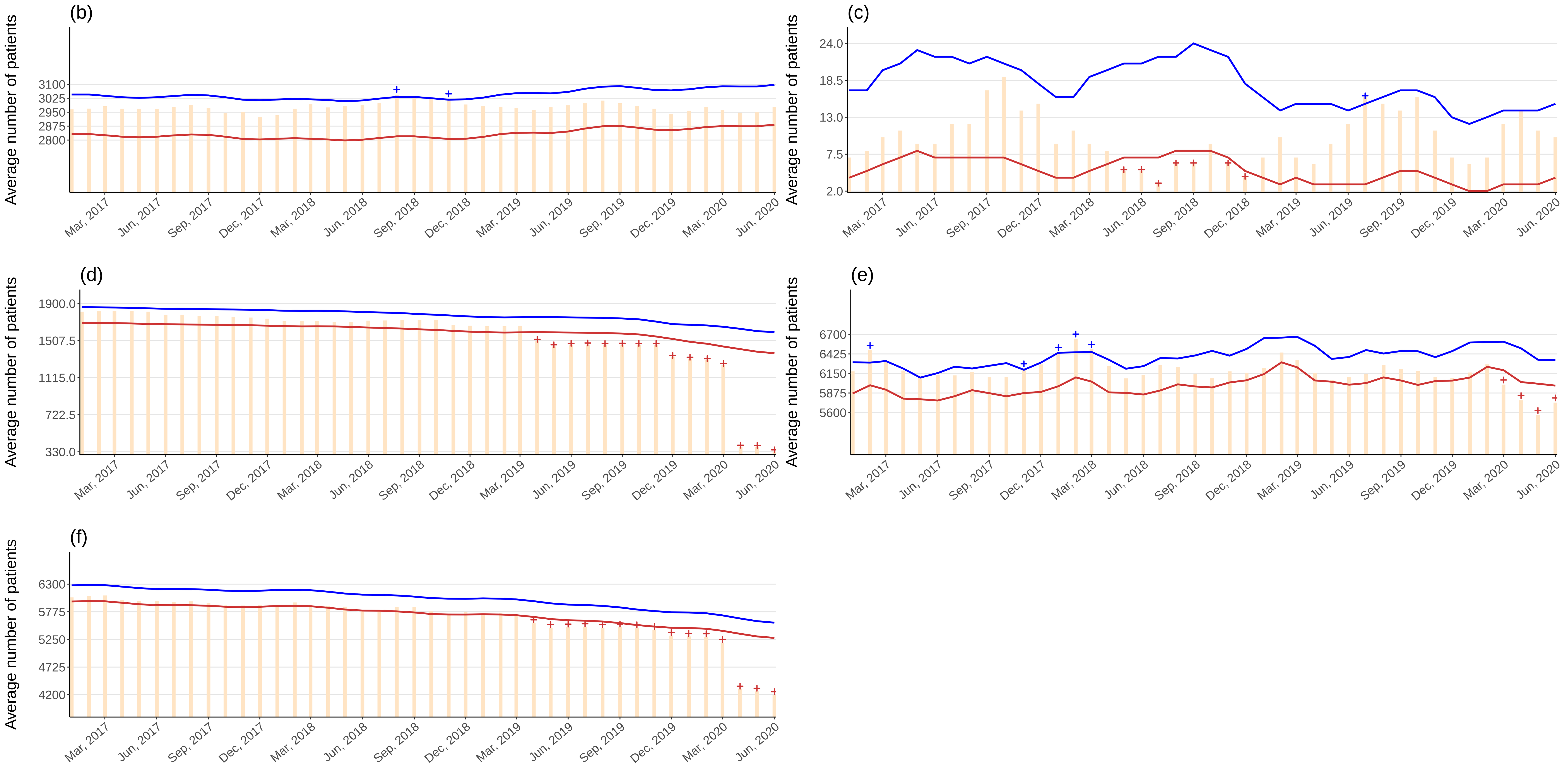

Fukuoka

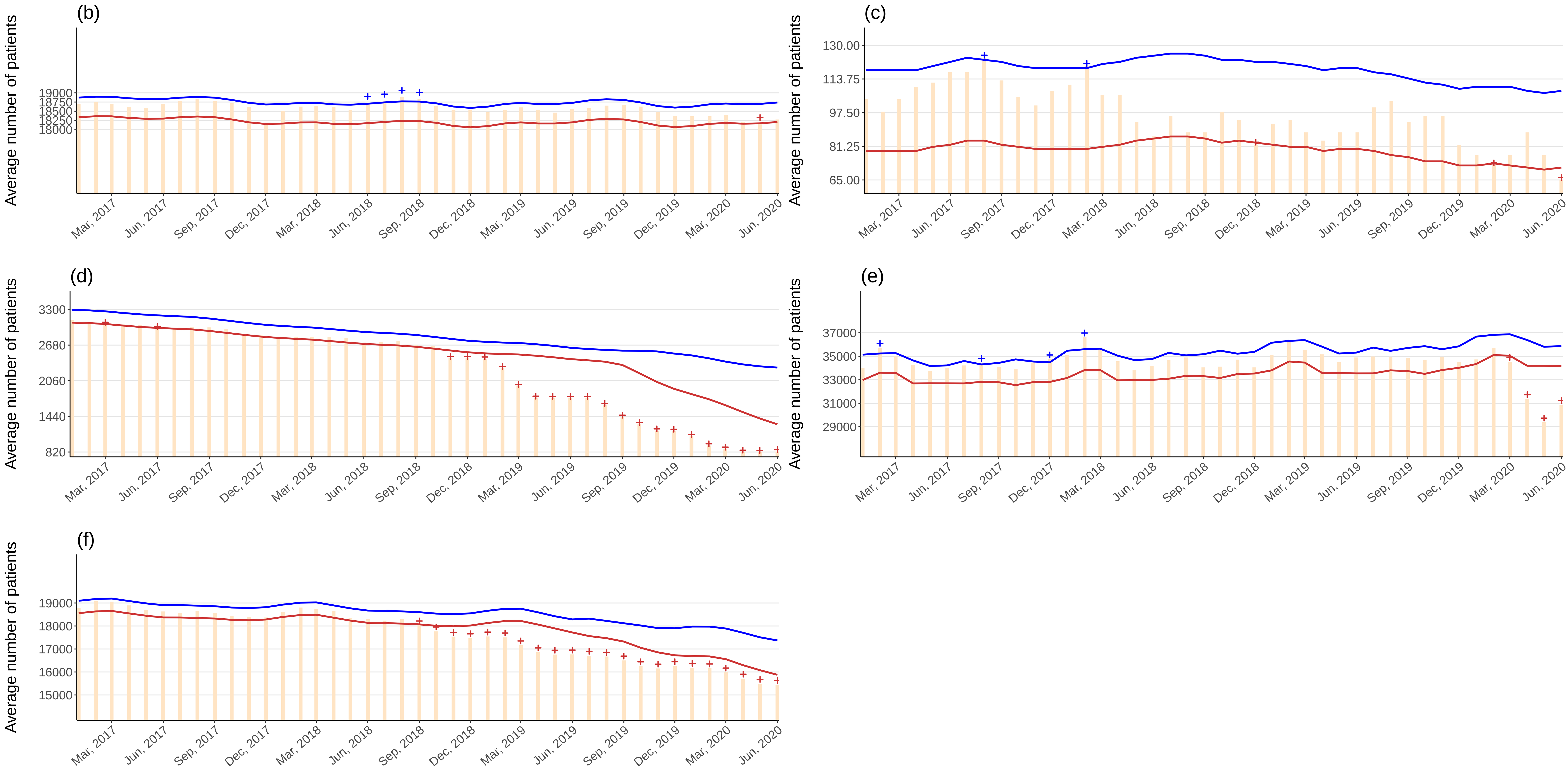

Saga

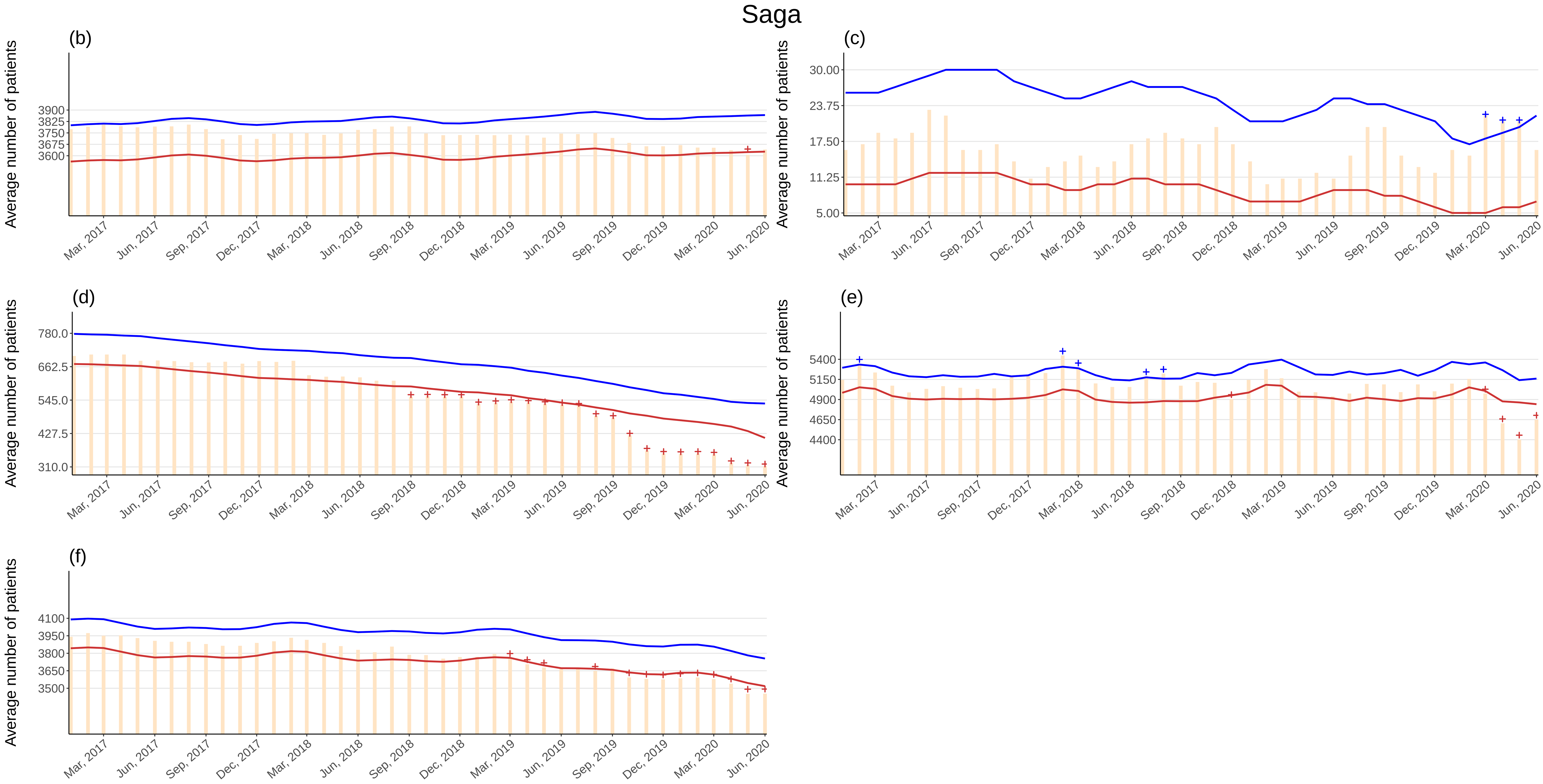

Nagasaki

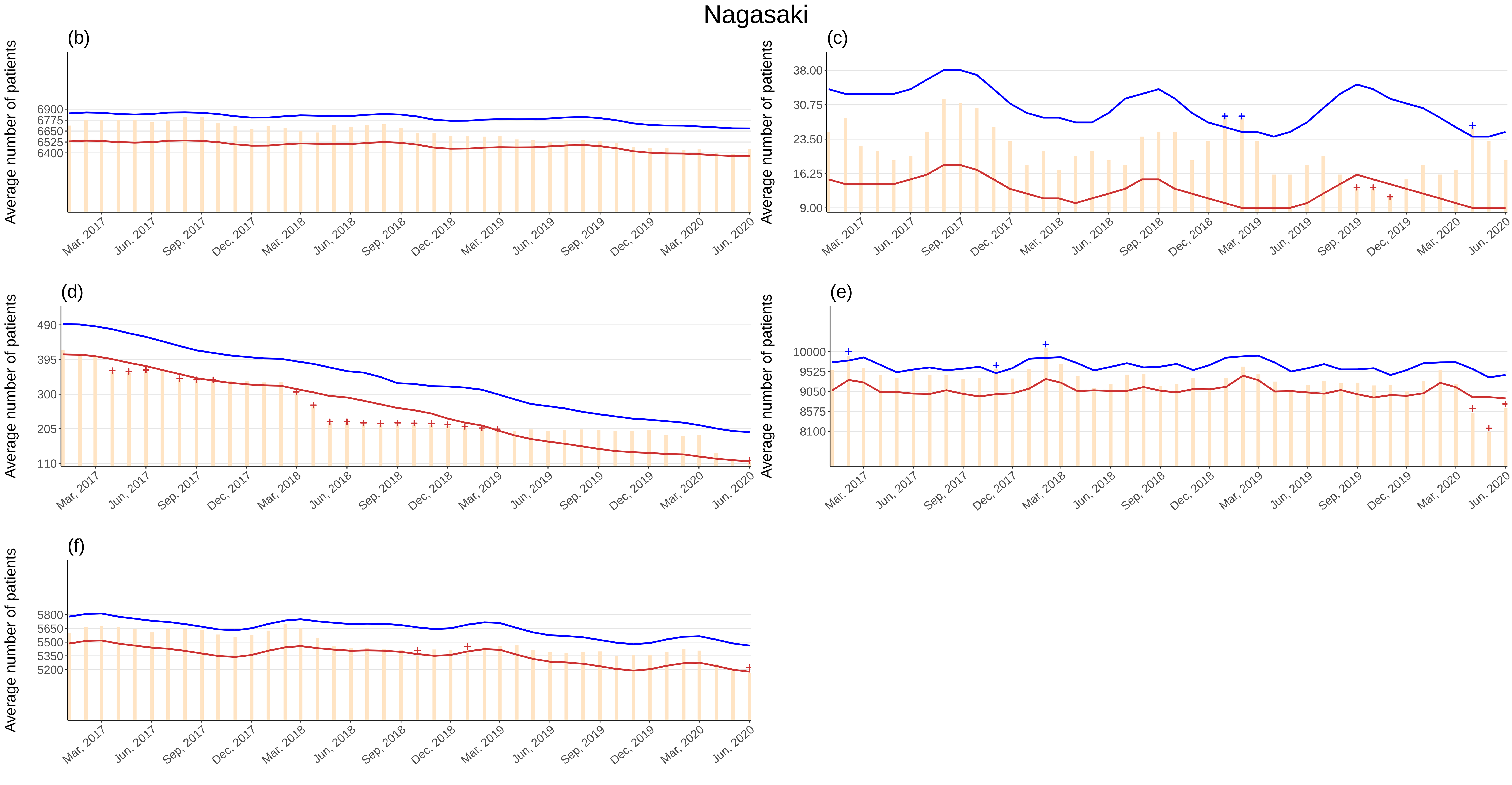

Kumamoto

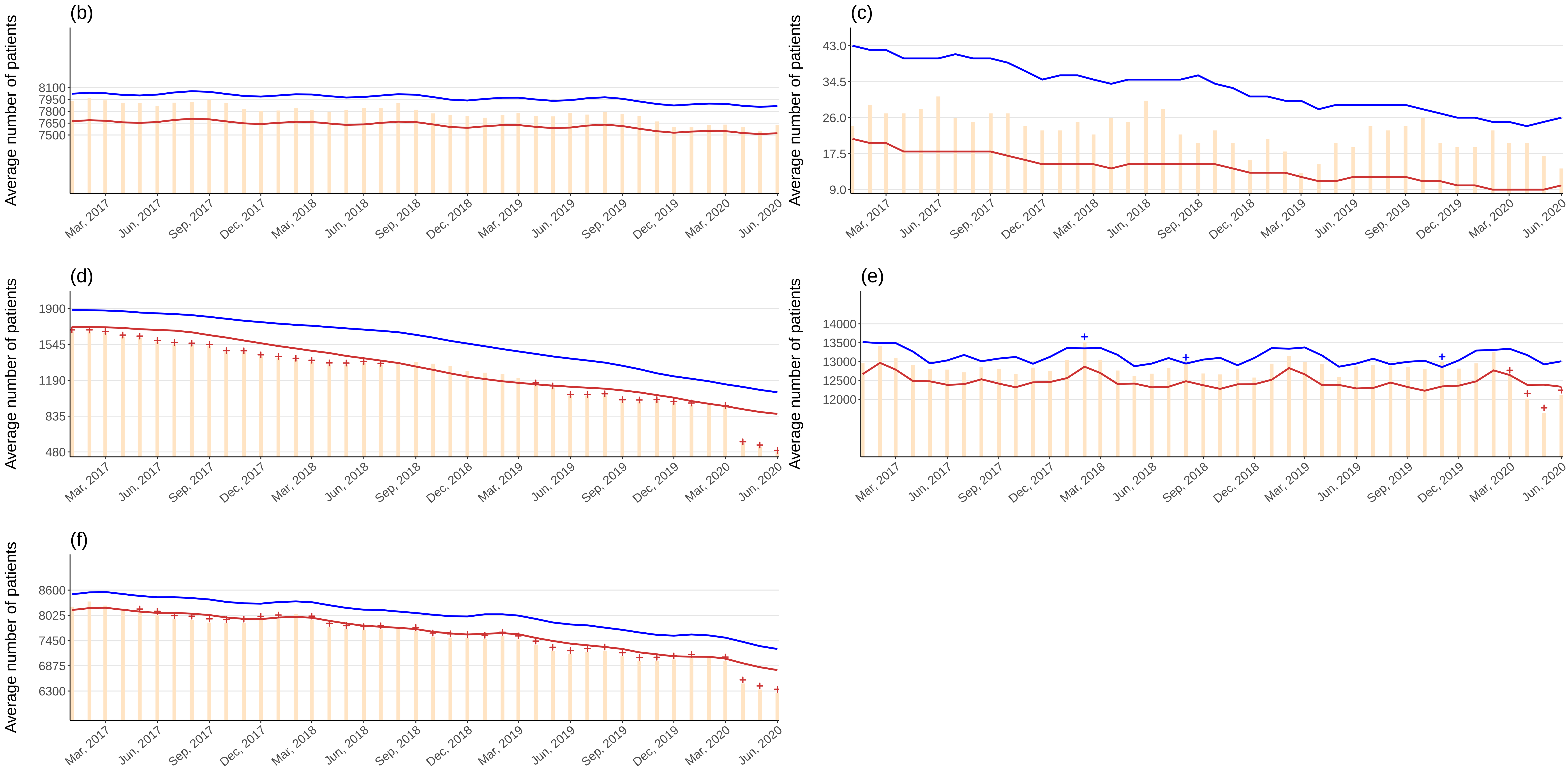

Oita

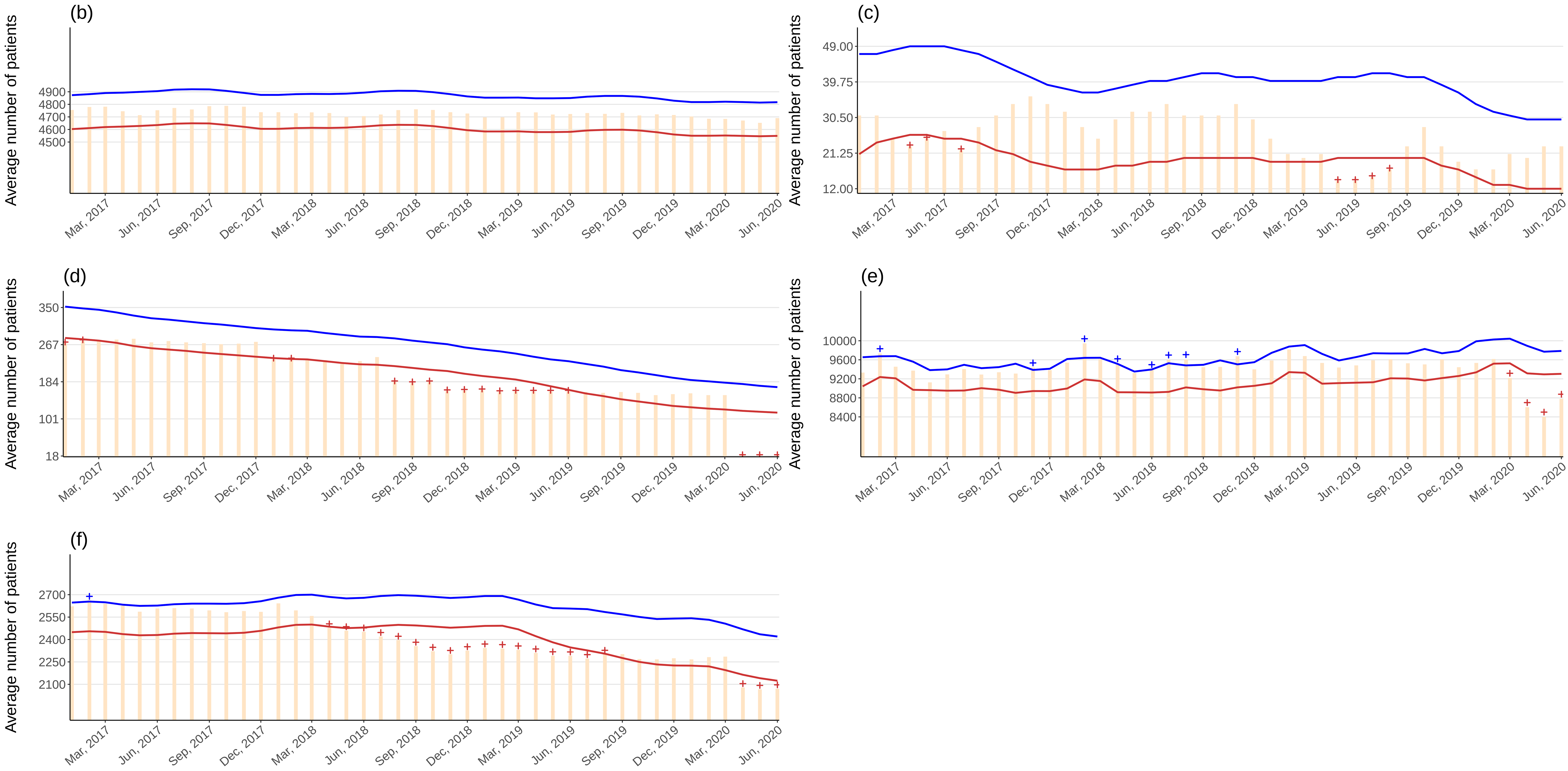

Miyazaki

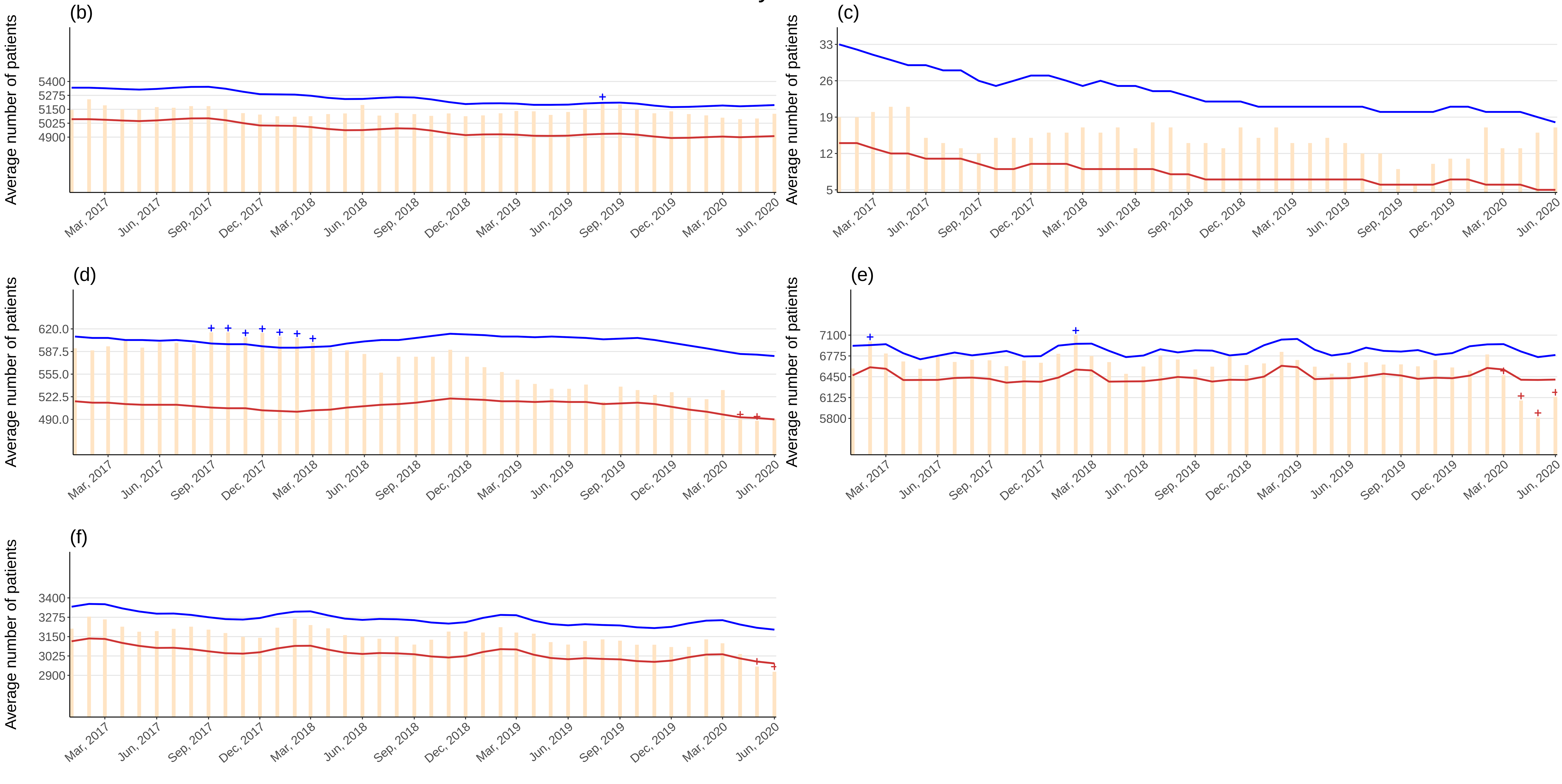

Kagoshima

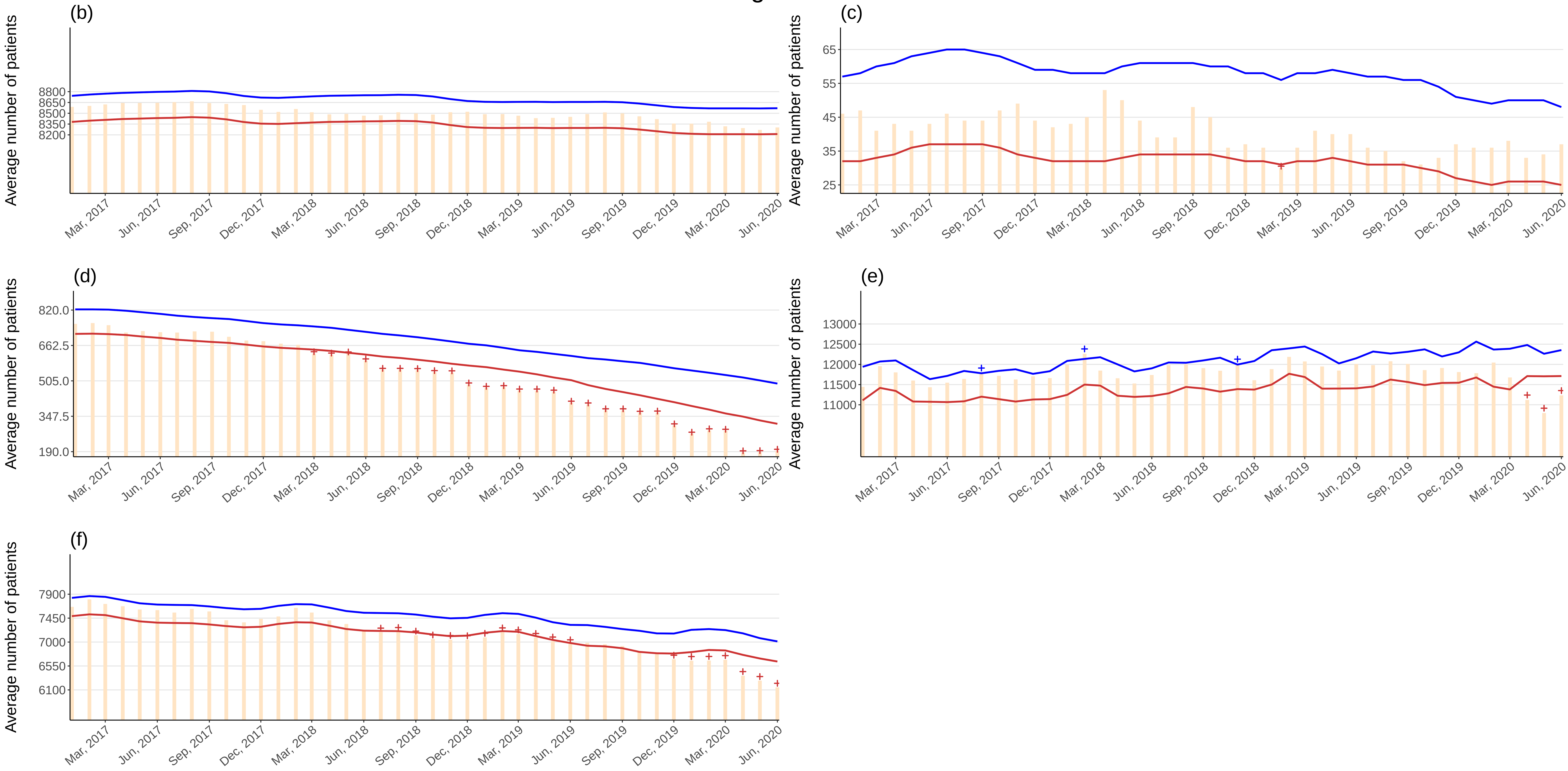

Okinawa

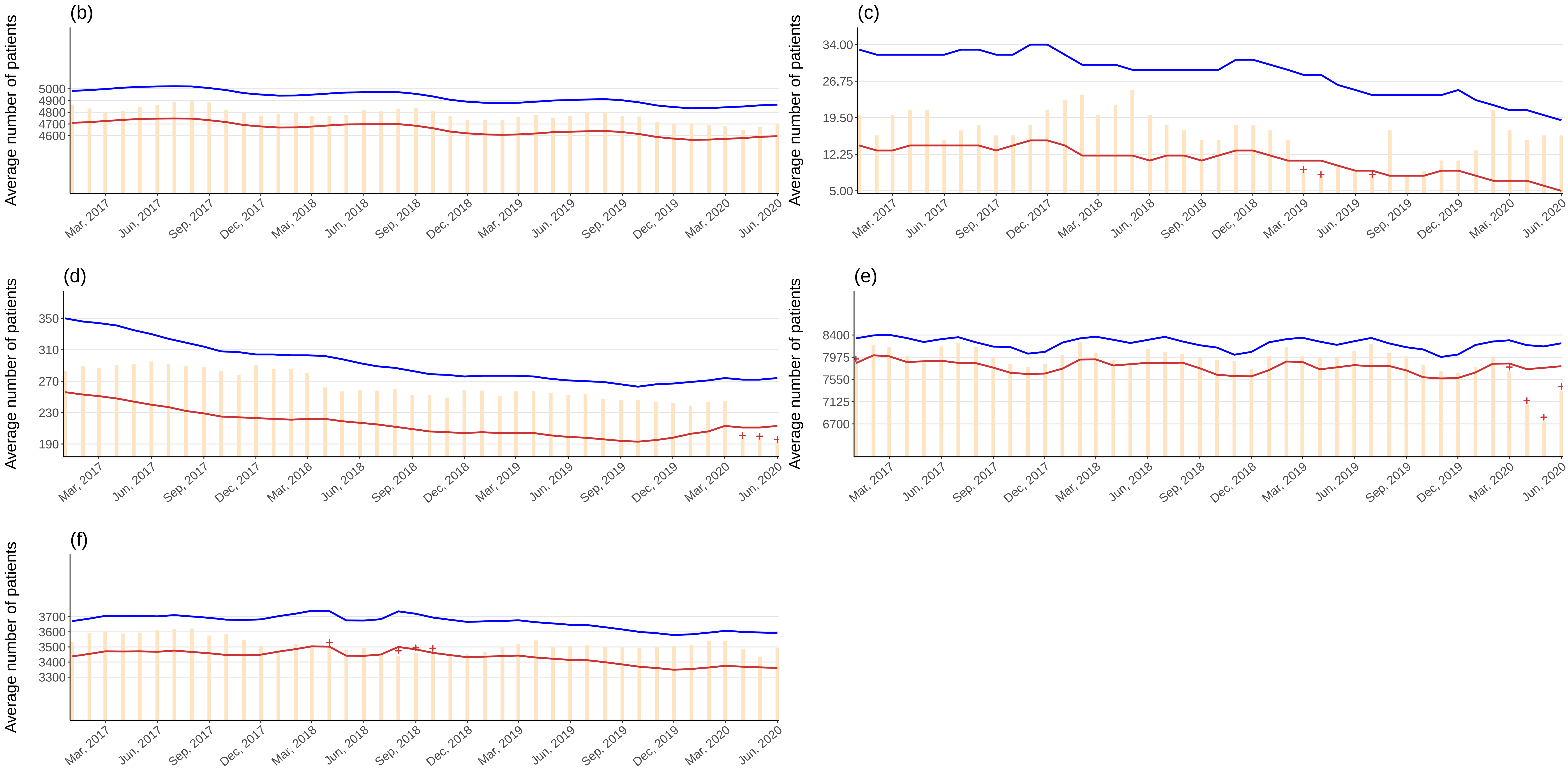

Supplement: Supplementary file 1 [file ijerph-18-03271-s001.zip › Appendix Figure 2.pdf]
