# Supplementary figures and images for: Trends in Healthcare Access in Japan during the First Wave of the COVID-19 Pandemic, up to June 2020
Source: Int J Environ Res Public Health. 2021 Mar 22;18(6):3271. doi: 10.3390/ijerph18063271 (PMC8004161; doi:10.3390/ijerph18063271)

Pearson's correlation coefficients = 0.7517

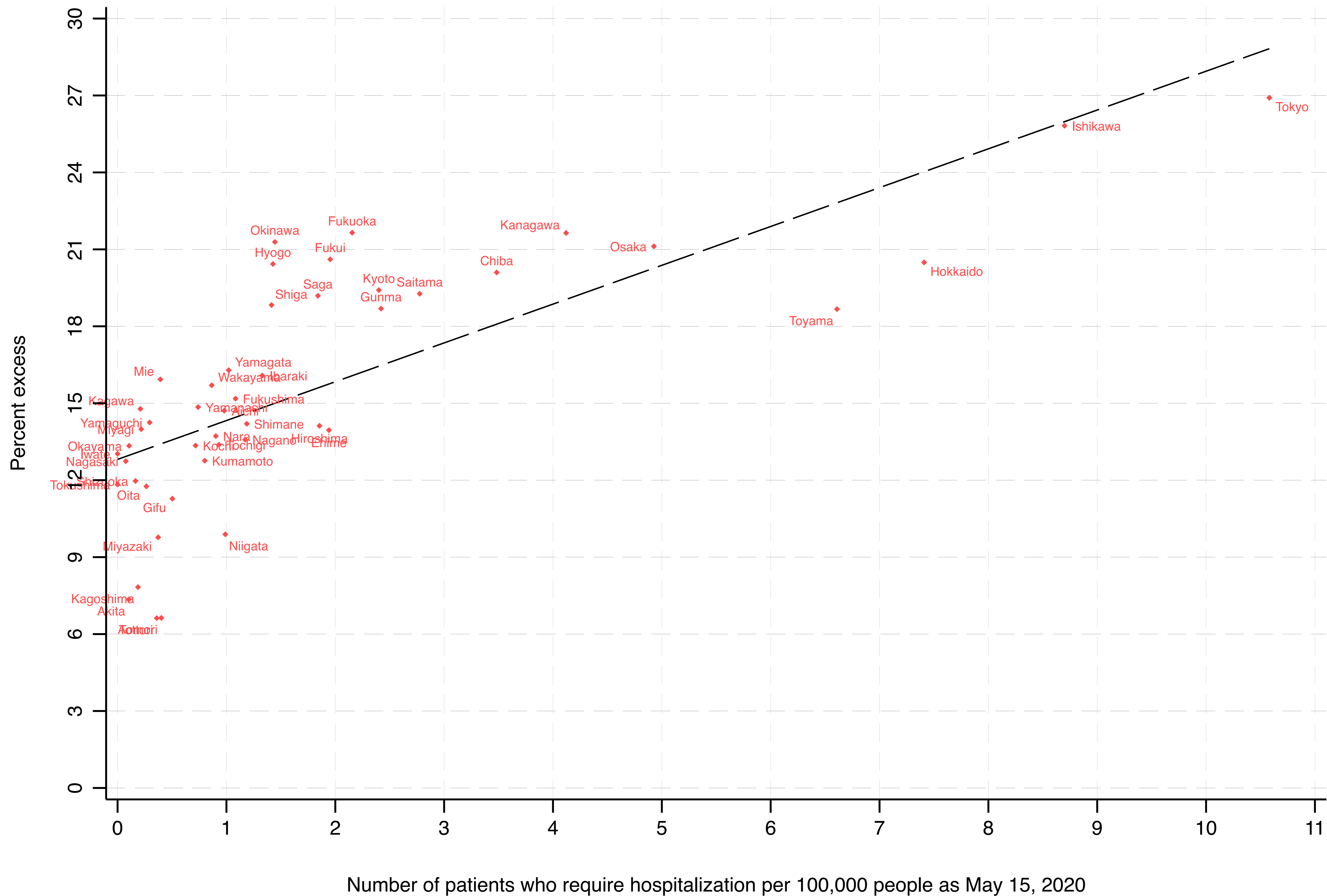

Supplement: Supplementary file 1 [file ijerph-18-03271-s001.zip › Appendix Figure 10.pdf]

Pearson's correlation coefficients = 0.8796

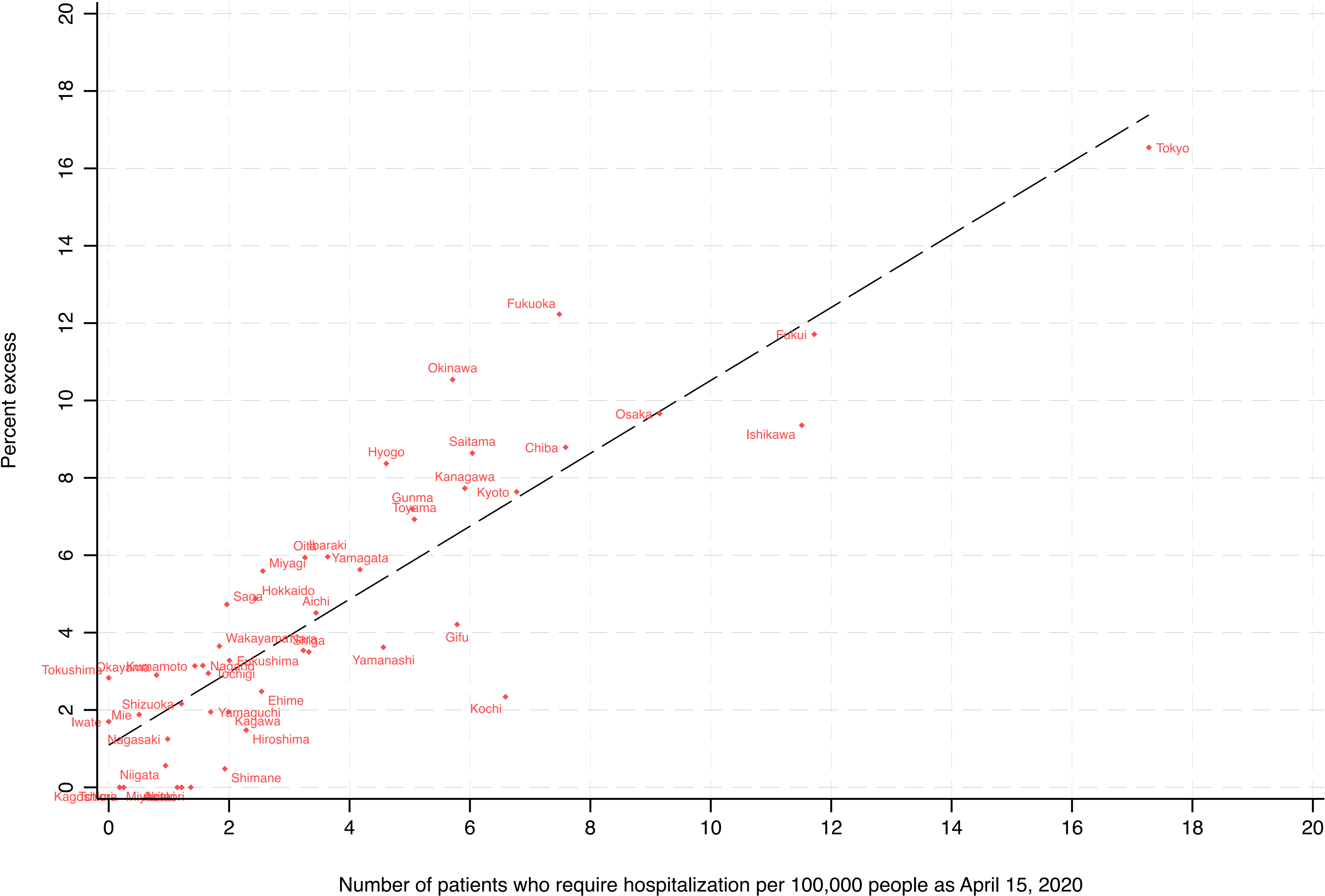

Supplement: Supplementary file 1 [file ijerph-18-03271-s001.zip › Appendix Figure 9.pdf]

Pearson's correlation coefficients = 0.8325

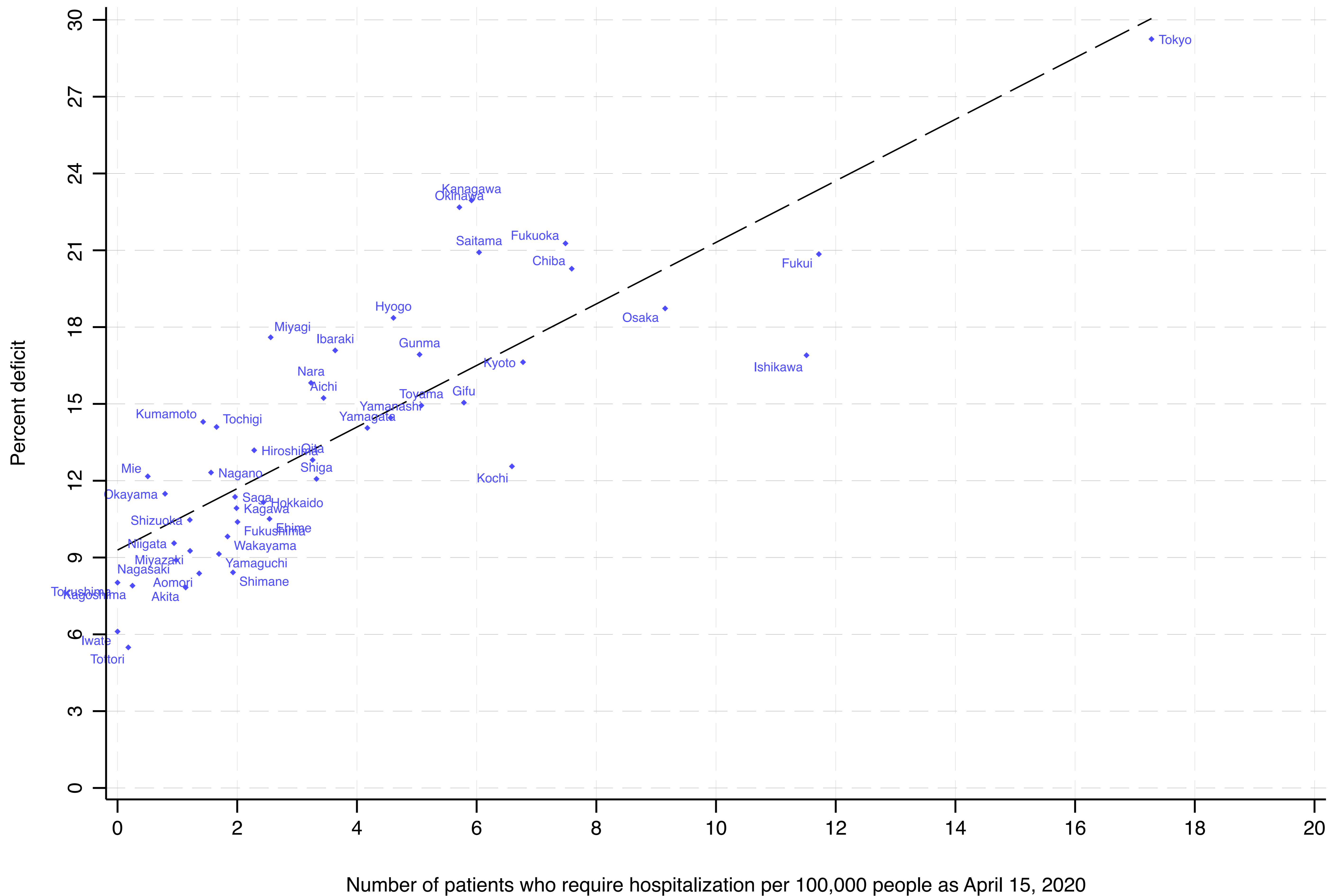

Supplement: Supplementary file 1 [file ijerph-18-03271-s001.zip › Appendix Figure 8.pdf]

Pearson's correlation coefficients = 0.8265

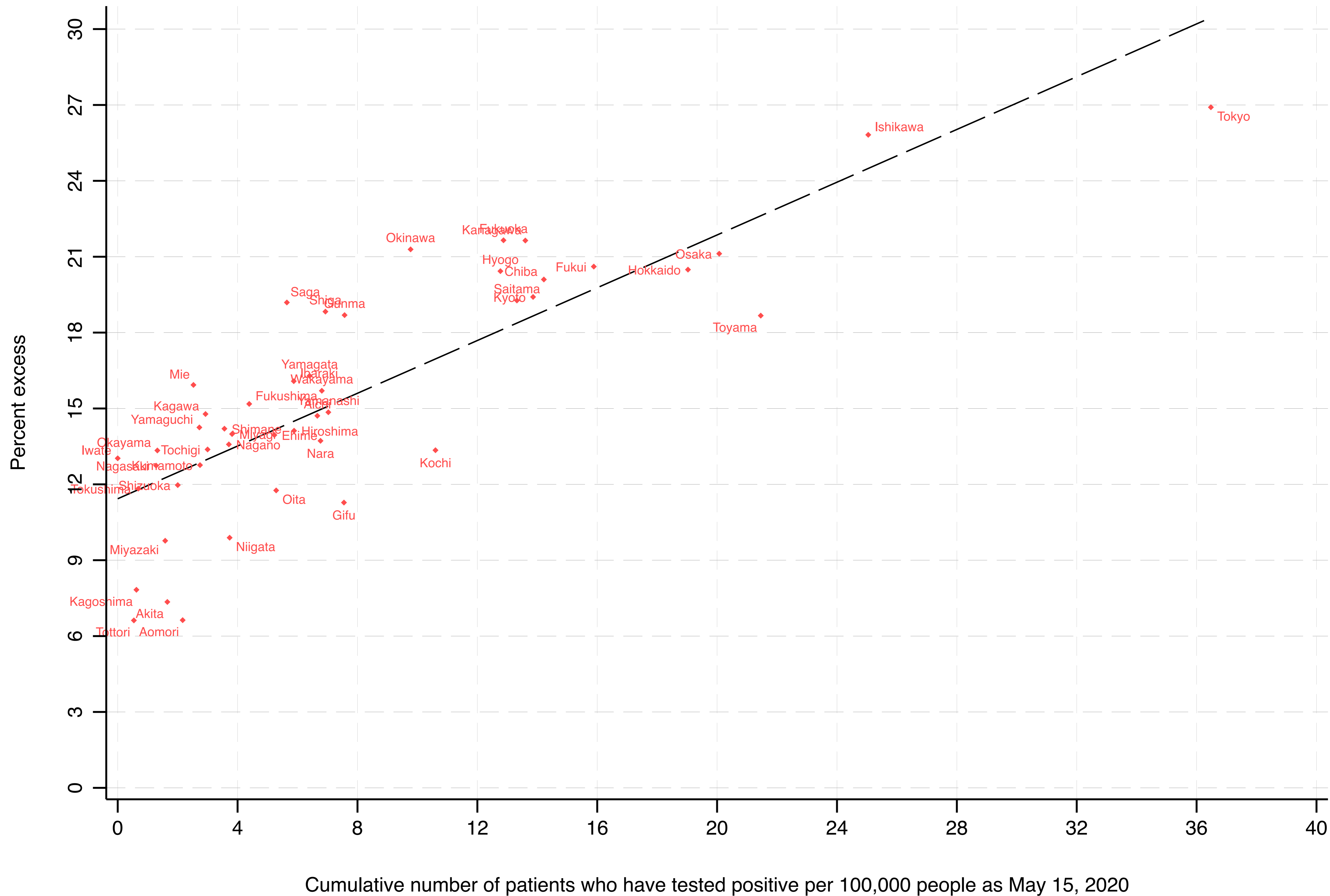

Supplement: Supplementary file 1 [file ijerph-18-03271-s001.zip › Appendix Figure 7.pdf]

Pearson's correlation coefficients = 0.8711

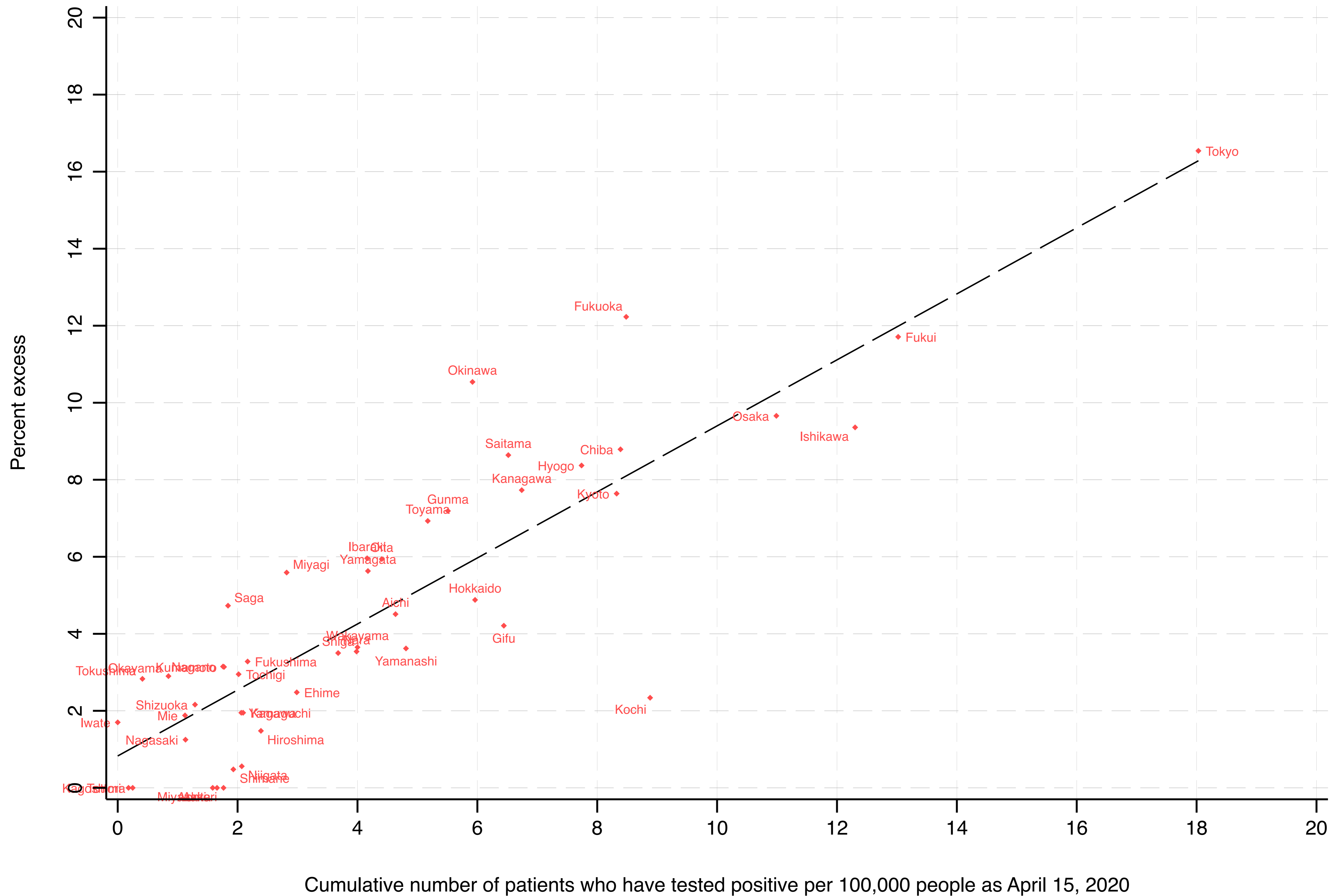

Supplement: Supplementary file 1 [file ijerph-18-03271-s001.zip › Appendix Figure 6.pdf]

Pearson's correlation coefficients = 0.7517

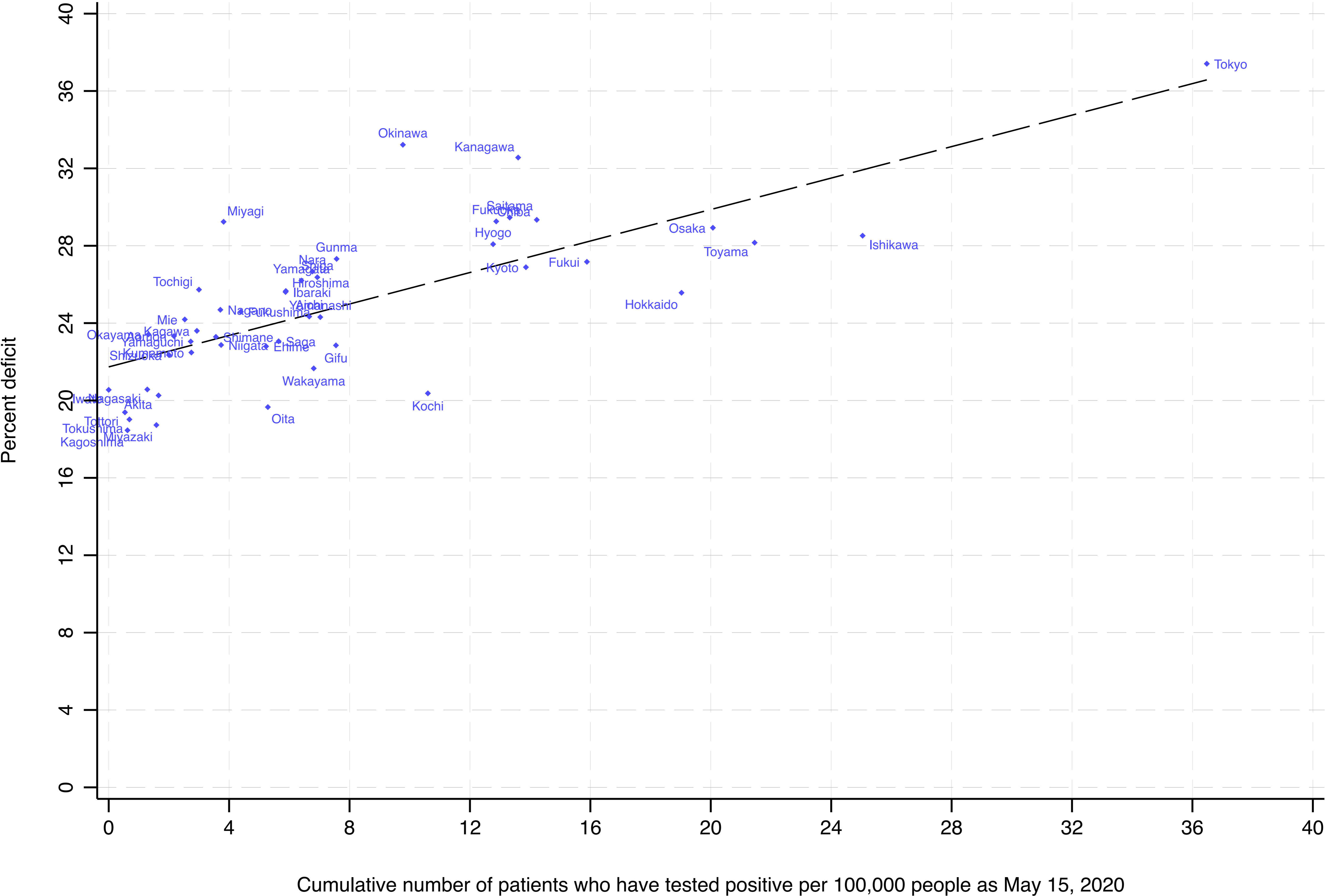

Supplement: Supplementary file 1 [file ijerph-18-03271-s001.zip › Appendix Figure 5.pdf]

Pearson's correlation coefficients = 0.8121

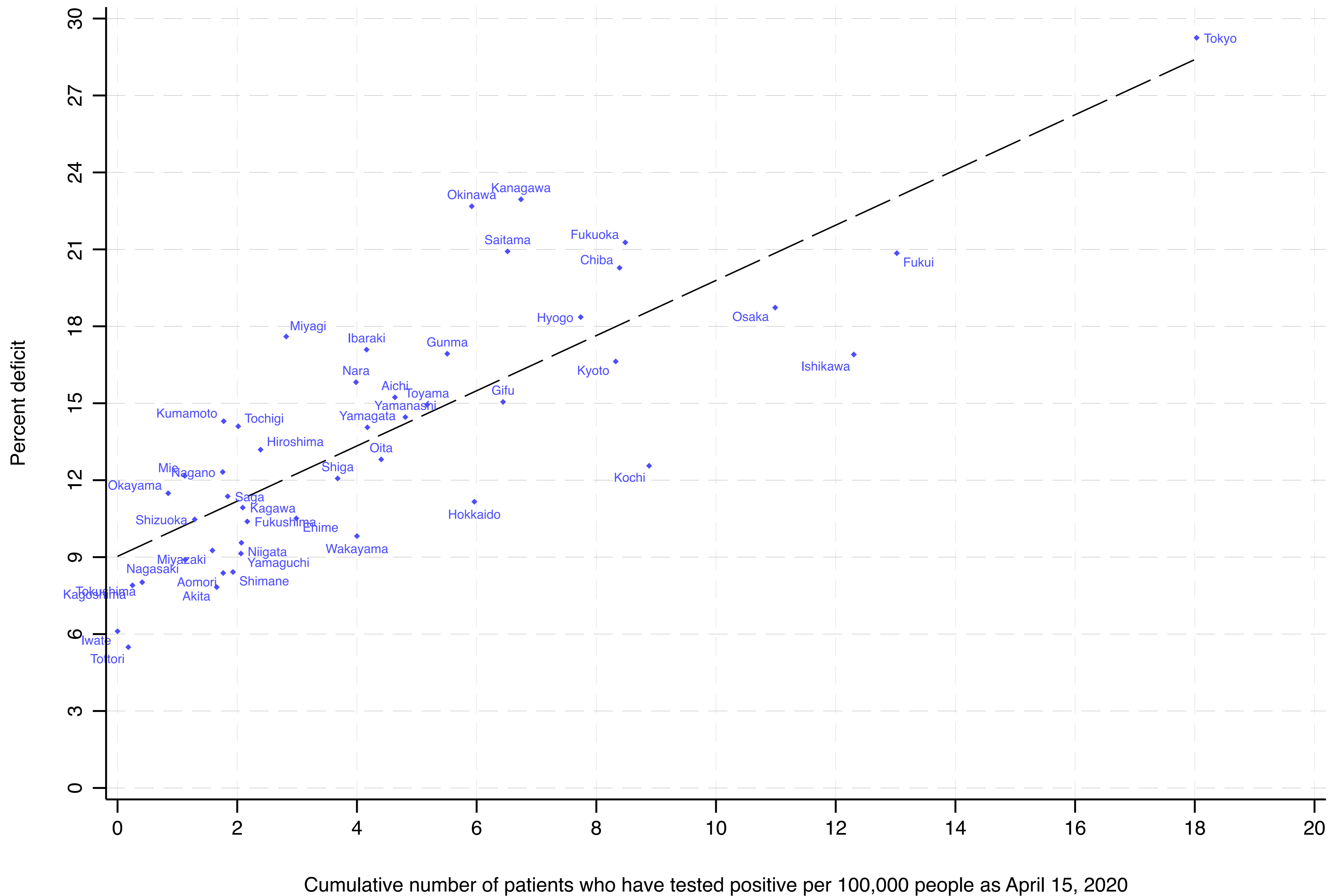

Supplement: Supplementary file 1 [file ijerph-18-03271-s001.zip › Appendix Figure 4.pdf]

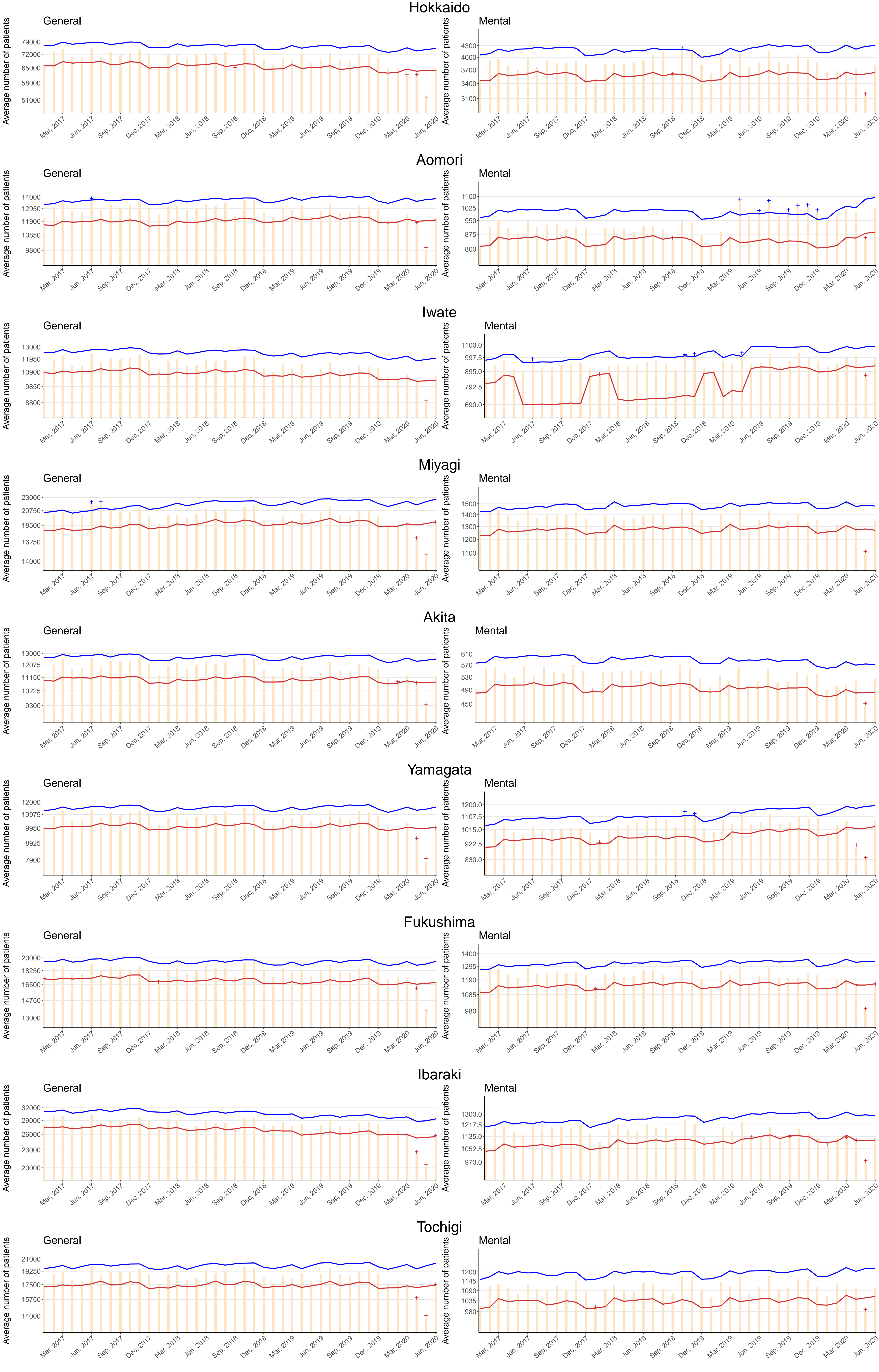



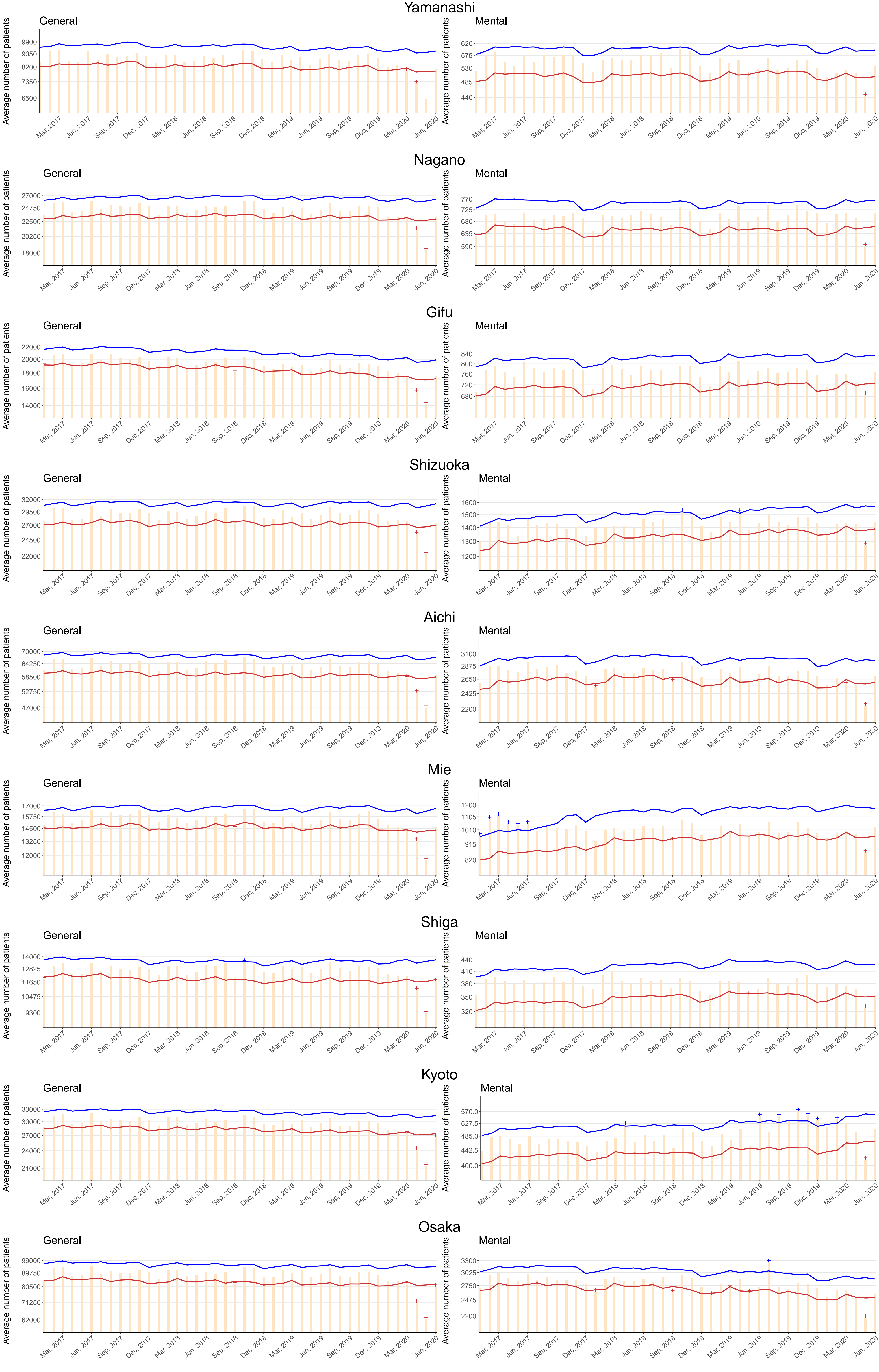

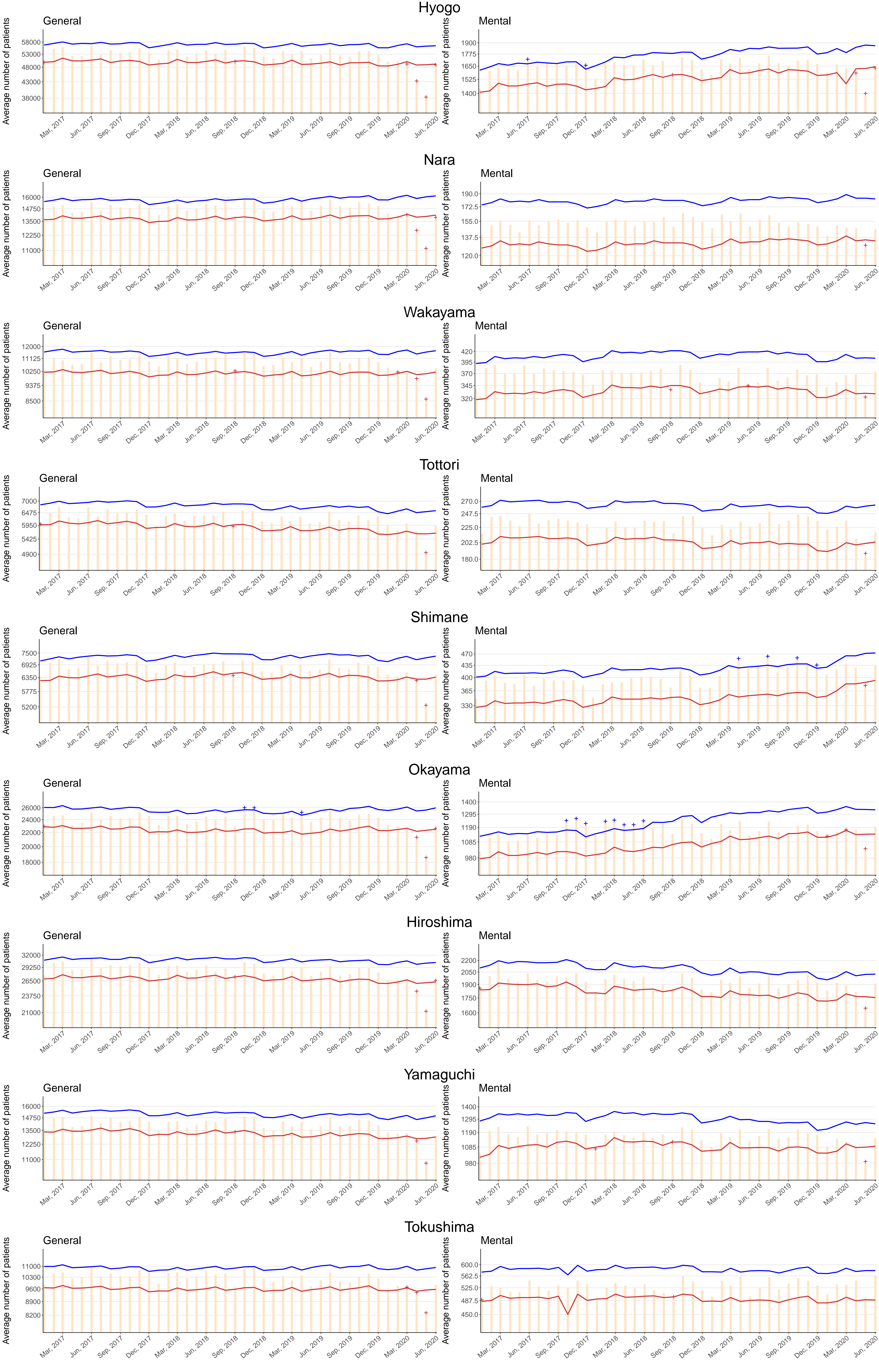

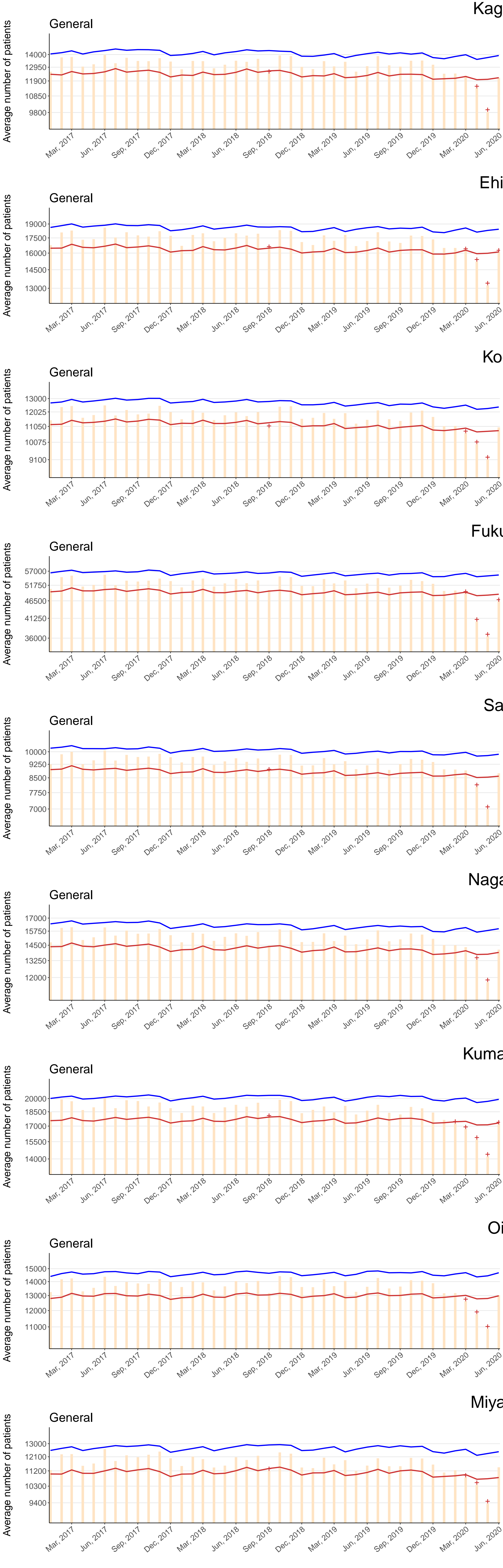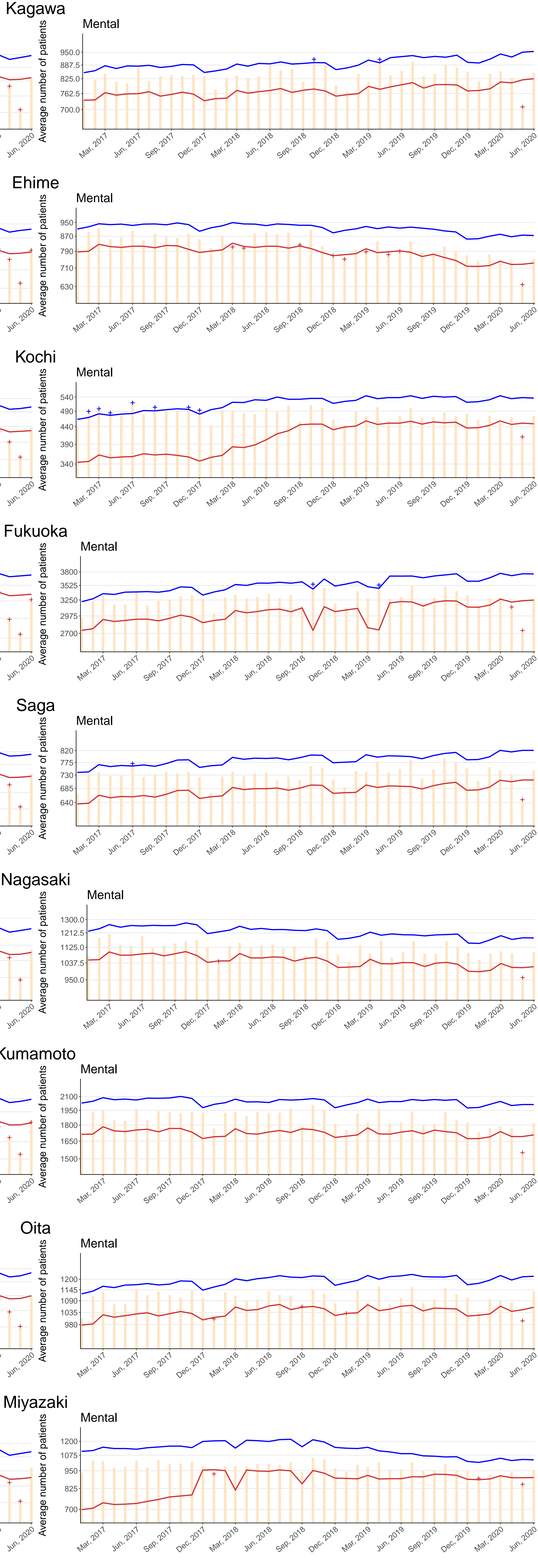

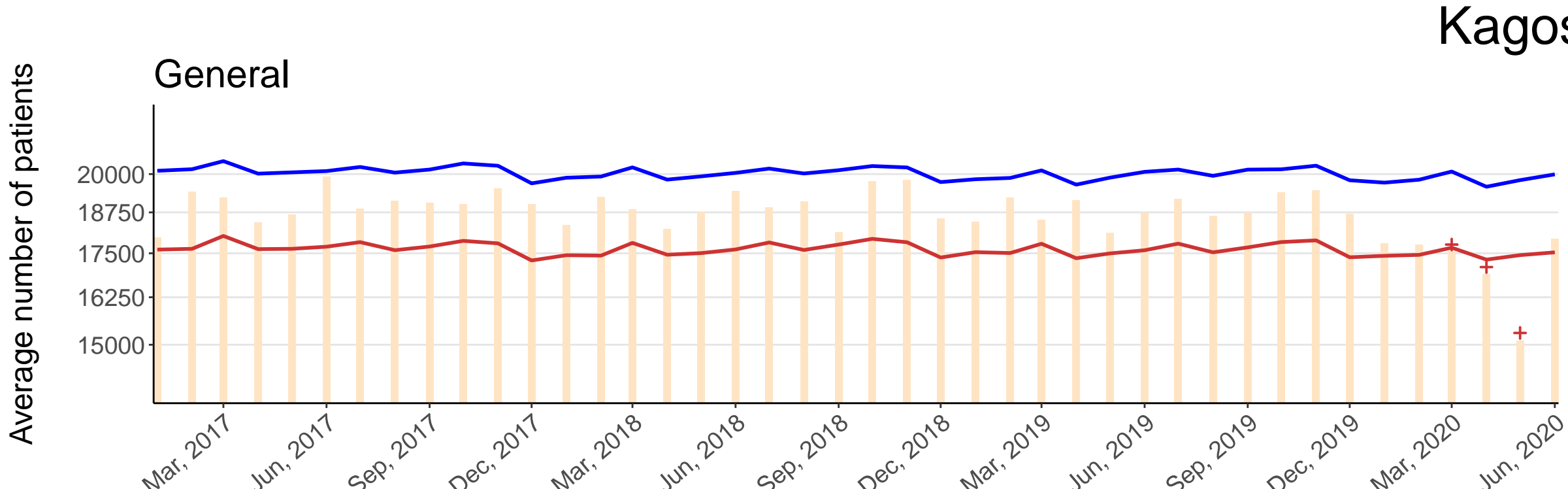

## Kagoshima

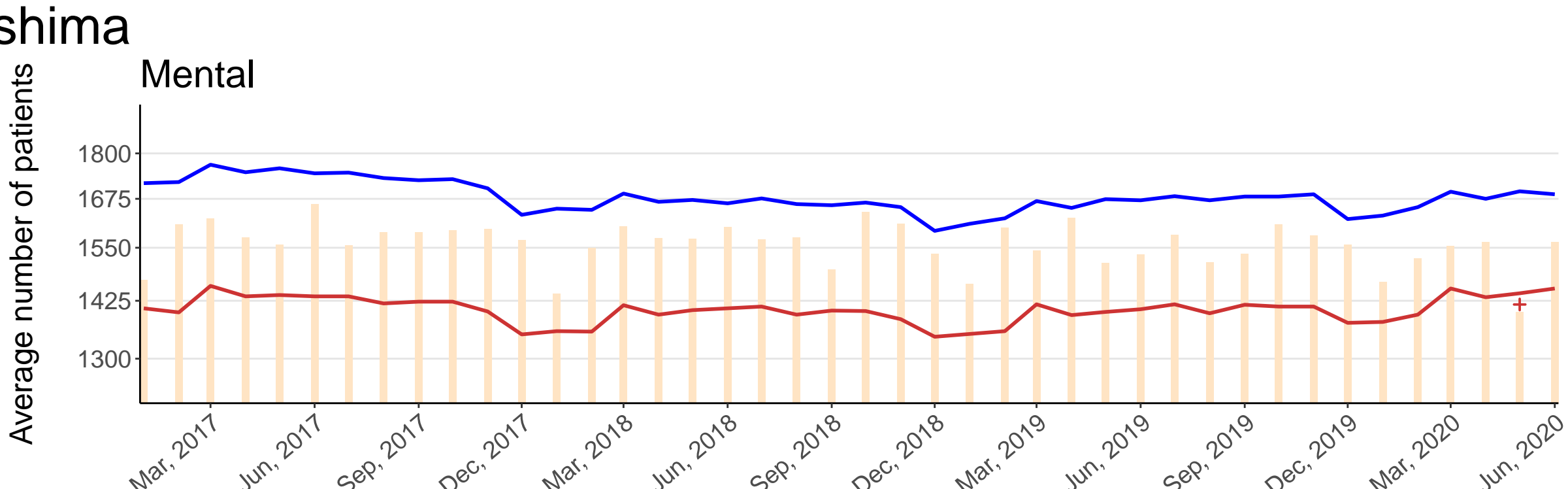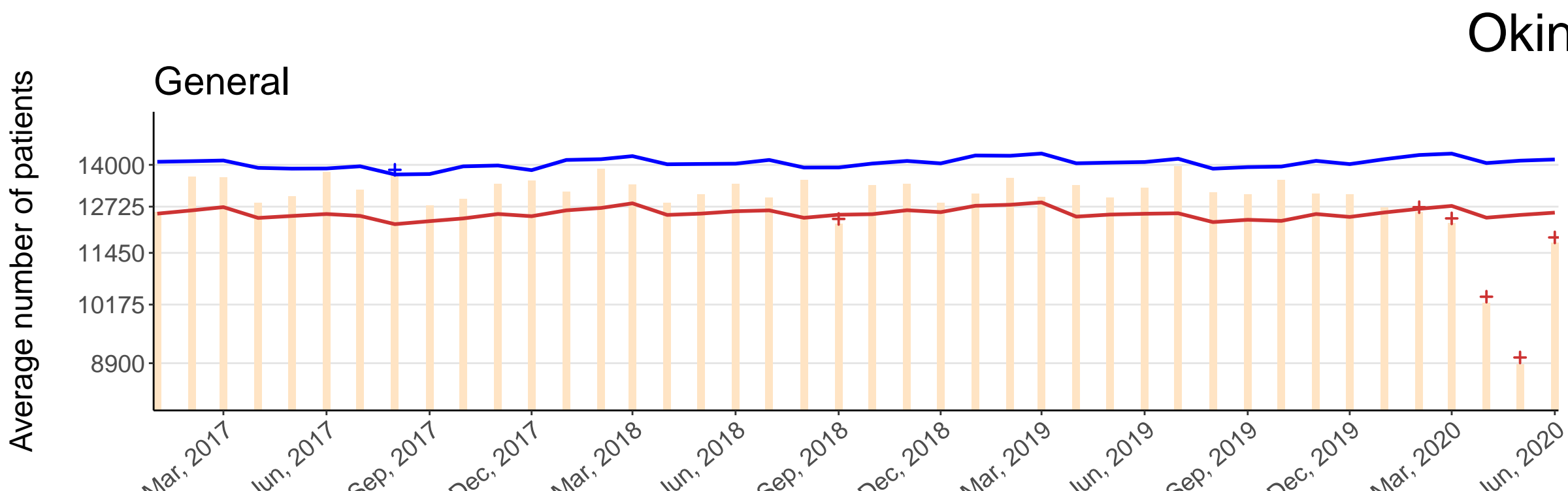

## Okinawa

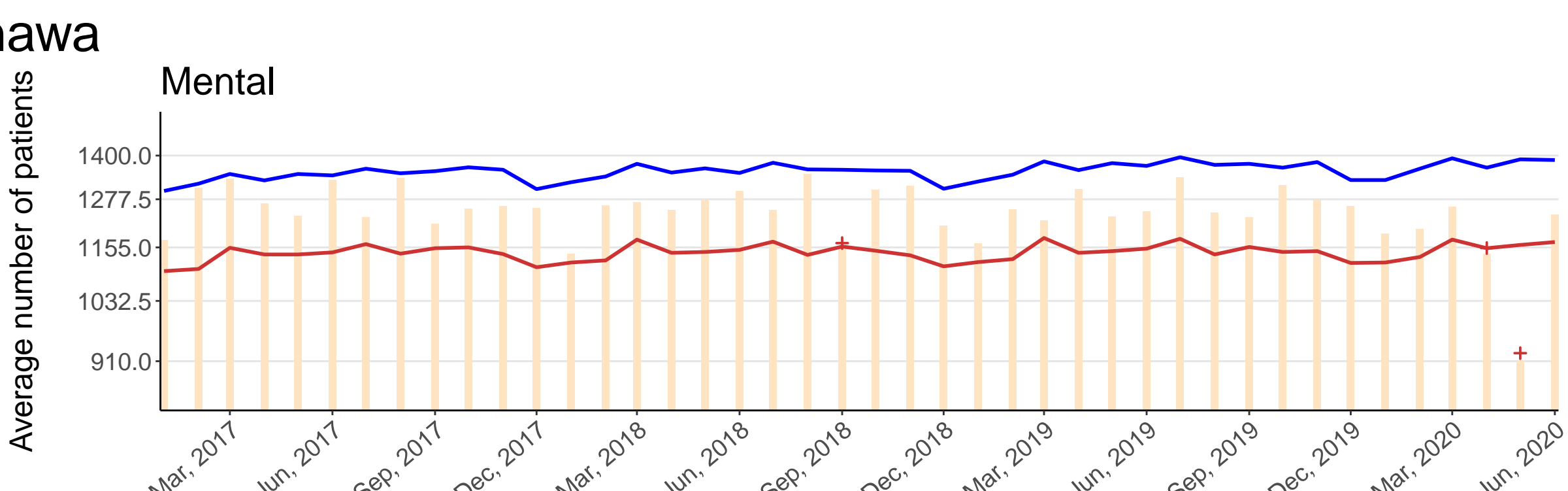

Supplement: Supplementary file 1 [file ijerph-18-03271-s001.zip › Appendix Figure 1.pdf]
